# Synthesis, neurotropic activity and docking studies of 1,2,4-triazole-linked hybrids based on 2,7-naphthyridine and bispidine rings

**DOI:** 10.1039/d6ra00302h

**Published:** 2026-07-03

**Authors:** Samvel N. Sirakanyan, Athina Geronikaki, Anush A. Hovakimyan, Anthi Petrou, Victor G. Kartsev, Hasmik A. Yegoryan, Hasmik V. Jughetsyan, Sahak P. Gasparyan, Ruzanna G. Paronikyan, Tatevik A. Araqelyan, Mariam V. Galstyan, Knarik A. Gevorkyan, Amalya D. Harutyunyan, Elmira K. Hakobyan

**Affiliations:** a Scientific Technological Center of Organic and Pharmaceutical Chemistry of National Academy of Science of Republic of Armenia, Institute of Fine Organic Chemistry of A. L. Mnjoyan 0014 Armenia anush.hovakimyan@gmail.com paronikyan.ruzanna@mail.ru; b Aristotle University of Thessaloniki, School of Pharmacy Thessaloniki 54124 Greece geronik@pharm.auth.gr; c InterBioScreen Moscow 119019 Russia vkartsev@ibscreen.chg.ru

## Abstract

A series of new 1,2,4-triazole-linked hybrids were synthesized by multistep reactions. Thus, 1-amino-3-chloro-2,7-naphthyridines were subjected to alkylation and then hydrazinolysis. The obtained carbohydrazides were converted into thioureido derivatives, which in turn were cyclized to the corresponding 5-thioxo-1,2,4-triazoles. Bispidine derivatives (3,7-diazabicyclo[3.3.1]non-3-yl(oxo)acetates) were synthesized starting from the 1,5-dialkyl-9-oxo-3,7-diazabicyclo[3.3.1]nonanes. These compounds were reacted with dimethyl oxalate and the obtained monoesters were acylated. After, the reaction of 5-thioxo-1,2,4-triazoles with 7-(chloroacetyl)-1,5-dialkyl-9-oxo-3,7-diazabicyclo[3.3.1]non-3-yl-(oxo)acetates led to the formation of aimed triazole-linked hybrids. The evaluation of neurotropic activity showed that the studied hybrid compounds exhibit pronounced anticonvulsant, anxiolytic and antidepressant properties. Some correlations between the structure and biological activity were revealed. The most active compounds in all biological tests were compounds 9c and 9k, which contain azepane fragment in their structures. Docking study is in agreement with experimental results.

## Abstract 2

A series of new 1,2,4-triazole-linked hybrids were synthesized by multistep reactions.

## Graphical abstract

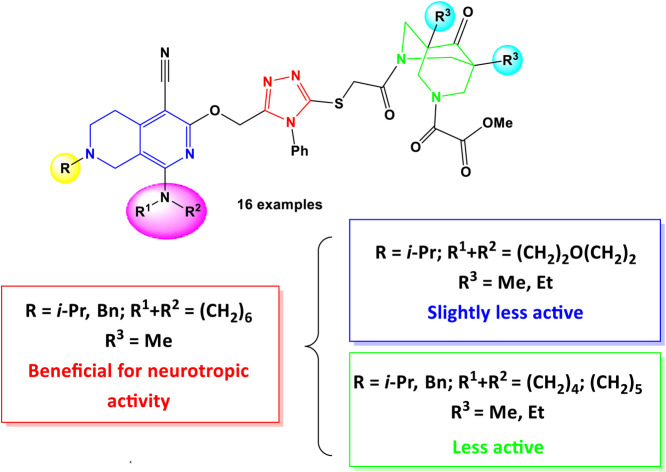

## 1 Introduction

Epilepsy is a common disease of the central nervous system (CNS), polymorphic in clinical manifestations, with an incompletely studied pathogenesis.^1^ Pharmacotherapy is the mainstay of treatment for epilepsy and symptomatic seizures. The duration and continuity of drug therapy, individual selection of the drug for each patient, the high incidence of adverse side reactions, and the prevalence of pharmacoresistant forms of the disease make epilepsy one of the most pressing problems of modern medicine.^2^ Therefore, the issue of developing new anticonvulsants with an effect on different mechanisms of the occurrence and development of convulsive conditions is urgent.^3^

**Fig. 1:**
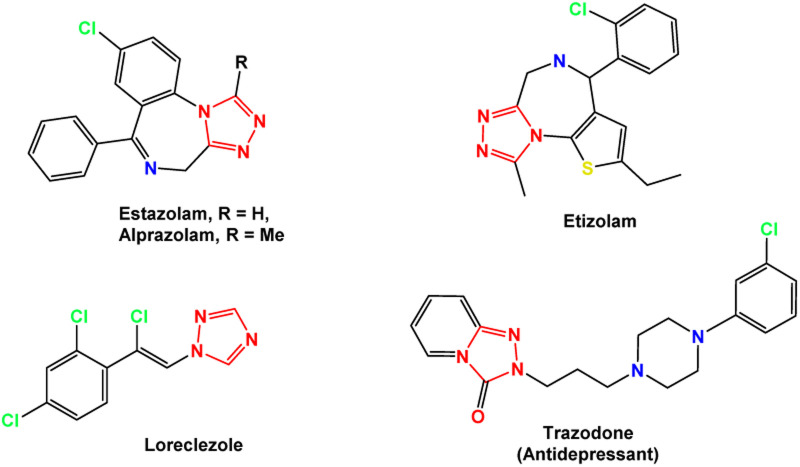
Some neurotropioc drugs containing 1,2,4-triazole ring.

The 1,2,4-triazole cycle is an important scaffold in medicinal chemistry. This moiety forms part of a variety of drugs available in clinical therapy, including anxiolytic, anticonvulsant and hypnotic (estazolam, alprazolam), anxiolytic and skeletal muscle relaxant (etizolam), antidepressant (trazodone) and anticonvulsant (loreclezole) drugs (Fig. 1).^4,5^

1,2,4-Triazole derivatives exhibited antibacterial, antifungal,^6,7^ antioxidant,^8^ anticancer,^9,10^ antiviral,^11^ antiepileptic,^12–14^ anxiolytic,^15^ antidepressant^16^ activities amongst others.^17–19^ It should be noted, that their synthetic methods, biological profile and applications were periodically discussed in reviews.^20–24^

1,2,4-Triazole-3-thione derivatives attracted a lot of interest from the scientific community due to their wide range of biological activities among which are antiviral and antiinfective,^25^ metallo-β-lactamase inhibitor,^26,27^ antimicrobial,^28,29^ analgetic,^30^ COX-2 inhibitor,^31,32^ anti-Alzheimer disease,^33^ against SARs-CoV-2,^34^ carbonic anhydrise inhibitor,^35^ antifungal,^36–38^ and anticancer.^39,40^

Furthermore, 1,2,4-triazol-3-thiones are known for their potential neurotropic activity since 1988. Nevertheless, they still attract the interest of researchers. Thus, K. Verma *et al.*^41^ synthesized fifteen 4,5-disubstituted 1,2,4-triazol-3-thione derivatives and evaluated for anticonvulsant activity with neurotoxicity determination. It was observed that the majority of the compounds exhibited activity in the MES test. In the molecular docking analysis, one compound demonstrated the strongest binding affinity, with a binding energy of −6.5 kcal mol^−1^ and a predicted inhibition constant of 17.2 µM.

M. Drabik *et al.*^42^ reported protective (antiseizure) effects of 4-butyl-5-[(4-chloro-2-methylphenoxy)-methyl]-2,4-dihydro-3*H*-1,2,4-triazole-3-thione (TPL-16) and acute neurotoxic effect in the tonic–clonic seizure model and rotarod test in mice. Compounds showed synergistic interaction with valproate and the additive interaction for TPL-16 with carbamazepine, phenobarbital and phenytoin in the tonic–clonic seizures in mice, which allow recommendation of TPL-16 as a promising agent for further experimental and clinical testing.

A. Karaküçük *et al.*^43^ design and synthesized a series of chiral 2,5-disubstituted-1,3,4-thiadiazoles and 4,5-disubstituted-1,2,4-triazole-3-thiones and evaluated activities of the new chiral compounds in several animal seizure models in mice and rats for initial (phase I) screening. All chiral compounds underwent an initial screening process involving both electrically and chemically induced acute seizure models in mice. The electrical models included the maximal electroshock seizure (MES) test and the 6 Hz psychomotor seizure model, while the chemical model employed subcutaneous pentylenetetrazol (scPTZ) to induce seizures. In addition, neurotoxicity was assessed using the rotarod performance test. Among the compounds evaluated, two compounds demonstrated strong potential as anticonvulsant agents and exhibited minimal neurotoxic effects.

B. Kapron *et al.*^44^ synthesized a series of 4-alkyl-5-substituted-1,2,4-triazole-3-thione derivatives, and their anticonvulsant activity using the maximal electroshock-induced seizure (MES) test in mice was screened.

L. Hu *et al.*^45^ designed and synthesized twenty compounds bearing triazole structural fragments and evaluated their anticonvulsant activity and neurotoxicity using several tests such as MES, subcutaneous pentylenetetrazole (scPTZ), and rotarod (ROT) tests *in vivo* animal models. Among these compounds, *N*-(4-(1*H*-1,2,4-triazol-1-yl)phenyl)-3-fluorobenzamide and *N*-(4-(1*H*-1,2,4-triazol-1-yl)phenyl)-3-methylbenzamide exhibited ED_50_ values of 13.1 and 19.7 mg kg^−1^, respectively, with protective index (PI) values of 45.9 and 22.1. Notably, *N*-(4-(1*H*-1,2,4-triazol-1-yl)phenyl)-3-fluorobenzamide demonstrated strong affinity for the GABAA receptor, with an IC_50_ value of 0.14 µM.

Furthermore, several review articles have reported that 1,2,4-triazole derivatives possess significant potential as anti-seizure agents.^22,46^

**Fig. 2:**
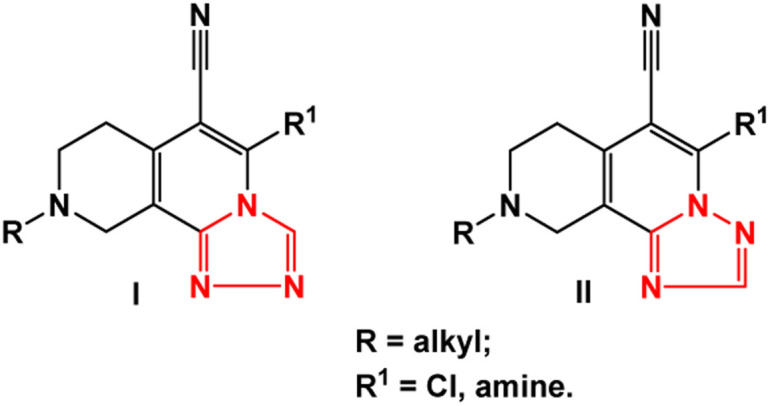
Previously synthesized condensed isomeric 1,2,4-triazoles.

In our previous studies^47,48^ we have synthesized 1,2,4-triazoles condensed with 2,7-naphthyridine ring, which is known to be a heterocycle with pronounced biological activities.^49–52^ The studies revealed that some of these triazolo[3,4-*a*]-2,7-naphthyridines I and triazolo[5,1-*a*]-2,7-naphthyridines II showed high neurotropic activity^47^ (Fig. 2).

Bispidine(3,7-diazabicyclo[3.3.1]nonanes) derivatives are biologically active molecules, also found in the structure of several alkaloids with biological importance.^53^ The works in this field are very diverse – from NMR^54^ to biological activity^55–60^ studies. It is also known, that bispidine derivatives are promising catalyzing ligands for some reactions.^61–64^

Molecular hybridization which combines two or more pharmacophores to develop new active molecules has been used to identify new drugs for treating many diseases, including cancer, malaria, and tuberculosis.^65,66^

Thus, the synthesis of hybrid compounds, which combine in their structure these three heterocycles, is highly interesting work, both in terms of the organic chemistry and in terms of the search for new biologically active compounds. In this regard, this work is an effort to synthesize novel 1,2,4-triazole-linked hybrids in search for new compounds with promising biological activity.

## 2 Results and discussion

### 2.1. Chemistry

**Scheme 1:**
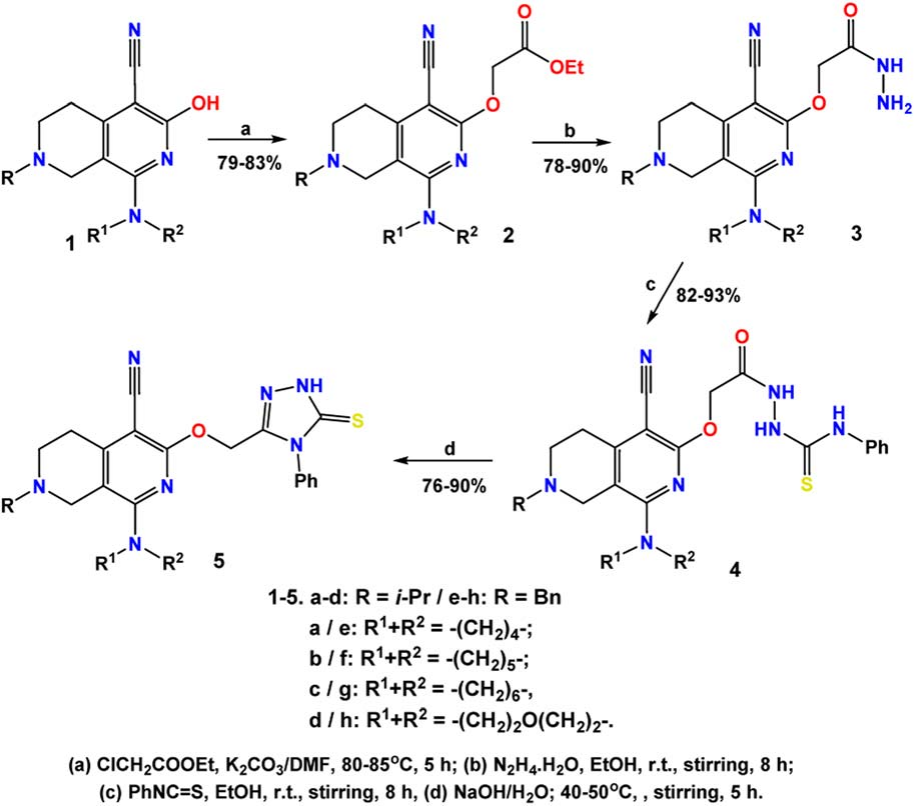
Synthesis of 3-(4-phenyl-5-thioxo-1,2,4-triazol-3-yl)-2,7-naphthyridines 5.

The starting 1,2,4-triazole-5-thiones 5a–h were synthesized using the procedures mentioned in the literature.^67^ Thus, 7-alkyl-1-amino-3-hydroxy-5,6,7,8-tetrahydro-2,7-naphthyridine-4-carbonitriles 1a–h^68,69^ were alkylated with ethyl chloroacetate in basic conditions, and as a result of the reactions the relevant *O*-alkylated derivatives 2a–h^68^ were obtained in high yields (Scheme 1).

Then, compounds 2 were subjected to hydrazinolysis with formation of carbohydrazides 3a–h. These compounds in turn upon reaction with phenylisothiocyanate in absolute ethanol were converted into the corresponding thioureido derivatives 4a–h. Further, the intramolecular cyclization of compounds 4a–h in aqueous solution of sodium hydroxide under heating at 40–50 °C resulted 7-alkyl-1-amino-3-[(4-phenyl-5-thioxo-4,5-dihydro-1*H*-1,2,4-triazol-3-yl)methoxy]5,6,7,8-tetrahydro-2,7-naphthyridine-4-carbonitriles 5a–h in high yields (Scheme 1). In the ^1^H NMR spectra of the synthesized 1,2,4-triazoles 5 the singlet signal of NH group was appeared at 13.77–13.87 ppm.

**Scheme 2:**
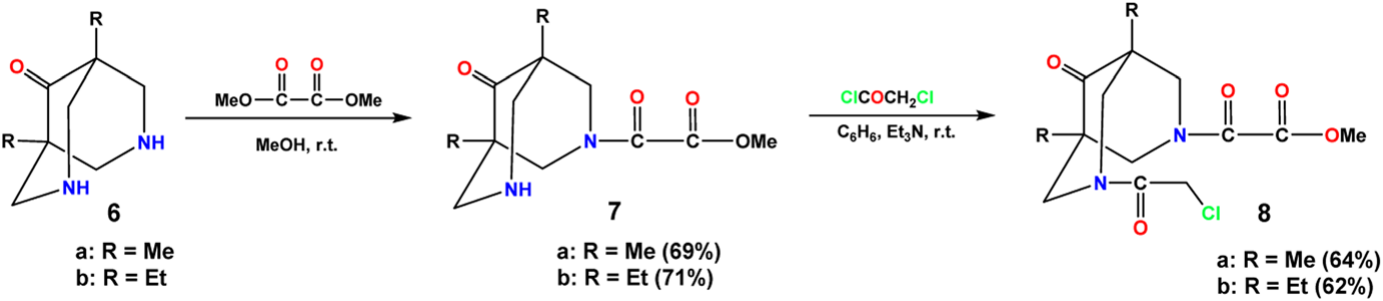
Synthesis of 7-(chloroacetyl)-1,5-dialkyl-9-oxo-3,7-diazabicyclo[3.3.1]non-3-yl-(oxo)acetates 8.

The second initial compounds – 7-(chloroacetyl)-1,5-dialkyl-9-oxo-3,7-diazabicyclo[3.3.1]non-3-yl-(oxo)acetates 8a,b were synthesized according to the Scheme 2. 1,5-Dialkyl-9-oxo-3,7-diazabicyclo[3.3.1]nonanes 6a,b^70^ were reacted with dimethyl oxalate in absolute methanol at room temperature thus resulting to monoesters of 3,7-diazabicyclo[3.3.1]nonane 7a,b. Compounds 7 in turn were acylated with chloroacetyl chloride in benzene at room temperature and the aimed 7-(chloroacetyl)-1,5-dialkyl-9-oxo-3,7-diazabicyclo[3.3.1]non-3-yl-(oxo)acetates 8a,b were obtained in moderate yields (Scheme 2).

**Scheme 3:**
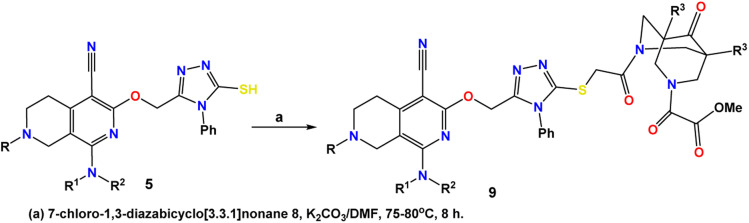
Synthesis of aimed 1,2,4-triazole-linked hybrids 9a–p.

Finally, the obtained 1,2,4-triazole-5-thiones 5a–h were stirred with compounds 8a,b at 75–80 °C in potassium carbonate/dimethylformamide (K_2_CO_3_/DMF) medium for 8 h. As 1,2,4-triazole-5-thiones 5 are ambident systems, therefore in these compounds the thilactam–thiolactim tautomerism can be exists *via* the transfer of a hydrogen atom between the two basic centers. Consequently, the alkylation of these heterocycles in basic conditions could give the formation of *S*- or *N*-alkylated isomers or their mixture.^71–73^ However the studies revealed that alkylation of compounds 5 exclusively took place at the most nucleophilic sulfur center^71–73^ with formation of *S*-alkylating products. The aimed 1,2,4-triazole-linked hybrids 9a–p were synthesized in high yields (Scheme 3 and Table 1).

**Table 1:** The structure of disubstituted 1,2,4-triazoles 9a–p

No.	1,2,4-Triazole-linked hybrids	Yield, %	No.	1,2,4-Triazole-linked hybrids	Yield, %
9a	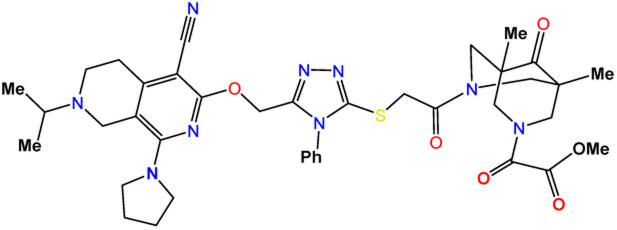	74	9i	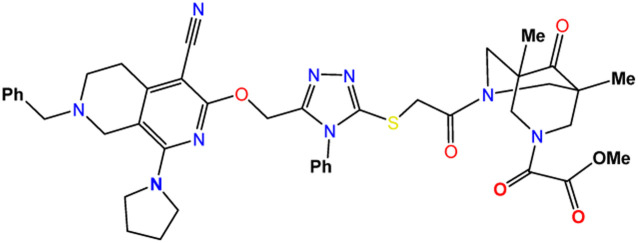	75
9b	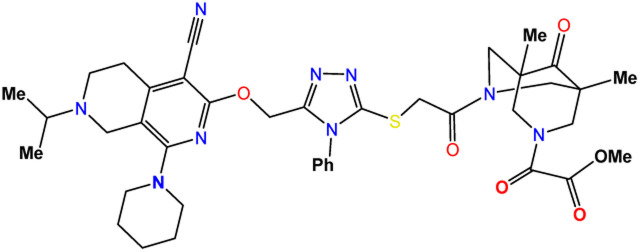	71	9j	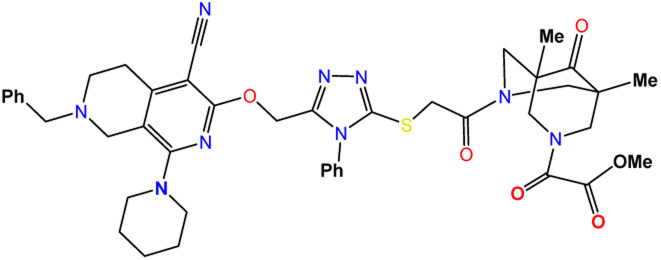	73
9c	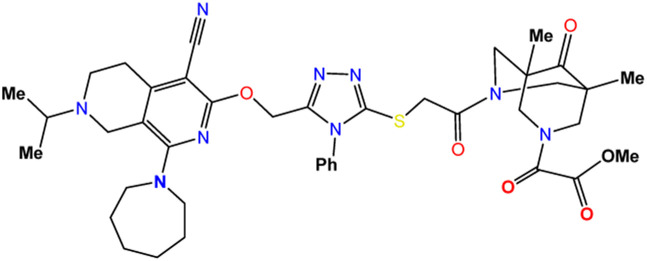	77	9k	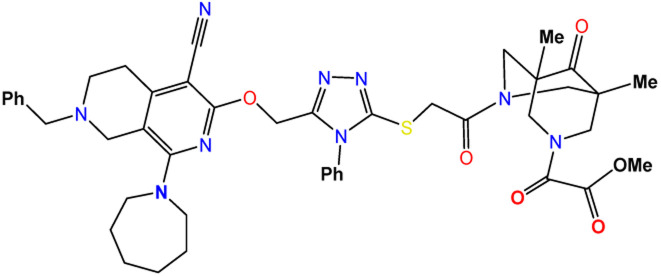	76
9d	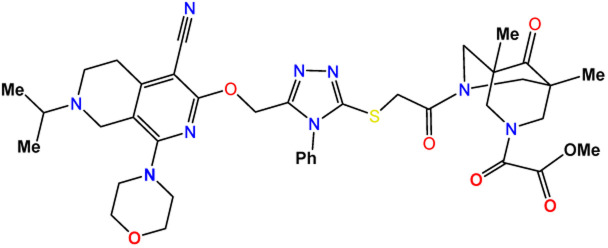	73	9l	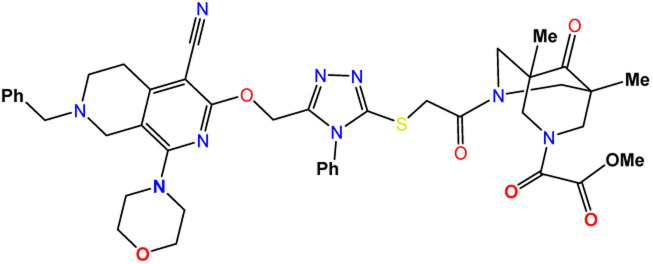	71
9e	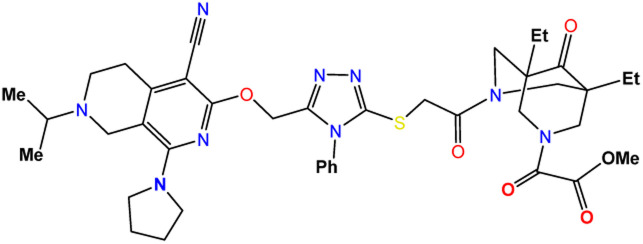	70	9m	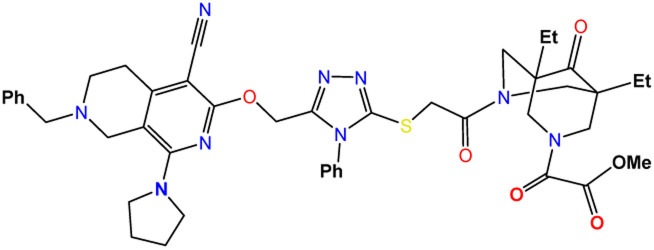	72
9f	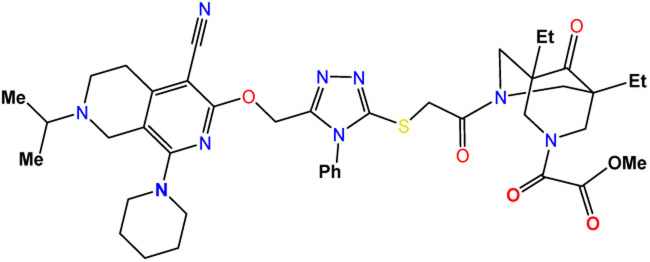	74	9n	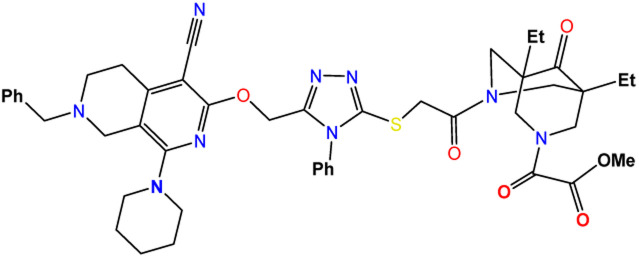	74
9g	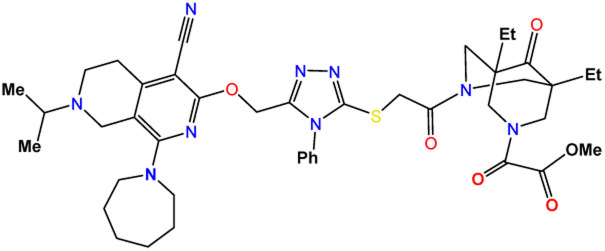	72	9o	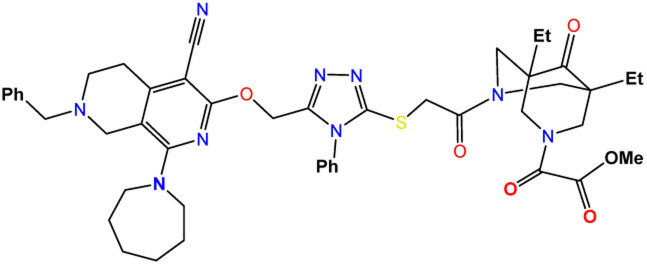	77
9h	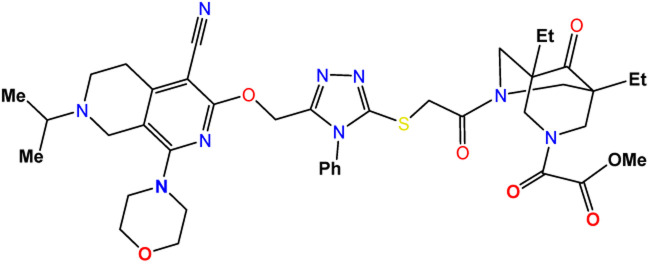	71	9p	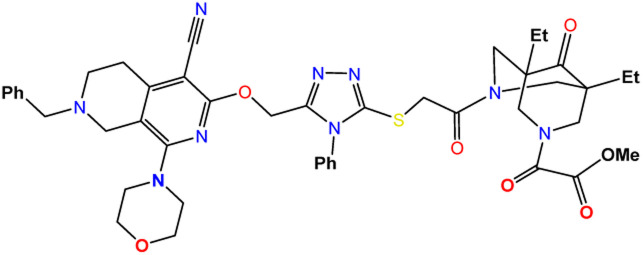	75

The structure of obtained hybrids 9a–p was confirmed by NMR, IR spectroscopy, MS spectrometry and by elemental analysis. All spectra were corresponded to the structure of compounds.

### 2.2. Biological evaluation

#### 2.2.1. Evaluation of anticonvulsant activity

The study of the neurotropic activity of new 16 synthesized compounds 9a–p was carried out according to indicators characterizing anticonvulsant, sedative, anti-anxiety antidepressant activity and side effects. The anticonvulsive action of the tested compounds was assessed by evaluating the antagonism to the convulsive pentylenetetrazole (PTZ), thiosemicarbazide (TSC) seizures.^74–78^ PTZ induced test is considered an experimental model for the clonic component of epilepsy seizures and prognostic anxiolytic activities of the compounds. The mechanism of convulsive action of PTZ is due to its inhibitory effect on the GABA-A site. The thiosemicarbazide (TSC) seizures indicate effects on GABA metabolism. Ethosuximide and the tranquilizer diazepam were used as a control.^79^ We selected ethosuximide as an analog with anticonvulsant activity, as our compounds demonstrate agonist activity with pentylenetetrazol, similar to ethosuximide. Diazepam, a clinically used psychotropic drug, shares similar effects with the compounds we investigated. Essentially, we used functional analogs that are employed in medicine to compare the data obtained from our compounds.

The side effects of the compounds-neurotoxicity (movement coordination disorder, myorelaxation and ataxia) with the test of rotating rod^78,80^ and maximal tolerated dose (MTD) were also studied on mice. To determine the 50% effective (ED_50_, causing the anticonvulsant effect of 50% of animals, is calculated by the test antagonizm to PTZ), 50% neurotoxic (TD_50_, causing myorelaxation in 50% of animals) doses a statistical method of probit analysis by Litchfield and Wilcoxon was used.^78,79^

The evaluation of the anticonvulsant activity of all synthesized compounds revealed that they exhibited PTZ antagonism to varying degrees. Compounds 9a–p, administered at a dose of 50 mg kg^−1^, prevented PTZ-induced clonic seizures in 40–80% of the animals. Intraperitoneal injection of these compounds in mice, starting at a dose of 25 mg kg^−1^, was associated with the prevention of PTZ-induced seizures, and the ED_50_ values ranged from 15.0 to 41.0 mg kg^−1^ (Table 2). The results demonstrated that the studied compounds exceeded the activity of ethosuximide in PTZ-induced seizures test but inferior to diazepam. The effective dose (ED_50_) of ethosuximide in antagonizing PTZ-induced seizures in mice was 155.0 mg kg^−1^, whereas that of diazepam was 0.5 mg kg^−1^ (Table 2). Unlike diazepam, however, the studied compounds did not induce muscle relaxation at the tested doses (12.5, 50, and 200 mg kg^−1^). They also exhibited low toxicity, with acute LD_50_ values exceeding 500–900 mg kg^−1^. Ethosuximide, at doses of 100–200 mg kg^−1^ in mice, likewise did not cause muscle relaxation.

**Table 2:** Anticonvulsant activity and toxicity of compounds 9a–p compared to standard drugs

Compound	ED_50_^a^ (mg kg^−1^) (by PTZ antagonism)	TD_50_^a^ (mg kg^−1^)	LD_50_^a^ (mg kg^−1^)	Latency of convulsions induced by TSC, min	
				*M* ± *m*	*I* b
Control	—	—	—	55.4 ± 3.0	1.0
9a	40	>200	>400	75.2 ± 7.0	1.38
9b	40	>200	>500	60.1 ± 7.0	1.1
9c	15.0 (12.5 ÷ 18.0)	>200	>500	69.4 ± 4.0	1.25
9d	21.0 (17.5 ÷ 25.2)	>200	>600	87.6 ± 5.0	1.58
9e	41.0 (25.6 ÷ 61.5)	>200	>600	70.6 ± 5.3	1.27
9f	40	>200	>600	59.8 ± 5.9	1.1
9g	22.0 (19.1 ÷ 25.3)	>200	>600	67.6 ± 7.0	1.22
9h	17.5 (13.5 ÷ 22.75)	>200	>500	86.8 ± 5.0	1.57
9i	25.5 (16.0 ÷ 40.8)	>200	>600	54.7 ± 12	0.98
9j	35.0 (22.6 ÷ 54.3)	>200	>600	62 ± 5.08	1.12
9k	18.5 (15.4 ÷ 22.2)	>200	>600	68.0 ± 4.5	1.23
9l	34.5 (21.6 ÷ 55.2)	>200	>700	98.4 ± 10	1.78
9m	40	>200	>600	56.4 ± 4.4	1.02
9n	22.0 (16.9 ÷ 28.6)	>200	>700	83.8 ± 6.5	1.5
9o	40	>200	>500	100.8 ± 9	1.82
9p	28.0 (20.0 ÷ 39.2)	>200	>800	116.0 ± 9.9	2.1
Ethosuximide (200 mg kg^−1^)	155 (117.5 ÷ 204.5)	520 (413 ÷ 655)	1325 (1200 ÷ 1462)	68.0 ± 6.2	1.22
Diazepam (2 mg kg^−1^)	0.5 (0.4 ÷ 0.7)	2.7 (1.4 ÷ 5.5)	180 (128 ÷ 252.0)	65.0 ± 3.5	1.17

a *P* = 0.05 at a probability level.

b *I* – an increase in the threshold.

As shown in Table 2, in the TSC model, most of the studied compounds prevented TSC-induced seizures in a manner comparable to diazepam and ethosuximide, with compounds 9l, 9o, and 9p exhibiting a more pronounced effect (up to 2.1-fold).

Among the studied compounds, the most active are: 9c, 9h, 9k, 9d, 9g and 9n. The order of anticonvulsant activity of compounds can be presented as follows: 9c > 9h > 9k > 9d > 9g = 9n > 9i > 9p > 9l > 9j > 9a = 9b = 9f = 9m > 9o > 9e. The structure–activity relationship on anticonvulsant activity revealed that these compounds 9c, 9h, 9d, 9g, besides 9k, in the 7th position of 2,7-naphthridine ring contain isopropyl group, and the first position is substituted by azepane or morpholine rings. The comparison of the pairs a–i, b–j, c–k, d–l, e–m, f–n, g–o and h–p revealed that in case of d–l, h–p and g–o the presence of isopropyl group at position 7 was beneficial. In the remaining pairs, except for c–k, which exhibited comparable activity, the presence of a benzyl group was more favorable. The nature of the R^3^ substituent in the 3,7-diazabicyclo[3.3.1]nonane ring generally did not significantly influenced anticonvulsant activity.

The psychotropic properties of the compounds were also investigated. Compounds 9a–p were evaluated using the open field, Elevated Plus Maze (EPM), forced swimming, and tail suspension tests at a dose of 50 mg kg^−1^, as the ED_50_ values of these compounds fall within this dose range at the confidence interval.

**Fig. 3:**
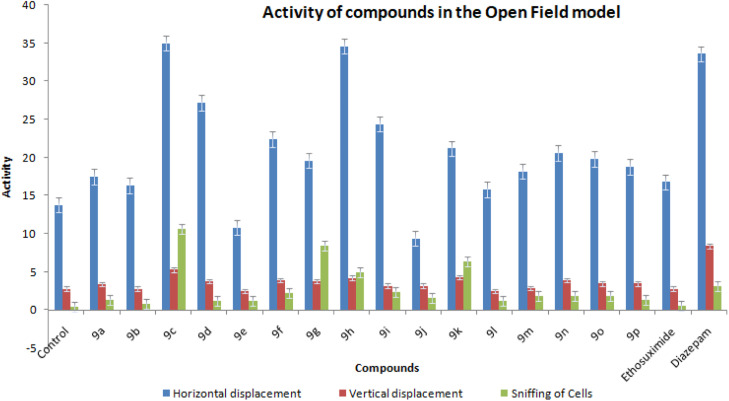
Graphical depiction of the behavioral activity of compounds 9a–p, ethosuximide, and diazepam in the open field test.

Locomotor activity was assessed using the open-field model by evaluating both horizontal and vertical movements of the animals. The effects of the compounds were recorded and analyzed, allowing the identification of both activating and sedative responses. In the open field behavioural model,^83–86^ rats in the control group exhibited mean values of 13.8 horizontal movements, 2.8 vertical movements, and 0.4 examined cells (Table 3 and Fig. 3). Administration of the studied compounds resulted in changes in behavioural parameters compared with the control group, demonstrating marked alterations in both horizontal and vertical locomotor activity.

**Table 3:** Assessment of the effects of compounds 9a–p on rat behavior in the open field test

Compound	Amount (absolute data during 5 min)^a^		
	Horizontal displacement	Vertical displacement	Sniffing of cells
Control	13.8 ± 2.5	2.8 ± 0.3	0.4 ± 0.02
9a	17.5 ± 3.8	3.4 ± 0.7	1.3 ± 0.1^b^
9b	16.3 ± 2.8	2.8 ± 0.5	0.8 ± 0.2^b^
9c	35.0 ± 4.0^b^	5.3 ± 0,6^b^	10.6 ± 2.7^b^
9d	27.2 ± 5.3^b^	3.8 ± 0.4^b^	1.2 ± 0.3^b^
9e	10.8 ± 2.2	2.5 ± 0.4	1.2 ± 0.2^b^
9f	22.4 ± 3.7^b^	3.9 ± 0.6^b^	2.2 ± 0,3^b^
9g	19.6 ± 3.6^b^	3.8 ± 0.5^b^	8.4 ± 0.4^b^
9h	34.6 ± 3.9^b^	4.2 ± 0.5^b^	4.9 ± 0.6^b^
9i	24.4 ± 3.3^b^	3.2 ± 0.8^b^	2.4 ± 0.6^b^
9j	9.4 ± 1.8^b^	3.2 ± 0.7^b^	1.6 ± 0.4^b^
9k	21.2 ± 2.0^b^	4.3 ± 0.5^b^	6.4 ± 2.1^b^
9l	15.8 ± 2.8	2.5 ± 0.4	1.2 ± 0.2^b^
9m	18.2 ± 3.1	2.9 ± 0.3	1.8 ± 0.4^b^
9n	20.6 ± 3.2^b^	3.9 ± 0.8^b^	1.9 ± 0.5^b^
9o	19.8 ± 1.5^b^	3.5 ± 0.3^b^	1.87 ± 0.4^b^
9p	18.8 ± 1.2^b^	3.5 ± 0.2^b^	1.29 ± 0.4^b^
Ethosuximide	16.8 ± 3.8	2.8 ± 1.2	0.5 ± 0.08
Diazepam	33.6 ± 4.2^b^	8.4 ± 1.0^b^	3.2 ± 0.9^b^

a *P* ≤ 0.05 at a probability level.

b The differences are statistically significant compared with the control.

Many of the compounds produced a statistically significant increase in horizontal and vertical movements, indicating a moderate activating effect. In addition, all compounds significantly increased the number of sniffing cell examinations compared with the control group, which may reflect anxiolytic activity (Table 3 and Fig. 3). This effect was particularly pronounced for compounds 9c, 9g, 9k, and 9h.

The rank order of activity based on horizontal displacement in the open field test was as follows: 9c > 9h > 9d > 9i > 9f > 9k > 9n > 9o > 9g > 9p > 9m > 9a > 9b > 9l > 9e > 9j.

Ethosuximide, administered at an effective dose of 200 mg kg^−1^, did not significantly affect any of the behavioral parameters assessed. In contrast, diazepam (2 mg kg^−1^) produced a marked increase in the number of examined cells compared with the control group, indicating a pronounced anxiolytic effect. Similar to diazepam, the studied compounds increased both horizontal and vertical locomotor activity, demonstrating an activating influence on behavior.

Structure–activity relationship analysis revealed that the presence of an azepane moiety at the 1-position of the 7-isopropyl-5,6,7,8-tetrahydro-2,7-naphthyridine-4-carbonitrile scaffold (compound 9c) is favorable for anxiolytic activity in this test. Substitution of the azepane ring with a morpholine moiety (compound 9h) slightly reduced the activity. Replacement of ethyl groups with methyl groups in the 2-((1*R*,5*S*)-9-oxo-3,7-diazabicyclo[3.3.1]nonan-3-yl)-2-oxoacetate fragment resulted in a less active compound (9d); however, it still remained among the three most active compounds.

On the other hand, replacement of the methyl groups in the 2-((1*R*,5*S*)-1,5-dimethyl-9-oxo-3,7-diazabicyclo[3.3.1]nonan-3-yl)-2-oxoacetate moiety of compound 9k with ethyl groups to form 2-((1*R*,5*S*)-1,5-diethyl-9-oxo-3,7-diazabicyclo[3.3.1]nonan-3-yl)-2-oxoacetate was detrimental to activity.

In general, the activity of the compounds in the open field test depends not only on the substituents at the 1- and 7-positions of the 2,7-naphthyridine ring but also on the nature of the substituents within the 3,7-diazabicyclo[3.3.1]nonane moiety.

To assess anxiety-related behaviour, the Elevated Plus Maze (EPM) methodology developed by Pellow *et al.* (1986) was employed.^87–89^ The EPM is a widely used behavioural assay for evaluating fear and the anxiolytic effects of pharmacological agents and synthetic compounds. Briefly, rats or mice were placed at the central junction of the four arms of the maze facing an open arm, and the number of entries into, as well as the time spent in each arm was recorded simultaneously by an observer and a video-tracking system over a 5-min period.

In this test, compound 9c again demonstrated the highest activity. This effect may be due to the presence of isopropyl and azepane substituents on the 2,7-naphthyridine moiety, along with two methyl groups on the 3,7-diazabicyclo[3.3.1]nonane fragment.

**Fig. 4:**
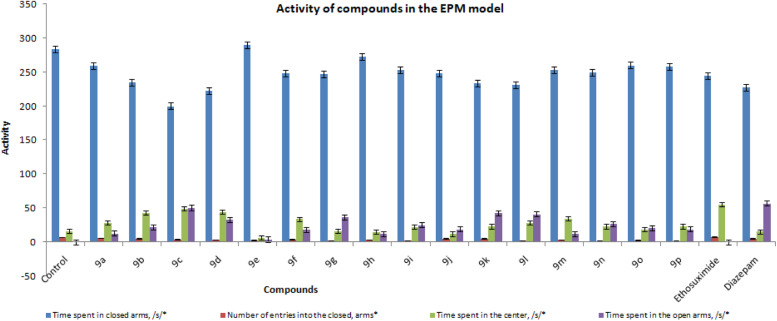
Graphical depiction of activity of compounds 9a–p, ethosuximide, and diazepam in the EPM model (5 min of research).

In the EPM model, control animals predominantly remained in the closed arms (Table 4 and Fig. 4). Administration of compounds 9a–p and reference drugs generally reduced the time spent in closed arms, with the exception of compound 9e. The decrease was statistically significant for three compounds: 9c, 9g, and 9k. All tested compounds also reduced the number of entries into the closed arms.

**Table 4:** Effect of compounds 9a–p on fear- and despair-related behavior in mice using the Elevated Plus Maze (EPM) model (5-minute observation period)

Compounds	Time spent in closed arms/s^a^	Number of entries into the closed, arms^a^	Time spent in the center/s^a^	Time spent in the open arms/s^a^
Control	284 (251 ÷ 321)	7.0 (5.8 ÷ 8.4)	16 (10 ÷ 24.8)	0
9a	259.3 (215.8 ÷ 310)	6 (3.8 ÷ 11)	28 (18 ÷ 45)^b^	12.7 (6.8 ÷ 25)^b^
9b	235 (196 ÷ 282)	5.6 (4 ÷ 7.8)	43 (27.7 ÷ 66.5)^b^	22 (18 ÷ 26)^b^
9c	200 (174 ÷ 230)^b^	4.2 (3.5 ÷ 5)^b^	49.4 (41.2 ÷ 59.3)^b^	50.6 (38.9 ÷ 65.8)^b^
9d	222.8 (193.7 ÷ 256)^b^	3.6 (3.0 ÷ 4)^b^	44 (33.8 ÷ 57.2)^b^	33.2 (25.5 ÷ 43.2)^b^
9e	290 (200 ÷ 420.5)	3.0 (2 ÷ 3.8)^b^	6.0 (5 ÷ 7.2)^b^	4 (3.1 ÷ 5.1)^b^
9f	248 (206 ÷ 297.6)	4.2 (3.5 ÷ 5)^b^	34 (27 ÷ 42.5)^b^	18 (12 ÷ 27)^b^
9g	247 (190 ÷ 321)	2 (1.59 ÷ 2.52)^b^	16 (13.3 ÷ 19.2)	37 (32 ÷ 42,5)^b^
9h	272.8 (218 ÷ 341)	3.4 (28 ÷ 4.1)^b^	15.2 (13.2 ÷ 17.5)^b^	12 (10 ÷ 14.4)^b^
9i	253 (210.8 ÷ 303.6)^b^	2.4 (1.85 ÷ 3.12)^b^	22 (17.6 ÷ 27.5)^b^	25 (20.8 ÷ 31.2)^b^
9j	248 (183.7 ÷ 334.8)	5.4 (4.5 ÷ 6.5)	12 (10 ÷ 14.4)	19 (15.8 ÷ 22.8) b
9k	234 (203.5 ÷ 269)^b^	5.0 (4.3 ÷ 5.7)^b^	23 (40 ÷ 57.6)^b^	43 (28 ÷ 37)^b^
9l	231 (204.4 ÷ 250)^b^	2.2 (1.8 ÷ 2.64)^b^	28 (25.5 ÷ 30.8)^b^	41 (31.5 ÷ 53.3)^b^
9m	253 (230 ÷ 278)^b^	3.4 (2.8 ÷ 4.1)^b^	35 (26.9 ÷ 45.5)^b^	12 (10 ÷ 14.4)^b^
9n	250 (192 ÷ 325)^b^	1.6 (1.23 ÷ 2.08)^b^	23 (17.6 ÷ 29.9)^b^	27 (22.5 ÷ 32.4)^b^
9o	260 (216.7 ÷ 312)^b^	2.5 (2.1 ÷ 3)^b^	19 (14.6 ÷ 24.7)^b^	21 (17.5 ÷ 25.2)^b^
9p	258 (191 ÷ 348)^b^	1.8 (1.5 ÷ 2.16)^b^	23 (20 ÷ 26.45)^b^	19 (15.2 ÷ 23.75)^b^
Ethosuximide	245 (213 ÷ 277.5)	8 (5.6 ÷ 10.6)	55 (46 ÷ 66)^b^	0
Diazepam	227.5 (190 ÷ 273)	5.5 (4.6 ÷ 6.6)	15.5 (13 ÷ 18.6)	57 (47.5 ÷ 68)^b^

a *P* ≤ 0.05 at a probability level.

b The differences are statistically significant compared with the control.

Many of the compounds significantly increased the time animals spent in the central area compared with controls. Following administration, experimental animals entered the open arms and remained there for 4 s (9e) to 50.6 s (9c), in contrast to control mice. The most active compound, 9c, reduced the time spent in closed arms by approximately 200 s and simultaneously increased time spent in the center by 49.4 s.

Control mice, as well as those treated with ethosuximide at 200 mg kg^−1^, did not enter the open arms, likely due to fear. In contrast, animals treated with diazepam (2 mg kg^−1^) entered the open arms and remained there for 57 s. The obtained data indicate that all tested compounds exhibit anxiolytic activity, with the strongest effects observed for compounds 9c, 9k, 9l, and 9g. Particularly, three of these four compounds (9c, 9g, and 9k) contain an azepane moiety in the 2,7-naphthyridine ring. The relative activity of the compounds in this assay can be ranked as follows: 9c > 9k > 9l > 9g > 9d > 9n > 9i > 9b > 9o > 9j = 9p > 9g > 9a > 9h = 9m > 9e. Consistent with observations from the other assays, compound 9c demonstrated the highest anxiolytic activity, whereas compound 9e was the least active.

The obtained data indicate the anxiolytic activity of all selected compounds, especially expressed by the compounds 9c, 9k, 9l and 9g. It should be mentioned, that all these four compounds 9c, 9g, 9k have azepane fragment in the 2,7-naphthridine ring. The order of activity of compounds in this assay can be presented as: 9c > 9k > 9l > 9g > 9d > 9n > 9i > 9b > 9o > 9j = 9p > 9g > 9a > 9h = 9m > 9e. Thus, again compound 9c is the most active like it was observed in other assays mentioned above. While, compound 9c showed the best activity in this test, compound 9e was the least active one.

**Fig. 5:**
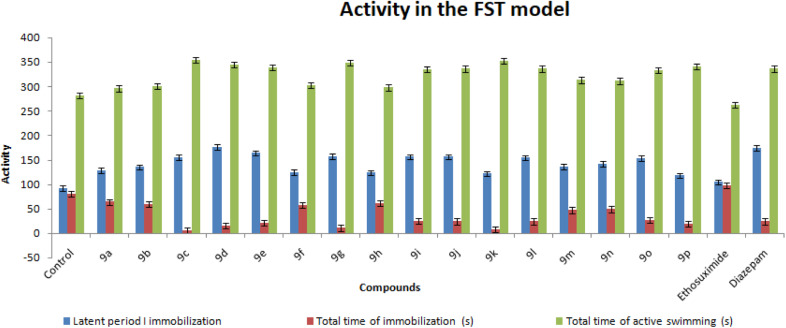
Graphical representation of the behavioral effects of compounds 9a–p, ethosuximide, and diazepam in the FST (6-minute observation).

The Forced Swimming Test (FST), also known as Porsolt's test, is one of the most widely used assays for evaluating depressive-like behavior.^90,91^ In this test, immobility is considered an indicator of behavioural despair. Similarly, the Tail Suspension Test (TST)^92–94^ is also generally employed to assess antidepressant-like activity, serving as a complementary method to the FST. In the Forced Swimming Test (FST) (Table 5 and Fig. 5), control mice showed the first immobilization at 92 s. Administration of the compounds at 50 mg kg^−1^ significantly increased the latency to first immobilization and total active swimming, while reducing immobility. For example, the latency to first immobilization increased to 155 s (9c), 176 s (9d), and 157.5 s (9g), and total active swimming for 9c, 9g, 9k, and 9d was 354, 349, 352, and 345 s, respectively.

**Table 5:** Effect of compounds 9a–p according to the forced swimming test (observation time 6 min)^a^

Compounds	Latent period I immobilization	Total time of immobilization (s)	Total time of active swimming (s)
Control	92 (80.0 ÷ 105.8)	81 (67.5 ÷ 97)	282 (254 ÷ 313)
9a	128 (116.4 ÷ 140.8)^b^	64 (53.3 ÷76.8)^b^	296 (255 ÷ 367)
9b	135 (112.5 ÷ 162)^b^	60 (53 ÷ 67.5)^b^	300 (231 ÷ 390)
9c	155 (129.2 ÷ 186)^b^	6 (5 ÷ 7.2)^b^	354 (316 ÷ 403)^b^
9d	176 (146.7 ÷ 211)^b^	15 (11.5 ÷ 19.5)^b^	345 (313.6 ÷ 379.5)^b^
9e	164 (128 ÷ 200)^b^	21 (11.6 ÷ 15)^b^	339 (313 ÷ 385)^b^
9f	125 (113.6 ÷ 137.5)^b^	58 (48.3 ÷ 69.6)^b^	302 (274.5 ÷ 332)^b^
9g	157.5 (131.2 ÷ 189)^b^	11 (9.56 ÷ 12.65)^b^	349 (317.3 ÷ 383.9)^b^
9h	124 (99.2 ÷ 155)^b^	62 (55.7 ÷ 73.6)	298 (248 ÷ 357.6)
9i	156 (130 ÷ 187.2)^b^	25 (19.2 ÷ 32.5)^b^	335 (304.5 ÷ 368.5)^b^
9j	156 (120 ÷ 202.8)	24 (20 ÷ 28.8)	336 (280 ÷ 403.2)^b^
9k	122 (101.6 ÷ 146.4)^b^	8 (6.7 ÷ 9.6)^b^	352 (293 ÷ 422.4)^b^
9l	154 (123.2 ÷ 192.5)^b^	24 (20 ÷ 28.8)	336 (305.5 ÷ 369.6)^b^
9m	136 (129.2 ÷ 186)^b^	47 (39.2 ÷ 56.4)^b^	313 (277 ÷ 353.7)^b^
9n	142 (117.3 ÷ 171.8)^b^	49 (40.5 ÷ 59.3)^b^	311 (257 ÷ 376)^b^
9o	153.3 (126.7 ÷ 185.5)^b^	27 (20.8 ÷ 35)^b^	333 (302.7 ÷ 366)^b^
9p	118 (98.3 ÷ 141.6)^b^	19 (15.2 ÷ 23.8)^b^	341 (294 ÷ 396)^b^
Ethosuximide	105 (87.5 ÷ 104.4)	98 (75.3 ÷ 127.4)	262 (200 ÷ 324)
Diazepam	174 (144 ÷ 204)^b^	24 (13.9 ÷ 34)^b^	336 (303 ÷ 373)

a *P* ≤ 0.05 at a probability level.

b The differences are statistically significant compared with the control.

These findings indicate that most compounds at 50 mg kg^−1^ exhibit antidepressant-like effects similar to diazepam, whereas ethosuximide (200 mg kg^−1^) showed no effect compared with controls. Results from the TST were consistent, confirming the antidepressant activity of the same compounds (9c, 9d, 9g, and 9k). The rank order of activity in the FST was: 9c > 9k > 9g > 9d > 9p > 9j = 9l > 9i > 9e > 9o > 9m > 9n > 9f > 9b > 9h > 9a.

**Fig. 6:**
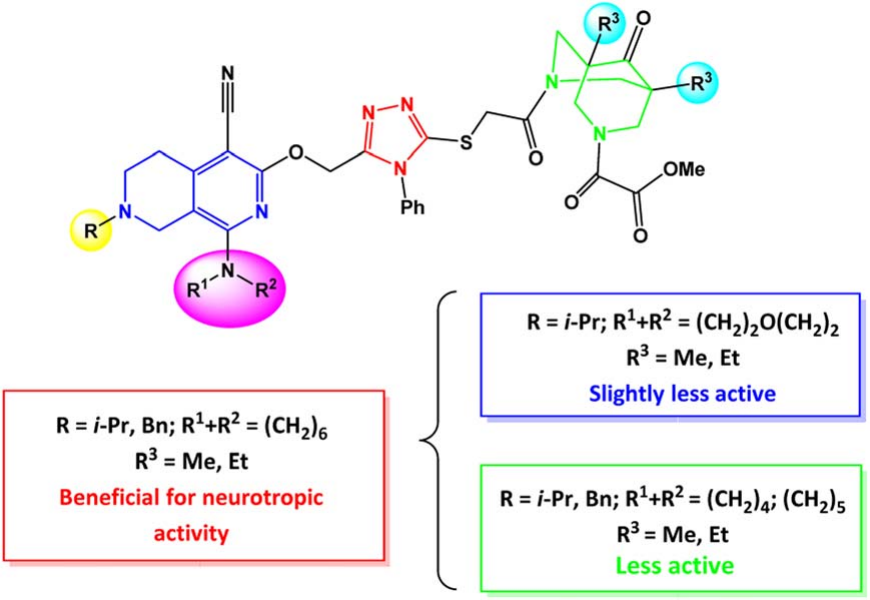
Structure–activity relationship on neurotropic activity for 1,2,4-triazole-linked hybrids 9a–p.

In summary, SAR analysis of neurotropic activity among the 16 studied compounds showed that the most active were 9c, 9h, 9k, 9d, and 9g, with compound 9c exhibiting the highest activity. These results indicate that the presence of an azepane ring at the 1-position and an isopropyl group at the 7-position of the 2,7-naphthyridine ring is favourable for neurotropic activity. Compounds containing a morpholine fragment were less active, and those with ethyl groups in the 3,7-diazabicyclo[3.3.1]nonane ring were generally less active than their methyl-substituted counterparts (Fig. 6). In particular, replacing the isopropyl group at the 7-position of the 2,7-naphthyridine ring with a benzyl group generally resulted in decreased activity, with the exception of compound 9k, which remained among the active compounds. The main structural difference between 9k and one of the most active compounds, 9c, is the presence of a benzyl group at the 7-position instead of an isopropyl group. The good activity of 9k compared with other benzyl-substituted compounds (9j–p) may be due to the combination of the benzyl group at the 7-position with an azepane ring at the 1-position of the 2,7-naphthyridine ring. Nevertheless, the compounds exhibited different behaviors in the various tests, which depended mainly on the substituents at the 1- and 7-positions of the 2,7-naphthyridine ring.

Nevertheless, the compounds showed different behavior in different test and is dependent mainly on substituents at position 1 and 7 of 2,7-naphthiridine cycle.

### 2.3. Molecular docking

#### 2.3.1. Docking to GABAA receptor – prediction of the mechanism of anticonvulsant and anxiolytic activity

**Fig. 7:**
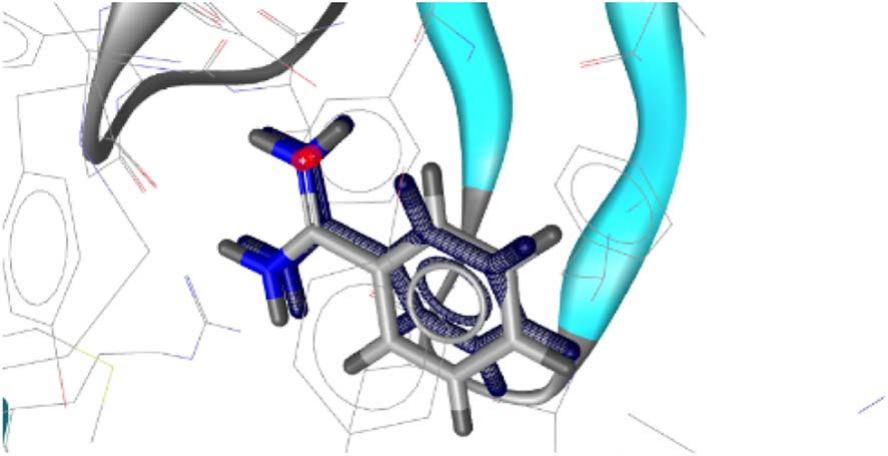
Alignment of the docked conformation of the re-docked initial inhibitor benzamidine (blue) and the co-crystalized ligand to the structure 4COF (RMSD: 0.34 Å).

Antiepileptic agents often exert their therapeutic effects by modulating the γ-aminobutyric acid type A (GABAA) receptor, either through inhibition of voltage-gated sodium channels or by enhancing GABAergic neurotransmission.^95,96^ To gain deeper insight into the molecular determinants of GABAA receptor modulation and to elucidate the key interactions within its active site, molecular docking studies were carried out for all investigated compounds. For this purpose, the crystal structure of the GABAA receptor was retrieved from the Protein Data Bank (PDB ID: 4COF).^97^ The selection of PDB structures was based on the best available crystallographic templates at the time of the study, which have been widely employed in previous docking investigations targeting the same proteins. The X-ray diffraction data indicated a resolution of 2.97 Å, with *R* and *R* free values of 0.206 and 0.226, respectively, confirming the quality of the structure for docking experiments. The co-crystallized ligand, benzamidine, was initially removed from the binding pocket and subsequently re-docked into the receptor's catalytic site under identical preparation and docking conditions as those applied to the tested compounds. This re-docking step served as a validation procedure for the docking protocol. The superimposition of the re-docked pose with the crystallographic orientation of benzamidine yielded a root-mean-square deviation (RMSD) of 0.34 Å (Fig. 7), indicating a high level of accuracy and reliability of the docking method (Table 6).

**Table 6:** Molecular docking into GABA_A_ receptor

No.	Est. binding energy (kcal mol^−1^)	Residues involved in hydrogen bond formation	Hydrophobic interactions	Aromatic interactions
9a	−6.92	Tyr205 (N⋯H, 2.72 Å)	Ala25, Phe200	—
9b	−6.82	—	Thr176, Tyr157	—
9c	−9.89	Thr202 (O⋯H, 2.15 Å)	Leu99, Val198, Thr176, Phe200, Ala201	—
9d	−7.56	Thr202 (O⋯H, 3.76 Å)	Thr176, Ala201, Phe200	—
9e	−8.45	Thr202 (O⋯H, 3.54 Å)	Thr176, Ala201, Phe200	—
9f	−7.04	Tyr97 (O⋯H, 2.79 Å)	Tyr62, Phe200	—
9g	−10.52	Ala201 (N⋯H, 3.72 Å), Thr202 (O⋯H, 3.56 Å)	Thr176, Ala201, Tyr205	—
9h	−9.52	Tyr97 (N⋯H, 3.79 Å), Thr202 (O⋯H, 2.72 Å)	Leu99, Thr176, Phe200, Ala201	—
9i	−6.86	—	Ile42, Tyr62, Tyr157, Tyr205	—
9j	−8.82	Tyr97 (N⋯H, 3.65 Å)	Phe200, Ala201, Tyr205	—
9k	−10.85	Tyr62 (O⋯H, 3.70 Å), Arg180 (O⋯H, 3.56 Å)	Leu99, Val198, Phe200, Ala201, Thr202	Asp43 (ionic)
9l	−9.59	Thr202 (N⋯H, 2.97 Å), Tyr205 (N⋯H, 2.86 Å)	Ile42, Leu99, Phe200	Phe200
9m	−8.10	Thr202 (O⋯H, 3.56 Å)	Thr176, Phe200	—
9n	−7.54	Tyr97 (N⋯H, 3.55 Å)	Thr176, Phe200, Ala201	—
9o	−8.17	Thr202 (O⋯H, 3.72 Å)	Thr176, Phe200	—
9p	−8.95	Tyr97 (N⋯H, 3.53 Å), Thr202 (O⋯H, 2.11 Å)	Ala201, Phe200, Tyr205	—
Diazepam	−8.90	Thr202 (N⋯H, 2.67 Å)	Tyr62, Thr176, Phe200, Ala201, Tyr205	Phe200

The docking results for compound 9k (Table 7) are in agreement with the *in vivo* pharmacological findings, reinforcing its role as a promising lead candidate. Compound 9k exhibited a superior binding energy to the GABAA receptor (−10.85 kcal mol^−1^) compared with diazepam, indicating a stronger binding affinity.

**Table 7:** Molecular docking in SERT transporter (PDB ID: 5I71)

No.	Est. binding energy (kcal mol^−1^)	Residues involved in hydrogen bond formation	Residues involved in hydrophobic interactions	Residues involved in positive ionizable interactions
9a	−7.11	Tyr95 (N⋯H, 3.54 Å)	Tyr176, Phe335	—
9b	−6.15	—	Tyr175	—
9c	−8.27	Tyr95 (O⋯H, 3.82 Å), Tyr175 (N⋯H, 3.03)	Ile172, Tyr175, Phe335	Asp98
9d	−4.19	—	Val501	—
9e	−6.28	—	Tyr175	—
9f	−5.19	—	Tyr175, Tyr176	—
9g	−8.32	Tyr95 (N⋯H, 3.10 Å)	Leu99, Tyr176, Phe335	Asp98
9h	−7.26	Tyr95 (N⋯H, 2.73 Å)	Phe335, Val501	Asp98
9i	−5.48	—	Tyr175	—
9j	−7.26	Tyr95 (N⋯H, 2.85 Å)	Tyr176	—
9k	−8.18	Tyr95 (O⋯H, 3.15 Å), Tyr175(N⋯H, 3.11 Å), Thr497 (N⋯H, 2.74 Å)	Ile172, Tyr175, Tyr176	Asp98
9l	−7.59	Thr497 (O⋯H, 3.50 Å)	Ile172, Ph335	Asp98
9m	−4.90	—	Tyr176, Phe335	—
9n	−7.62	Tyr95 (N⋯H, 2.74 Å)	Ala173, Ile266, Leu443	—
9o	−4.19	—	—	—
9p	−7.05	—	Ile172, Ph335	Asp98

**Fig. 8:**
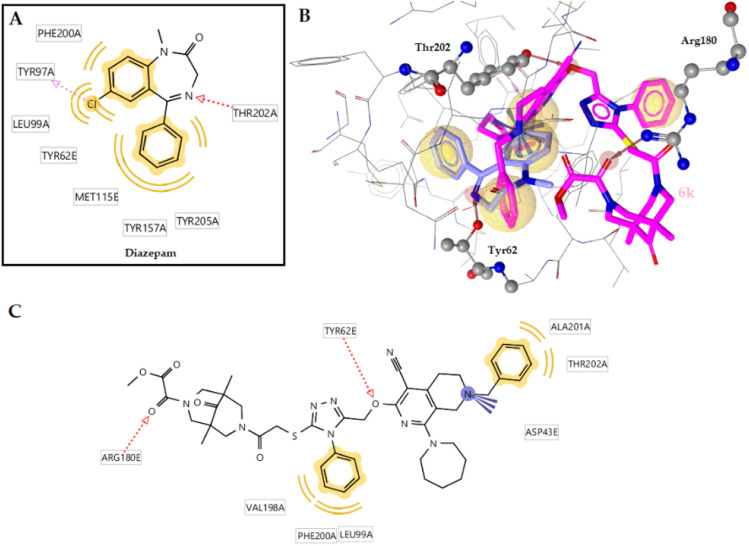
(A) 2D pose of diazepam; (B) superposition of compound 9k with diazepam; (C) 2D pose of compounds 9k.

The predicted binding pose revealed that 9k occupies the classical benzodiazepine binding site at the α1–γ2 subunit interface, forming two key hydrogen bonds with Tyr62 and Arg180 that stabilize the ligand within the pocket. In addition, 9k establishes multiple hydrophobic contacts with Leu99, Val198, Phe200, Ala201, and Thr202, contributing to a well-packed and energetically favorable complex. In contrast, diazepam forms only a single hydrogen bond with Thr202, resulting in a less extensive interaction network. The presence of both hydrogen-bonding and hydrophobic anchoring points in 9k suggests enhanced receptor stabilization and stronger modulation of the GABAergic site. This binding profile is consistent with its higher anticonvulsant potency observed *in vivo*, as evidenced by its lower ED_50_ in the PTZ model and protective effect against TSC-induced seizures (Fig. 8).

#### 2.3.2. Docking to SERT transporter and β_2_-adrenergic receptor

Tricyclic antidepressants and selective serotonin reuptake inhibitors (SSRIs) represent the two main classes of antidepressants.^98^ These drugs inhibit the serotonin transporter (SERT), preventing reuptake of serotonin into presynaptic neurons. SERT, a transmembrane protein located in the presynaptic membrane, normally clears serotonin from the synaptic cleft. Increased serotonin levels subsequently activate 5-HT1A receptors, which reduce serotonergic neurotransmission and cause a delay in antidepressant onset until these receptors become desensitized and serotonin release is restored.^99,100^

Considering these mechanisms, we extended our docking studies to both the SERT transporter and the β_2_-adrenergic receptor to evaluate whether the synthesized compounds could act as dual inhibitors—blocking SERT, while also antagonizing the presynaptic au-to-inhibitory β_2_-adrenergic receptors. The X-ray structure of the ts3 human serotonin transporter complexed with *s*-citalopram at the central site (PDB ID: 5I71) was used.^101^

**Fig. 9:**
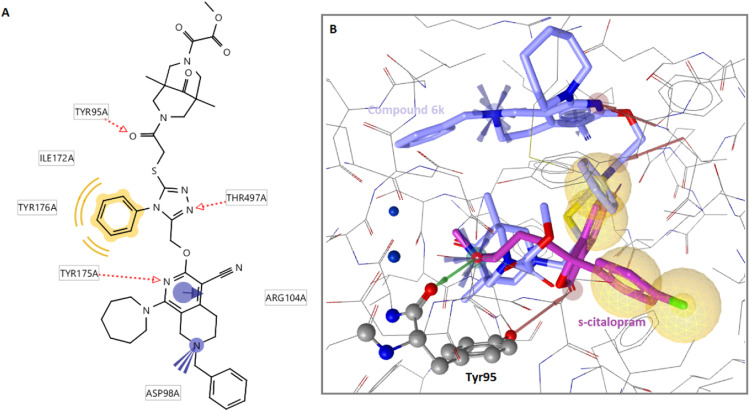
(A) 2D ligand interaction diagram for docked compound 9k. (B) Docked pose of compound 9k (purple) and *s*-citalopram (magenta) into SERT transporter.

Compound 9k demonstrated the most favorable docking score, supported by the formation of three key hydrogen bonds (Table 7). The first bond was observed between the oxygen atom of the carbonyl group and the hydrogen of the Tyr95 side chain at a distance of 3.15 Å, while the second involved the nitrogen atom and the hydrogen of the Tyr175 side chain. The last one hydrogen bond is formed between the nitrogen atom and the residue Thr497 (Fig. 9). These binding interactions are consistent with the experimental pharmacological data, indicating strong alignment between the computational predictions and *in vivo* results.

**Fig. 10:**
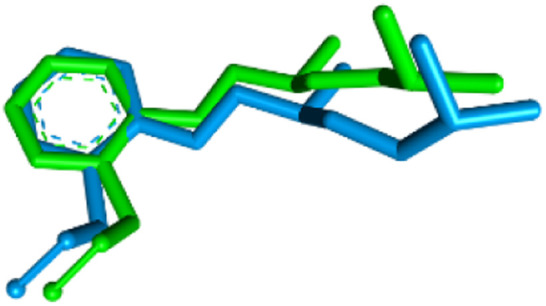
Alignment of the docked conformation of the re-docked initial inhibitor alprenolol (blue) and the co-crystalized ligand (green) to the structure 3NYA (RMSD: 0.98 Å).

For the docking studies on the β_2_ adrenergic receptor, the crystal structure of the human β_2_ adrenergic receptor in complex with the antagonist alprenolol (PDB ID: 3NYA) was employed as a structural template.^102,103^ To validate the docking protocol, the co-crystallized ligand alprenolol was extracted and re-docked into the receptor's active site under the same conditions as the test compounds. The resulting root-mean-square deviation (RMSD) between the experimental and re-docked conformations was 0.98 Å, confirming the reliability of the docking procedure (Fig. 10).

**Fig. 11:**
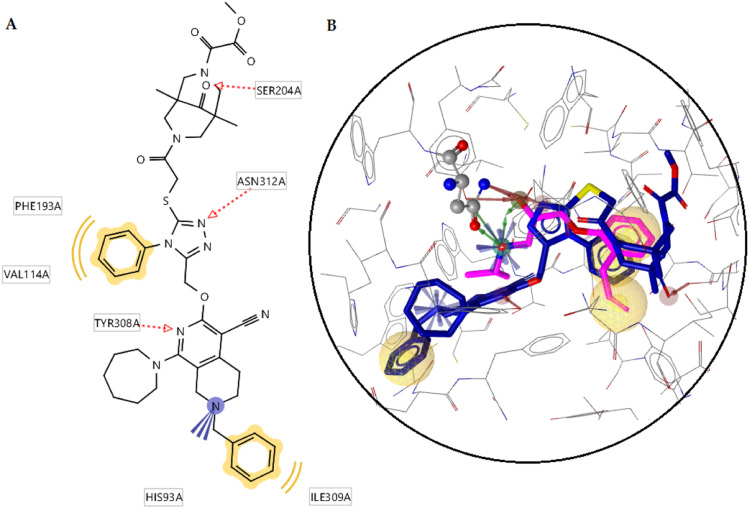
(A) 2D ligand interaction diagram for docked compound 9k. (B) Docked pose of compound 9k (blue) and alprenolol (magenta) into β_2_ adrenergic receptor.

Docking of the synthesized compounds into the β_2_-adrenergic receptor (Table 8) showed that compound 9k had the most favorable predicted binding energy (−12.10 kcal mol^−1^), forming hydrogen bonds with Asn312, Thr308, and Ser204, along with hydrophobic contacts with Ile309, Phe193, and Val114 (Fig. 11). Similar to alprenolol, 9k engaged Asn312 through hydrogen bonding and occupied the same binding cavity (Fig. 11B).

**Table 8:** Molecular docking free binding energies (kcal mol^−1^) in β_2_ adrenergic receptor (PDB ID: 3NYA)

No.	Est. binding energy (kcal mol^−1^)	Residues involved in hydrogen bond formation	Residues involved in hydrophobic interactions
9a	−5.49	—	Ala200
9b	−6.10	—	Trp109, Val114, Ile309, Tyr308
9c	−9.92	Thr195 (O⋯H, 3.11 Å), Asn312 (N⋯H, 3.50 Å)	Trp109, Thr118, Ala200, Asn312
9d	−8.67	Tyr316 (O⋯H, 3.18 Å)	Val114, Thr118, Ala200, Asn312
9e	−7.37	—	Thr118, Ala200, Asn312
9f	−6.86	—	Thr118, Trp286
9g	−9.06	Tyr316 (O⋯H, 3.02 Å)	Thr118, Ala200, Asn312
9h	−7.03	—	Trp109, Phe193, Ala200, Phe289, Tyr308
9i	−5.10	—	Thr118, Trp286
9j	−8.25	Tyr308 (O⋯H, 2.54 Å)	Trp286, Asn312
9k	−12.10	Ser204 (O⋯H, 3.12 Å), Asn312 (N⋯H, 3.52 Å), Tyr308 (N⋯H, 3.76 Å)	Val114, Phe193, Ile309
9l	−8.70	Asn312 (O⋯H, 2.74 Å)	Trp109, Val114, Ala200, Phe289, Tyr316
9m	−8.02	Asn312 (N⋯H, 3.47 Å)	Trp109, Arg431
9n	−8.64	Tyr316 (O⋯H, 3.75 Å)	Trp109, Ile309, Ile201, Ala200
9o	−5.96	—	Val114, Thr118, Ala200
9p	−7.82	Tyr308 (O⋯H, 2.58 Å)	Ala200, Asn312
Alprenolol	−13.19	Asp113, Asn312, Tyr316	Val114, Tyr118, Ala200, Tyr308

These computational findings suggest a potentially stronger interaction of 9k with the receptor; however, they do not confirm functional activity. Likewise, the observed affinity in both SERT and β_2_AR docking studies indicates that 9k may possess structural features compatible with multi-target engagement, though experimental validation would be required to substantiate any dual-acting pharmacological profile.

### 2.4. Limitations and correlation with biological data

Although the docking studies provided valuable structural insights into the interactions between the synthesized compounds and their molecular targets, certain limitations should be acknowledged. The docking simulations were performed on static receptor structures and therefore do not fully account for protein flexibility, solvent effects, or dynamic conformational changes that may occur *in vivo*. Moreover, the use of the bacterial homolog LeuT as a model for human SERT and the β_2_-adrenergic receptor as a template for the 5-HT_1_A receptor introduces potential structural discrepancies that could influence the predicted binding affinities. Nevertheless, the observed docking trends are largely consistent with the biological results, as compound 9k exhibited both the most favorable binding energies and the highest anticonvulsant and antidepressant-like activity in the pharmacological assays. This concordance supports the reliability of the computational findings, while the identified limitations highlight the need for further validation through molecular dynamics simulations or experimental binding studies.

## 3 Materials and methods

### 3.1. Chemistry

General information: all chemicals and solvents were of commercially high purity grade and purchased from Sigma Aldrich (Saint Louis, MO, USA). ^1^H, ^13^C NMR spectra for compounds 2b, 2d–h; 3a–h; 4a–h; 5a–h; 8a,b and 9a–n, 9p were recorded in DMSO-*d*_6_/CCl_4_ (1/3) and DMSO-*d*_6_ solutions (300 MHz for ^1^H and 75 MHz for ^13^C, respectively) on a Mercury 300VX spectrometer (Varian Inc., Palo Alto, CA, USA). ^1^H, ^13^C NMR spectra for compounds 7a,b and 9o were recorded on a BRUKER AVANCE NEO spectrometer (Bruker BioSpin GmbH, Rheinstetten, Germany) with operating frequencies of 400 and 100 MHz, respectively, (in CDCl_3_) at 303 K. Chemical shifts were reported as *δ* (parts per million) relative to TMS as internal standard. The IR spectra were recorded on a Nicolet Avatar 330-FT-IR spectrophotometer (Thermo Nicolet, Foster, CA, USA) in vaseline, νmax in cm^−1^. MS spectra were recorded on Waters XEVO G3 Q-Tof spectrometer (Waters Corporation Company, Milford, MA, USA). Elemental analyses were performed on a Elemental Analyzer Euro EA 3000 (EuroVector, Pavia, Italy). Melting points were determined on a MP450 melting point apparatus. The physicochemical data for compounds 2a,c^68^ and 6 (ref. 70) were already reported.

#### 3.1.1. General procedure for the synthesis of compounds 2b, 2d–h

To a suspension of compound 1 (100 mmol) and potassium carbonate (27.6 g, 200 mmol) in absolute DMF (150 mL) ethyl chloroacetate (12.8 mL, 120 mmol) was added dropwise under stirring. The reaction mixture was maintained at 80–85 °C for 5 h, then cooled to room temperature and poured onto ice water. The resulting crystals were filtered off, washed with water, dried, and recrystallized from ethanol.

##### 3.1.1.1 Ethyl[(4-cyano-7-isopropyl-1-piperidin-1-yl-5,6,7,8-tetrahydro-2,7-naphthyridin-3-yl)oxy]acetate (2b)

Colorless solid, yield 79%, m.p. 118–120 °C. IR *ν*/cm^−1^: 1747 (C=O), 2208 (C≡N). ^1^H NMR (300 MHz, DMSO-*d*_6_/CCl_4_, 1/3) *δ* 1.09 (d, *J* = 6.5 Hz, 6H, CH(CH̲_3_)_2_), 1.26 (t, *J* = 7.2 Hz, 3H, CH_2_CH̲_3_), 1.60–1.69 (m, 6H, C_5_H_10_N), 2.74 (t, *J* = 5.8 Hz, 2H, 6-CH_2_), 2.77–2.94 (m, 3H, 5-CH_2_, CH̲(CH_3_)_2_), 3.16–3.24 (m, 4H, N(CH_2_)_2_), 3.34 (s, 2H, 8-CH_2_), 4.17 (q, *J* = 7.1 Hz, 2H, CH̲_2_CH_3_), 4.82 (s, 2H, OCH_2_). ^13^C NMR (75 MHz, DMSO-*d*_6_/CCl_4_, 1/3) *δ* 13.7, 18.1, 23.9, 25.3, 28.8, 44.5, 48.4, 49.4, 53.2, 59.9, 62.0, 85.3, 114.1, 114.5, 151.0, 159.0, 159.9, 167.3. Anal. calcd for C_21_H_30_N_4_O_3_: C 65.26; H 7.82; N 14.50%. Found: C 64.92; H 7.99; N 14.25%.

##### 3.1.1.2 Ethyl[(4-cyano-7-isopropyl-1-morpholin-4-yl-5,6,7,8-tetrahydro-2,7-naphthyridin-3-yl)oxy]acetate (2d)

Colorless solid, yield 82%, m.p. 120–121 °C. IR *ν*/cm^−1^: 1758 (C=O), 2211 (C≡N). ^1^H NMR (300 MHz, DMSO-*d*_6_/CCl_4_, 1/3) *δ* 1.09 (d, *J* = 6.5 Hz, 6H, CH(CH̲_3_)_2_), 1.27 (t, *J* = 7.1 Hz, 3H, CH_2_CH̲_3_), 2.74 (t, *J* = 5.8 Hz, 2H, 6-CH_2_), 2.80–2.94 (m, 3H, 5-CH_2_, CH̲(CH_3_)_2_), 3.19–3.25 (m, 4H, N(CH_2_)_2_), 3.36 (s, 2H, 8-CH_2_), 3.66–3.72 (m, 4H, O(CH_2_)_2_), 4.18 (q, *J* = 7.1 Hz, 2H, CH̲_2_CH_3_), 4.83 (s, 2H, OCH_2_). ^13^C NMR (75 MHz, DMSO-*d*_6_/CCl_4_, 1/3) *δ* 13.8, 18.0, 28.8, 44.2, 48.3, 48.8, 53.2, 60.0, 62.1, 62.2, 65.7, 86.3, 113.8, 114.8, 151.5, 158.3, 159.8, 167.3. Anal. calcd for C_20_H_28_N_4_O_4_: C 61.84; H 7.27; N 14.42%. Found: C 62.21; H 7.45; N 14.66%.

##### 3.1.1.3 Ethyl[(7-benzyl-4-cyano-1-pyrrolidin-1-yl-5,6,7,8-tetrahydro-2,7-naphthyridin-3-yl)oxy]acetate (2e)

Yellow solid, yield 80%, m.p. 129–131 °C. IR *ν*/cm^−1^: 1763 (C=O), 2209 (C≡N). ^1^H NMR (300 MHz, DMSO-*d*_6_/CCl_4_, 1/3) *δ* 1.26 (t, *J* = 7.1 Hz, 3H, CH_2_CH̲_3_), 1.82–1.95 (m, 4H, C_4_H_8_N), 2.67 (t, *J* = 5.9 Hz, 2H, 6-CH_2_), 2.85 (t, *J* = 5.8 Hz, 2H, 5-CH_2_), 3.44–3.50 (m, 4H, N(CH_2_)_2_), 3.54 (s, 2H, 8-CH_2_), 3.65 (s, 2H, CH̲_2_Ph), 4.16 (q, *J* = 7.1 Hz, 2H, CH̲_2_CH_3_), 4.78 (s, 2H, OCH_2_), 7.17–7.32 (m, 5H, Ph). ^13^C NMR (75 MHz, DMSO-*d*_6_/CCl_4_, 1/3) *δ* 13.7, 24.8, 28.4, 47.9, 49.1, 53.0, 59.8, 61.6, 61.9, 81.3, 109.3, 114.7, 126.6, 127.7, 128.1, 137.45, 149.4, 156.0, 159.9, 167.4. Anal. calcd for C_24_H_28_N_4_O_3_: C 68.55; H 6.71; N 13.32%. Found: C 68.19; H 6.86; N 13.11%.

##### 3.1.1.4 Ethyl[(7-benzyl-4-cyano-1-piperidin-1-yl-5,6,7,8-tetrahydro-2,7-naphthyridin-3-yl)oxy]acetate (2f)

Yellow solid, yield 78%, m.p. 133–135 °C. IR *ν*/cm^−1^: 1754 (C=O), 2212 (C≡N). ^1^H NMR (300 MHz, DMSO-*d*_6_/CCl_4_, 1/3) *δ* 1.27 (t, *J* = 7.1 Hz, 3H, CH_2_CH̲_3_), 1.51–1.67 (m, 6H, C_5_H_10_N), 2.72 (t, *J* = 6.1 Hz, 2H, 6-CH_2_), 2.91 (t, *J* = 5.9 Hz, 2H, 5-CH_2_), 3.11–3.20 (m, 4H, N(CH_2_)_2_), 3.31 (s, 2H, 8-CH_2_), 3.66 (s, 2H, CH̲_2_Ph), 4.17 (q, *J* = 7.1 Hz, 2H, CH̲_2_CH_3_), 4.82 (s, 2H, OCH_2_), 7.18–7.33 (m, 5H, Ph). ^13^C NMR (75 MHz, DMSO-*d*_6_/CCl_4_, 1/3) *δ* 13.7, 23.8, 25.2, 28.2, 48.4, 49.2, 52.7, 59.9, 61.6, 62.0, 85.2, 113.5, 114.0, 126.6, 127.6, 128.8, 137.3, 150.6, 158.9, 160.0, 167.3. Anal. calcd for C_25_H_30_N_4_O_3_: C 69.10; H 6.96; N 12.89%. Found: C 69.48; H 7.13; N 13.15%.

##### 3.1.1.5 Ethyl[(1-azepan-1-yl-7-benzyl-4-cyano-5,6,7,8-tetrahydro-2,7-naphthyridin-3-yl)oxy]acetate (2g)

Yellow solid, yield 81%, m.p. 147–149 °C. IR *ν*/cm^−1^: 1761 (C=O), 2204 (C≡N). ^1^H NMR (300 MHz, DMSO-*d*_6_/CCl_4_, 1/3) *δ* 1.26 (t, *J* = 7.1 Hz, 3H, CH_2_CH̲_3_), 1.45–1.55 (m, 4H, C_6_H_12_N), 1.62–1.73 (m, 4H, C_6_H_12_N), 2.70 (t, *J* = 6.1 Hz, 2H, 6-CH_2_), 2.90 (t, *J* = 6.1 Hz, 2H, 5-CH_2_), 3.34 (s, 2H, 8-CH_2_), 3.43–3.50 (m, 4H, N(CH_2_)_2_), 3.65 (s, 2H, CH̲_2_Ph), 4.15 (q, *J* = 7.1 Hz, 2H, CH̲_2_CH_3_), 4.81 (s, 2H, OCH_2_), 7.18–7.31 (m, 5H, Ph). ^13^C NMR (75 MHz, DMSO-*d*_6_/CCl_4_, 1/3) *δ* 13.7, 26.1, 27.9, 28.6, 48.3, 50.6, 53.8, 59.9, 61.7, 61.8, 82.8, 110.4, 114.6, 126.58, 127.7, 128.2, 137.4, 150.3, 158.0, 159.5, 167.4. Anal. calcd for C_26_H_32_N_4_O_3_: C 69.62; H 7.19; N 12.49%. Found: C 69.28; H 7.37; N 12.72%.

##### 3.1.1.6 Ethyl[(7-benzyl-4-cyano-1-morpholin-4-yl-5,6,7,8-tetrahydro-2,7-naphthyridin-3-yl)oxy]acetate (2h)

Light yellow solid, yield 83%, m.p. 184–186 °C. IR *ν*/cm^−1^: 1755 (C=O), 2213 (C≡N). ^1^H NMR (300 MHz, DMSO-*d*_6_/CCl_4_, 1/3) *δ* 1.27 (t, *J* = 7.1 Hz, 3H, CH_2_CH̲_3_), 2.71 (t, *J* = 6.1 Hz, 2H, 6-CH_2_), 2.93 (t, *J* = 6.1 Hz, 2H, 5-CH_2_), 3.14–3.22 (m, 4H, N(CH_2_)_2_), 3.33 (s, 2H, 8-CH_2_), 3.58–3.66 (m, 4H, O(CH_2_)_2_), 3.66 (s, 2H, CH̲_2_Ph), 4.18 (q, *J* = 7.1 Hz, 2H, CH̲_2_CH_3_), 4.83 (s, 2H, OCH_2_), 7.19–7.33 (m, 5H, Ph). ^13^C NMR (75 MHz, DMSO-*d*_6_/CCl_4_, 1/3) *δ* 13.8, 28.8, 48.3, 48.7, 52.5, 60.0, 61.5, 62.2, 65.6, 86.2, 113.8, 113.9, 126.6, 127.7, 128.3, 137.3, 151.1, 158.2, 160.0, 167.2. Anal. calcd for C_24_H_28_N_4_O_4_: C 66.04; H 6.47; N 12.84%. Found: C 66.41; H 6.61; N 13.08%.

#### 3.1.2. General procedure for the synthesis of compounds 3a–h

A mixture of compound 2 (10 mmol) and hydrazine hydrate (100 mmol) in absolute ethanol (70 mL) was stirred for 8 h at room temperature. After, water (50 mL) was added, the precipitate was filtered off, washed with water, dried and recrystallized from ethanol.

##### 3.1.2.1 2-[(4-Cyano-7-isopropyl-1-pyrrolidin-1-yl-5,6,7,8-tetrahydro-2,7-naphthyridin-3-yl)oxy]acetohydrazide (3a)

Cream solid, yield 84%, m.p. 191–193 °C. IR *ν*/cm^−1^: 1694 (C=O), 2206 (C≡N), 3225, 3319 (NH, NH_2_). ^1^H NMR (300 MHz, DMSO-*d*_6_/CCl_4_, 1/3) *δ* 1.08 (d, *J* = 6.5 Hz, 6H, CH(CH̲_3_)_2_), 1.87–1.98 (m, 4H, C_4_H_8_N), 2.68 (t, *J* = 5.8 Hz, 2H, 6-CH_2_), 2.81 (t, *J* = 5.8 Hz, 2H, 5-CH_2_), 2.83 (sp, *J* = 6.5, 1H, CH̲(CH_3_)_2_), 3.53–3.63 (m, 4H, N(CH_2_)_2_), 3.56 (s, 2H, 8-CH_2_), 4.02 (br s, 2H, NH_2_), 4.67 (s, 2H, OCH_2_), 8.88 (s, 1H, NH). ^13^C NMR (75 MHz, DMSO-*d*_6_/CCl_4_, 1/3) *δ* 18.0, 24.9, 29.0, 44.0, 48.6, 49.3, 53.4, 63.2, 81.3, 109.8, 115.3, 149.5, 156.4, 160.0, 166.7. Anal. calcd for C_18_H_26_N_6_O_2_: C 60.32; H 7.31; N 23.45%. Found: C 59.97; H 7.16; N 23.20%.

##### 3.1.2.2 2-[(4-Cyano-7-isopropyl-1-piperidin-1-yl-5,6,7,8-tetrahydro-2,7-naphthyridin-3-yl)oxy]acetohydrazide (3b)

Colorless solid, yield 90%, m.p. 186–188 °C. IR *ν*/cm^−1^: 1687 (C=O), 2206 (C≡N), 3296, 3324 (NH, NH_2_). ^1^H NMR (300 MHz, DMSO-*d*_6_/CCl_4_, 1/3) *δ* 1.08 (d, *J* = 6.5 Hz, 6H, CH(CH̲_3_)_2_), 1.62–1.70 (m, 6H, C_5_H_10_N), 2.74 (t, *J* = 6.0 Hz, 2H, 6-CH_2_), 2.79–2.95 (m, 3H, 5-CH_2_, CH̲(CH_3_)_2_), 3.18–3.27 (m, 4H, N(CH_2_)_2_), 3.33 (s, 2H, 8-CH_2_), 4.01 (br s, 2H, NH_2_), 4.71 (s, 2H, OCH_2_), 8.95 (s, 1H, NH). ^13^C NMR (75 MHz, DMSO-*d*_6_/CCl_4_, 1/3) *δ* 18.1, 24.0, 25.4, 28.8, 44.6, 48.5, 49.4, 53.2, 63.3, 85.2, 114.0, 114.6, 150.7, 159.2, 160.1, 166.5. Anal. calcd for C_19_H_28_N_6_O_2_: C 61.27; H 7.58; N 22.56%. Found: C 60.94; H 7.74; N 22.34%.

##### 3.1.2.3 2-[(1-Azepan-1-yl-4-cyano-7-isopropyl-5,6,7,8-tetrahydro-2,7-naphthyridin-3-yl)oxy]acetohydrazide (3c)

Cream solid, yield 81%, m.p. 161–163 °C. IR *ν*/cm^−1^: 1693 (C=O), 2206 (C≡N), 3298, 3331 (NH, NH_2_). ^1^H NMR (300 MHz, DMSO-*d*_6_/CCl_4_, 1/3) *δ* 1.08 (d, *J* = 6.5 Hz, 6H, CH(CH̲_3_)_2_), 1.52–1.64 (m, 4H, C_6_H_12_N), 1.72–1.85 (m, 4H, C_6_H_12_N), 2.71 (t, *J* = 5.8 Hz, 2H, 6-CH_2_), 2.79–2.93 (m, 3H, 5-CH_2_, CH̲(CH_3_)_2_), 3.40 (s, 2H, 8-CH_2_), 3.50–3.59 (m, 4H, N(CH_2_)_2_), 4.02 (br s, 2H, NH_2_), 4.69 (s, 2H, OCH_2_), 8.89 (s, 1H, NH). ^13^C NMR (75 MHz, DMSO-*d*_6_/CCl_4_, 1/3) *δ* 18.1, 26.3, 28.1, 29.1, 44.3, 49.5, 50.7, 53.4, 63.2, 82.9, 110.9, 115.0, 146.6, 150.4, 158.4, 159.6, 166.5. Anal. calcd for C_20_H_30_N_6_O_2_: C 62.15; H 7.82; N 21.74%. Found: C 61.83; H 7.96; N 21.51%.

##### 3.1.2.4 2-[(4-Cyano-7-isopropyl-1-morpholin-4-yl-5,6,7,8-tetrahydro-2,7-naphthyridin-3-yl)oxy]acetohydrazide (3d)

Cream solid, yield 87%, m.p. 188–190 °C. IR *ν*/cm^−1^: 1689 (C=O), 2207 (C≡N), 3289, 3332 (NH, NH_2_). ^1^H NMR (300 MHz, DMSO-*d*_6_/CCl_4_, 1/3) *δ* 1.08 (d, *J* = 6.6, 6H, CH(CH̲_3_)_2_), 2.73 (t, *J* = 5.9 Hz, 2H, 6-CH_2_), 2.86 (sp, *J* = 6.6 Hz, 1H, CH̲(CH_3_)_2_), 2.88 (t, *J* = 6.1 Hz, 2H, 5-CH_2_), 3.22–3.32 (m, 4H, N(CH_2_)_2_), 3.35 (s, 2H, 8-CH_2_), 3.66–3.73 (m, 4H, O(CH_2_)_2_), 4.02 (br s, 2H, NH_2_), 4.70 (s, 2H, OCH_2_), 9.02 (s, 1H, NH). ^13^C NMR (75 MHz, DMSO-*d*_6_/CCl_4_, 1/3) *δ* 18.1, 28.8, 44.3, 48.4, 48.8, 53.2, 63.4, 63.5, 65.9, 86.1, 114.2, 114.3, 151.2, 158.4, 160.2, 166.5. Anal. calcd for C_18_H_26_N_6_O_3_: C 57.74; H 7.00; N 22.44%. Found: C 58.10; H 6.83; N 22.20%.

##### 3.1.2.5 2-[(7-Benzyl-4-cyano-1-pyrrolidin-1-yl-5,6,7,8-tetrahydro-2,7-naphthyridin-3-yl)oxy]acetohydrazide (3e)

Cream solid, yield 78%, m.p. 143–145 °C. IR *ν*/cm^−1^: 1698 (C=O), 2205 (C≡N), 3231, 3293 (NH, NH_2_). ^1^H NMR (300 MHz, DMSO-*d*_6_/CCl_4_, 1/3) *δ* 1.83–1.94 (m, 4H, C_4_H_8_N), 2.66 (t, *J* = 5.9 Hz, 2H, 6-CH_2_), 2.83 (t, *J* = 5.8 Hz, 2H, 5-CH_2_), 3.47–3.57 (m, 6H, 8-CH_2_, N(CH_2_)_2_), 4.02 (br s, 2H, NH_2_), 3.65 (s, 2H, CH̲_2_Ph), 4.67 (s, 2H, OCH_2_), 7.17–7.33 (m, 5H, Ph), 8.90 (s, 1H, NH). ^13^C NMR (75 MHz, DMSO-*d*_6_/CCl_4_, 1/3) *δ* 24.9, 28.4, 48.0, 49.2, 53.0, 61.7, 63.2, 81.3, 108.9, 115.2, 126.6, 127.7, 128.2, 137.5, 149.2, 156.2, 160.1, 166.6. Anal. calcd for C_22_H_26_N_6_O_2_: C 65.01; H 6.45; N 20.68%. Found: C 65.35; H 6.63; N 20.94%.

##### 3.1.2.6 2-[(7-Benzyl-4-cyano-1-piperidin-1-yl-5,6,7,8-tetrahydro-2,7-naphthyridin-3-yl)oxy]acetohydrazide (3f)

Colorless solid, yield 85%, m.p. 169–171 °C. IR *ν*/cm^−1^: 1684 (C=O), 2204 (C≡N), 3315 (NH, NH_2_). ^1^H NMR (300 MHz, DMSO-*d*_6_/CCl_4_, 1/3) *δ* 1.51–1.67 (m, 6H, C_5_H_10_N), 2.71 (t, *J* = 6.1 Hz, 2H, 6-CH_2_), 2.89 (t, *J* = 6.0 Hz, 2H, 5-CH_2_), 3.15–3.23 (m, 4H, N(CH_2_)_2_), 3.28 (s, 2H, 8-CH_2_), 3.65 (s, 2H, CH̲_2_Ph), 4.01 (br s, 2H, NH_2_), 4.71 (s, 2H, OCH_2_), 7.16–7.33 (m, 5H, Ph), 8.96 (s, 1H, NH). ^13^C NMR (75 MHz, DMSO-*d*_6_/CCl_4_, 1/3) *δ* 23.9, 25.3, 28.2, 48.6, 49.3, 52.8, 61.6, 63.3, 85.2, 113.1, 114.5, 126.6, 127.7, 128.3, 137.4, 150.3, 159.1, 160.2, 166.4. Anal.calcd forC_23_H_28_N_6_O_2_: C 65.69; H 6.71; N 19.99%. Found: C 66.07; H 6.84; N 20.21%.

##### 3.1.2.7 2-[(1-Azepan-1-yl-7-benzyl-4-cyano-5,6,7,8-tetrahydro-2,7-naphthyridin-3-yl)oxy]acetohydrazide (3g)

Cream solid, yield 83%, m.p. 130–132 °C. IR *ν*/cm^−1^: 1693 (C=O), 2205 (C≡N), 3292, 3345 (NH, NH_2_). ^1^H NMR (300 MHz, DMSO-*d*_6_/CCl_4_, 1/3) *δ* 1.43–1.55 (m, 4H, C_6_H_12_N), 1.62–1.75 (m, 4H, C_6_H_12_N), 2.70 (t, *J* = 5.9 Hz, 2H, 6-CH_2_), 2.88 (t, *J* = 5.8 Hz, 2H, 5-CH_2_), 3.32 (s, 2H, 8-CH_2_), 3.44–3.52 (m, 4H, N(CH_2_)_2_), 3.65 (s, 2H, CH̲_2_Ph), 4.01 (br s, 2H, NH_2_), 4.68 (s, 2H, OCH_2_), 7.16–7.33 (m, 5H, Ph), 8.91 (s, 1H, NH). ^13^C NMR (75 MHz, DMSO-*d*_6_/CCl_4_, 1/3) *δ* 26.2, 28.0, 28.6, 48.4, 50.6, 53.8, 61.8, 63.2, 82.7, 110.0, 115.0, 126.6, 127.7, 128.3, 137.5, 150.0, 158.2, 159.8, 166.5. Anal. calcd for C_24_H_30_N_6_O_2_: C 66.34; H 6.96; N 19.34%. Found: C 66.69; H 7.15; N 19.09%.

##### 3.1.2.8 2-[(7-Benzyl-4-cyano-1-morpholin-4-yl-5,6,7,8-tetrahydro-2,7-naphthyridin-3-yl)oxy]acetohydrazide (3h)

Colorless solid, yield 81%, m.p. 182–184 °C. IR *ν*/cm^−1^: 1671 (C=O), 2208 (C≡N), 3292, 3327 (NH, NH_2_). ^1^H NMR (300 MHz, DMSO-*d*_6_/CCl_4_, 1/3) *δ* 2.71 (t, *J* = 6.1 Hz, 2H, 6-CH_2_), 2.91 (t, *J* = 5.9 Hz, 2H, 5-CH_2_), 3.19–3.25 (m, 4H, N(CH_2_)_2_), 3.31 (s, 2H, 8-CH_2_), 3.59–3.66 (m, 4H, O(CH_2_)_2_), 3.65 (s, 2H, CH̲_2_Ph), 4.03 (br s, 2H, NH_2_), 4.70 (s, 2H, OCH_2_),7.19–7.34 (m, 5H, Ph), 9.03 (s, 1H, NH). ^13^C NMR (75 MHz, DMSO-*d*_6_/CCl_4_, 1/3) *δ* 28.2, 48.4, 48.7, 52.6, 61.5, 63.5, 65.8, 86.0, 113.3, 114.2, 126.6, 127.7, 128.3, 137.4, 150.8, 158.3, 160.3, 166.4. Anal. calcd for C_22_H_26_N_6_O_3_: C 62.54; H 6.20; N 19.89%. Found: C 62.87; H 6.39; N 19.65%.

#### 3.1.3. General procedure for the synthesis of compounds 4a–h

A mixture of compound 3 (5 mmol) and phenylisothiocyanate (5 mmol) in absolute ethanol was stirred for 8 h at room temperature. After the precipitate was filtered off, washed with ethanol, dried and recrystallized from ethanol.

##### 3.1.3.1 2-{[(4-Cyano-7-isopropyl-1-pyrrolidin-1-yl-5,6,7,8-tetrahydro-2,7-naphthyridin-3-yl)oxy]acetyl}-*N*-phenylhydrazinecarbothioamide (4a)

Colorless solid, yield 89%, m.p. 197–199 °C. IR *ν*/cm^−1^: 1630 (C=O), 2210 (C≡N), 3129, 3425 (NH). ^1^H NMR (300 MHz, DMSO-*d*_6_/CCl_4_, 1/3) *δ* 1.09 (d, *J* = 6.3 Hz, 6H, CH(CH̲_3_)_2_), 1.78–1.89 (m, 4H, C_4_H_8_N), 2.65–2.98 (m, 5H, 5,6-CH_2_, CH̲(CH_3_)_2_), 3.49–3.63 (m, 6H, 8-CH_2_, N(CH_2_)_2_), 4.85 (s, 2H, OCH_2_), 7.09–7.16 (m, 1H, Ph), 7.25–7.34 (m, 2H, Ph), 7.40–7.49 (m, 2H, Ph), 9.26 (br, 1H, NH), 9.59 (br, 1H, NH), 10.06 (s, 1H, NH). Anal. calcd for C_25_H_31_N_7_O_2_S: C 60.83; H 6.33; N 19.86%. Found: C 61.20; H 6.49; N 20.13%.

##### 3.1.3.2 2-{[(4-Cyano-7-isopropyl-1-piperidin-1-yl-5,6,7,8-tetrahydro-2,7-naphthyridin-3-yl)oxy]acetyl}-*N*-phenylhydrazinecarbothioamide (4b)

Colorless solid, yield 92%, m.p. 174–176 °C. IR *ν*/cm^−1^: 1632 (C=O), 2210 (C≡N), 3299 (NH). ^1^H NMR (300 MHz, DMSO-*d*_6_/CCl_4_, 1/3) *δ* 1.09 (d, *J* = 6.4 Hz, 6H, CH(CH̲_3_)_2_), 1.59–1.68 (m, 6H, C_5_H_10_N), 2.75 (t, *J* = 5.8 Hz, 2H, 6-CH_2_), 2.80–2.97 (m, 3H, 5-CH_2_, CH̲(CH_3_)_2_), 3.17–3.27 (m, 4H, N(CH_2_)_2_), 3.34 (s, 2H, 8-CH_2_), 4.90 (s, 2H, OCH_2_), 7.09–7.14 (m, 1H, Ph), 7.23–7.33 (m, 2H, Ph), 7.46–7.55 (m, 2H, Ph), 9.36 (br, 1H, NH), 9.54 (br, 1H, NH), 10.04 (s, 1H, NH). ^13^C NMR (75 MHz, DMSO-*d*_6_/CCl_4_, 1/3) *δ* 18.2, 24.0, 25.5, 28.8, 44.7, 48.5, 49.4, 53.3, 63.2, 85.2, 114.1, 114.7, 124.0, 124.1, 124.2, 127.5, 138.9, 150.7, 159.3, 160.2, 180.3. Anal. calcd for C_26_H_33_N_7_O_2_S: C 61.51; H 6.55; N 19.31%. Found: C 61.17; H 6.40; N 19.54%.

##### 3.1.3.3 2-{[(1-Azepan-1-yl-4-cyano-7-isopropyl-5,6,7,8-tetrahydro-2,7-naphthyridin-3-yl)oxy]acetyl}-*N*-phenylhydrazinecarbothioamide (4c)

Yellow solid, yield 91%, m.p. 146–148 °C. IR *ν*/cm^−1^: 1635 (C=O), 2211 (C≡N), 3279 (NH). ^1^H NMR (300 MHz, DMSO-*d*_6_/CCl_4_, 1/3) *δ* 1.09 (d, *J* = 6.5 Hz, 6H, CH(CH̲_3_)_2_), 1.49–1.63 (m, 4H, C_6_H_12_N), 1.70–1.84 (m, 4H, C_6_H_12_N), 2.72 (t, *J* = 5.9 Hz, 2H, 6-CH_2_), 2.80–2.95 (m, 3H, 5-CH_2_, CH̲(CH_3_)_2_), 3.39 (s, 2H, 8-CH_2_), 3.47–3.57 (m, 4H, N(CH_2_)_2_), 4.87 (s, 2H, OCH_2_), 7.06–7.13 (m, 1H, Ph), 7.23–7.32 (m, 2H, Ph), 7.46–7.54 (m, 2H, Ph), 9.33 (br, 1H, NH), 9.50 (br, 1H, NH), 10.03 (s, 1H, NH). ^13^C NMR (75 MHz, DMSO-*d*_6_/CCl_4_, 1/3) *δ* 18.1, 26.4, 28.1, 29.2, 44.3, 49.6, 50.7, 53.4, 63.1, 82.8, 104.5, 111.1, 115.1, 124.0, 124.1, 127.4, 138.8, 150.4, 158.6, 159.7, 180.2. Anal. calcd for C_27_H_35_N_7_O_2_S: C 62.16; H 6.76; N 18.79%. Found: C 62.52; H 6.62; N 18.52%.

##### 3.1.3.4 2-{[(4-Cyano-7-isopropyl-1-morpholin-4-yl-5,6,7,8-tetrahydro-2,7-naphthyridin-3-yl)oxy]acetyl}-*N*-phenylhydrazinecarbothioamide (4d)

Colorless solid, yield 93%, m.p. 191–192 °C. IR *ν*/cm^−1^: 1702, 1726 (C=O), 2222 (C≡N), 3281 (NH). ^1^H NMR (300 MHz, DMSO-*d*_6_/CCl_4_, 1/3) *δ* 1.08 (d, *J* = 6.6 Hz, 6H, CH(CH̲_3_)_2_), 2.75 (t, *J* = 5.7 Hz, 2H, 6-CH_2_), 2.79–2.96 (m, 3H, 5-CH_2_, CH̲(CH_3_)_2_), 3.18–3.31 (m, 4H, N(CH_2_)_2_), 3.36 (s, 2H, 8-CH_2_), 3.63–3.74 (m, 4H, O(CH_2_)_2_), 4.90 (s, 2H, OCH_2_), 7.06–7.15 (m, 1H, Ph), 7.23–7.33 (m, 2H, Ph), 7.47–7.60 (m, 2H, Ph), 9.40 (br, 1H, NH), 9.57 (br, 1H, NH), 10.04 (s, 1H, NH). ^13^C NMR (75 MHz, DMSO-*d*_6_/CCl_4_, 1/3) *δ* 18.1, 28.9, 44.4, 48.5, 48.8, 53.3, 63.4, 66.0, 78.4, 86.1, 114.3, 114.4, 124.2, 124.2, 127.5, 138.9, 151.2, 158.6, 160.2, 180.4. Anal. calcd for C_25_H_31_N_7_O_3_S: C 58.92; H 6.13; N 19.24%. Found: C 58.57; H 6.29; N 19.50%.

##### 3.1.3.5 2-{[(7-Benzyl-4-cyano-1-pyrrolidin-1-yl-5,6,7,8-tetrahydro-2,7-naphthyridin-3-yl)oxy]acetyl}-*N*-phenylhydrazinecarbothioamide (4e)

Cream solid, yield 88%, m.p. 171–173 °C. IR *ν*/cm^−1^: 1706 (C=O), 2206 (C≡N), 3147, 3307 (NH). ^1^H NMR (300 MHz, DMSO-*d*_6_/CCl_4_, 1/3) *δ* 1.62–1.85 (m, 4H, C_4_H_8_N), 2.69 (t, *J* = 5.9 Hz, 2H, 6-CH_2_), 2.86 (t, *J* = 5.8 Hz, 2H, 5-CH_2_), 3.41–3.50 (m, 4H, N(CH_2_)_2_), 3.51 (s, 2H, 8-CH_2_), 3.66 (s, 2H, CH̲_2_Ph), 4.84 (s, 2H, OCH_2_), 7.04–7.11 (m, 1H, Ph), 7.19–7.32 (m, 7H, Ph), 7.40–7.48 (m, 2H, Ph), 9.11 (br, 1H, NH), 9.54 (br, 1H, NH), 10.02 (s, 1H, NH). ^13^C NMR (75 MHz, DMSO-*d*_6_/CCl_4_, 1/3) *δ* 24.8, 28.5, 48.1, 49.2, 53.0, 61.7, 63.4, 63.4, 81.2, 109.0, 115.3, 123.9, 124.2, 126.6, 127.5, 127.8, 128.3, 137.6, 138.7, 149.2, 156.1, 160.3, 166.3, 180.2. Anal. calcd for C_29_H_31_N_7_O_2_S: C 64.30; H 5.77; N 18.10%. Found: C 64.62; H 5.93; N 17.86%.

##### 3.1.3.6 2-{[(7-Benzyl-4-cyano-1-piperidin-1-yl-5,6,7,8-tetrahydro-2,7-naphthyridin-3-yl)oxy]acetyl}-*N*-phenylhydrazinecarbothioamide (4f)

Cream solid, yield 82%, m.p. 137–139 °C. IR *ν*/cm^−1^: 1712 (C=O), 2215 (C≡N), 3165, 3239 (NH). ^1^H NMR (300 MHz, DMSO-*d*_6_/CCl_4_, 1/3) *δ* 1.41–1.63 (m, 6H, C_5_H_10_N), 2.73 (t, *J* = 5.8 Hz, 2H, 6-CH_2_), 2.91 (t, *J* = 5.7 Hz, 2H, 5-CH_2_), 3.08–3.22 (m, 4H, N(CH_2_)_2_), 3.28 (s, 2H, 8-CH_2_), 3.66 (s, 2H, CH̲_2_Ph), 4.90 (s, 2H, OCH_2_),7.05–7.13 (m, 1H, Ph), 7.19–7.33 (m, 7H, Ph), 7.40–7.61 (m, 2H, Ph), 9.33 (br, 1H, NH), 9.50 (br, 1H, NH), 10.04 (s, 1H, NH). ^13^C NMR (75 MHz, DMSO-*d*_6_/CCl_4_, 1/3) *δ* 24.0, 25.4, 28.3, 48.7, 49.3, 52.9, 61.7, 63.3, 85.1, 113.2, 114.7, 124.2, 126.7, 127.5, 127.8, 128.4, 137.4, 138.8, 150.3, 159.2, 160.4, 166.3, 180.3. Anal. calcd forC_30_H_33_N_7_O_2_S: C 64.84; H 5.99; N 17.64%. Found: C 64.50; H 6.14; N 17.39%.

##### 3.1.3.7 2-{[(1-Azepan-1-yl-7-benzyl-4-cyano-5,6,7,8-tetrahydro-2,7-naphthyridin-3-yl)oxy]acetyl}-*N*-phenylhydrazinecarbothioamide (4g)

Yellow solid, yield 85%, m.p. 256–258 °C. IR *ν*/cm^−1^: 1661, 1678 (C=O), 2208 (C≡N), 3156, 3267 (NH). ^1^H NMR (300 MHz, DMSO-*d*_6_/CCl_4_, 1/3) *δ* 1.40–1.54 (m, 4H, C_6_H_12_N), 1.59–1.75 (m, 4H, C_6_H_12_N), 2.72 (t, *J* = 5.9 Hz, 2H, 6-CH_2_), 2.89 (t, *J* = 5.8 Hz, 2H, 5-CH_2_), 3.32 (s, 2H, 8-CH_2_), 3.40–3.49 (m, 4H, N(CH_2_)_2_), 3.65 (s, 2H, CH̲_2_Ph), 4.87 (s, 2H, OCH_2_), 7.05–7.12 (m, 1H, Ph), 7.18–7.32 (m, 7H, Ph), 7.45–7.58 (m, 2H, Ph), 9.31 (br, 1H, NH), 9.48 (br, 1H, NH), 10.03 (s, 1H, NH). ^13^C NMR (75 MHz, DMSO-*d*_6_/CCl_4_, 1/3) *δ* 26.2, 28.0, 28.6, 48.5, 50.6, 53.8, 61.8, 62.6, 82.7, 110.1, 115.1, 124.1, 124.1, 126.6, 127.4, 127.7, 128.3, 137.5, 138.8, 150.0, 158.3, 159.8, 166.1, 180.3. Anal. calcd for C_31_H_35_N_7_O_2_S: C 65.35; H 6.19; N 17.21%. Found: C 65.72; H 6.35; N 17.47%.

##### 3.1.3.8 2-{[(7-Benzyl-4-cyano-1-morpholin-4-yl-5,6,7,8-tetrahydro-2,7-naphthyridin-3-yl)oxy]acetyl}-*N*-phenylhydrazinecarbothioamide (4h)

Colorless solid, yield 88%, m.p. 191–192 °C. IR *ν*/cm^−1^: 1700 (C=O), 2213 (C≡N), 3224, 3304 (NH). ^1^H NMR (300 MHz, DMSO-*d*_6_/CCl_4_, 1/3) *δ* 2.73 (t, *J* = 5.8 Hz, 2H, 6-CH_2_), 2.93 (t, *J* = 5.9 Hz, 2H, 5-CH_2_), 3.15–3.26 (m, 4H, N(CH_2_)_2_), 3.31 (s, 2H, 8-CH_2_), 3.56–3.63 (m, 4H, O(CH_2_)_2_), 3.66 (s, 2H, CH̲_2_Ph),4.90 (s, 2H, OCH_2_),7.07–7.14 (m, 1H, Ph), 7.18–7.34 (m, 7H, Ph), 7.46–7.60 (m, 2H, Ph), 9.39 (br, 1H, NH), 9.55 (br, 1H, NH), 10.05 (s, 1H, NH). ^13^C NMR (75 MHz, DMSO-*d*_6_/CCl_4_, 1/3) *δ* 28.2, 48.5, 48.6, 52.7, 61.5, 63.4, 65.8, 85.9, 113.3, 114.3, 124.1, 124.3, 126.6, 127.4, 127.7, 128.3, 137.4, 138.8, 150.7, 158.4, 160.3, 166.4, 180.4. Anal. calcd for C_29_H_31_N_7_O_3_S: C 62.46; H 5.60; N 17.58%. Found: C 62.13; H 5.74; N 17.35%.

#### 3.1.4. General procedure for the synthesis of compounds 5a–h

To a stirred solution of NaOH (10 mmol) in water (30 mL) the corresponding thiosemicarbazide 4 (5 mmol) was added, and the mixture was stirred at 40–50 °C for 5 h. After the mixture was diluted with water, and acidified with hydrochloric acid to pH 5–6. The formed crystals were filtered off, washed with water, dried and recrystallized from ethanol.

##### 3.1.4.1 7-Isopropyl-3-[(4-phenyl-5-thioxo-4,5-dihydro-1*H*-1,2,4-triazol-3-yl)methoxy]-1-pyrrolidin-1-yl-5,6,7,8-tetrahydro-2,7-naphthyridine-4-carbonitrile (5a)

Cream solid, yield 81%, m.p. 203–204 °C. IR *ν*/cm^−1^: 1600 (C=C_ar_), 2208 (C≡N). ^1^H NMR (300 MHz, DMSO-*d*_6_/CCl_4_, 1/3) *δ* 1.12 (d, *J* = 6.5 Hz, 6H, CH(CH̲_3_)_2_), 1.86–1.98 (m, 4H, C_4_H_8_N), 2.61–3.10 (m, 5H, 5,6-CH_2_, CH̲(CH_3_)_2_), 3.49–3.68 (m, 6H, 8-CH_2_, N(CH_2_)_2_), 5.19 (s, 2H, OCH_2_), 7.37–7.54 (m, 5H, Ph), 13.84 (br, 1H, NH). ^13^C NMR (75 MHz, DMSO-*d*_6_/CCl_4_, 1/3) *δ* 18.0, 24.9, 28.4, 43.7, 48.1, 49.3, 53.8, 56.7, 81.1, 114.7, 127.9, 128.6, 128.9, 132.9, 147.1, 149.3, 156.1, 159.3, 168.7. Anal. calcd for C_25_H_29_N_7_OS: C 63.13; H 6.15; N 20.62%. Found: C 63.45; H 5.98; N 20.38%.

##### 3.1.4.2 7-Isopropyl-3-[(4-phenyl-5-thioxo-4,5-dihydro-1*H*-1,2,4-triazol-3-yl)methoxy]-1-piperidin-1-yl-5,6,7,8-tetrahydro-2,7-naphthyridine-4-carbonitrile (5b)

Cream solid, yield 83%, m.p. 192–194 °C. IR *ν*/cm^−1^: 1597 (C=C_ar_), 2209 (C≡N). ^1^H NMR (300 MHz, DMSO-*d*_6_/CCl_4_, 1/3) *δ* 1.08 (d, *J* = 6.6 Hz, 6H, CH(CH̲_3_)_2_), 1.58–1.70 (m, 6H, C_5_H_10_N), 2.72 (t, *J* = 5.7 Hz, 2H, 6-CH_2_), 2.77–2.92 (m, 3H, 5-CH_2_, CH̲(CH_3_)_2_), 3.16–3.25 (m, 4H, N(CH_2_)_2_), 3.30 (s, 2H, 8-CH_2_), 5.21 (s, 2H, OCH_2_), 7.38–7.53 (m, 5H, Ph), 13.86 (br, 1H, NH). ^13^C NMR (75 MHz, DMSO-*d*_6_/CCl_4_, 1/3) *δ* 18.1, 23.9, 25.3, 28.7, 44.5, 48.4, 49.4, 53.2, 56.8, 85.0, 114.0, 114.4, 128.0, 128.6, 128.9, 132.9, 146.9, 150.9, 159.0, 159.2, 168.7. Anal. calcd for C_26_H_31_N_7_OS: C 63.78; H 6.38; N 20.02%. Found: C 64.14; H 6.56; N 20.30%.

##### 3.1.4.3 1-Aazepan-1-yl-7-isopropyl-3-[(4-phenyl-5-thioxo-4,5-dihydro-1*H*-1,2,4-triazol-3-yl)methoxy]-5,6,7,8-tetrahydro-2,7-naphthyridine-4-carbonitrile (5c)

Colorless solid, yield 90%, m.p. 196–198 °C. IR *ν*/cm^−1^: 1601 (C=C_ar_), 2210 (C≡N). ^1^H NMR (300 MHz, DMSO-*d*_6_/CCl_4_, 1/3) *δ* 1.07 (d, *J* = 6.5 Hz, 6H, CH(CH̲_3_)_2_), 1.50–1.61 (m, 4H, C_6_H_12_N), 1.70–1.81 (m, 4H, C_6_H_12_N), 2.69 (t, *J* = 5.8 Hz, 2H, 6-CH_2_), 2.81 (t, *J* = 5.9 Hz, 2H, 5-CH_2_), 2.83 (sp, *J* = 6.5 Hz, 1H, CH̲(CH_3_)_2_), 3.36 (s, 2H, 8-CH_2_), 3.48–3.55 (m, 4H, N(CH_2_)_2_), 5.17 (s, 2H, OCH_2_), 7.37–7.54 (m, 5H, Ph), 13.85 (br, 1H, NH). ^13^C NMR (75 MHz, DMSO-*d*_6_/CCl_4_, 1/3) *δ* 18.1, 26.2, 28.0, 29.1, 44.2, 49.5, 50.7, 53.3, 56.7, 82.7, 111.4, 114.4, 127.9, 128.6, 128.8, 132.9, 147.0, 150.7, 158.3, 158.8, 168.7. Anal. calcd for C_27_H_33_N_7_OS: C 64.39; H 6.60; N 19.47%. Found: C 64.04; H 6.75; N 19.22%.

##### 3.1.4.4 7-Isopropyl-1-morpholin-4-yl-3-[(4-phenyl-5-thioxo-4,5-dihydro-1*H*-1,2,4-triazol-3-yl)methoxy]-5,6,7,8-tetrahydro-2,7-naphthyridine-4-carbonitrile (5d)

Yellow solid, yield 76%, m.p. 204–206 °C. IR *ν*/cm^−1^: 1593 (C=C_ar_), 2221 (C≡N). ^1^H NMR (300 MHz, DMSO-*d*_6_/CCl_4_, 1/3) *δ* 1.07 (d, *J* = 6.6 Hz, 6H, CH(CH̲_3_)_2_), 2.71 (t, *J* = 5.8 Hz, 2H, 6-CH_2_), 2.80–2.90 (m, 3H, 5-CH_2_, CH̲(CH_3_)_2_), 3.21–3.27 (m, 4H, N(CH_2_)_2_), 3.32 (s, 2H, 8-CH_2_), 3.65–3.73 (m, 4H, N(CH_2_)_2_), 5.23 (s, 2H, OCH_2_), 7.38–7.54 (m, 5H, Ph), 13.77 (br, 1H, NH). ^13^C NMR (75 MHz, DMSO-*d*_6_/CCl_4_, 1/3) *δ* 18.0, 28.8, 44.2, 48.4, 48.8, 53.2, 57.0, 65.8, 86.0, 113.7, 114.7, 128.0, 128.7, 128.9, 132.9, 146.9, 151.5, 158.3, 159.2, 168.8. Anal. calcd for C_25_H_29_N_7_O_2_S: C 61.08; H 5.95; N 19.94%. Found: C 60.74; H 6.08; N 19.72%.

##### 3.1.4.5 7-Benzyl-3-[(4-phenyl-5-thioxo-4,5-dihydro-1*H*-1,2,4-triazol-3-yl)methoxy]-1-pyrrolidin-1-yl-5,6,7,8-tetrahydro-2,7-naphthyridine-4-carbonitrile (5e)

Yellow solid, yield 87%, m.p. 140–142 °C. IR *ν*/cm^−1^: 1598 (C=C_ar_), 2207 (C≡N). ^1^H NMR (300 MHz, DMSO-*d*_6_/CCl_4_, 1/3) *δ* 1.82–1.94 (m, 4H, C_4_H_8_N), 2.64 (t, *J* = 5.9 Hz, 2H, 6-CH_2_), 2.79 (t, *J* = 5.8 Hz, 2H, 5-CH_2_), 3.43–3.54 (m, 6H, 8-CH_2_, N(CH_2_)_2_), 3.64 (s, 2H, CH̲_2_Ph), 5.18 (s, 2H, OCH_2_), 7.16–7.33 (m, 5H, Ph), 7.37–7.54 (m, 5H, Ph), 13.85 (br, 1H, NH). ^13^C NMR (75 MHz, DMSO-*d*_6_/CCl_4_, 1/3) *δ* 24.8, 28.3, 47.9, 49.2, 52.9, 56.6, 61.6, 81.1, 109.4, 114.7, 126.6, 127.7, 128.0, 128.1, 128.6, 128.8, 132.9, 137.5, 147.1, 149.3, 156.0, 159.3, 168.7. Anal. calcd for C_29_H_29_N_7_OS: C 66.52; H 5.58; N 18.72%. Found: C 66.90; H 5.74; N 18.95%.

##### 3.1.4.6 7-Benzyl-3-[(4-phenyl-5-thioxo-4,5-dihydro-1*H*-1,2,4-triazol-3-yl)methoxy]-1-piperidin-1-yl-5,6,7,8-tetrahydro-2,7-naphthyridine-4-carbonitrile (5f)

Yellow solid, yield 83%, m.p. 124–126 °C. IR *ν*/cm^−1^: 1594 (C=C_ar_), 2213 (C≡N). ^1^H NMR (300 MHz, DMSO-*d*_6_/CCl_4_, 1/3) *δ* 1.49–1.65 (m, 6H, C_5_H_10_N), 2.69 (t, *J* = 5.8 Hz, 2H, 6-CH_2_), 2.85 (t, *J* = 5.9 Hz, 2H, 5-CH_2_), 3.10–3.21 (m, 4H, N(CH_2_)_2_), 3.28 (s, 2H, 8-CH_2_), 3.63 (s, 2H, CH̲_2_Ph), 5.21 (s, 2H, OCH_2_), 7.19–7.32 (m, 5H, Ph), 7.38–7.53 (m, 5H, Ph), 13.87 (br, 1H, NH). ^13^C NMR (75 MHz, DMSO-*d*_6_/CCl_4_, 1/3) *δ* 23.9, 25.3, 28.2, 48.5, 49.3, 52.7, 56.9, 61.5, 84.8, 113.5, 114.0, 126.7, 127.7, 128.0, 128.3, 128.7, 128.9, 132.9, 137.3, 147.0, 150.5, 158.9, 159.4, 168.8. Anal. calcd for C_30_H_31_N_7_OS: C 67.01; H 5.81; N 18.24%. Found: C 66.70; H 5.96; N 18.00%.

##### 3.1.4.7 1-Azepan-1-yl-7-benzyl-3-[(4-phenyl-5-thioxo-4,5-dihydro-1*H*-1,2,4-triazol-3-yl)methoxy]-5,6,7,8-tetrahydro-2,7-naphthyridine-4-carbonitrile (5g)

Yellow solid, yield 81%, m.p. 135–137 °C. IR *ν*/cm^−1^: 1596 (C=C_ar_), 2210 (C≡N). ^1^H NMR (300 MHz, DMSO-*d*_6_/CCl_4_, 1/3) *δ* 1.41–1.52 (m, 4H, C_6_H_12_N), 1.59–1.72 (m, 4H, C_6_H_12_N), 2.68 (t, *J* = 6.0 Hz, 2H, 6-CH_2_), 2.85 (t, *J* = 6.0 Hz, 2H, 5-CH_2_), 3.28 (s, 2H, 8-CH_2_), 3.41–3.49 (m, 4H, N(CH_2_)_2_), 3.64 (s, 2H, CH̲_2_Ph), 5.17 (s, 2H, OCH_2_), 7.17–7.33 (m, 5H, Ph), 7.37–7.55 (m, 5H, Ph), 13.86 (br, 1H, NH). ^13^C NMR (75 MHz, DMSO-*d*_6_/CCl_4_, 1/3) *δ* 26.1, 27.9, 28.5, 48.3, 50.6, 53.7, 56.7, 61.7, 82.5, 110.4, 114.4, 126.6, 127.7, 127.9, 128.2, 128.6, 128.8, 132.9, 137.4, 147.0, 150.2, 158.1, 158.9, 168.7. Anal. calcd for C_31_H_33_N_7_OS: C 67.49; H 6.03; N 17.77%. Found: C 67.12; H 5.85; N 18.03%.

##### 3.1.4.8 7-Benzyl-1-morpholin-4-yl-3-[(4-phenyl-5-thioxo-4,5-dihydro-1*H*-1,2,4-triazol-3-yl)methoxy]-5,6,7,8-tetrahydro-2,7-naphthyridine-4-carbonitrile (5h)

Yellow solid, yield 90%, m.p. 182–184 °C. IR *ν*/cm^−1^: 1590 (C=C_ar_), 2221 (C≡N). ^1^H NMR (300 MHz, DMSO-*d*_6_/CCl_4_, 1/3) *δ* 2.69 (t, *J* = 6.1 Hz, 2H, 6-CH_2_), 2.86 (t, *J* = 5.9 Hz, 2H, 5-CH_2_), 3.17–3.23 (m, 4H, N(CH_2_)_2_), 3.28 (s, 2H, 8-CH_2_), 3.58–3.64 (m, 4H, O(CH_2_)_2_), 3.64 (s, 2H, CH̲_2_Ph), 5.23 (s, 2H, OCH_2_),7.18–7.32 (m, 5H, CH_2_**Ph**), 7.38–7.54 (m, 5H, NPh), 13.87 (s, 1H, NH). ^13^C NMR (75 MHz, DMSO-*d*_6_/CCl_4_, 1/3) *δ* 28.2, 48.3, 48.7, 52.6, 57.0, 61.4, 65.7, 85.8, 113.8, 113.8, 126.7, 127.7, 128.0, 128.3, 128.7, 128.9, 132.9, 137.3, 146.9, 151.1, 158.2, 159.4, 168.7. Anal. calcd for C_29_H_29_N_7_O_2_S: C 64.54; H 5.42; N 18.17%. Found: C 64.90; H 5.59; N 18.42%.

#### 3.1.5. General procedure for the synthesis of compounds 7a,b

1,5-Dialkyl-9-oxo-3,7-diazabicyclo[3.3.1]nonane (10 mmol) 6 were dissolved in absolute methanol (30 mL) and a freshly prepared solution of dimethyl oxalate (10 mmol) in absolute methanol was added with stirring at room temperature. After 30 min, the precipitated crystals were filtered off, washed with methanol and recrystallized from methanol.

##### 3.1.5.1 Methyl(1,5-dimethyl-9-oxo-3,7-diazabicyclo[3.3.1]non-3-yl)(oxo)acetate (7a)

Colorless solid, yield 69%, m.p. 145–146 °C. IR *ν*/cm^−1^: 1708, 1744, 1760 (C=O), 3392 (NH). ^1^H NMR (400 MHz, CDCl_3_) *δ* 0.96 (s, 3H, CH_3_), 1.00 (s, 3H, CH_3_), 2.08 (s, 1H, NH), 2.87–2.98 (m, 3H), 3.32–3.45 (m, 3H), 3.88–3.96 (m, 1H), 3.95 (s, 3H, OCH_3_), 4.76–4.82 (m, 1H). ^13^C NMR (100 MHz, CDCl_3_) *δ* 16.70, 16.86, 48.03, 48.28, 53.06, 53.08, 57.25, 61.37, 61.62, 160.41, 163.11, 212.50. Anal. calcd for C_12_H_18_N_2_O_4_: C 56.68; H 7.13; N 11.02%. Found: C 56.36; H 7.24; N 11.13%.

##### 3.1.5.2 Methyl(1,5-diethyl-9-oxo-3,7-diazabicyclo[3.3.1]non-3-yl)(oxo)acetate (7b)

Colorless solid, yield 71%, m.p. 130–131 °C. IR *ν*/cm^−1^: 1650, 1704, 1747 (C=O), 3401 (NH). ^1^H NMR (400 MHz, CDCl_3_) *δ* 0.85 (t, *J* = 7.4 Hz, 3H, CH_2_CH̲_3_), 0.90 (t, *J* = 7.4 Hz, 3H, CH_2_CH̲_3_), 1.31–1.59 (m, 4H, 2CH̲_2_CH_3_), 1.99 (s, 1H, NH), 2.66–2.77 (m, 2H), 2.96–3.00 (m, 1H), 3.29–3.42 (m, 2H), 3.79–3.80 (m, 4H), 4.40 (m, 1H). ^13^C NMR (100 MHz, CDCl_3_) *δ* 7.67, 7.74, 23.94, 24.23, 50.2, 50.67, 50.78, 53.00, 54.90, 59.30, 59.65, 160.63, 163.20, 212.64. Anal. calcd for C_14_H_22_N_2_O_4_: C 59.56; H 7.85; N 9.92%. Found: C 59.86; H 7.96; N 10.06%.

#### 3.1.6. General procedure for the synthesis of compounds 8a,b

To a stirred solution of compound 7 (10 mmol) and triethylamine (10 mmol, 1.40 mL) in anhydrous benzene (50 mL) a solution of chloroacetyl chloride (10 mmol, 0.8 mL) was added dropwise at room temperature and the mixture was stirred for 1 h. After water (30 mL) was added and the mixture was extracted with benzene. The benzene layer was dried over MgSO_4_ and was distilled to dryness. Then water was added, the resulting crystals were filtered off, washed with water and recrystallized from methanol.

##### 3.1.6.1 7-(2-Chloroacetyl)-1,5-dimethyl-9-oxo-3,7-diazabicyclo[3.3.1]nonan-3-yl)-2-oxoacetate (8a)

Colorless solid, yield 64%, m.p. 173–174 °C. IR *ν*/cm^−1^: 1660, 1735, 1756 (C=O). ^1^H NMR (300 MHz, DMSO-*d*_6_/CCl_4_, 1/3) *δ* 1.00 (s, 3H, CH_3_), 1.04 (s, 3H, CH_3_), 2.78–2.97 (m, 2H), 3.21–3.42 (m, 2H), 3.84 and 3.88 (both s, 3H, OCH_3_), 3.93–4.30 (m, 3H), 4.40–4.90 (m, 3H). ^13^C NMR (75 MHz, DMSO-*d*_6_/CCl_4_, 1/3) *δ* 15.55, 15.78, 16.20, 41.33, 44.99, 51.87, 53.47, 53.70, 55.35, 55.99, 159.00, 162.43, 164.46, 209.61. Anal. calcd for C_14_H_19_ClN_2_O_5_: C 50.84; H 5.79; N 8.47%. Found: C 51.15; H 5.91; N 8.32%.

##### 3.1.6.2 7-(2-Chloroacetyl)-1,5-diethyl-9-oxo-3,7-diazabicyclo[3.3.1]nonan-3-yl)-2-oxoacetate (8b)

Colorless solid, yield 62%, m.p. 165–166 °C. IR *ν*/cm^−1^: 1668, 1715, 1740 (C=O). ^1^H NMR (300 MHz, DMSO-*d*_6_/CCl_4_, 1/3) *δ* 0.93 (br t, *J* = 7.4 Hz, 3H, CH_2_CH̲_3_), 0.96 (br t, *J* = 7.4 Hz, 3H, CH_2_CH̲_3_), 1.52 (br q, *J* = 7.4 Hz, 2H, CH̲_2_CH_3_), 1.58 (br q, *J* = 7.4 Hz, 2H, CH̲_2_CH_3_), 2.76–3.01 (m, 2H), 3.23–3.43 (m, 2H), 3.79–4.22 (m, 6H), 4.36–4.86 (m, 3H). Anal. calcd for C_16_H_23_ClN_2_O_5_: C 53.56; H 6.46; N 7.81%. Found: C 53.91; H 6.61; N 7.97%.

#### 3.1.7. General procedure for the synthesis of compounds 9a–p

In the to a stirred suspension of compound 5 (5 mmol) and potassium carbonate (1.38 g, 10 mmol) in absolute DMF (30 mL) the corresponding chloride of 3,7-diazabicyclo[3.3.1]nonane 8 (5.5 mmol) was added and the reaction mixture was maintained at 75–80 °C for 8 h. After cooling water was added, the resulting crystals were filtered off, washed with water, dried and recrystallized from the mixture of ethanol–chloroform (1 : 2).

##### 3.1.7.1 Methyl(7-{[(5-{[(4-cyano-7-isopropyl-1-pyrrolidin-1-yl-5,6,7,8-tetrahydro-2,7-naphthyridin-3-yl)oxy]methyl}-4-phenyl-4*H*-1,2,4-triazol-3-yl)thio]acetyl}-1,5-dimethyl-9-oxo-3,7-diazabicyclo[3.3.1]non-3-yl)(oxo)acetate (9a)

Cream solid, yield 74%, m.p. 267–268 °C. IR *ν*/cm^−1^: 1601, 1662, 1726 (C=O), 2200 (C≡N). ^1^H NMR (300 MHz, DMSO-*d*_6_) *δ* 0.91–1.01 (m, 6H, 2CH_3,_ bispidine), 1.03 (d, *J* = 6.4 Hz, 6H, CH(CH̲_3_)_2_), 1.79–1.90 (m, 4H, C_4_H_8_N), 2.59–3.06 (m, 7H, bispidine, 5,6-CH_2_-naph., CH̲(CH_3_)_2_), 3.22–3.62 (m, 8H, bispidine, N(CH_2_)_2_, 8-CH_2_), 3.69–4.82 (m, 6H, bispidine), 3.79 and 3.83 (both s, 3H, OCH_3_), 5.38 (s, 2H, OCH_2_), 7.38–7.57 (m, 5H, Ph). Anal. calcd for C_39_H_47_N_9_O_6_S: C 60.84; H 6.15; N 16.37%. Found: C 60.49; H 6.02; N 16.62%. ESI HRMS [C_39_H_47_N_9_O_6_S + H^+^] calculated: 770.3448. Found: 770.3480.

##### 3.1.7.2 Methyl(7-{[(5-{[(4-cyano-7-isopropyl-1-piperidin-1-yl-5,6,7,8-tetrahydro-2,7-naphthyridin-3-yl)oxy]methyl}-4-phenyl-4*H*-1,2,4-triazol-3-yl)thio]acetyl}-1,5-dimethyl-9-oxo-3,7-diazabicyclo[3.3.1]non-3-yl)(oxo)acetate (9b)

Cream solid, yield 71%, m.p. 231–233 °C. IR *ν*/cm^−1^: 1595, 1662, 1726 (C=O), 2205 (C≡N). ^1^H NMR (300 MHz, DMSO-*d*_6_) *δ* 0.90–1.03 (m, 6H, 2CH_3,_ bispidine), 1.03 (d, *J* = 6.3 Hz, 6H, CH(CH̲_3_)_2_), 1.47–1.67 (m, 6H, C_5_H_10_N), 2.63–3.08 (m, 7H, bispidine, 5,6-CH_2_-naph., CH̲(CH_3_)_2_), 3.10–3.47 (m, 8H, bispidine, N(CH_2_)_2_,8-CH_2_), 3.72 and 3.78 (both s, 3H, OCH_3_), 3.95–4.83 (m, 6H, bispidine), 5.42 (s, 2H, OCH_2_), 7.37–7.59 (m, 5H, Ph). ^13^C NMR (75 MHz, DMSO-*d*_6_) *δ* 15.6, 15.8, 16.2, 18.3, 23.9, 25.4, 28.8, 37.6, 44.6, 44.9, 45.1, 48.4, 49.3, 51.9, 52.7, 52.7, 53.1, 53.8, 55.4, 55.9, 56.9, 57.0, 83.9, 114.2, 115.1, 127.0, 127.1, 129.7, 130.0, 132.3, 151.1, 151.5, 151.7, 159.1, 159.5, 159.6, 163.0, 165.6, 210.9. Anal. calcd for C_40_H_49_N_9_O_6_S: C 61.28; H 6.30; N 16.08%. Found: C 60.91; H 6.45; N 16.31%. ESI HRMS [C_40_H_49_N_9_O_6_S + H^+^] calculated: 784.3605. Found: 784.3634.

##### 3.1.7.3 Methyl(7-{[(5-{[(1-azepan-1-yl-4-cyano-7-isopropyl-5,6,7,8-tetrahydro-2,7-naphthyridin-3-yl)oxy]methyl}-4-phenyl-4*H*-1,2,4-triazol-3-yl)thio]acetyl}-1,5-dimethyl-9-oxo-3,7-diazabicyclo[3.3.1]non-3-yl)(oxo)acetate (9c)

Cream solid, yield 77%, m.p. 230–232 °C. IR *ν*/cm^−1^: 1595, 1660, 1727 (C=O), 2207 (C≡N). ^1^H NMR (300 MHz, DMSO-*d*_6_) *δ* 0.95–1.05 (m, 6H, 2CH_3_, bispidine), 1.06 (d, *J* = 6.4 Hz, 6H, CH(CH̲_3_)_2_), 1.49–1.59 (m, 4H, C_6_H_12_N), 1.70–1.81 (m, 4H, C_6_H_12_N), 2.63–3.03 (m, 7H, bispidine, 5,6-CH_2_-naph., CH̲(CH_3_)_2_), 3.24–3.44 (m, 4H, bispidine, 8-CH_2_), 3.48–3.58 (m, 4H, N(CH_2_)_2_), 3.75 and 3.82 (both s, 3H, OCH_3_, bispidine), 4.00–4.88 (m, 6H, bispidine), 5.30 (s, 2H, OCH_2_), 7.42–7.57 (m, 5H, Ph). Anal. calcd for C_41_H_51_N_9_O_6_S: C 61.71; H 6.44; N 15.80%. Found: C 62.09; H 6.59; N 16.06%. ESI HRMS [C_41_H_51_N_9_O_6_S + H^+^] calculated: 798.3761. Found: 798.3804.

##### 3.1.7.4 Methyl(7-{[(5-{[(4-cyano-7-isopropyl-1-morpholin-4-yl-5,6,7,8-tetrahydro-2,7-naphthyridin-3-yl)oxy]methyl}-4-phenyl-4*H*-1,2,4-triazol-3-yl)thio]acetyl}-1,5-dimethyl-9-oxo-3,7-diazabicyclo[3.3.1]non-3-yl)(oxo)acetate (9d)

Cream solid, yield 73%, m.p. 244–246 °C. IR *ν*/cm^−1^: 1594, 1662, 1725 (C=O), 2205 (C≡N). ^1^H NMR (300 MHz, DMSO-*d*_6_) *δ* 0.90–1.02 (m, 6H, 2CH_3_, bispidine), 1.03 (d, *J* = 6.4 Hz, 6H, CH(CH̲_3_)_2_), 2.64–3.06 (m, 7H, bispidine, 5,6-CH_2_-naph., CH̲(CH_3_)_2_), 3.20–3.32 (m, 4H, N(CH_2_)_2_), 3.25–3.45 (m, 4H, bispidine, 8-CH_2_), 3.62–3.70 (m, 4H, O(CH_2_)_2_), 3.72 and 3.78 (both s, 3H, OCH_3_), 3.98–4.82 (m, 6H, bispidine), 5.44 (s, 2H, OCH_2_), 7.40–7.56 (m, 5H, Ph). ^13^C NMR (75 MHz, DMSO-*d*_6_) *δ* 15.6, 15.7, 16.2, 18.2, 28.8, 44.2, 44.9, 45.1, 48.3, 48.8, 52.7, 53.1, 53.8, 57.1, 57.2, 65.9, 84.8, 114.5, 114.8, 127.0, 127.0, 129.6, 129.7, 130.0, 132.3, 151.4, 151.6, 151.7, 158.4, 159.5, 159.6, 163.0, 165.6, 210.9. Anal. calcd for C_39_H_47_N_9_O_7_S: C 59.60; H 6.03; N 16.04%. Found: C 59.94; H 6.20; N 16.26%. ESI HRMS [C_39_H_47_N_9_O_7_S + H^+^] calculated: 786.3397. Found: 786.4131.

##### 3.1.7.5 Methyl(7-{[(5-{[(4-cyano-7-isopropyl-1-pyrrolidin-1-yl-5,6,7,8-tetrahydro-2,7-naphthyridin-3-yl)oxy]methyl}-4-phenyl-4*H*-1,2,4-triazol-3-yl)thio]acetyl}-1,5-diethyl-9-oxo-3,7-diazabicyclo[3.3.1]non-3-yl)(oxo)acetate (9e)

Cream solid, yield 70%, m.p. 228–230 °C. IR *ν*/cm^−1^: 1650, 1674, 1728 (C=O), 2198 (C≡N). ^1^H NMR (300 MHz, DMSO-*d*_6_) *δ* 0.77–0.93 (m, 6H, 2CH_3_, bispidine), 1.02 (d, *J* = 6.5 Hz, 6H, CH(CH̲_3_)_2_), 1.40–1.60 (m, 4H, 2CH̲_2_CH_3_, bispidine), 1.77–1.88 (m, 4H, C_4_H_8_N), 2.57–2.70 and 2.77–3.12 (both m, 7H, bispidine, 5,6-CH_2_-naph., CH̲(CH_3_)_2_), 3.22–3.59 (m, 8H, bispidine, 8-CH_2_, N(CH_2_)_2_), 3.68–4.74 (m, 6H, bispidine), 3.72 and 3.80 (both s, 3H, OCH_3_, bispidine), 5.38 (s, 2H, OCH_2_), 7.38–7.56 (m, 5H, Ph). Anal. calcd for C_41_H_51_N_9_O_6_S: C 61.71; H 6.44; N 15.80%. Found: C 61.38; H 6.62; N 16.01%. ESI HRMS [C_41_H_51_N_9_O_6_S + H^+^] calculated: 798.3761. Found: 798.3774.

##### 3.1.7.6 Methyl(7-{[(5-{[(4-cyano-7-isopropyl-1-piperidin-1-yl-5,6,7,8-tetrahydro-2,7-naphthyridin-3-yl)oxy]methyl}-4-phenyl-4*H*-1,2,4-triazol-3-yl)thio]acetyl}-1,5-diethyl-9-oxo-3,7-diazabicyclo[3.3.1]non-3-yl)(oxo)acetate (9f)

Cream solid, yield 74%, m.p. 212–214 °C. IR *ν*/cm^−1^: 1596, 1662, 1724 (C=O), 2209 (C≡N). ^1^H NMR (300 MHz, DMSO-*d*_6_) *δ* 0.88–1.04 (m, 6H, 2CH_3_, bispidine), 1.08 (d, *J* = 6.4 Hz, 6H, CH(CH̲_3_)_2_), 1.47–1.64 (m, 4H, 2CH̲_2_CH_3_, bispidine), 1.61–1.73 (m, 6H, C_5_H_10_N), 2.73 (t, *J* = 5.9 Hz, 2H, 6-CH_2_), 2.75–3.01 (m, 5H, bispidine, 5-CH_2_-naph., CH̲(CH_3_)_2_), 3.19–3.30 (m, 4H, N(CH_2_)_2_), 3.29–3.44 (m, 4H, bispidine, 8-CH_2_), 3.74–4.88 (m, 6H, bispidine), 3.77 and 3.86 (both s, 3H, OCH_3_, bispidine), 5.33 (s, 2H, OCH_2_), 7.44–7.59 (m, 5H, Ph). Anal. calcd for C_42_H_53_N_9_O_6_S: C 62.12; H 6.58; N 15.52%. Found: C 61.76; H 6.72; N 15.76%. ESI HRMS [C_42_H_53_N_9_O_6_S + H^+^] calculated: 812.3918. Found: 812.3953.

##### 3.1.7.7 Methyl(7-{[(5-{[(1-azepan-1-yl-4-cyano-7-isopropyl-5,6,7,8-tetrahydro-2,7-naphthyridin-3-yl)oxy]methyl}-4-phenyl-4*H*-1,2,4-triazol-3-yl)thio]acetyl}-1,5-diethyl-9-oxo-3,7-diazabicyclo[3.3.1]non-3-yl)(oxo)acetate (9g)

Cream solid, yield 72%, m.p. 197–199 °C. IR *ν*/cm^−1^: 1597, 1663, 1723 (C=O), 2207 (C≡N). ^1^H NMR (300 MHz, DMSO-*d*_6_) *δ* 0.78–0.93 (m, 6H, 2CH_3_, bispidine), 1.02 (d, *J* = 6.5 Hz, 6H, CH(CH̲_3_)_2_), 1.40–1.57 (m, 8H, 2CH̲_2_CH_3_-adam, C_6_H_12_N), 1.62–1.76 (m, 4H, C_6_H_12_N), 2.60–3.12 (m, 7H, bispidine, 5,6-CH_2_-naph., CH̲(CH_3_)_2_), 3.12–3.45 (m, 4H, bispidine, 8-CH_2_), 3.45–3.55 (m, 4H, N(CH_2_)_2_), 3.70–4.75 (m, 6H, adam), 3.72 and 3.79 (both s, 3H, OCH_3_, bispidine), 5.37 (s, 2H, OCH_2_), 7.39–7.56 (m, 5H, Ph). ^13^C NMR (75 MHz, DMSO-*d*_6_) *δ* 7.4, 7.8, 18.3, 22.9, 23.3, 26.3, 27.1, 29.2, 37.4, 44.2, 47.3, 47.5, 47.6, 49.3, 49.9, 50.7, 52.1, 52.8, 53.3, 54.1, 54.5, 56.9, 56.9, 81.5, 111.4, 115.5, 127.0, 129.7, 130.0, 132.3, 150.7, 151.5, 158.5, 159.2, 163.0, 166.0, 210.7. Anal. calcd for C_43_H_55_N_9_O_6_S: C 62.52; H 6.71; N 15.26%. Found: C 62.89; H 6.89; N 15.49%. ESI HRMS [C_43_H_55_N_9_O_6_S + H^+^] calculated: 826.4074. Found: 826.4117.

##### 3.1.7.8 Methyl(7-{[(5-{[(4-cyano-7-isopropyl-1-morpholin-4-yl-5,6,7,8-tetrahydro-2,7-naphthyridin-3-yl)oxy]methyl}-4-phenyl-4*H*-1,2,4-triazol-3-yl)thio]acetyl}-1,5-diethyl-9-oxo-3,7-diazabicyclo[3.3.1]non-3-yl)(oxo)acetate (9h)

Cream solid, yield 71%, m.p. 229–230 °C. IR *ν*/cm^−1^: 1596, 1663, 1724 (C=O), 2208 (C≡N). ^1^H NMR (300 MHz, DMSO-*d*_6_) *δ* 0.78–0.94 (m, 6H, 2CH_3_, bispidine), 1.03 (d, *J* = 6.5 Hz, 6H, CH(CH̲_3_)_2_), 1.38–1.60 (m, 4H, 2CH̲_2_CH_3_, adam), 2.62–3.13 (m, 7H, bispidine, 5,6-CH_2_-naph., CH̲(CH_3_)_2_), 3.20–3.51 (m, 8H, bispidine, N(CH_2_)_2_, 8-CH_2_), 3.62–3.69 (m, 4H, O(CH_2_)_2_), 3.68–4.85 (m, 6H, bispidine), 3.72 and 3.80 (both s, 3H, OCH_3_), 5.44 (s, 2H, OCH_2_), 7.40–7.58 (m, 5H, Ph). Anal.calcd for C_41_H_51_N_9_O_7_S: C 60.50; H 6.32; N 15.49%. Found: C 60.85; H 6.49; N 15.71%. ESI HRMS [C_41_H_51_N_9_O_7_S + H^+^] calculated: 814.3710. Found: 814.3749.

##### 3.1.7.9 Methyl(7-{[(5-{[(7-benzyl-4-cyano-1-pyrrolidin-1-yl-5,6,7,8-tetrahydro-2,7-naphthyridin-3-yl)oxy]methyl}-4-phenyl-4*H*-1,2,4-triazol-3-yl)thio]acetyl}-1,5-dimethyl-9-oxo-3,7-diazabicyclo[3.3.1]non-3-yl)(oxo)acetate (9i)

Light yellowsolid, yield 75%, m.p. 218–220 °C. IR *ν*/cm^−1^: 1598, 1659, 1725 (C=O), 2203 (C≡N). ^1^H NMR (300 MHz, DMSO-*d*_6_) *δ* 0.92–1.16 (m, 6H, 2CH_3_, bispidine), 1.79–1.93 (m, 4H, C_4_H_8_N), 2.60–3.04 (m, 7H, bispidine, 5,6-CH_2_-naph., CH̲(CH_3_)_2_), 3.22–3.58 (m, 8H, bispidine, N(CH_2_)_2_, 8-CH_2_), 3.67 (s, 2H, CH̲_2_Ph), 3.75 and 3.81 (both s, 3H, OCH_3_), 4.00–4.89 (m, 5H, bispidine), 5.32 (s, 2H, OCH_2_), 7.20–7.35 (m, 5H, CH_2_**Ph**), 7.39–7.59 (m, 5H, Ph). Anal. calcd for C_43_H_47_N_9_O_6_S: C 63.14; H 5.79; N 15.41%. Found: C 62.76; H 5.95; N 15.66%. ESI HRMS [C_43_H_47_N_9_O_6_S + H^+^] calculated: 818.3448. Found: 818.3494.

##### 3.1.7.10 Methyl(7-{[(5-{[(7-benzyl-4-cyano-1-piperidin-1-yl-5,6,7,8-tetrahydro-2,7-naphthyridin-3-yl)oxy]methyl}-4-phenyl-4*H*-1,2,4-triazol-3-yl)thio]acetyl}-1,5-dimethyl-9-oxo-3,7-diazabicyclo[3.3.1]non-3-yl)(oxo)acetate (9j)

Cream solid, yield 73%, m.p. 252–254 °C. IR *ν*/cm^−1^: 1595, 1661, 1726 (C=O), 2208 (C≡N). ^1^H NMR (300 MHz, DMSO-*d*_6_) *δ* 0.95–1.11 (m, 6H, 2CH_3_, bispidine), 1.50–1.67 (m, 6H, C_5_H_10_N), 2.70 (t, *J* = 5.6 Hz, 2H, 6-CH_2_), 2.77–3.00 (m, 4H, bispidine, 5-CH_2_-naph.), 3.14–3.25 (m, 4H, N(CH_2_)_2_), 3.26 (s, 2H, 8-CH_2_), 3.27–3.43 (m, 2H, bispidine), 3.65 (s, 2H, CH̲_2_Ph), 3.77 and 3.84 (both s, 3H, OCH_3_), 4.00–4.94 (m, 6H, bispidine), 5.33 (s, 2H, OCH_2_), 7.18–7.33 (m, 5H, CH_2_**Ph**), 7.43–7.59 (m, 5H, Ph). Anal. calcd for C_44_H_49_N_9_O_6_S: C 63.52; H 5.94; N 15.15%. Found: C 63.88; H 6.12; N 15.39%. ESI HRMS [C_44_H_49_N_9_O_6_S + H^+^] calculated: 832.3605. Found: 832.3641.

##### 3.1.7.11 Methyl(7-{[(5-{[(1-azepan-1-yl-7-benzyl-4-cyano-5,6,7,8-tetrahydro-2,7-naphthyridin-3-yl)oxy]methyl}-4-phenyl-4*H*-1,2,4-triazol-3-yl)thio]acetyl}-1,5-dimethyl-9-oxo-3,7-diazabicyclo[3.3.1]non-3-yl)(oxo)acetate (9k)

Yellow solid, yield 76%, m.p. 196–198 °C. IR *ν*/cm^−1^: 1595, 1658, 1725 (C=O), 2207 (C≡N). ^1^H NMR (300 MHz, DMSO-*d*_6_) *δ* 0.90–1.08 (m, 6H, 2CH_3_, bispidine), 0.41–1.54 (m, 4H, C_6_H_12_N), 1.60–1.73 (m, 4H, C_6_H_12_N), 2.68 (t, *J* = 5.7 Hz, 2H, 6-CH_2_), 2.75–2.88 (m, 4H, bispidine, 5-CH_2_-naph.), 3.01–3.24 (m, 4H, N(CH_2_)_2_), 3.29 (s, 2H, 8-CH_2_), 3.42–3.53 (m, 2H, bispidine), 3.64 (s, 2H, CH̲_2_Ph), 3.77 and 3.85 (both s, 3H, OCH_3_), 4.05–4.95 (m, 6H, bispidine), 5.28 (s, 2H, OCH_2_), 7.17–7.32 (m, 5H, CH_2_**Ph**), 7.35–7.58 (m, 5H, Ph). Anal. calcd for C_45_H_51_N_9_O_6_S: C 63.89; H 6.08; N 14.90%. Found: C 63.55; H 6.23; N 15.16%. ESI HRMS [C_45_H_51_N_9_O_6_S + H^+^] calculated: 846.3761. Found: 846.3820.

##### 3.1.7.12 Methyl(7-{[(5-{[(7-benzyl-4-cyano-1-morpholin-4-yl-5,6,7,8-tetrahydro-2,7-naphthyridin-3-yl)oxy]methyl}-4-phenyl-4*H*-1,2,4-triazol-3-yl)thio]acetyl}-1,5-dimethyl-9-oxo-3,7-diazabicyclo[3.3.1]non-3-yl)(oxo)acetate (9l)

Yellow solid, yield 71%, m.p. 234–236 °C. IR *ν*/cm^−1^: 1593, 1659, 1725 (C=O), 2205 (C≡N). ^1^H NMR (300 MHz, DMSO-*d*_6_) *δ* 0.91–1.01 (m, 6H, 2CH_3_, bispidine), 2.68 (t, *J* = 5.5 Hz, 2H, 6-CH_2_), 2.77–3.06 (m, 4H, bispidine, 5-CH_2_-naph.), 3.16–3.22 (m, 4H, N(CH_2_)_2_), 3.25 (s, 2H, 8-CH_2_), 3.33–3.45 (m, 2H, bispidine), 3.54–3.60 (m, 4H, O(CH_2_)_2_), 3.64 (s, 2H, CH̲_2_Ph), 3.71 and 3.78 (both s, 3H, OCH_3_), 3.99–4.81 (m, 6H, bispidine), 5.44 (s, 2H, OCH_2_), 7.24–7.38 (m, 5H, Ph), 7.39–7.55 (m, 5H, Ph). Anal. calcd for C_43_H_47_N_9_O_7_S: C 61.93; H 5.68; N 15.12%. Found: C 61.56; H 5.49; N 14.91%. ESI HRMS [C_43_H_47_N_9_O_7_S + H^+^] calculated: 834.3397. Found: 834.3444.

##### 3.1.7.13 Methyl(7-{[(5-{[(7-benzyl-4-cyano-1-pyrrolidin-1-yl-5,6,7,8-tetrahydro-2,7-naphthyridin-3-yl)oxy]methyl}-4-phenyl-4*H*-1,2,4-triazol-3-yl)thio]acetyl}-1,5-diethyl-9-oxo-3,7-diazabicyclo[3.3.1]non-3-yl)(oxo)acetate (9m)

Light yellow solid, yield 72%, m.p. 183–185 °C. IR *ν*/cm^−1^: 1598, 1652, 1726 (C=O), 2204 (C≡N). ^1^H NMR (300 MHz, DMSO-*d*_6_) *δ* 0.84–1.02 (m, 6H, 2CH_3_, bispidine), 1.43–1.62 (m, 4H, 2CH̲_2_CH_3_, bispidine), 1.81–1.93 (m, 4H, C_4_H_8_N), 2.64 (t, *J* = 5.6 Hz, 2H, 6-CH_2_), 2.71–3.17 (m, 4H, bispidine, 5-CH_2_-naph.), 3.26–3.57 (m, 8H, bispidine, N(CH_2_)_2_, 8-CH_2_), 3.64 (s, 2H, CH̲_2_Ph), 3.77 and 3.85 (both s, 3H, OCH_3_), 3.89–4.90 (m, 6H, bispidine), 5.29 (s, 2H, OCH_2_), 7.16–7.32 (m, 5H, CH_2_**Ph**), 7.41–7.58 (m, 5H, Ph). Anal. calcd for C_45_H_51_N_9_O_6_S: C 63.89; H 6.08; N 14.90%. Found: C 63.54; H 6.25; N 15.13%. ESI HRMS [C_45_H_51_N_9_O_6_S + H^+^] calculated: 846.3761. Found: 846.3770.

##### 3.1.7.14 Methyl(7-{[(5-{[(7-benzyl-4-cyano-1-piperidin-1-yl-5,6,7,8-tetrahydro-2,7-naphthyridin-3-yl)oxy]methyl}-4-phenyl-4*H*-1,2,4-triazol-3-yl)thio]acetyl}-1,5-diethyl-9-oxo-3,7-diazabicyclo[3.3.1]non-3-yl)(oxo)acetate (9n)

Yellow solid, yield 74%, m.p. 167–169 °C. IR *ν*/cm^−1^: 1593, 1661, 1725 (C=O), 2206 (C≡N). ^1^H NMR (300 MHz, DMSO-*d*_6_) *δ* 1.03–1.20 (m, 6H, 2CH_3_, bispidine), 1.63–1.89 (m, 10H, 2CH̲_2_CH_3_, bispidine, C_5_H_10_N), 2.87 (t, *J* = 5.6 Hz, 2H, 6-CH_2_), 2.94–3.20 (m, 4H, bispidine, 5-CH_2_-naph.), 3.30–3.62 (m, 8H, bispidine, N(CH_2_)_2_, 8-CH_2_), 3.82 (s, 2H, CH̲_2_Ph), 3.94 and 4.03 (both s, 3H, OCH_3_), 4.04–5.06 (m, 6H, bispidine), 5.51 (s, 2H, OCH_2_), 7.35–7.51 (m, 5H, CH_2_**Ph**), 7.60–7.77 (m, 5H, Ph). Anal. calcd for C_46_H_53_N_9_O_6_S: C 64.24; H 6.21; N 14.66%. Found: C 63.86; H 6.39; N 14.91%. ESI HRMS [C_46_H_53_N_9_O_6_S + H^+^] calculated: 860.3918. Found: 860.3979.

##### 3.1.7.15 Methyl(7-{[(5-{[(1-azepan-1-yl-7-benzyl-4-cyano-5,6,7,8-tetrahydro-2,7-naphthyridin-3-yl)oxy]methyl}-4-phenyl-4*H*-1,2,4-triazol-3-yl)thio]acetyl}-1,5-diethyl-9-oxo-3,7-diazabicyclo[3.3.1]non-3-yl)(oxo)acetate (9o)

Light yellow solid, yield 77%, m.p. 151–153 °C. IR *ν*/cm^−1^: 1595, 1660, 1725 (C=O), 2209 (C≡N). ^1^H NMR (400 MHz, CDCl_3_) *δ* 0.83–1.09 (m, 6H, 2CH_3_, bispidine), 1.36–1.79 (m, 12H, 2CH̲_2_CH_3_, bispidine, C_6_H_12_N), 2.71–3.11 (m, 6H, bispidine, 5,6-CH_2_-naph.), 3.20–3.53 (m, 8H, N(CH_2_)_2_), bispidine, 8-CH_2_),3.70 (s, 2H, CH̲_2_Ph), 3.82 and 3.90 (both s, 3H, OCH_3_), 4.03–5.03 (m, 6H, bispidine), 5.32 (s, 2H, OCH_2_), 7.23–7.56 (m, 10H, Ph). Anal. calcd for C_47_H_55_N_9_O_6_S: C 64.58; H 6.34; N 14.42%. Found: C 64.22; H 6.19; N 14.20%. ESI HRMS [C_47_H_55_N_9_O_6_S + H^+^] calculated: 874.4074. Found: 874.4129.

##### 3.1.7.16 Methyl(7-{[(5-{[(7-benzyl-4-cyano-1-morpholin-4-yl-5,6,7,8-tetrahydro-2,7-naphthyridin-3-yl)oxy]methyl}-4-phenyl-4*H*-1,2,4-triazol-3-yl)thio]acetyl}-1,5-diethyl-9-oxo-3,7-diazabicyclo[3.3.1]non-3-yl)(oxo)acetate (9p)

Light yellow solid, yield 75%, m.p. 172–174 °C. IR *ν*/cm^−1^: 1593, 1661, 1737 (C=O), 2215 (C≡N). ^1^H NMR (300 MHz, DMSO-*d*_6_) *δ* 0.85–1.03 (m, 6H, 2CH_3_, bispidine), 1.46–1.65 (m, 4H, 2CH̲_2_CH_3_, bispidine), 2.66–3.01 (m, 6H, bispidine, 5,6-CH_2_-naph.), 3.20–3.28 (m, 4H, N(CH_2_)_2_), 3.28–3.44 (m, 4H, bispidine, 8-CH_2_), 3.58–3.65 (m, 4H, O(CH_2_)_2_), 3.68 (s, 2H, CH̲_2_Ph), 3.77 and 3.85 (both s, 3H, OCH_3_), 3.89–4.88 (m, 6H, bispidine), 5.36 (s, 2H, OCH_2_), 7.19–7.33 (m, 5H, CH_2_**Ph**), 7.43–7.59 (m, 5H, Ph). Anal. calcd for C_45_H_51_N_9_O_7_S: C 62.70; H 5.96; N 14.62%. Found: C 63.03; H 6.13; N 14.86%. ESI HRMS [C_45_H_51_N_9_O_7_S + H^+^] calculated: 862.3710. Found: 862.3751.

### 3.2. Biological evaluation

#### 3.2.1. Evaluation of the anticonvulsant activity of the synthesized compounds

Compounds were studied for their potential neurotropic activities (anticonvulsant, sedative, and anti-anxiety effects) as well as side effects in 250 outbred mice of both sexes (weighing 18–22 g) and 100 male Wistar rats (weighing 120–140 g). All groups of animals were maintained at 22 ± 2 °C in the same room and fed a common diet. The anticonvulsant effect of the new synthesized compounds was investigated by tests: PTZ (Acros organics, New Jersey, USA), convulsions and TSC seizures.^74–78^

PTZ test is an experimental model for inducing myoclonic seizures, as well as for predicting the anxiolytic properties of compounds. PTZ test was carried out in mice by subcutaneous administration of analeptic at a dose of 85 mg kg^−1^ and the effectiveness of the preparations was determined by the prevention of clonic seizures.

Substances were administered intraperitoneally in doses of 10, 25, 50, 75, 100 mg kg^−1^ in suspension with carboxymethylcellulose (“Viadi – Ingredients”) with tween-80 (“Ferak Berlin”) 45 minutes before the injection of the convulsive agent pentilentetrazole causing electrical irritation. The control animals were administered an emulsifier. Each dose of compounds for each test was studied in 8 animals. Analogues of comparison were an anticonvulsant drug from the group of succinimide ethosuximide (neuraxpharm Arzneimittel GmbH (Germany) and tranquilizer diazepam.^79^ Comparison drugs, ethosuximide in doses from 100 to 300 mg kg^−1^, diazepam in doses 2 mg kg^−1^ was administered intraperitoneally.

To determine the 50% effective (ED_50_, causing the anticonvulsant effect of 50% of animals, is calculated by the test antagonizm to PTZ), 50% neurotoxic (TD_50_, causing myorelaxation in 50% of animals) doses a statistical method of probit analysis by Litchfield and Wilcoxon was used.^81,82^ In addition to the above method, we always use a statistical method called the *χ*^2^ test.

#### 3.2.2. Evaluation of the psychotropic properties of the synthesized compounds

Psychotropic properties of compounds were studied by tests: open field, EPM, FST.

Open field test.^83–86^ The research-motor behavior of rats was studied on a modified open field model. For this purpose, an installation was used, the bottom of which is divided into squares with holes (cells). Experiments were performed in the daytime with natural light. Within 5 minutes of the experiment, the indicators of sedative and activating behavior were determined – the number of horizontal movements, standing on the hind legs (vertical movements), sniffing of the cells. The number of animals on this model was 8 for each compound, control, and reference drug. The studied compounds were administered to rats in the most effective dose of 50 mg kg^−1^ intraperitoneally as a suspension with methylcarboxycellulose with Tween-80.

EPM test.^87–89^ Anti-anxiety and sedative effects were studied on a model of the “elevated plus maze” in mice. The labyrinth is a cruciform machine raised above the floor, having a pair of open and closed sleeves opposed to each other. Normal animals prefer to spend most of their time in the closed (dark) sleeves of the labyrinth. The anxiolytic effect of the compounds was estimated by the increase in the number of entries into open (light) sleeves and the time spent in, without increasing the total motor activity. This record, the time spent in the closed sleeve, the number of attempts to enter the installation center. In the above model, the test compounds and the reference drug were injected intraperitoneally before the experiments. The control animals were administered an emulsifier. Results were processed statistically (*P* ≤ 0.05).

Forced swimming test.^90,91^ To assess “despair and depression” the model “compelling swimming” was used. Experimental animals were forced to swim in a glass container (height 22 cm, diameter 14 cm), filled 1/3 with water. Intact mice swim very actively, but soon they will be forced to immobilize. The latent period of immobilization, the total duration of active swimming, immobilization is fixed for 6 minutes. The experiments were conducted under natural light.

Tail suspension test.^92–94^ This test is a mouse behavioral test useful in the screening of potential antidepressant drugs, and assessing of other manipulations that are expected to affect depression related behaviors. Mice are suspended by their tails with tape, in such a position that it cannot escape or hold on to nearby surfaces. During this test, typically six minutes in duration, the resulting escape-oriented behaviors are quantified. The tail-suspension test is a valuable tool in drug discovery for high-throughput screening of prospective antidepressant compounds.

#### 3.2.3. Evaluation of coordination of movements in the rotating rod test

Adverse neurotoxic (muscle relaxant) effect of compounds was studied in doses of 50 to 100 mg kg^−1^ when administered intraperitoneally, as well as reference drugs in effective anticonvulsant doses. Miorelaxation was investigated by the test of a rotating rod in mice.^74,80^ To this end, mice were planted on a metal rod with a corrugated rubber coating, which rotated at a speed of 5 rpm. The number of animals that cannot stay on it for 2 minutes was determined. Maximal tolerated dose (MTD) is also studied. The compounds by i.p. injection in doses from 500–1800 mg kg^−1^ were investigated.

### 3.3. Docking studies

Docking studies were performed using AutoDock 4 (ver. 4.2.6) into the 3D structures of GABAA receptor (PDB code: 4COF), SERT transporter (PDB code: 5I71) and β_2_-adrenergic receptor (PDB code: 3NYA), retrieved from Protein Data Bank (PDB). For the finally preparation of both ligands and protein preparation Wizard of AutoDock tools 1.5.6 are used. As reported in previous paper.^52^

## 4 Conclusions

The synthesis of novel 1,2,4-triazole-linked hybrids based on 2,7-naphthyridine and bispidine (3,7-diazabicyclo[3.3.1]nonane) cores was carried out successfully. The target compounds were obtained in good yields ranging from 70% to 77%. All synthesized hybrids were evaluated for their neurotropic activity, specifically anticonvulsant, anxiolytic, and antidepressant effects, using the following *in vivo* models: PTZ test, open field test, EPM, FST, and tail suspension test. In the TSC-induced seizure model, the most active compounds were identified as 9c, 9h, 9k, 9d, and 9g, with 9c and 9k – both containing an azepane moiety – exhibiting the highest anticonvulsant activity, with ED_50_ values of 15.0 mg kg^−1^ and 18.5 mg kg^−1^, respectively.

In open field test, compounds 9c, 9g and 9k demonstrated significant activity, with 9c again emerging as the most effective. Overall, compound 9c showed the highest activity across all behavioral models, suggesting a broad neurotropic profile.

A molecular docking study was performed for all compounds targeting the GABAA receptor, the serotonin transporter (SERT), and the 5-HT1A receptor. The best binding affinities to the GABAA receptor were observed for compounds 9c and 9k, with free binding energies of −9.89 kcal mol^−1^ and −10.85 kcal mol^−1^, respectively—both surpassing that of the reference drug diazepam (−8.90 kcal mol^−1^), aligning well with the experimental findings. For the SERT transporter, compounds 9c and 9g showed the most favorable binding. Regarding the 5-HT1A receptor, compounds 9c (−9.92 kcal mol^−1^) and 9k (−12.10 kcal mol^−1^) exhibited strong binding affinities, comparable to the reference compound.

Based on these results, it can be concluded that neurotropic activity in this series is influenced by the nature of the substituents on the 2,7-naphthyridine core. In particular, the presence of isopropyl and azepane fragments appears to enhance biological activity.

## Ethical statement

The study reports that animal experiments were conducted in accordance with international guidelines and approved by the Ethics Committee of the Yerevan State Medical University (protocol code 5, 18.03.2024).

## Author contributions

Conceptualization, S. N. S., A. G. and R. G. P.; methodology, S. N. S., R. G. P.; software, A. P.; validation, A. A. H., T. A. A. and M. V. G.; formal analysis, H. A. Y., K. A. G., A. D. H.; investigation, A. A. H., E. K. H., T. A. A.; resources, S. N. S.; data curation, H. V. J.; writing—original draft preparation, S. N. S, A. G., R. G. P.; writing—review and editing, A. G., V. G. K.; visualization, E. K. H.; supervision, S. N. S.; project administration, S. N. S., R. G. P.; funding acquisition, S. N. S. All authors have read and agreed to the published version of the manuscript.

## Conflicts of interest

The authors declare no conflict of interest.

## Supplementary Material

RA-016-D6RA00302H-s001

RA-016-D6RA00302H-s002

## Data Availability

The data supporting this article have been included as part of the supplementary information (SI). Supplementary information is available. See DOI: https://doi.org/10.1039/d6ra00302h.

## References

[cit1] Majkowski, Antiepileptic Drugs: Combination Therapy and Interactions, ed. J. Majkowski, B. F. D. Bourgeois, P. N. Patsalos and R. H. Mattson, Cambridge University Press, New York, NY, USA, 2005, pp. 54–60

[cit2] Genton P. (2005). Acta Neurol. Scand. Suppl..

[cit3] Waris A., Siraj M., Khan A., Lin J., Asim M., Alhumaydh F. A. (2024). ACS Pharmacol. Transl. Sci..

[cit4] Peyton L. R., Gallagher S., Hashemzadeh M. (2015). Drugs Today.

[cit5] Zhou C.-H., Wang Y. (2012). Curr. Med. Chem..

[cit6] Saffour S., AL-Sharabi A. A., Evren A. E., Cankiliç M. Y., Yurttas L. (2024). J. Mol. Struct..

[cit7] Sadeghian S., Emami L., Mojaddami A., Khabnadideh S., Faghih Z., Zomorodian K., Rashidi M., Rezaei Z. (2023). J. Mol. Struct..

[cit8] Pachuta-Stec A. (2022). Mini Rev. Med. Chem..

[cit9] Kumari M., Tahlan S., Narasimhan B., Ramasamy K., Lim S. M., Shah S. A., Mani V., Kakkar S. (2021). BMC Chem..

[cit10] Tapera M., Kekeçmuhammed H., Tüzün B., Dastan S. D., Çelik M. S., Taslimi P., Dastan T., Topcu K. S. B., Cacan E., Sahin O., Sarıpınar E. (2024). J. Mol. Struct..

[cit11] Simurova N. V., Maiboroda O. I. (2021). Chem. Heterocycl. Compd..

[cit12] Borysenko N. M., Hubenko I. Y., Parchenko V. V., Bushuieva I. V., Demchenko A. V., Sukhovyi G. P. (2025). World J. Pharm. Sci. Res..

[cit13] Hu L., Li M., Liu Z., Yan H., Wang X., Wang S. (2025). Molecules.

[cit14] Jahani R., Abtahi S. R., Nematpour M., Dastjerdi H. F., Chamanara M., Hami Z., Paknejad B. (2020). Bioorg. Chem..

[cit15] Kapron B., Czarnomysy R., Wysokinski M., Andrys R., Musilek K., Angeli A., Supuran C. T., Plech T. (2020). J. Enzym. Inhib. Med. Chem..

[cit16] Pal R., Kumar B., Swamy G. P. M., Chawla P. A. (2023). Bioorg. Chem..

[cit17] El-Saghier A. M., Mohamed M. A., Abd-Allah O. A., Kadry A. M., Ibrahim T. M., Bekhit A. A. (2019). Chem. Res..

[cit18] Liao L., Jiang C., Chen J., Shi J., Li X., Wang Y., Wen J., Zhou S., Liang J., Lao Y., Zhang J. (2020). Eur. J. Med. Chem..

[cit19] Opsomer T. and Dehaen W., Comprehensive Heterocyclic Chemistry IV, 2022, vol. 5, pp. 78–121

[cit20] Rodrigues S. C., Moratório de Moraes R. S., Almeida Pinto G. T., Martins M. T., Nascimento P. A., Soares D. L., Botelho A. B., Cruz C. C., Cunha A. S. (2025). Chem. Rec..

[cit21] Abdelli A., Azzouni S., Plais R., Gaucher A., Efrit M. L., Prim D. (2021). Tetrahedron Lett..

[cit22] Aggarwal R., Sumran G. (2020). Eur. J. Med. Chem..

[cit23] Strzelecka M., Świątek P. (2021). Pharmaceuticals.

[cit24] Matin M. M., Matin P., Rahman M. R., Ben Hadda T., Almalki F. A., Mahmud S., Ghoneim M. M., Alruwaily M., Alshehri S. (2022). Front. Mol. Biosci..

[cit25] Aly A. A., Hassan A. A., Makhlouf M. M., Bräse S. (2020). Molecules.

[cit26] Legru A., Verdirosa F., Vo-Hoang Y., Tassone G., Vascon F., Thomas C. A., Sannio F., Corsica G., Benvenuti M., Feller G., Coulon R., Marcoccia F., Devente S. R., Bouajila E., Piveteau C., Leroux F., Deprez-Poulain R., Deprez B., Licznar-Fajardo P., Crowder M. W., Cendron L., Pozzi C., Mangani S., Docquier J.-D., Hernandez J.-F., Gavara L. (2022). J. Med. Chem..

[cit27] Bersani M., Failla M., Vascon F., Gianquinto E., Bertarini L., Baroni M., Cruciani G., Verdirosa F., Sannio F., Docquier J.-D., Cendron L., Spyrakis F., Lazzarato L., Tondi D. (2023). Pharmaceuticals.

[cit28] Karpun Y., Parchenko V., Fotina T., Demianenko D., Fotin A., Nahornyi V., Nahorna N. (2021). Pharmacia.

[cit29] Rouzi K., Brandan S. A., El Houssni I., Poyraz E. B., El-Hassani I. A., Dege N., Abuelizz H. A., Oulmidi A., Bouatia M., Karrouch K. (2025). J. Mol. Struct..

[cit30] Zaheer M., Zia-Ur-Rehman M., Munir R., Jamil N., Ishtiaq S., Zaib Saleem R. S., Elsegood M. R. J. (2021). ACS Omega.

[cit31] Abdellatif K. R. A., Abdelall E. K. A., Elshemy H. A. H., Philoppes J. N., Hassanein E. H. M., Kahk N. M. (2021). Bioorg. Chem..

[cit32] Fadaly W. A. A., Nemr M. T. M., Zidan T. H., Mohamed F. E. A., Abdelhakeem M. M., Abu Jayab N. N., Omar H. A., Abdellatif K. R. A. (2023). J. Enzyme Inhib. Med. Chem..

[cit33] Koçak Aslan E., Sezer A., Küçükkılınç T. T., Palaska E. (2024). Arch. Pharm..

[cit34] Al-Humaidi J. Y., Shaaban M. M., Rezki N., Aouad M. R., Zakaria M., Jaremko M., Hagar M., Elwakil B. H. (2022). Life.

[cit35] Kumar A., Arya P., Giovannuzzi S., Mohan B., Raghav N., Supuran C. T., Sharma P. K. (2024). Future Med. Chem..

[cit36] Hu Y., Liu Z., Zha G., Long S., Sridhara M. B., Kumar K. S. S., Rakesh K. P. (2023). Process Biochem..

[cit37] Ziyaev A. A., Sasmakov S. A., Toshmurodov T. T., Abdurakhmanov J. M., Ikramov S. A., Khasanov S. S., Ashirov O. N., Ziyaeva M. A., Begimqulova D. B. (2025). Organics.

[cit38] Kazeminejad Z., Marzi M., Shiroudi A., Kouhpayeh S. A., Farjam M., Zarenezhad E. (2022). Biomed Res. Int..

[cit39] Šermukšnytė A., Kantminienė K., Jonuškienė I., Tumosienė I., Petrikaitė V. (2022). Pharmaceuticals.

[cit40] Almasmoum H. A., Almaimani G., Almaimani R. A., Babakr A. T., Alsunbul M., Alshwyeh H. A., Zayed E. S., Ibrahim I. A. A., Saied E. M. (2025). RSC Adv..

[cit41] Verma K. K., Singh U. K., Jain J. (2020). Cent. Nerv. Syst. Agents Med. Chem..

[cit42] Drabik M., Głuszak M., Wróblewska-Łuczka P., Plewa Z., Jankiewicz M., Kozińska J., Florek-Łuszczki M., Plech T., Łuszczki J. J. (2021). Neurochem. Res..

[cit43] Karaküçük-İyidoğan A., Başaran E., Tatar-Yılmaz G., Oruç-Emre E. E. (2024). Bioorg. Chem..

[cit44] Kapron B., Luszczki J. J., Siwek A., Karcz T., Nowak G., Zagaja M., Andres-Mach M., Stasiłowicz A., Cielecka-Piontek J., Kocki J., Plech T. (2020). Bioorg. Chem..

[cit45] Hu L., Li M., Liu Z., Yan H., Wang X., Wang S. (2025). Molecules.

[cit46] Wang Y., Quan Z., Liu D. (2023). Lett. Drug Des. Discovery.

[cit47] Sirakanyan S. N., Tonoyants N. A., Noravyan A. S., Dzhagatspanyan I. A., Nazaryan I. M., Akopyan A. G., Paronikyan R. G. (2014). Pharm. Chem. J..

[cit48] Sirakanyan S. N., Avetisyan N. G., Noravyan A. S. (2012). Chem. Heterocycl. Compd..

[cit49] Wójcicka A. (2021). Curr. Org. Chem..

[cit50] Guo C., Guzzo P. R., Hadden M., Sargent B. J., Yet L., Kan Y., Palyha O., Kelly T. M., Guan X., Rosko K., Gagen K., Metzger J. M., Dragovic J., Lyons K., Lin L. S., Nargund R. P. (2010). Bioorg. Med. Chem. Lett..

[cit51] Ukita T., Nakamura Y., Kubo A., Yamamoto Y., Moritani Y., Saruta K., Higashijima T., Kotera J., Fujishige K., Takagi M., Kikkawa K., Omori K. (2003). Bioorg. Med. Chem. Lett..

[cit52] Sirakanyan S. N., Hakobyan E. K., Geronikaki A., Spinelli D., Petrou A., Kartsev V. G., Yegoryan H. A., Jughetsyan H. J., Manukyan M. E., Paronikyan R. G., Araqelyan T., Hovakimyan A. A. (2025). Pharmaceuticals.

[cit53] Sacchetti A., Rossetti A. (2021). Eur. J. Org Chem..

[cit54] Krut’ko D. P., Medved’ko A. V., Lyssenko K. A., Churakov A. V., Dalinger A. I., Kalinin M. A., Gudovannyy A. O., Ponomarev K. Y., Suslov E. V., Vatsadze S. Z. (2022). Molecules.

[cit55] Cieslik P. A., Roth D., Nisli E., Genz J., Berton C., Grundler P. V., Hillhouse C. C., Moiseeva A. N., Nolff M., Braband H., van der Meulen N. P., Holland J. P. (2025). JACS Au.

[cit56] Shcherbakov D., Baev D., Kalinin M., Dalinger A., Chirkova V., Belenkaya S., Khvostov A., Krut’ko D., Medved’ko A., Volosnikova E., Sharlaeva E., Shanshin D., Tolstikova T., Yarovaya O., Maksyutov R., Salakhutdinov N., Vatsadze S. (2022). ACS Med. Chem. Lett..

[cit57] Matthews J., Veremeeva P. N., Golubeva E. A., Lavrov M. I., Radchenko E. V., Zamoyski V. L., Grigoriev V. V., Palyulin V. A. (2024). Chem. Heterocycl. Compd..

[cit58] Harutyunyan A. D., Gevorkyan K. A., Galstyan M. V., Buniatyan J. M., Muradyan R. E., Gasparyan S. P. (2022). Chem. J. Arm..

[cit59] Salykina M. A., Bunev A. S., Lozinskaya N. A., Sosonyuk S. E. (2025). Tetrahedron.

[cit60] Murineddu G., Asproni B., Corona P., Gessi S., Merighi S., Battistello E., Sturaro C., Calo G., Galeotti N., Temml V., Herdinger S., Schuster D., Pinna G. A. (2020). Bioorg. Chem..

[cit61] Suslov E. V., Ponomarev K. Y., Patrusheva O. S., Kuranov S. O., Okhina A. A., Rogachev A. D., Munkuev A. A., Ottenbacher R. V., Dalinger A. I., Kalinin M. A., Vatsadze S. Z., Volcho K. P., Salakhutdinov N. F. (2021). Molecules.

[cit62] Zhang Y. C., Gao J. Y., Shi N. Y., Zhao J. Q. (2011). Adv. Mater. Res..

[cit63] Liu X., Dong S., Lin L., Feng X. (2018). Chin. J. Chem..

[cit64] Bleher K., Cieslik P. A., Comba P. (2025). Dalton Trans..

[cit65] Mass E. B., Duarte G. V., Russowsky D. (2020). Mini Rev. Med. Chem..

[cit66] Mass E. B., de Lima C. A., D'Oca M. G. M., Sciani J. M., Longato G. B., Russowsky D. (2022). Drugs Drug-Candidates.

[cit67] Aly A. A., Hassan A. A., Makhlouf M. M., Bräse S. (2020). Molecules.

[cit68] Sirakanyan S. N., Spinelli D., Geronikaki A., Zuppiroli L., Zuppiroli R., Kartsev V., Hakobyan E. K., Yegoryan H. A., Hovakimyan A. A. (2022). Int. J. Mol. Sci..

[cit69] Sirakanyan S. N., Spinelli D., Mattioli E. J., Calvaresi M., Geronikaki A., Kartsev V. G., Hakobyan E. K., Yegoryan H. A., Jughetsyan H. V., Manukyan M. E., Hovakimyan A. A. (2024). Int. J. Mol. Sci..

[cit70] Kocharov S. L., Gevorkyan K. A., Arutyunyan A. D., Galstyan M. V., Panosyan H. A., Avakimyan J. A., Stepanyan H. M. (2023). Russ. J. Org. Chem..

[cit71] Galstyan A. S., Ghochikyan T. V., Samvelyan M. A., Frangyan V. R., Sarfraz M. (2019). ChemistrySelect.

[cit72] Boraei A. T. A., El Ashry E. S. H., Duerkop A. (2016). Chem. Cent. J..

[cit73] Petrosyan A. V., Ayvazyan A. G., Ghochikyan T. V., Galstyan A. S. (2024). Eur. J. Org Chem..

[cit74] Vogel H. G. and Vogel W. H., Psychotropic and neurotropic activity, in Drug Discovery and Evaluation: Pharmacological Assays, ed. H. E. Vogel, Springer, Berlin & N.-Y., 2008, pp. 569–874

[cit75] Loscher W., Schmidt D. (1988). Epilepsy Res..

[cit76] Swinyard E. A. , Experimental Models of Epilepsy, ed. D. P. Purpura, J. K. Penry, D. Tower, D. M. Woodbury and R. Walter, Raven Press, New-York, 1992, pp. 433–458

[cit77] Katzung B. , Drugs Used in Generalized Seizures, Basic and Clinical Pharmacology, Large Medical Books, McGraw-Hill, 9th edn, 2003

[cit78] Mironov A. H. , The 1th Part, in Manual for Preclinical Studies of Drugs, Medicine, Moscow, Russia, 2012, pp. 235–250, (In Russian)

[cit79] Mashkovsky M. D. , Medicines, 16th edn, M.: New wave, 2021, p. 1216

[cit80] Dunham N. W., Miya T. S., Edwards L. D. (1957). J. Am. Pharm. Assoc..

[cit81] Jr Litchfield J. T., Wilcoxon F. (1949). J. Pharmacol. Exp. Ther..

[cit82] Tallarida R. J. and Murray R. B., Litchfield and Wilcoxon I: Confidence Limits of ED50, in Manual of Pharmacologic Calculations, Springer, New York, NY, 1987, p. 153

[cit83] File S. E. (2001). Behav. Brain Res..

[cit84] Rogawski M. A., Löscher W. (2004). Nat. Rev. Neurosci..

[cit85] Stanford S. C. (2007). J. Psychopharmacol..

[cit86] Prut L., Belzung C. (2003). Eur. J. Pharmacol..

[cit87] Pellow S., Chopin P., File S. E., Briley M. (1985). J. Neurosci. Methods.

[cit88] Pellow S., File S. E. (1986). Pharmacol. Biochem. Behav..

[cit89] Jardim M. C., Nogueira R. L., Graeff F. G., Nunes-De-Souza R. L. (1999). Brain Res. Bull..

[cit90] Graeff F. G., Netto C. F., Zangrossi J. (1998). Neurosci. Biobehav. Rev..

[cit91] Porsolt R. D., Anton G., Blavet N., Jalfre M. (1978). Eur. J. Pharmacol..

[cit92] Can A., Dao D. T., Terrillion C. E., Piantadosi S. C., Bhat S., Gould T. D. (2012). J. Vis. Exp..

[cit93] Castagné V., Moser P., Roux S., Porsolt R. D. (2010). Curr. Protoc. Pharmacol..

[cit94] Ferreira M. F., Castanheira L., Sebastião A. M., Telles-Correia D. (2018). Curr. Top. Med. Chem..

[cit95] Rogawski M. A., Löscher W. (2004). Nat. Rev. Neurosci..

[cit96] Kammerer M., Rassner M. P., Freiman T. M., Feuerstein T. J. (2011). Naunyn Schmiedebergs Arch. Pharmacol..

[cit97] Miller P. S., Aricescu A. R. (2014). Nature.

[cit98] Saier Jr M. H. (2000). Microbiol. Mol. Biol. Rev..

[cit99] Perrone R., Berardi F., Colabufo N. A., Lacivita E., Larizza C., Leopoldo M., Tortorella V. (2005). J. Pharm. Pharmacol..

[cit100] Blier P. (2001). J. Clin. Psychiatry.

[cit101] Coleman J., Green E., Gouaux E. (2016). Nature.

[cit102] Kuipers W., Link R., Standaar P. J., Stoit A. R., Wijngaarden V. I., Leurs R., Ijzerman A. P. (1997). Mol. Pharmacol..

[cit103] Wacker D., Fenalti G., Brown M. A., Katritch V., Abagyan R., Cherezov V., Stevens R. C. (2010). J. Am. Chem. Soc..

