# Supplementary material for: Synthesis, neurotropic activity and docking studies of 1,2,4-triazole-linked hybrids based on 2,7-naphthyridine and bispidine rings
Source: RSC Adv. 2026 Jul 3;16(35):36518–40. doi: 10.1039/d6ra00302h (PMC13330782; doi:10.1039/d6ra00302h)

Supplementary Information (SI) for RSC Advances.
This journal is © The Royal Society of Chemistry 2026

Supplementary Data \ File 1

Synthesis, neurotropic activity and docking studies of 1,2,4-triazole-linked hybrids based on 2,7-naphthyridine and bispidine rings

Samvel N. Sirakanyan,[a] Athina Geronikaki,[b,*] Anush A. Hovakimyan,[a,*] Anthi Petrou,[b] Victor G. Kartsev,[c] Hasmik A. Yegoryan,[a] Hasmik V. Jughetsyan,[a] Sahak P. Gasparyan,[a] Ruzanna G. Paronikyan,[a,*] Tatevik A. Araqelyan,[a] Mariam V. Galstyan,[a] Knarik A. Gevorkyan,[a] Amalya D. Harutyunyan,[a] and Elmira K. Hakobyan[a]

[a] Scientific Technological Center of Organic and Pharmaceutical Chemistry of National Academy of Science of Republic of Armenia, Institute of Fine Organic Chemistry of A.L.Mnjoyan, Armenia 0014, Yerevan; shnnr@mail.ru

[b] Department of Pharmacy, School of Health, Aristotle University of Thessaloniki, 54124 Thessaloniki, Greece; geronik@pharm.auth.gr

[c] InterBioScreen, Moscow 119019, Russia; vkartsev@ibscreen.chg.ru

* Correspondence: geronik@pharm.auth.gr (A.G.); anush.hovakimyan@gmail.com (A.A.H.); paronikyan.ruzanna@mail.ru (R.G.P.)

^{1}H and ^{13}C NMR spectra for new synthesized compounds: 2b,d–h; 3a–h; 4a–h; 5a–h; 7a,b and 8a,b

2б

Molecular Structure Research Centre, Yerevan, Armenia, Varian Mercury-300VX

H1 300.088 MHz, nt=16, np=32000, temp=30.0 C, lb=-0.2, solvent=DMSO/CCl4 1/3

Mar 28 2022

ANUSH_TEMA t21-141

T21-141

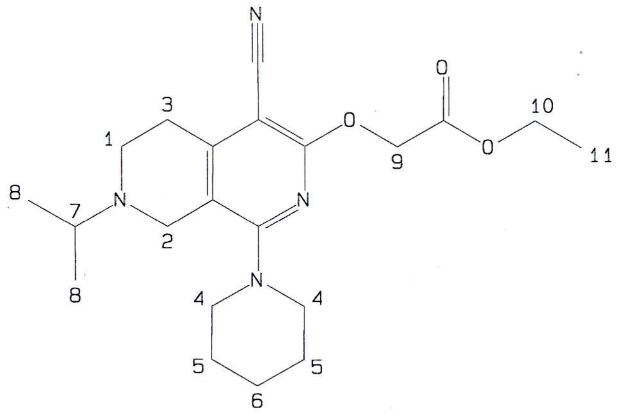

$C_{21}H_{30}N_4O_3$

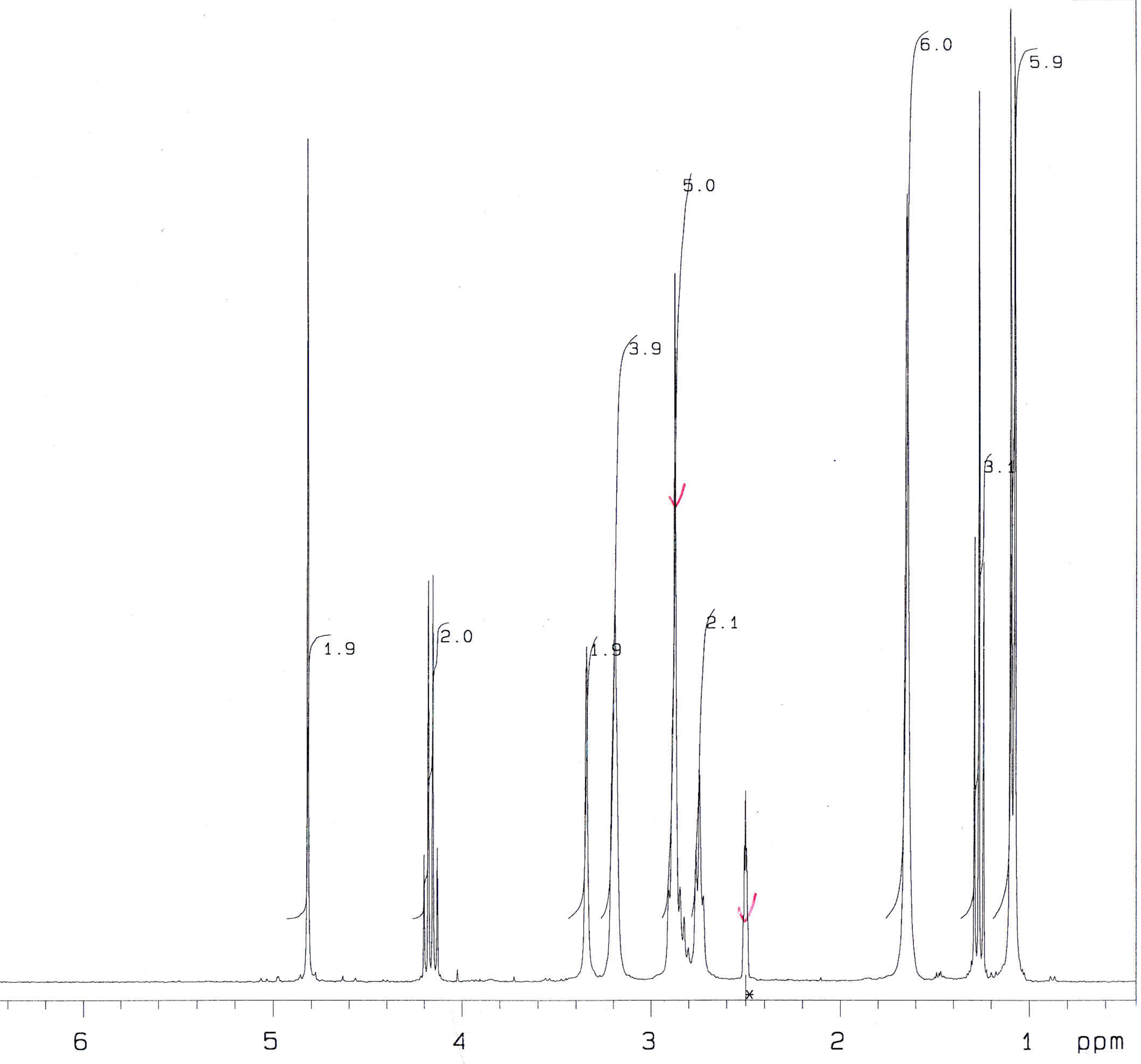

+

26

Molecular Structure Research Centre, Yerevan, Armenia, Varian Mercury-300VX C13 75.465 MHz, nt=672, np=19998, temp=30.0 C, lb=1.0, solvent=DMSO/CCl4 1/3 **Mar 28 2022**

T21-141

ANUSH_TEMA t21-141

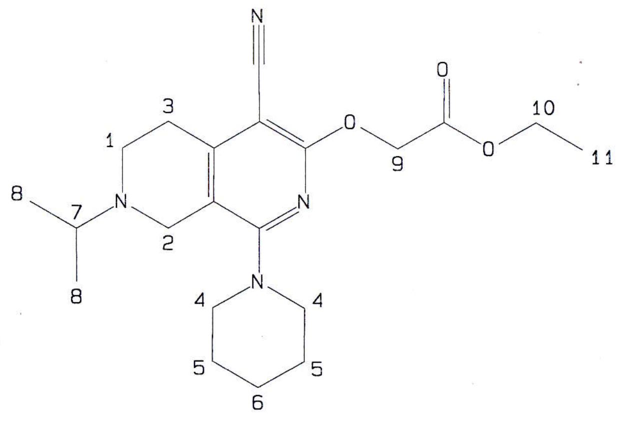

$C_{21}H_{30}N_4O_3$

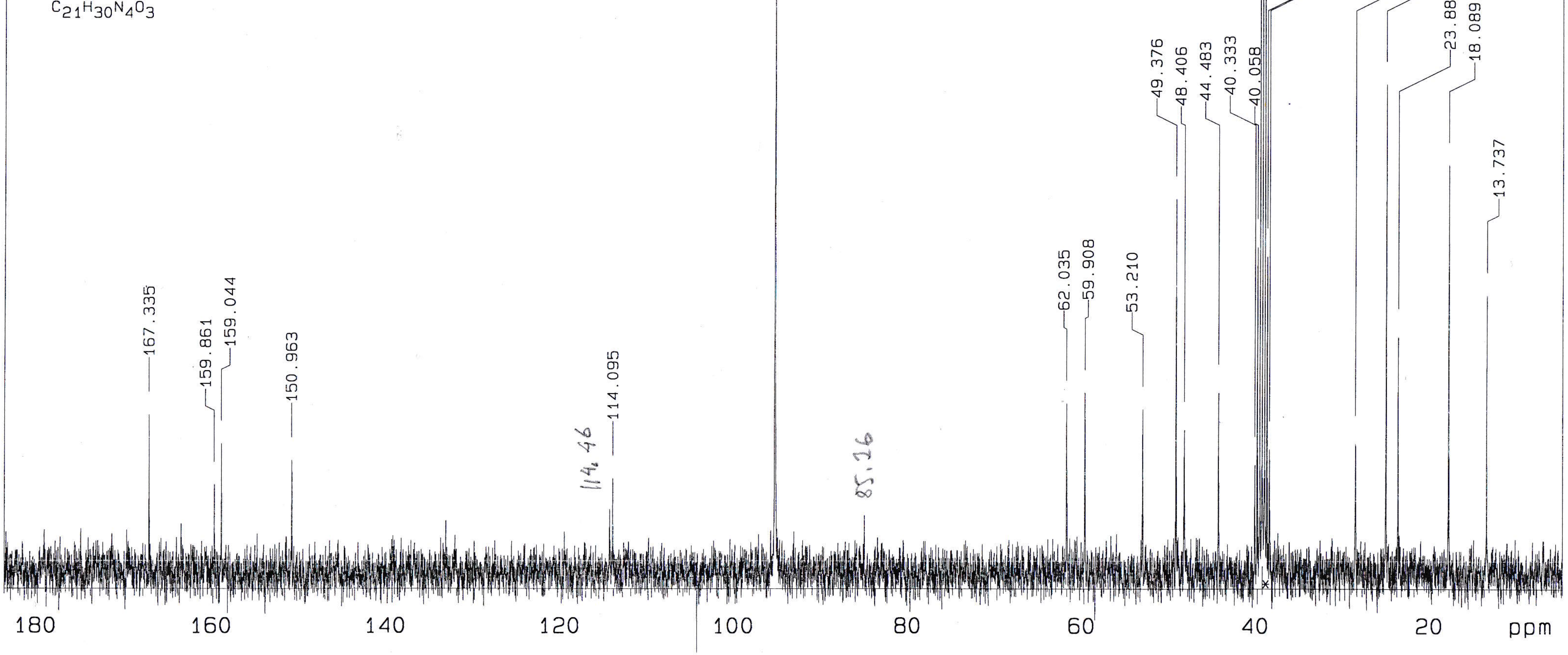

2d

Molecular Structure Research Centre, Yerevan, Armenia, Varian Mercury-300VX

H1 300.088 MHz, nt = 16, np = 32000, temp = 30.0 C, lb = -0.2, solvent = DMSO

Feb 24 2022

NOCI_22 t21-110

T21-110

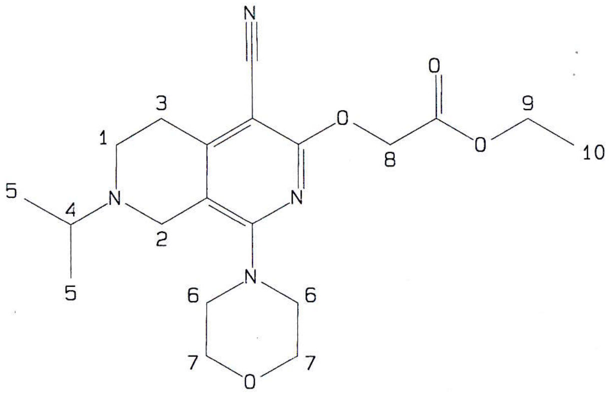

$C_{20}H_{28}N_4O_4$

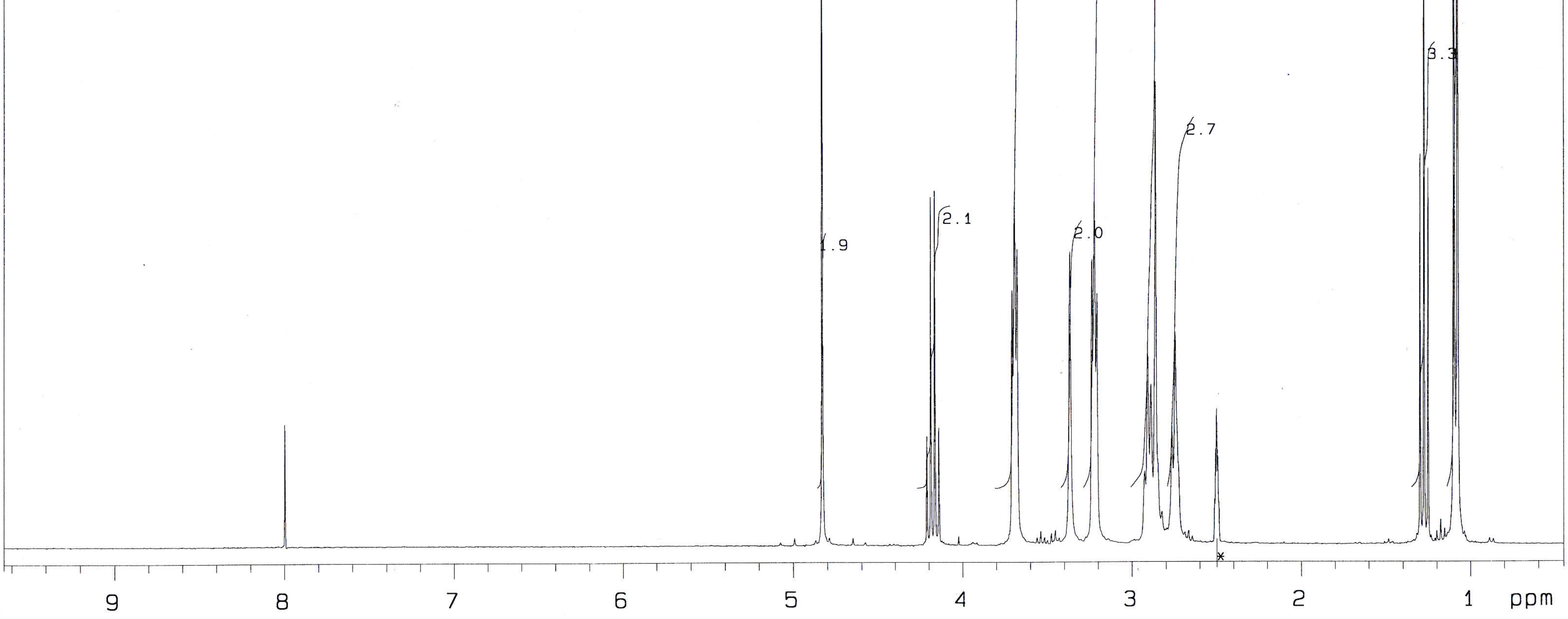

+

2d

Molecular Structure Research Centre, Yerevan, Armenia, Varian Mercury-300VX

C13 75.465 MHz, nt=320, np=19998, temp=30.0 C, lb=1.0, solvent=DMSO/CCl4 1/3

Feb 24 2022

NOCI_22 t21-110

T21-110

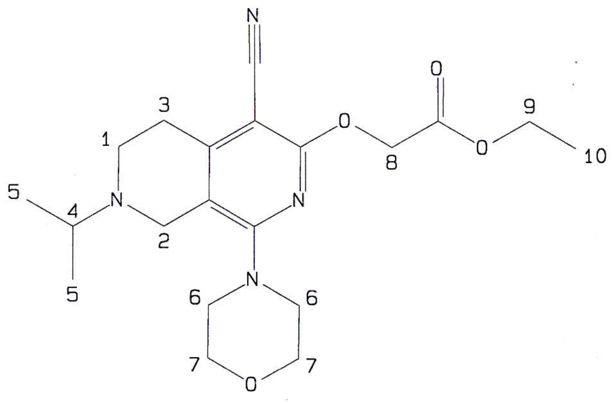

$C_{20}H_{28}N_4O_4$

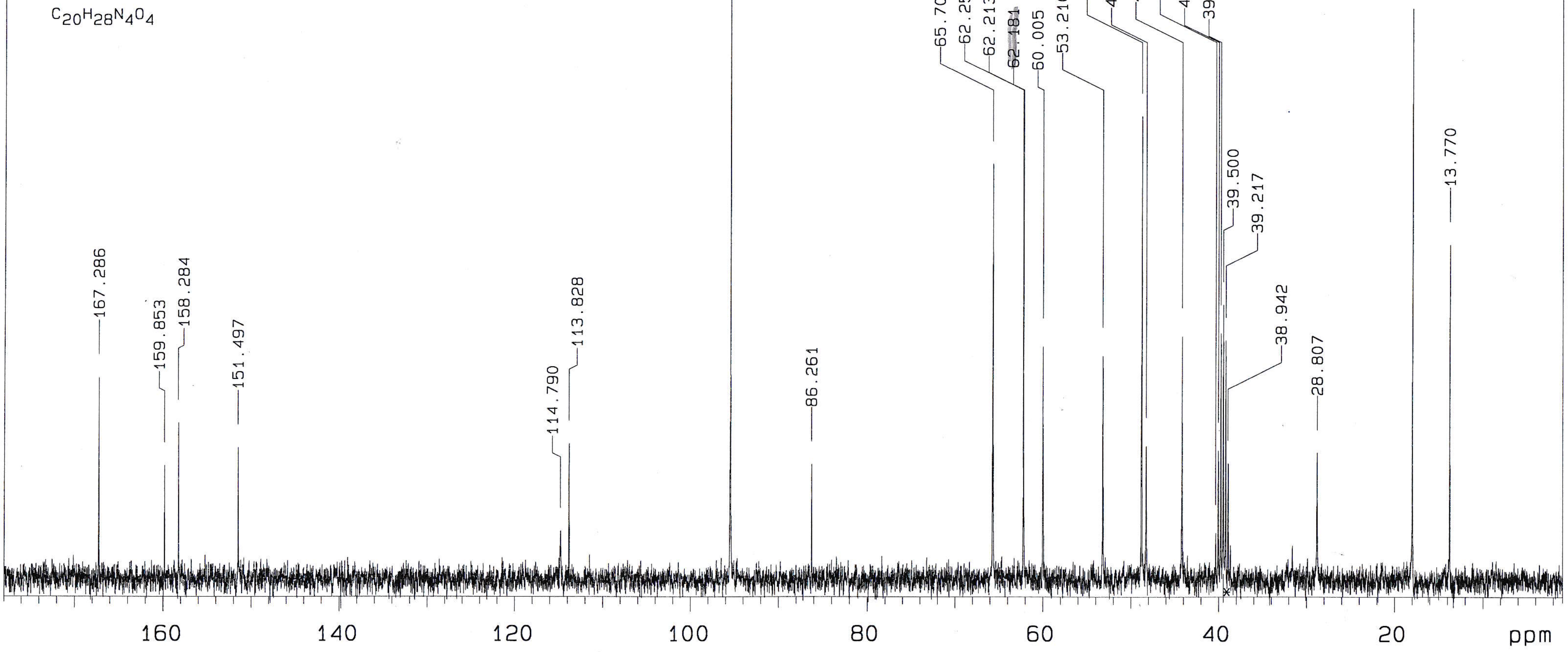

2e

Molecular Structure Research Centre, Yerevan, Armenia, Varian Mercury-300VX

H1 300.088 MHz, nt = 16, np = 32000, temp = 30.0 C, lb = -0.2, solvent = DMSO/CCl4 1/3

Apr 4 2023

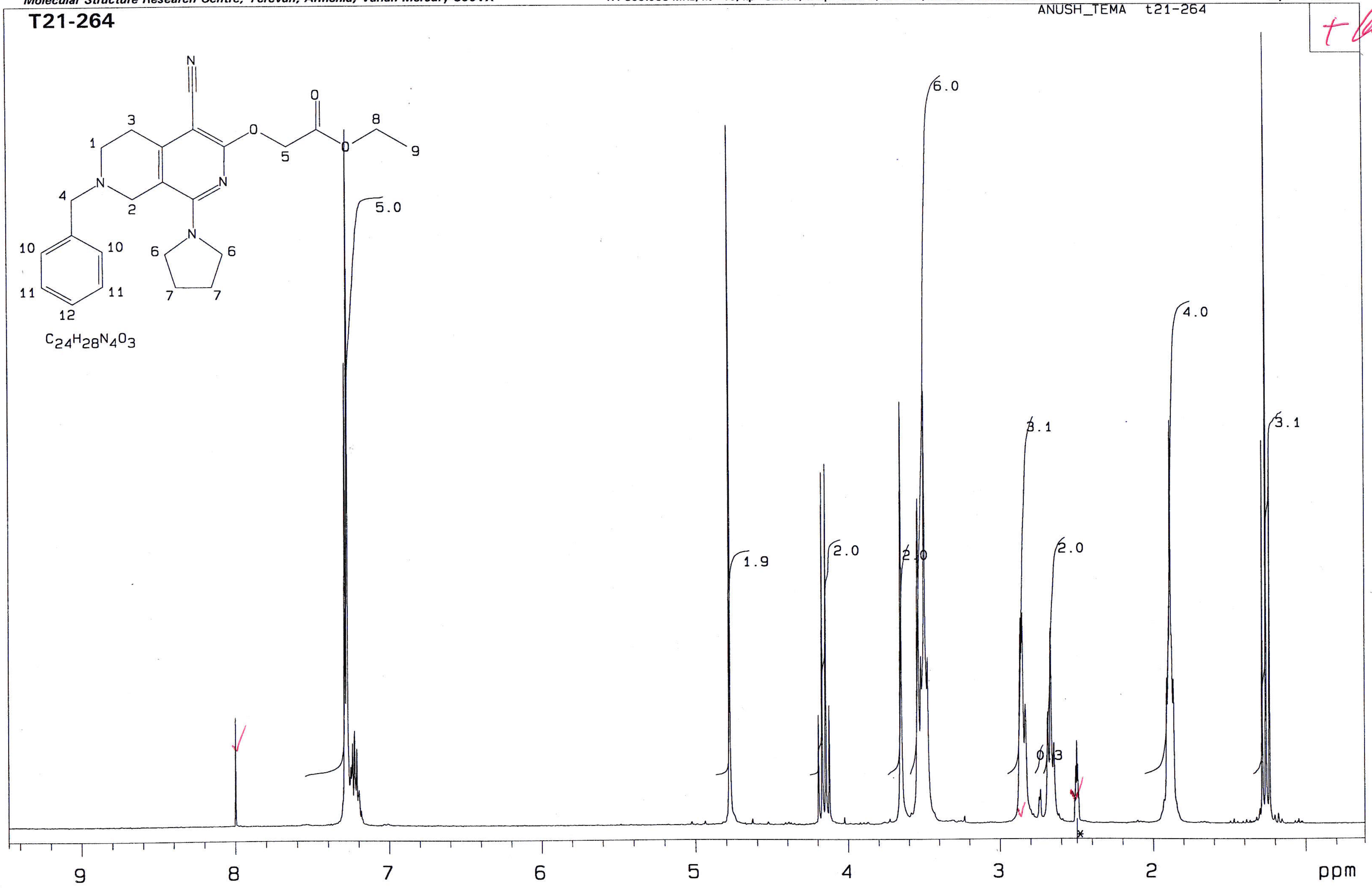

2e

Molecular Structure Research Centre, Yerevan, Armenia, Varian Mercury-300VX

C13 75.465 MHz, nt=400, np=19998, temp=30.0 C, lb=1.0, solvent=DMSO/CCl4 1/3

Apr 4 2023

ANUSH_TEMA t21-264

T21-264

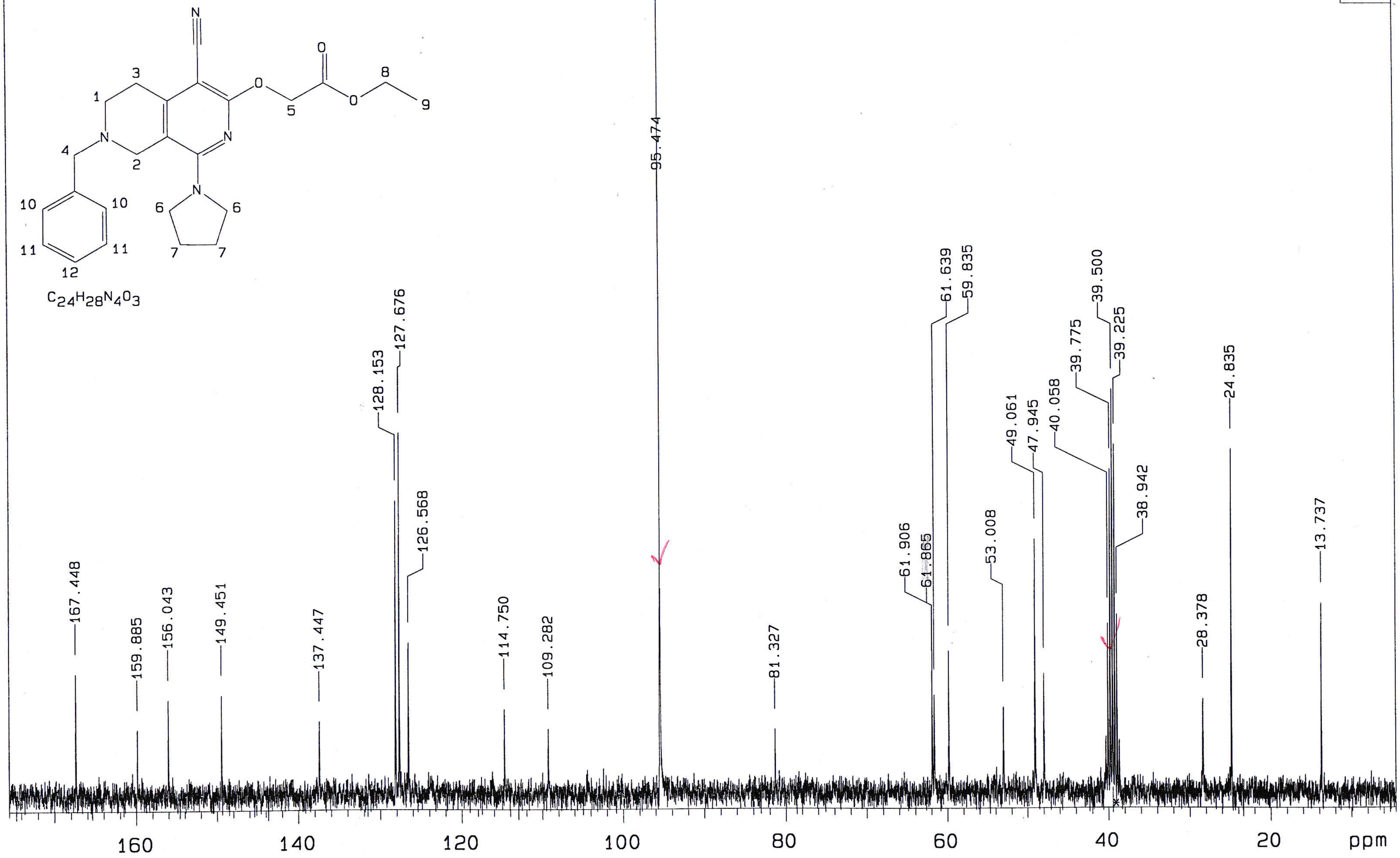

2f

Molecular Structure Research Centre, Yerevan, Armenia, Varian Mercury-300VX

H1 300.088 MHz, nt=16, np=32000, temp=30.0 C, lb=-0.2, solvent=DMSO/CCl4 1/3

Apr 20 2023

ANUSH_TEMA t21-273

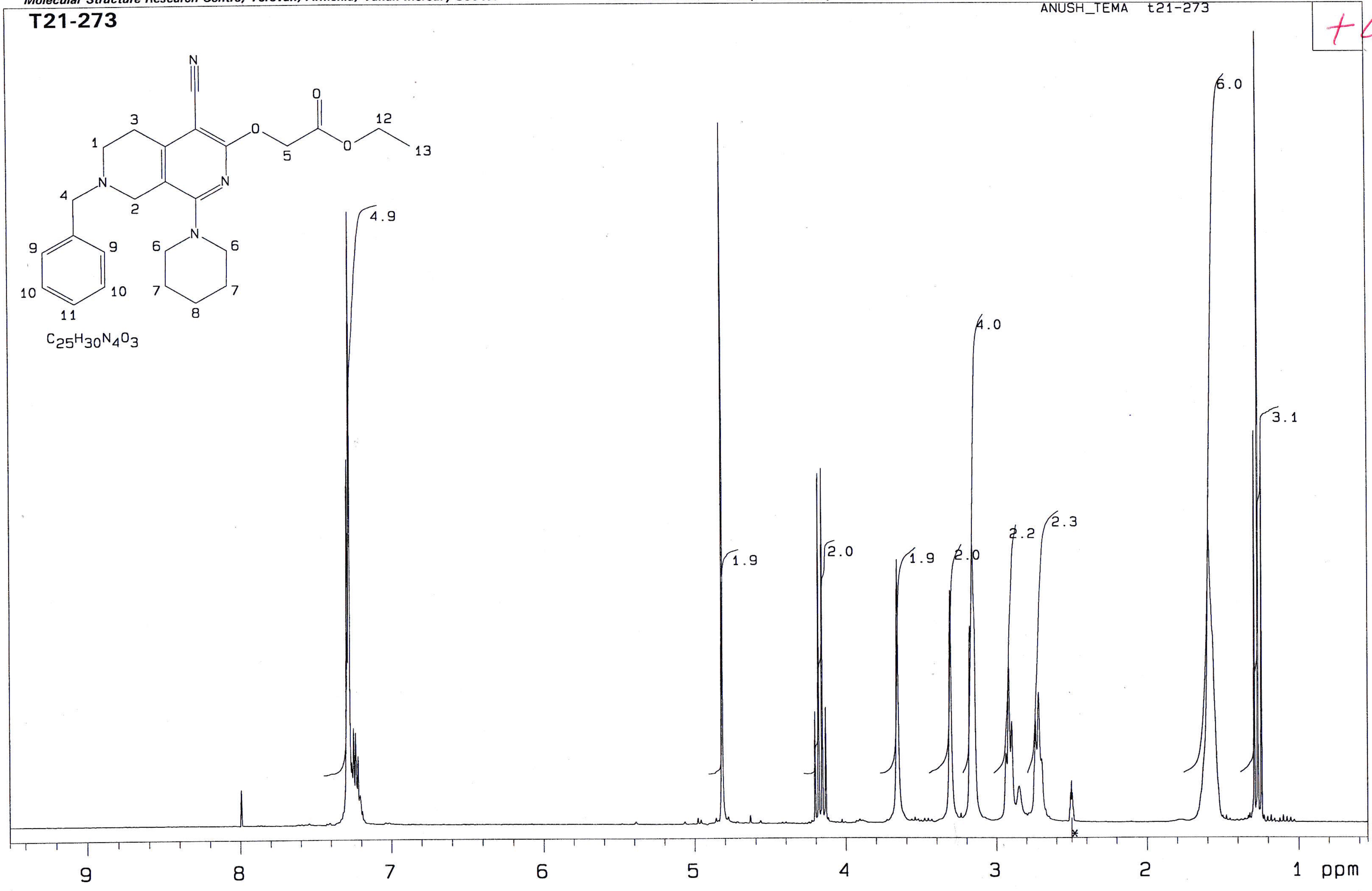

+

Molecular Structure Research Centre, Yerevan, Armenia, Varian Mercury-300VX   C13 75.465 MHz, nt=400, np=19998, temp=30.0 C, lb=1.0, solvent=DMSO/CCl4 1/3   **Apr 20 2023**

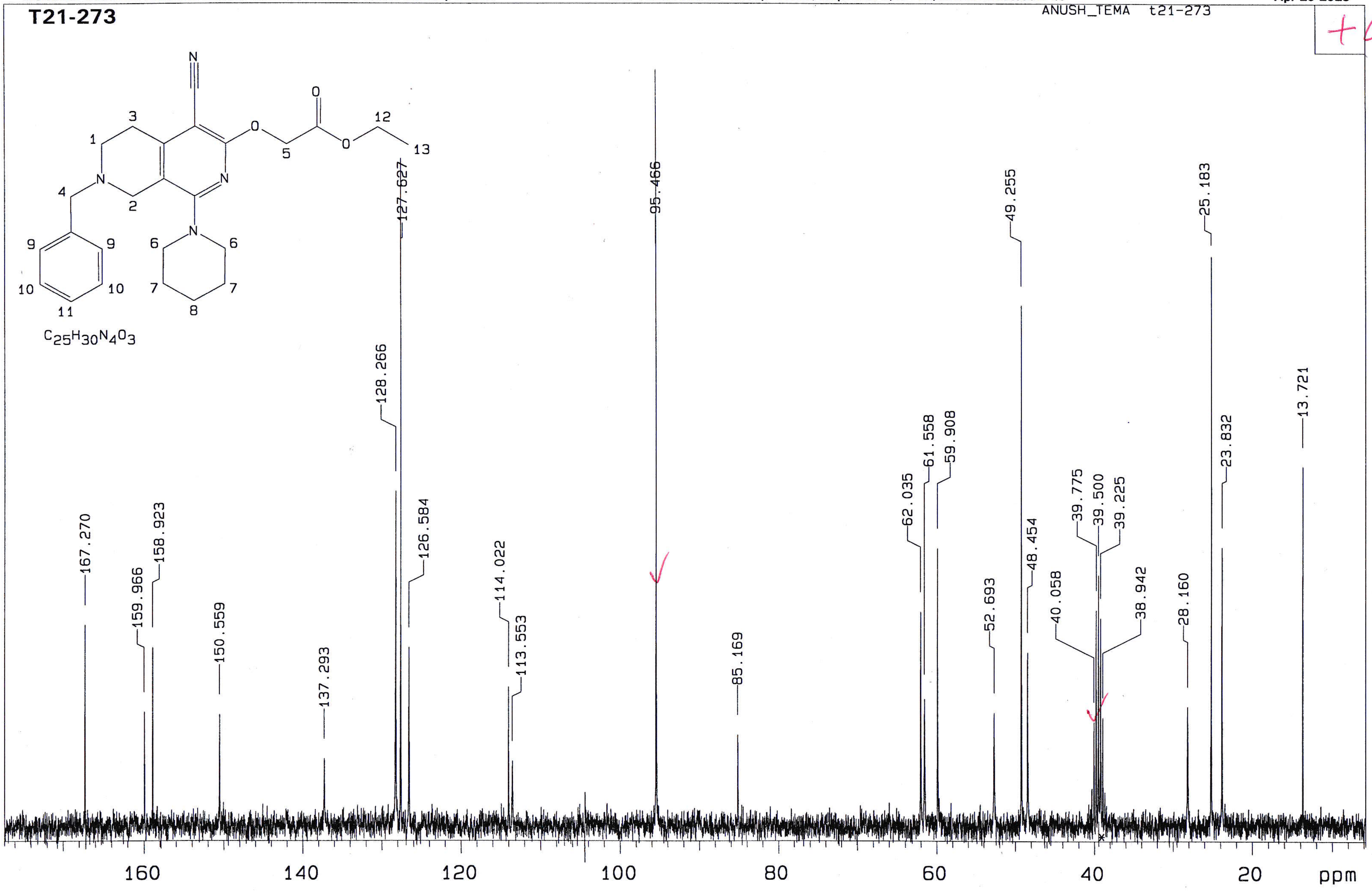

29

Molecular Structure Research Centre, Yerevan, Armenia, Varian Mercury-300VX

H1 300.088 MHz, nt=16, np=32000, temp=30.0 C, lb=-0.2, solvent=DMSO/CCl4 1/3

Mar 7 2023

ANUSH_TEMA t21-240

T21-240

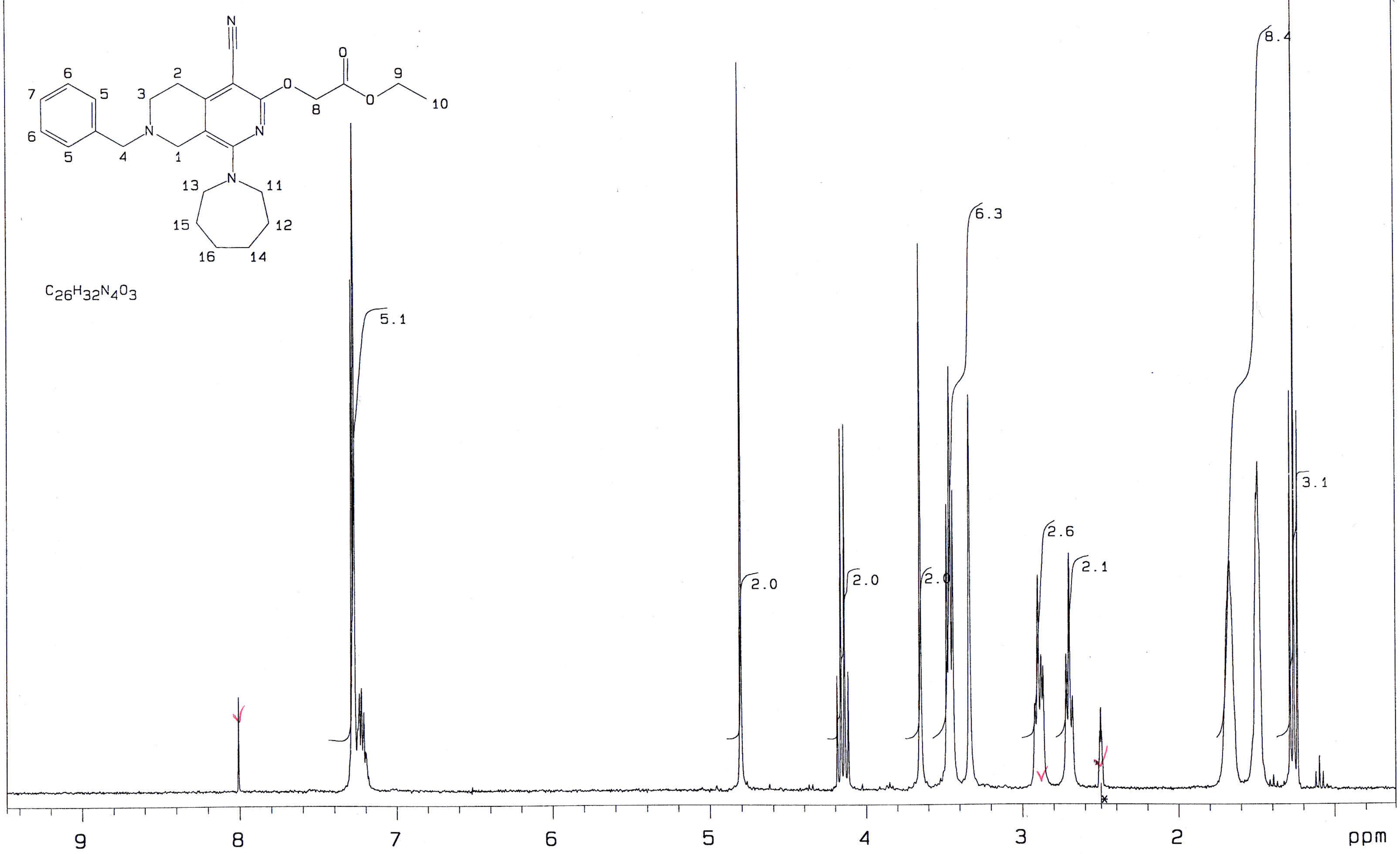

Molecular Structure Research Centre, Yerevan, Armenia, Varian Mercury-300VX

C13 75.465 MHz, nt = 288, np = 19998, temp = 30.0 C, lb = 1.0, solvent = DMSO/CCl4 1/3

Mar 7 2023

ANUSH_TEMA t21-240

T21-240

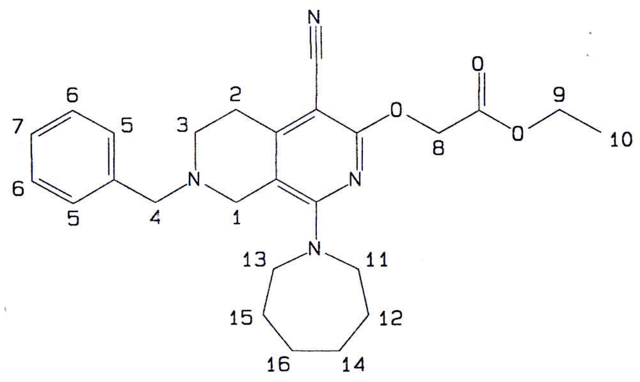

$C_{26}H_{32}N_4O_3$

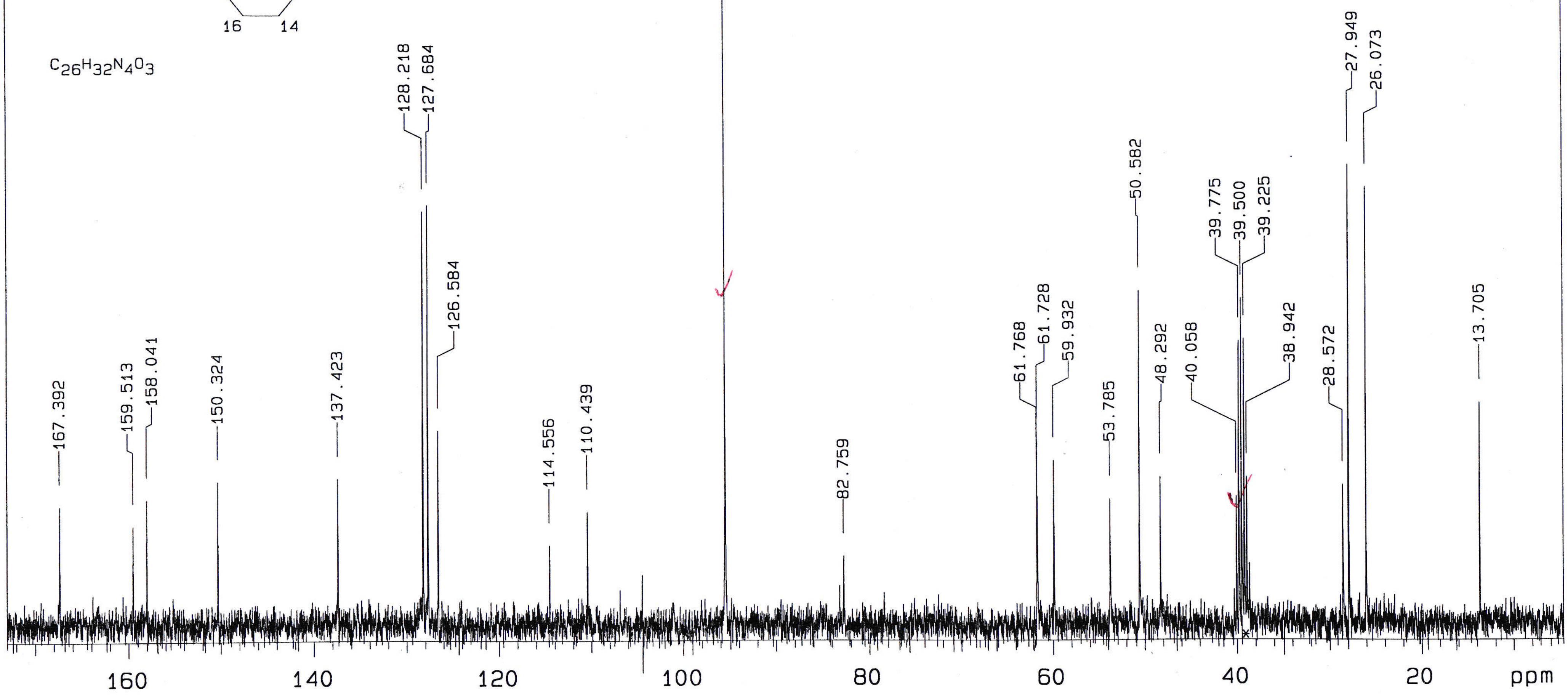

2h

Molecular Structure Research Centre, Yerevan, Armenia, Varian Mercury-300VX

H1 300.088 MHz, nt = 16, np = 32000, temp = 30.0 C, lb = -0.2, solvent = DMSO/CCl4 1/3

Apr 14 2023

ANUSH_TEMA t21-269

T21-269

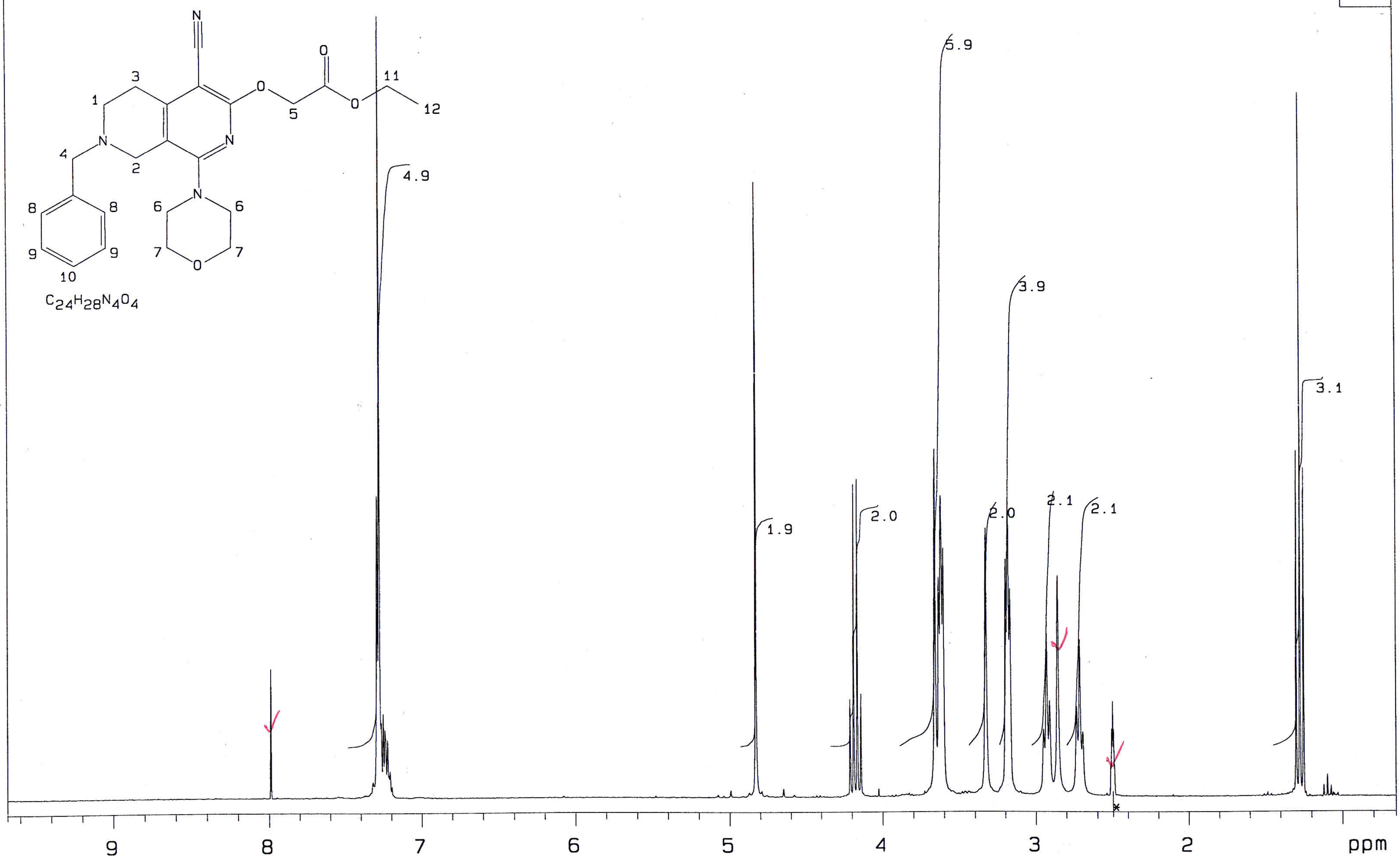

2h

Molecular Structure Research Centre, Yerevan, Armenia, Varian Mercury-300VX   C13 75.465 MHz, nt=480, np=19998, temp=30.0 C, lb=1.0, solvent=DMSO/CCl4 1/3   **Apr 14 2023**

ANUSH_TEMA t21-269

T21-269

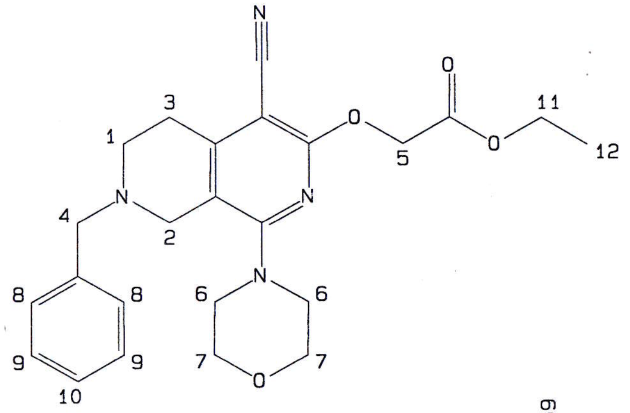

$C_{24}H_{28}N_4O_4$

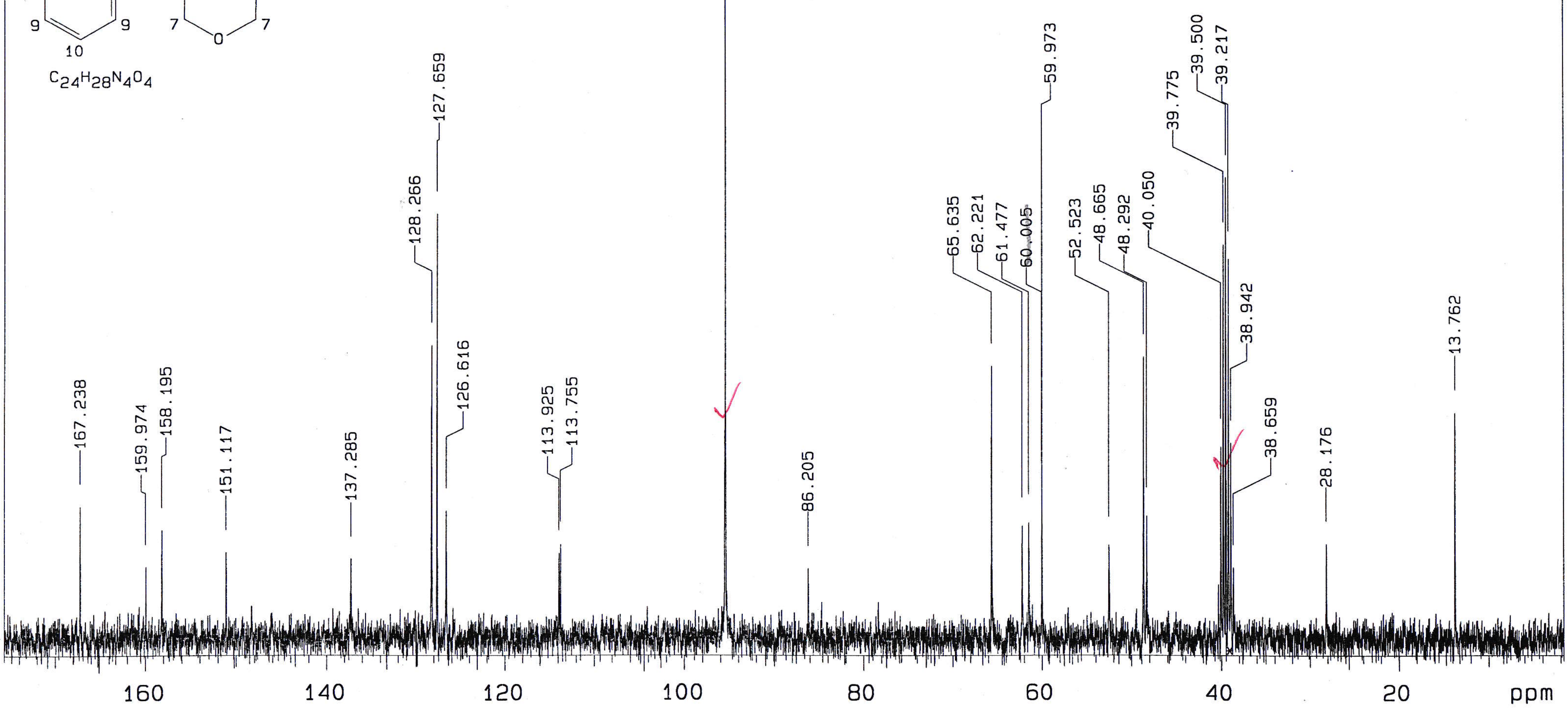

3a

Molecular Structure Research Centre, Yerevan, Armenia, Varian Mercury-300VX

H1 300.088 MHz, nt = 16, np = 32000, temp = 30.0 C, lb = -0.2, solvent = DMSO/CCl4 1/3

May 10 2021

ANUSH_TEMA s12-007

S12-007 T21-085

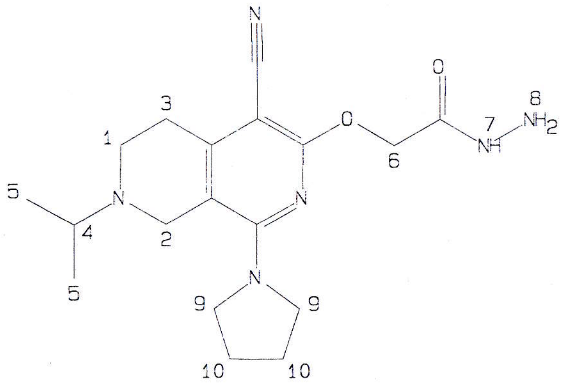

$C_{18}H_{26}N_6O_2$

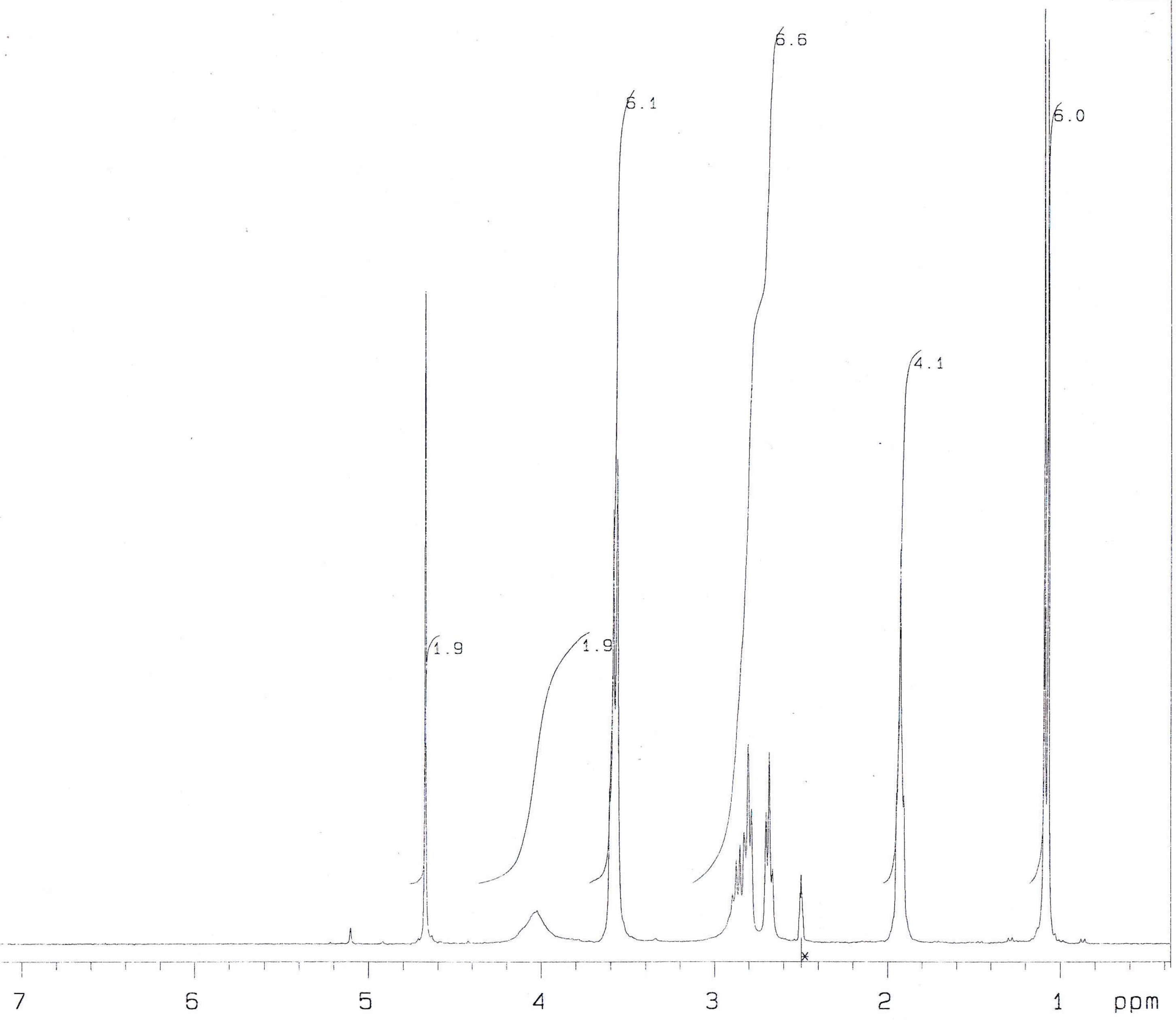

3a

Molecular Structure Research Centre, Yerevan, Armenia, Varian Mercury-300VX

C13 75.465 MHz, nt = 256, np = 19998, temp = 30.0 C, lb = 2.0, solvent = DMSO/CCl4 1/3

May 10 2021

ANUSH_TEMA s12-007

S12-007 T21-085

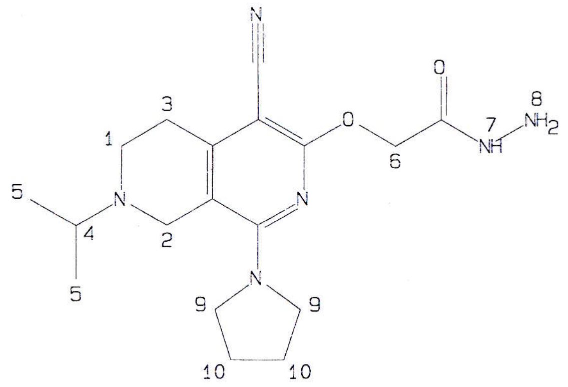

$C_{18}H_{26}N_6O_2$

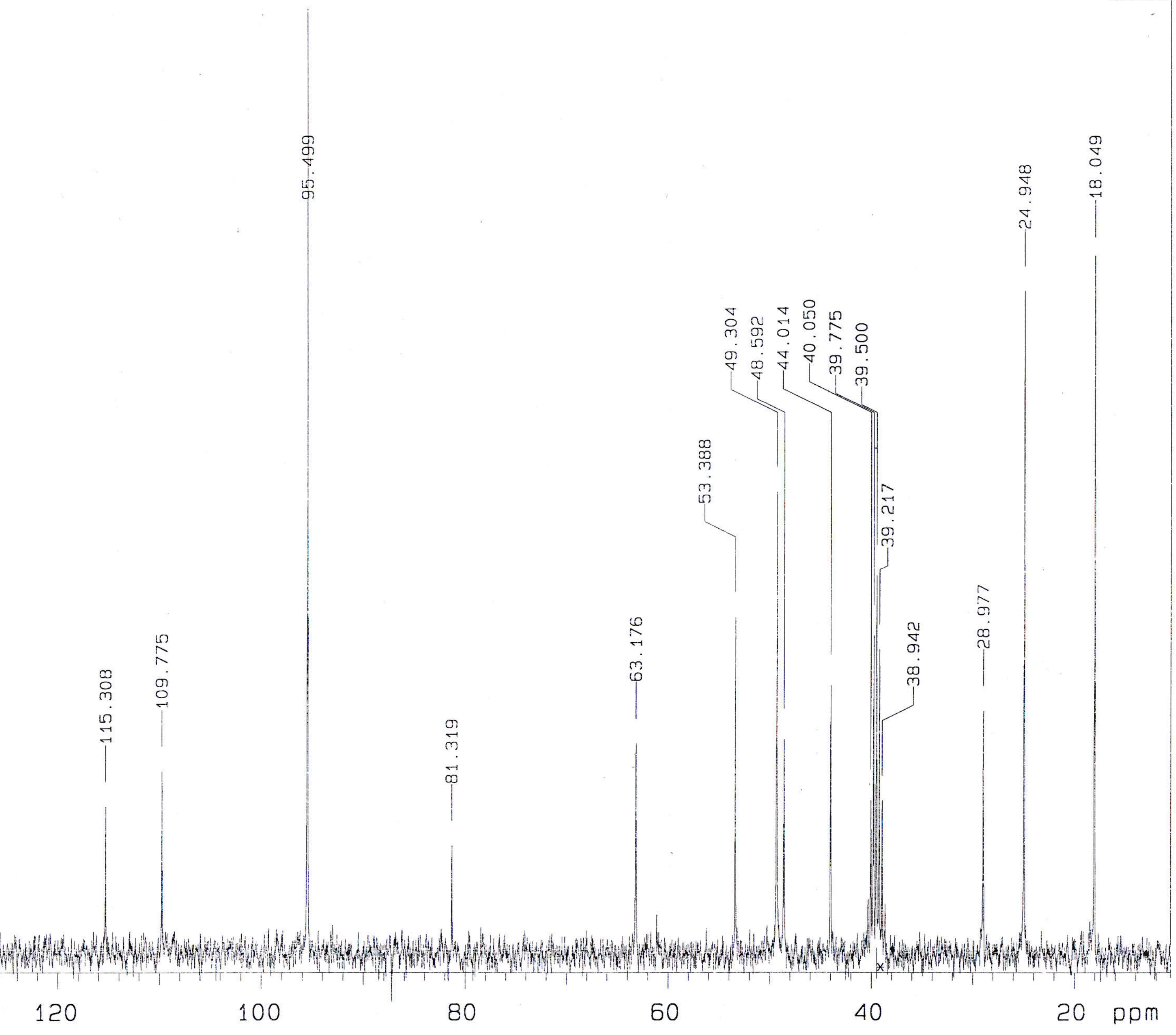

3b

Molecular Structure Research Centre, Yerevan, Armenia, Varian Mercury-300VX

H1 300.088 MHz, nt = 16, np = 32000, temp = 30.0 C, lb = -0.2, solvent = DMSO/CCl4 1/3

Apr 4 2022

NOCI_22 t21-147

T21-147

$C_{19}H_{28}N_6O_2$

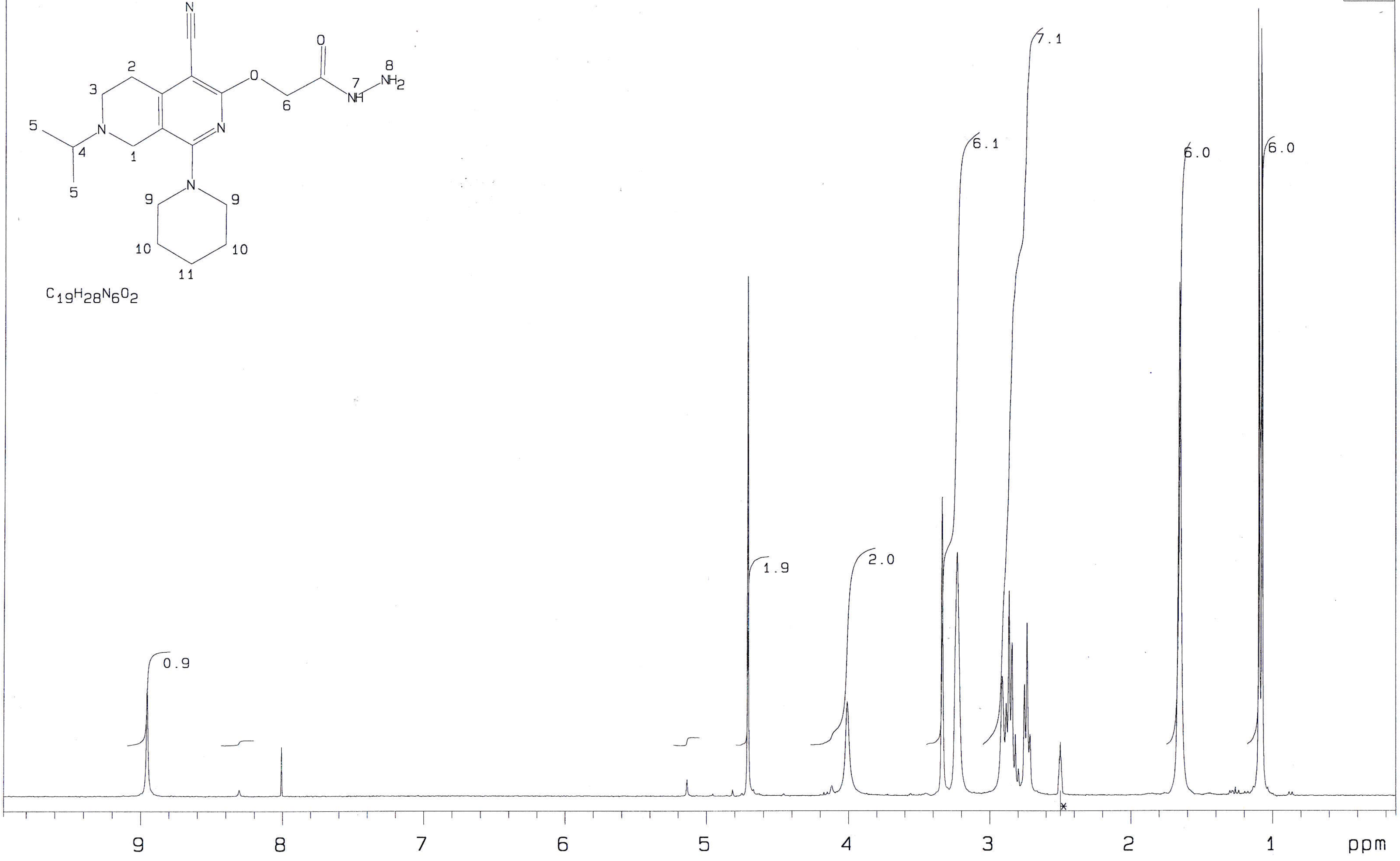

36

Molecular Structure Research Centre, Yerevan, Armenia, Varian Mercury-300VX

C13 75.465 MHz, nt = 256, np = 19998, temp = 30.0 C, lb = 1.0, solvent = DMSO/CCl4 1/3

Apr 4 2022

NOCI_22 t21-147

T21-147

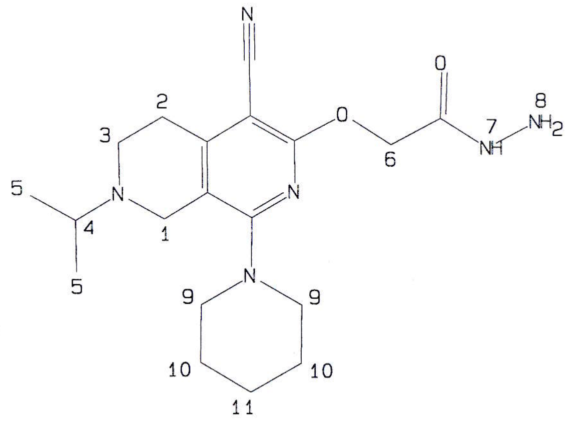

$C_{19}H_{28}N_6O_2$

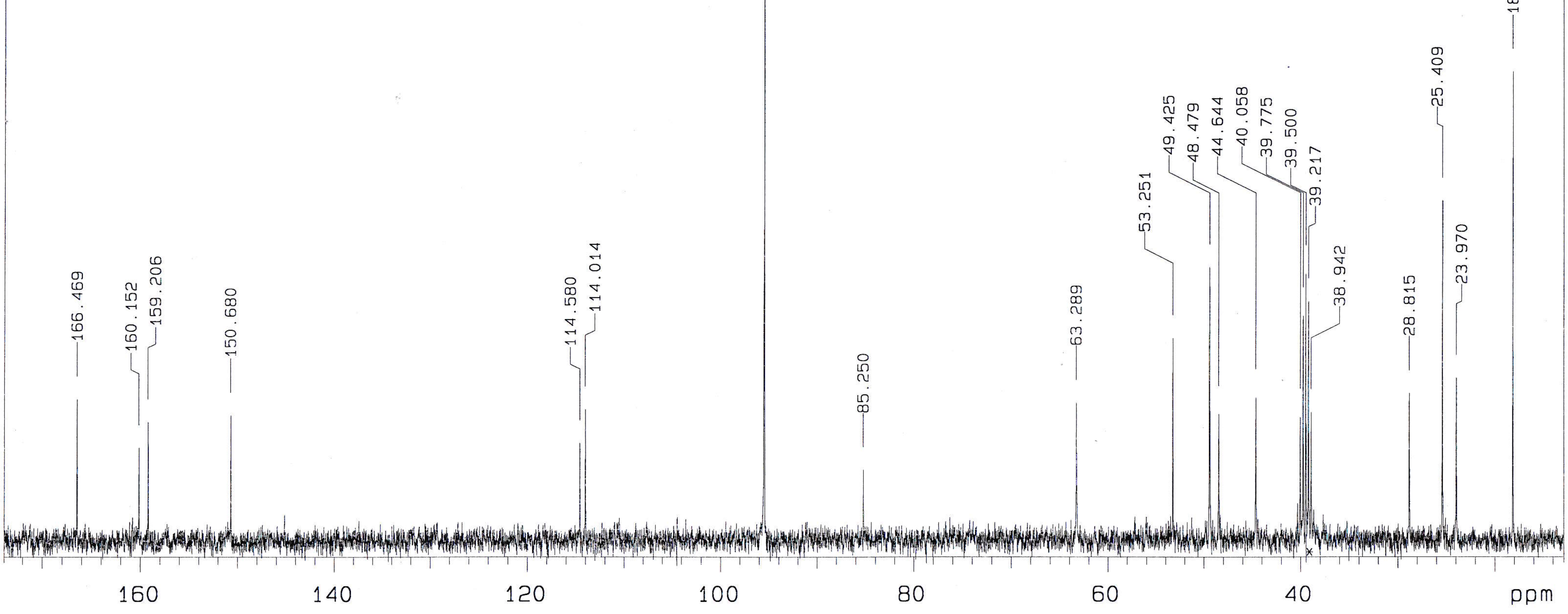

T21-037 3c

Molecular Structure Research Centre, Yerevan, Armenia, Varian Mercury-300VX H1 300.088 MHz, nt=16, np=32000, temp=30.0 C, lb=-0.2, solvent=DMSO/CCl4 1/3 May 25 2021

NOCI_21 s12-015

S12-015

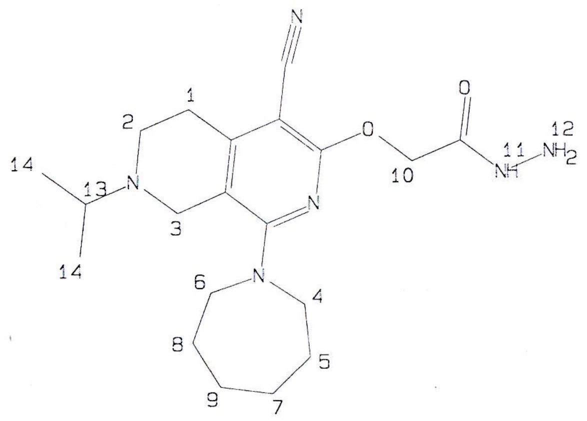

$C_{20}H_{30}N_6O_2$

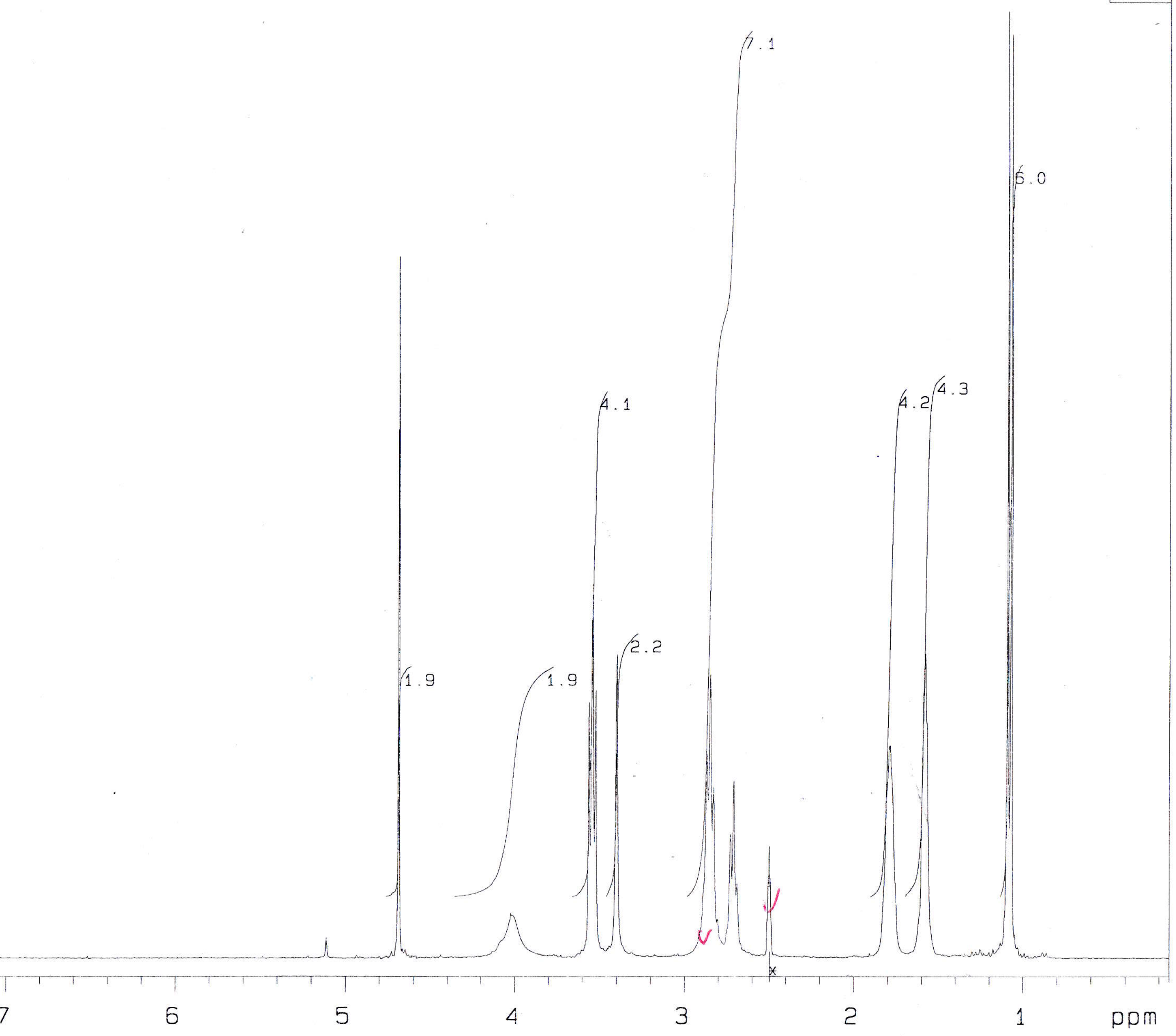

T21-037 3e

Molecular Structure Research Centre, Yerevan, Armenia, Varian Mercury-300VX C13 75.465 MHz, nt = 400, np = 19998, temp = 30.0 C, lb = 1.0, solvent = DMSO/CCl4 1/3 May 25 2021

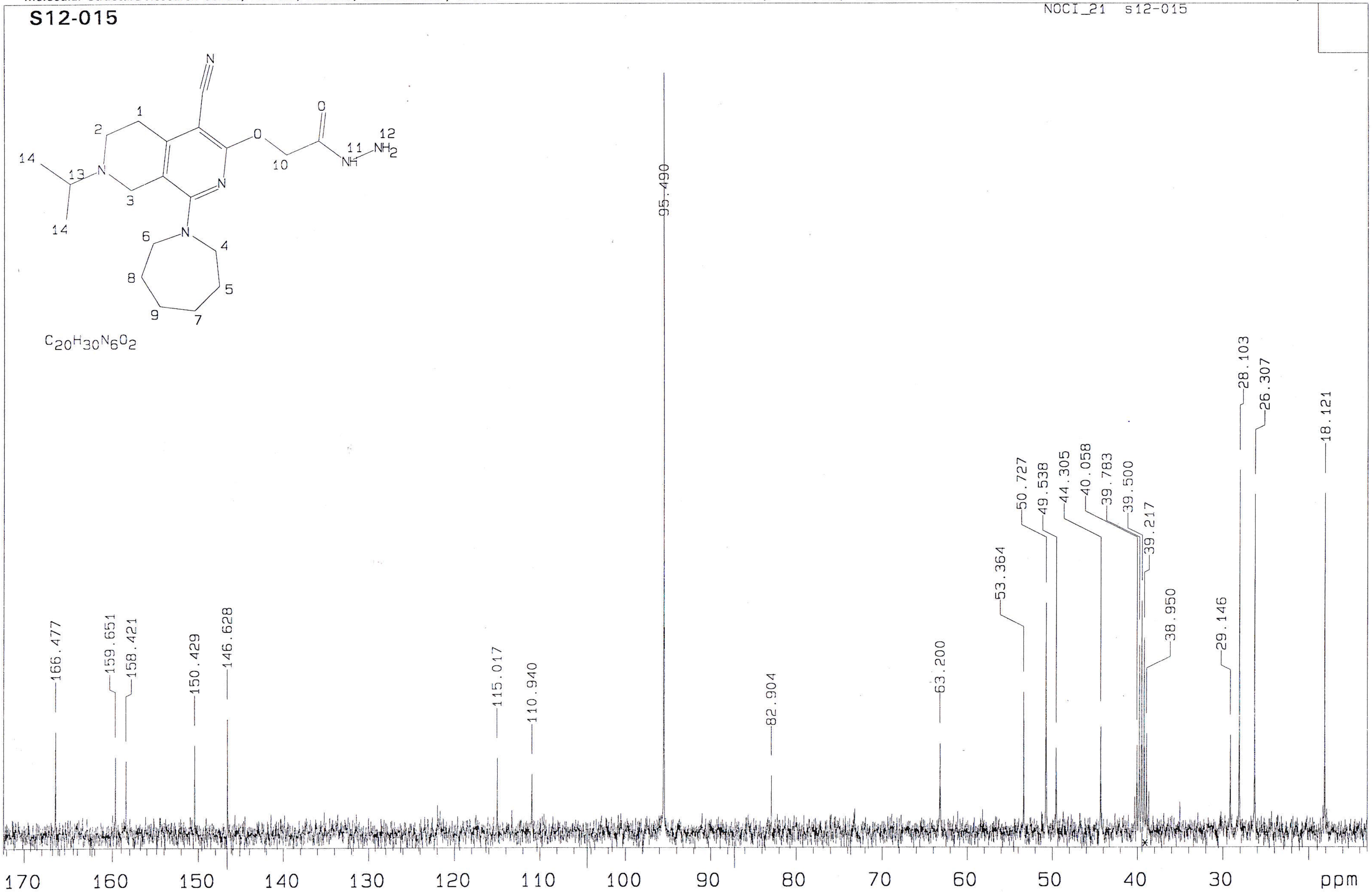

3d

Molecular Structure Research Centre, Yerevan, Armenia, Varian Mercury-300VX

H1 300.088 MHz, nt = 16, np = 32000, temp = 30.0 C, lb = -0.2, solvent = DMSO/CCl4 1/3

May 18 2022

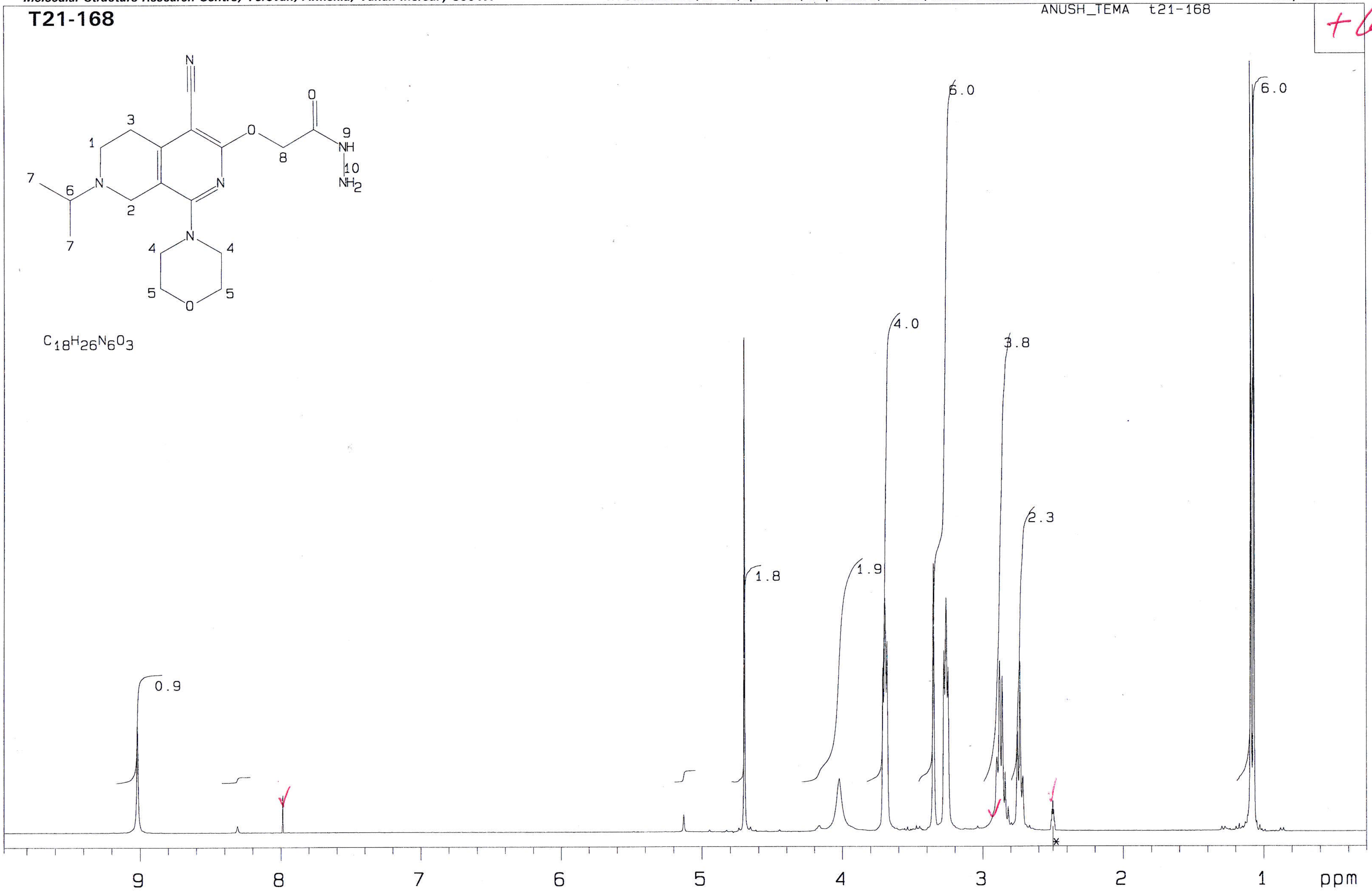

Molecular Structure Research Centre, Yerevan, Armenia, Varian Mercury-300VX

C13 75.465 MHz, nt = 160, np = 19998, temp = 30.0 C, lb = 1.0, solvent = DMSO/CCl4 1/3

May 18 2022

ANUSH_TEMA t21-168

T21-168

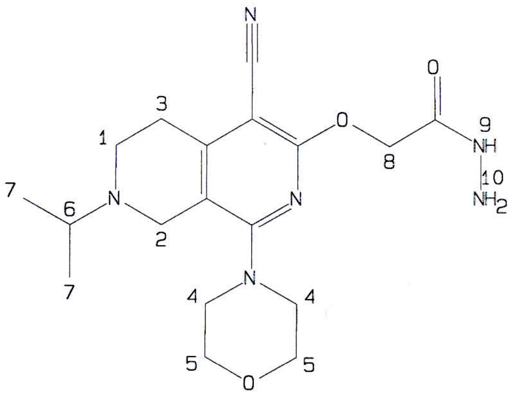

$C_{18}H_{26}N_6O_3$

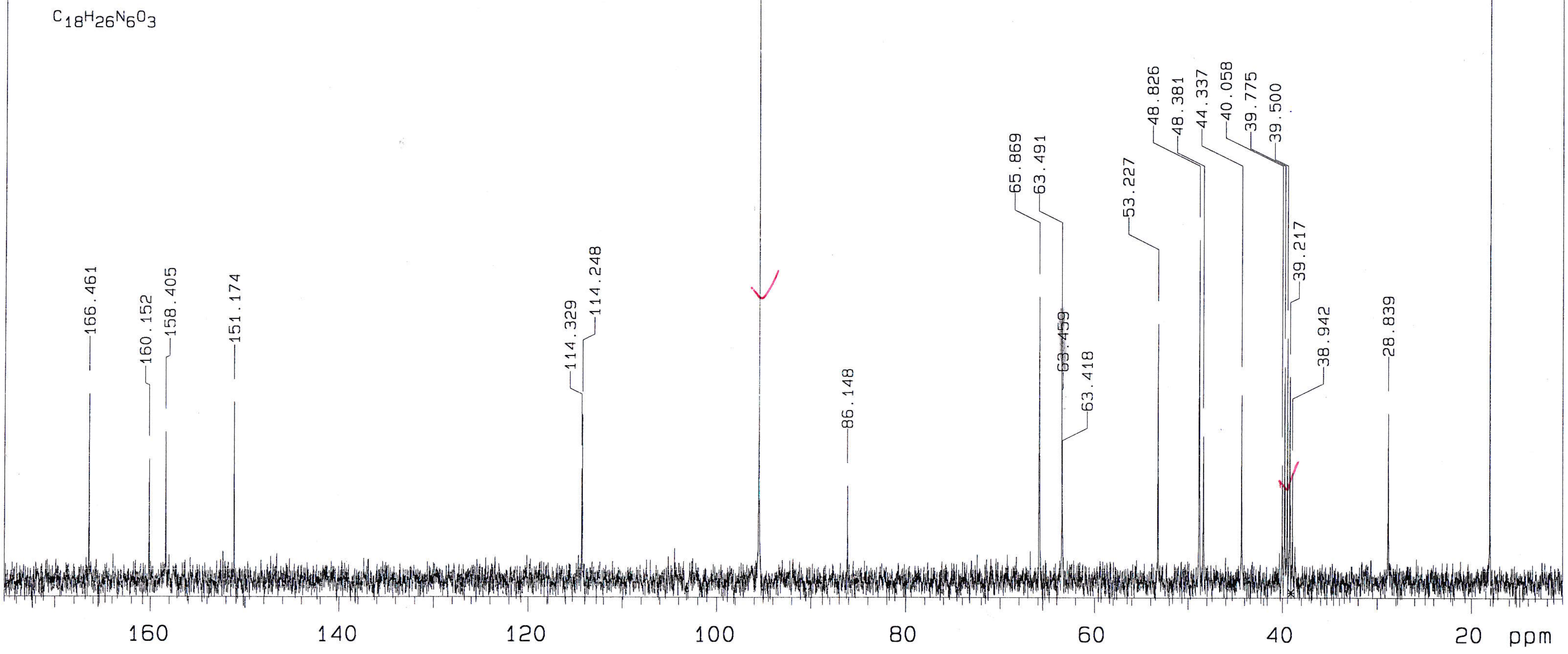

3e

Molecular Structure Research Centre, Yerevan, Armenia, Varian Mercury-300VX

H1 300.088 MHz, nt = 16, np = 32000, temp = 30.0 C, lb = -0.2, solvent = DMSO/CCl4 1/3

May 10 2023

ANUSH_TEMA t21-287

T21-287

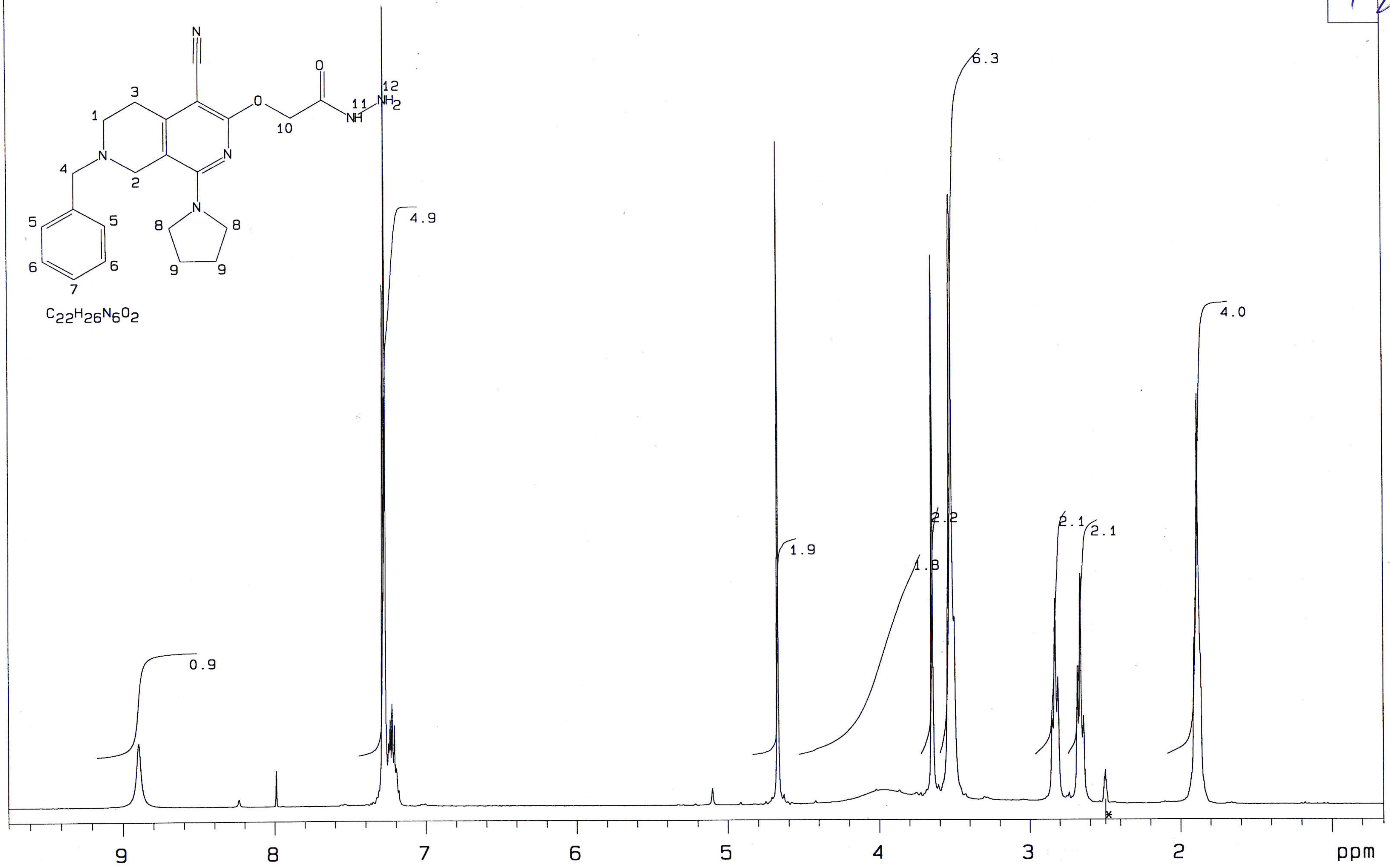

+

3e

Molecular Structure Research Centre, Yerevan, Armenia, Varian Mercury-300VX

C13 75.465 MHz, nt=224, np=19998, temp=30.0 C, lb=1.0, solvent=DMSO/CCl4 1/3

May 10 2023

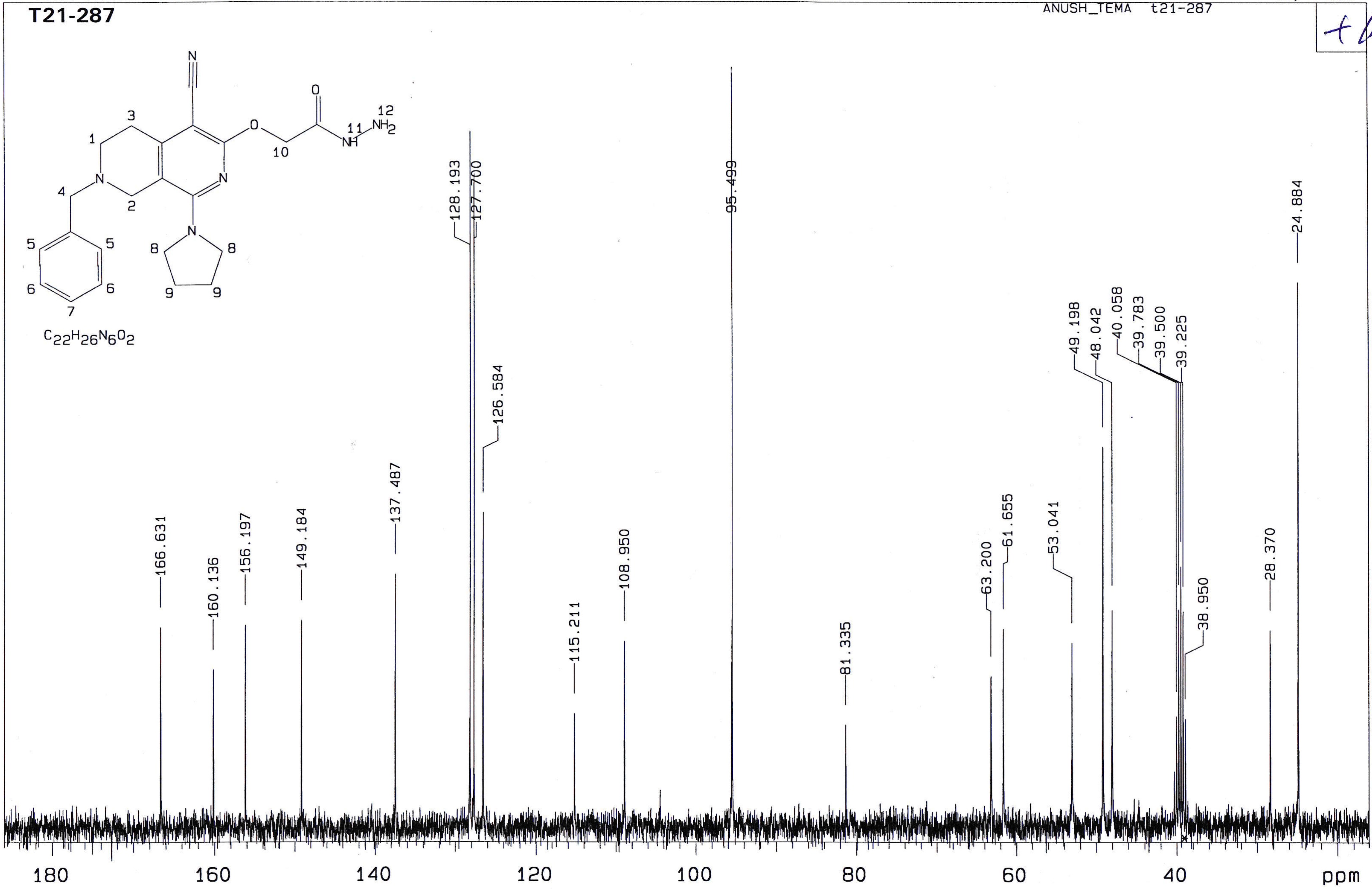

3f

Molecular Structure Research Centre, Yerevan, Armenia, Varian Mercury-300VX

H1 300.088 MHz, nt=16, np=32000, temp=30.0 C, lb=-0.2, solvent=DMSO/CCl4 1/3

May 2 2023

ANUSH_TEMA t21-279

T21-279

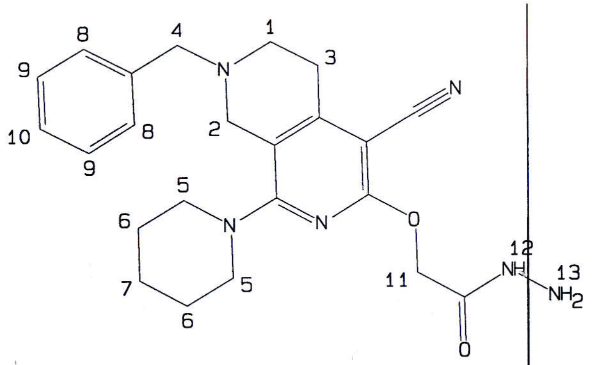

$C_{23}H_{28}N_6O_2$

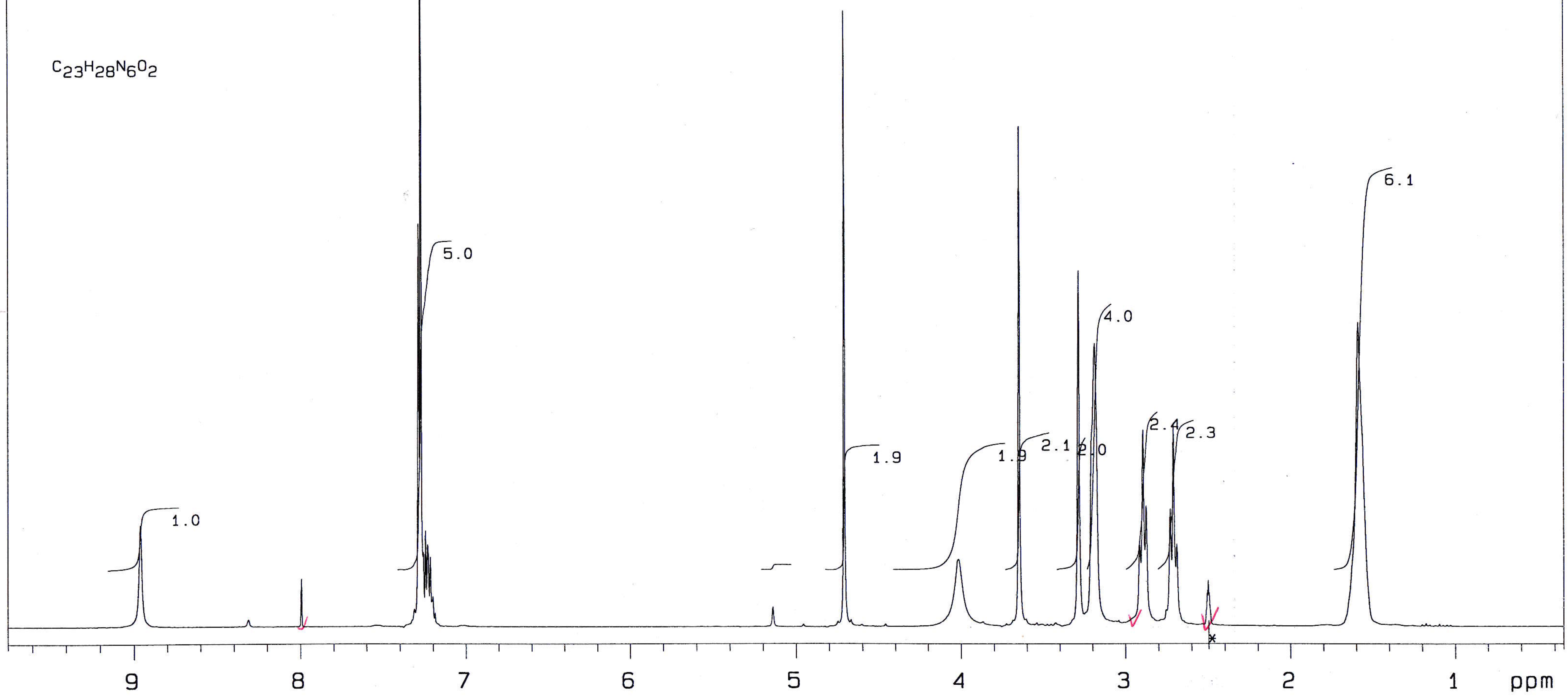

3f

Molecular Structure Research Centre, Yerevan, Armenia, Varian Mercury-300VX

C13 75.465 MHz, nt=272, np=19998, temp=30.0 C, lb=1.0, solvent=DMSO/CCl4 1/3

May 2 2023

ANUSH_TEMA t21-279

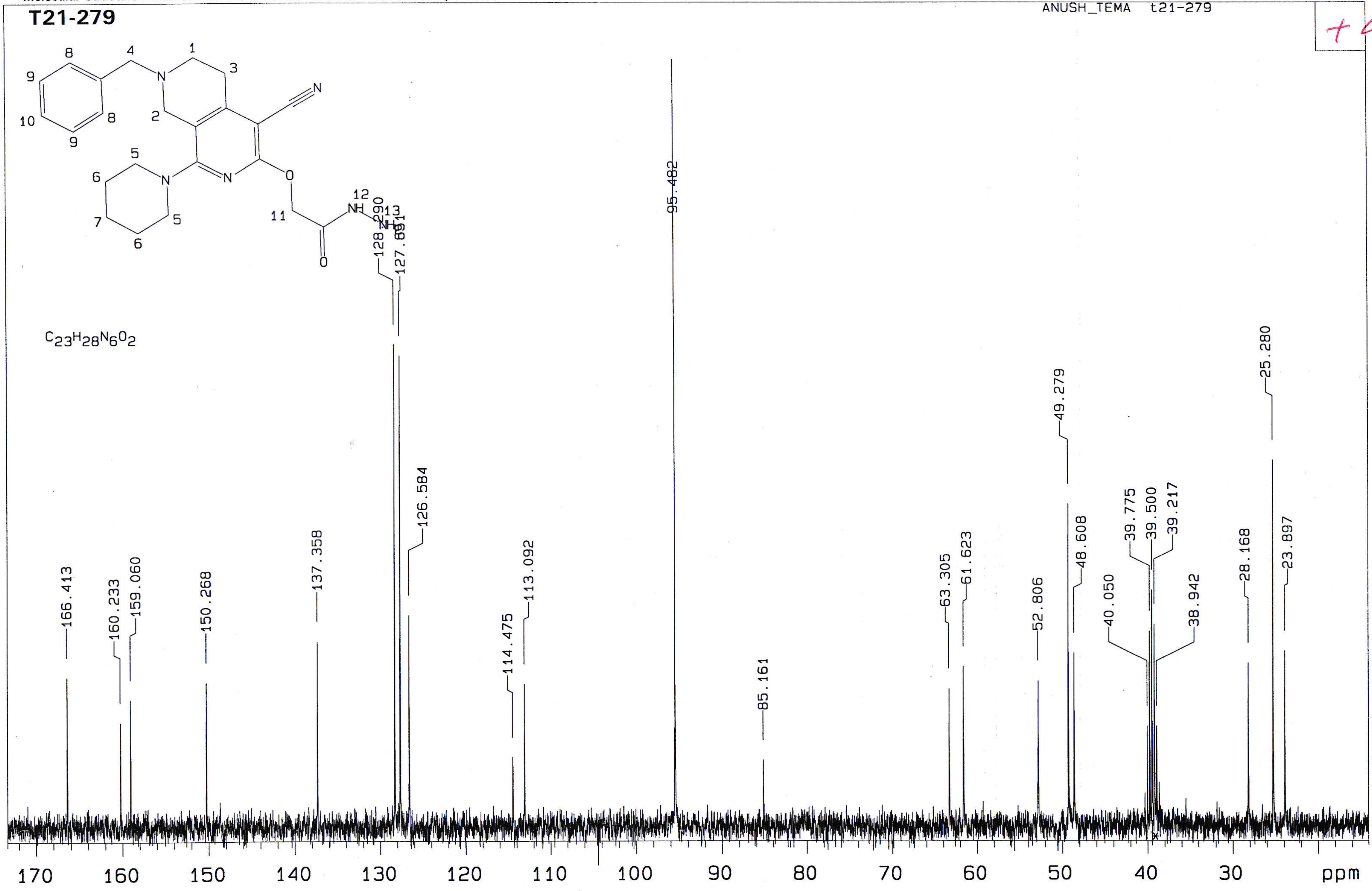

39

Molecular Structure Research Centre, Yerevan, Armenia, Varian Mercury-300VX

H1 300.088 MHz, nt = 16, np = 32000, temp = 30.0 C, lb = -0.2, solvent = DMSO/CCl4 1/3

Mar 21 2023

ANUSH_TEMA t21-245

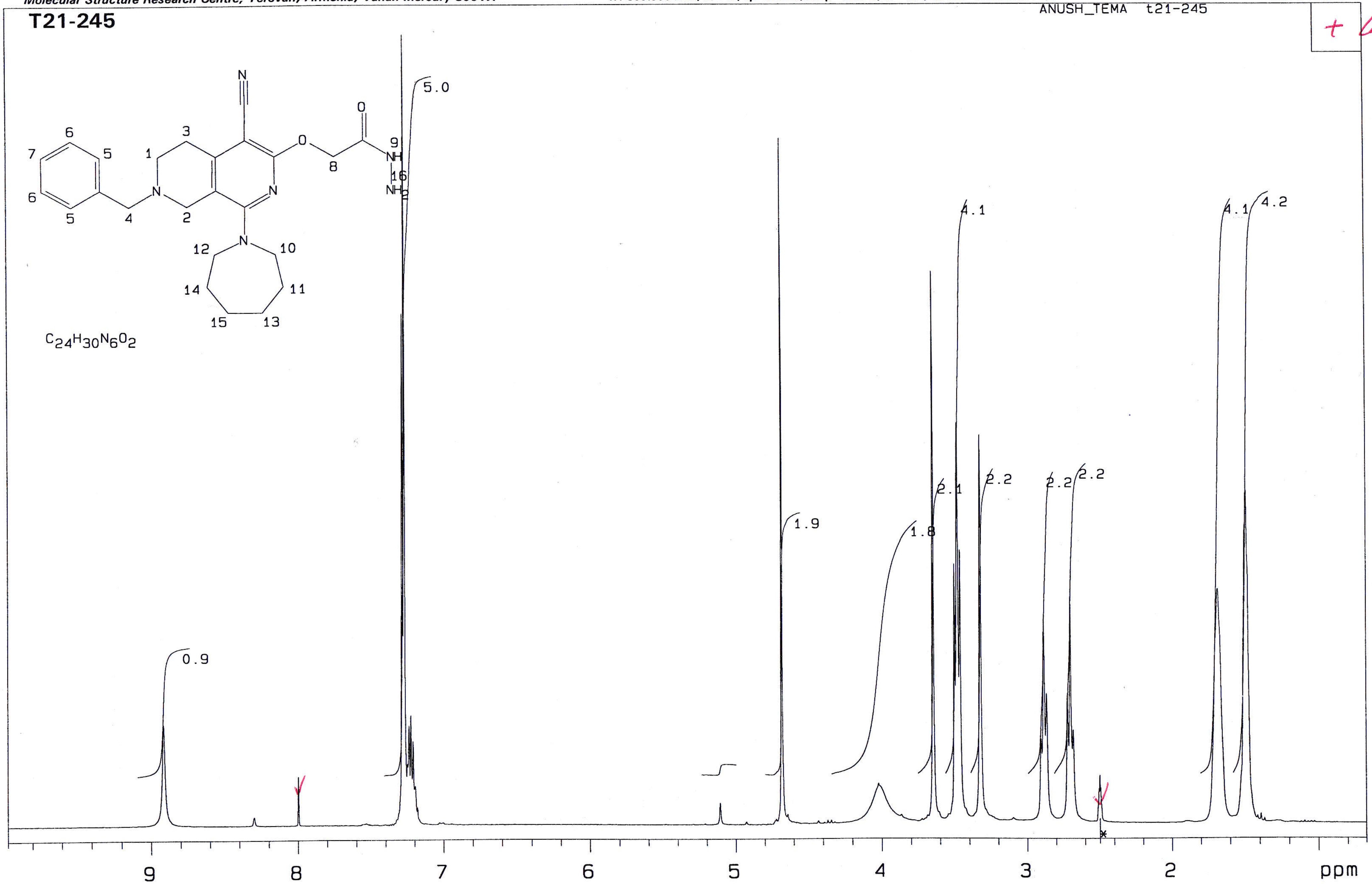

3g

Molecular Structure Research Centre, Yerevan, Armenia, Varian Mercury-300VX

C13 75.465 MHz, nt = 128, np = 19998, temp = 30.0 C, lb = 1.0, solvent = DMSO/CCl4 1/3

Mar 21 2023

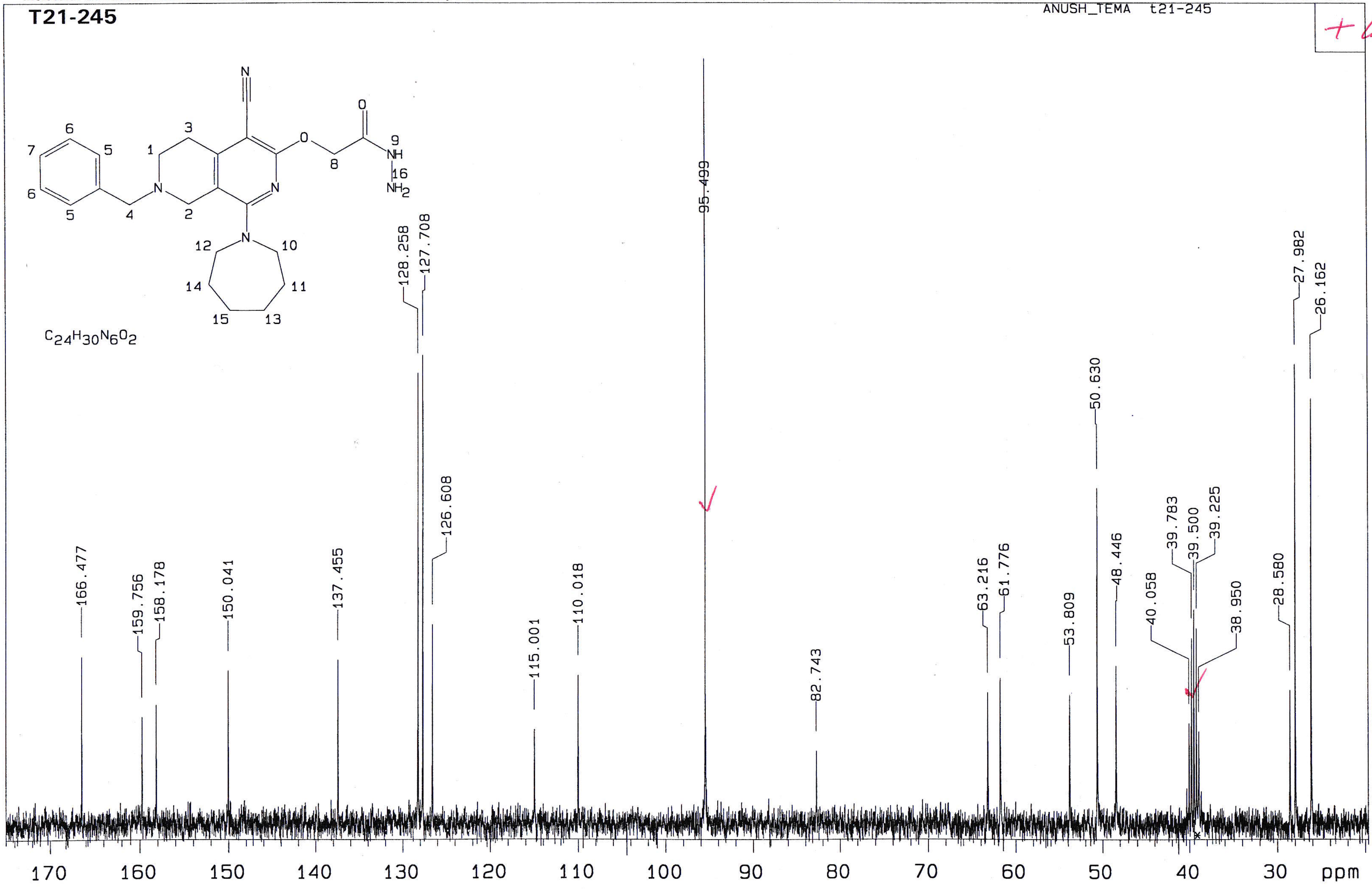

3h

Molecular Structure Research Centre, Yerevan, Armenia, Varian Mercury-300VX

H1 300.088 MHz, nt = 16, np = 32000, temp = 30.0 C, lb = -0.2, solvent = DMSO/CCl4 1/3

Apr 28 2023

ANUSH_TEMA t21-272-1

T21-272-1

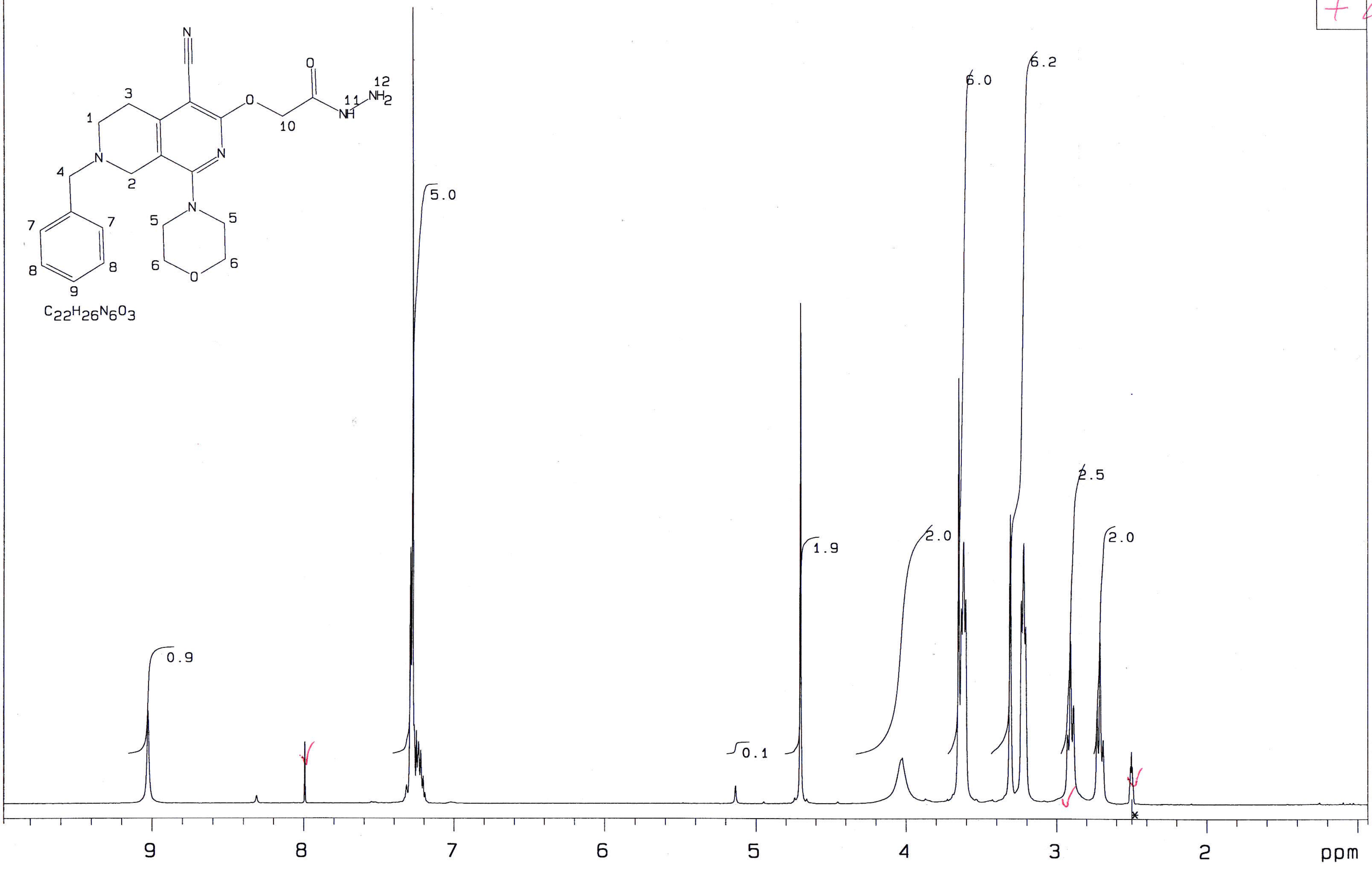

3h

Molecular Structure Research Centre, Yerevan, Armenia, Varian Mercury-300VX

C13 75.465 MHz, nt=352, np=19998, temp=30.0 C, lb=2.0, solvent=DMSO/CCl4 1/3

Apr 28 2023

ANUSH_TEMA t21-272-1

T21-272-1

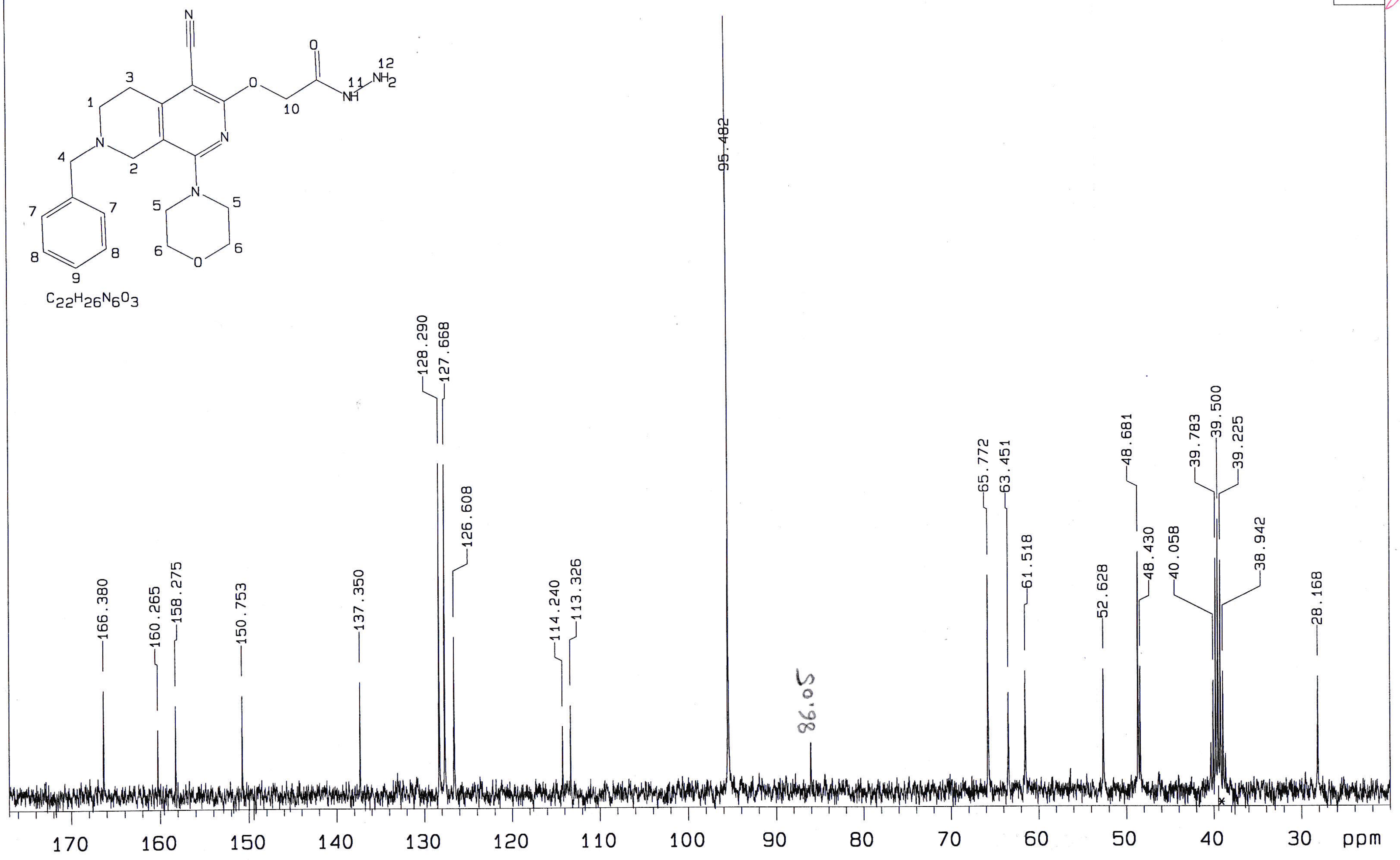

4a

Molecular Structure Research Centre, Yerevan, Armenia, Varian Mercury-300VX

H1 300.088 MHz, nt = 16, np = 32000, temp = 30.0 C, lb = -0.2, solvent = DMSO/CCl4 1/3

Feb 15 2022

ANUSH_TEMA t21-094

T21-094

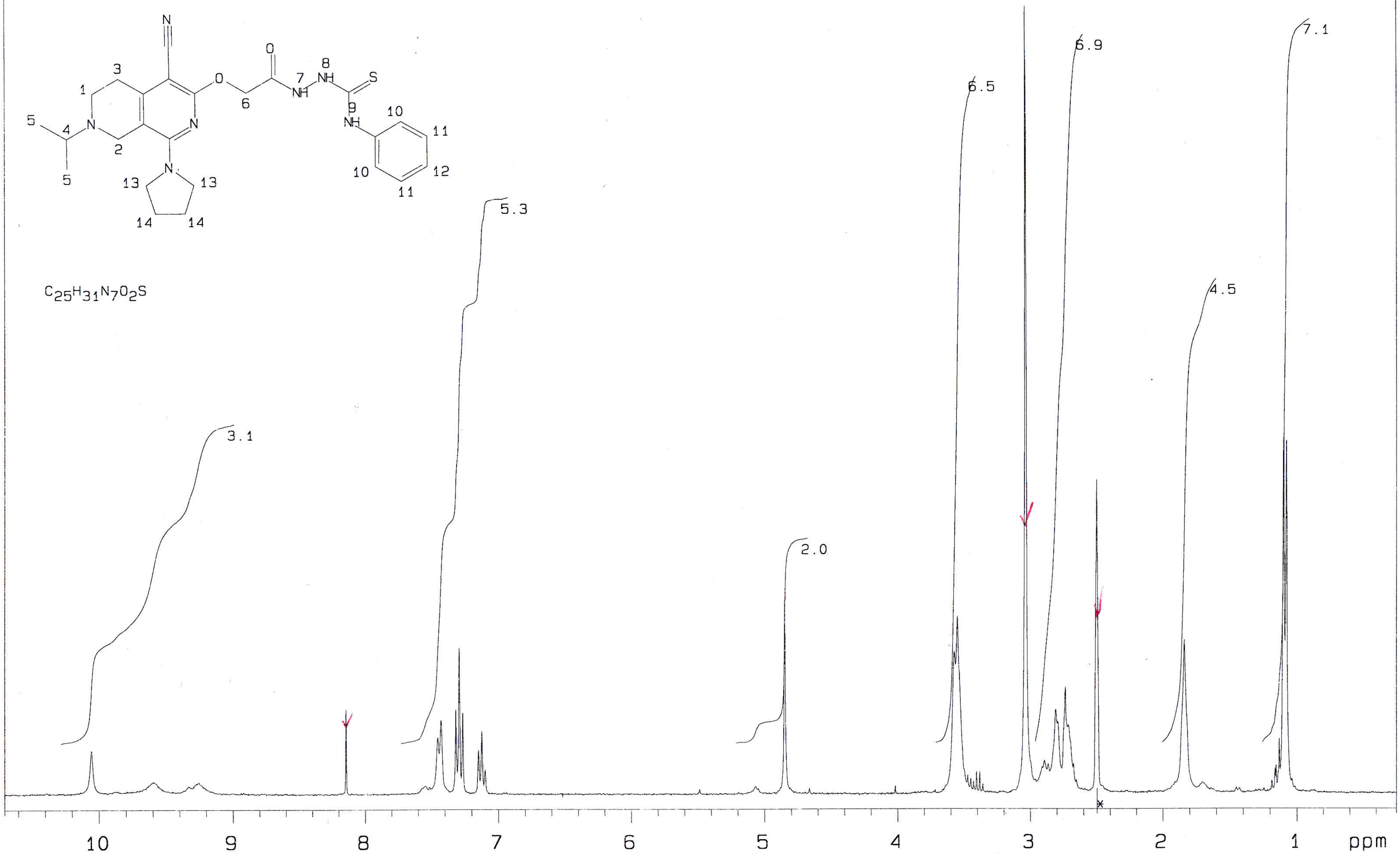

4в

Molecular Structure Research Centre, Yerevan, Armenia, Varian Mercury-300VX H1 300.088 MHz, nt = 16, np = 32000, temp = 30.0 C, lb = -0.2, solvent = DMSO/CCl4 1/3 Apr 12 2022

ANUSH_TEMA t21-152

T21-152

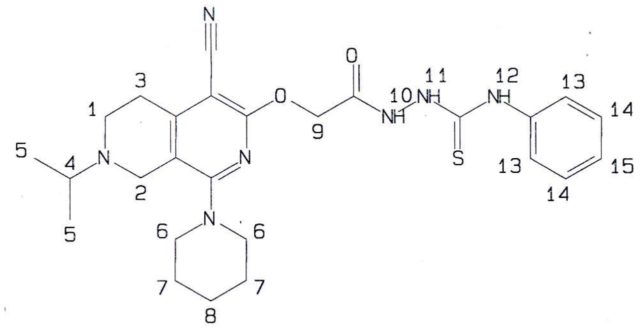

$C_{26}H_{33}N_7O_2S$

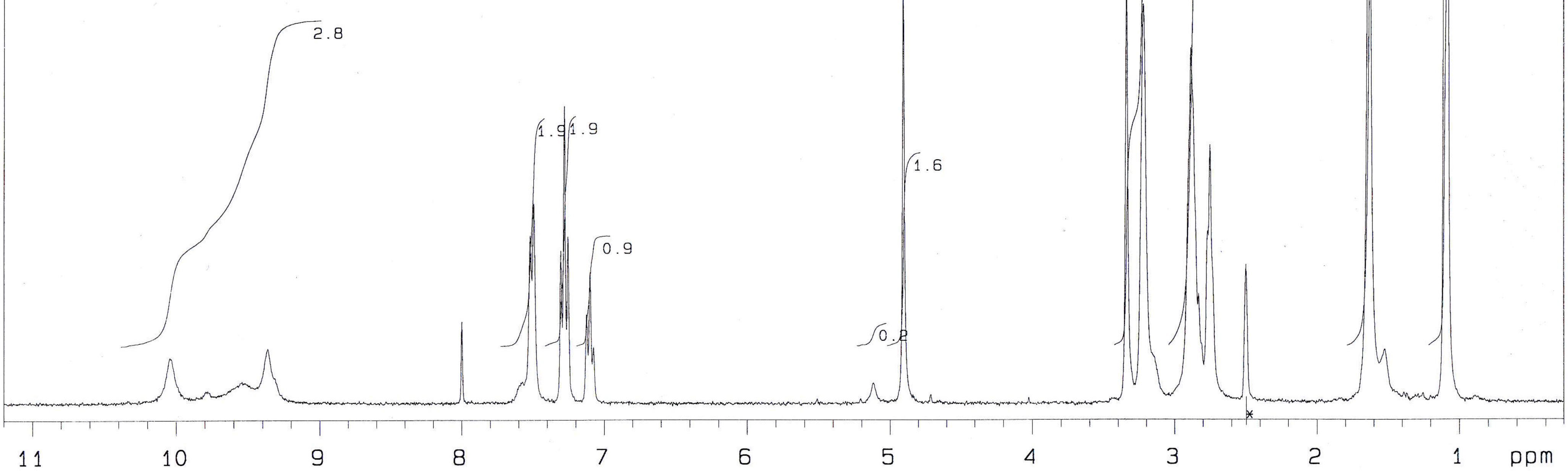

46

Molecular Structure Research Centre, Yerevan, Armenia, Varian Mercury-300VX

C13 75.465 MHz, nt = 752, np = 19998, temp = 30.0 C, lb = 1.0, solvent = DMSO/CCl4 1/3

Apr 12 2022

ANUSH_TEMA t21-152

T21-152

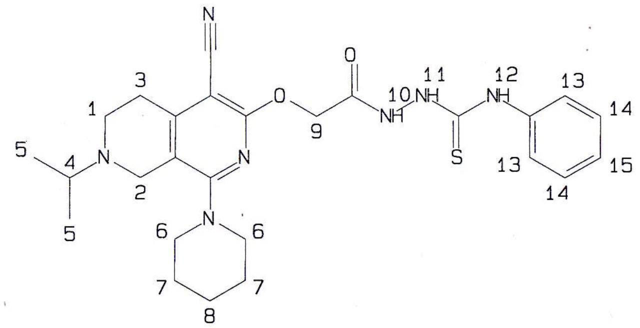

$C_{26}H_{33}N_7O_2S$

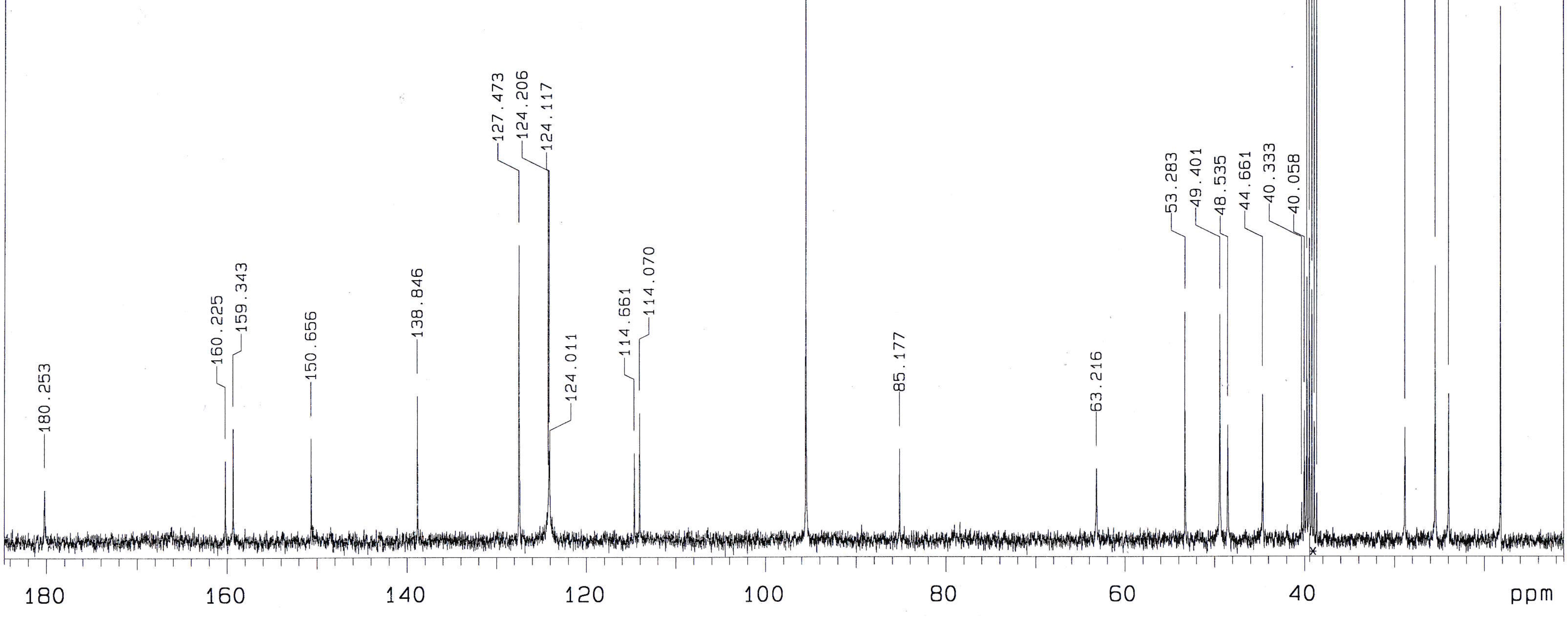

4c

Molecular Structure Research Centre, Yerevan, Armenia, Varian Mercury-300VX   H1 300.088 MHz, nt = 16, np = 32000, temp = 30.0 C, lb = -0.2, solvent = DMSO/CCl4 1/3   Dec 2 2021

ANUSH_TEMA t21-044

T21-044

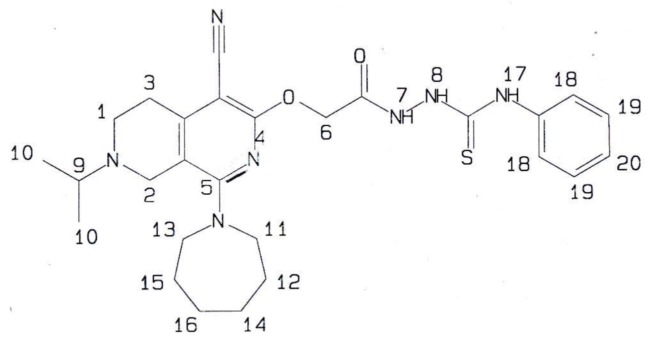

$C_{27}H_{37}N_7O_2S$

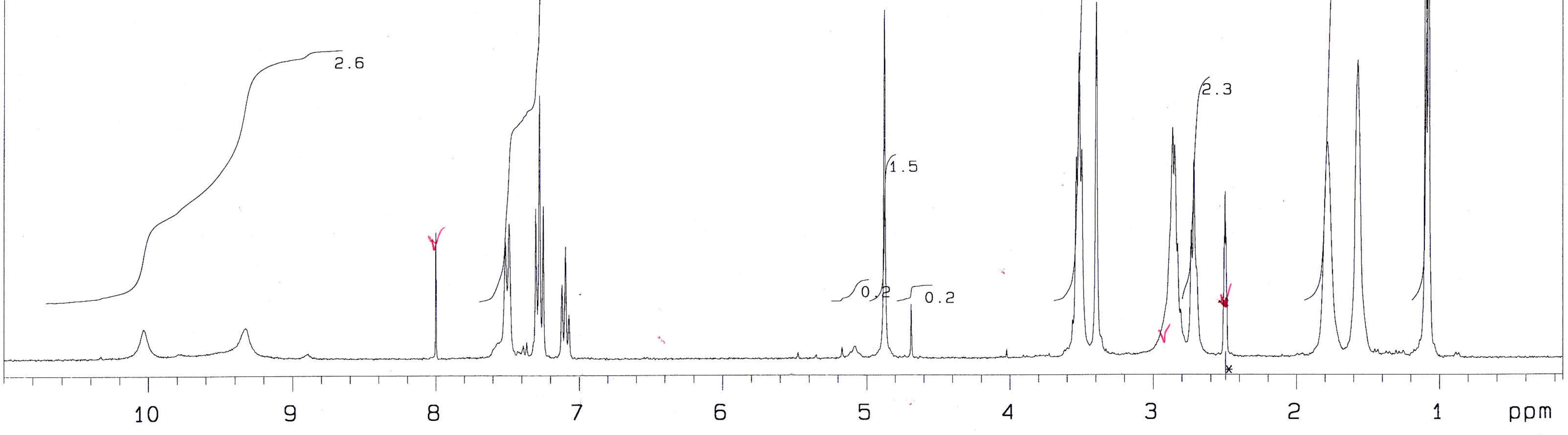

4c

Molecular Structure Research Centre, Yerevan, Armenia, Varian Mercury-300VX

C13 75.465 MHz, nt = 1184, np = 19998, temp = 30.0 C, lb = 1.0, solvent = DMSO/CCl4 1/3

Dec 2 2021

T21-044

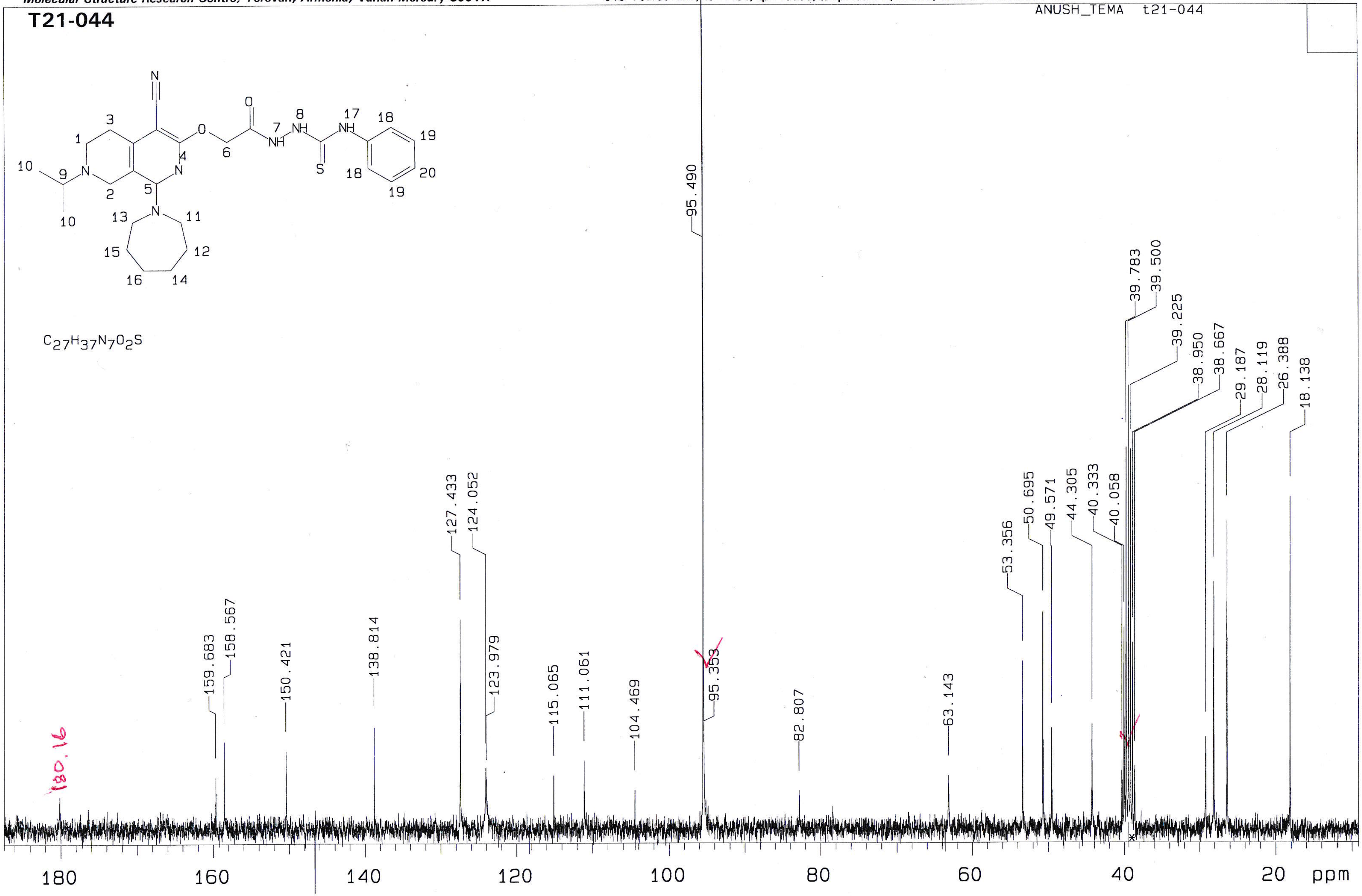

4d

Molecular Structure Research Centre, Yerevan, Armenia, Varian Mercury-300VX

H1 300.088 MHz, nt = 16, np = 32000, temp = 30.0 C, lb = -0.2, solvent = DMSO/CCl4 1/3

Apr 12 2023

ANUSH_TEMA t21-174-1

T21-174-1

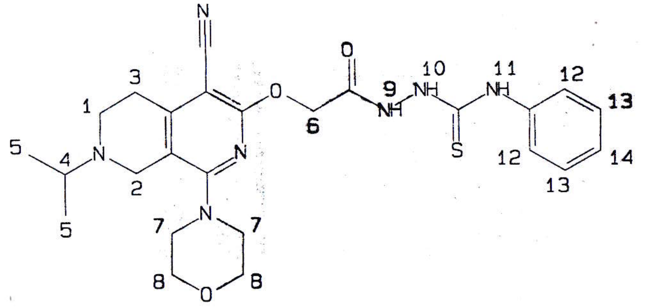

$C_{25}H_{31}N_7O_3S$

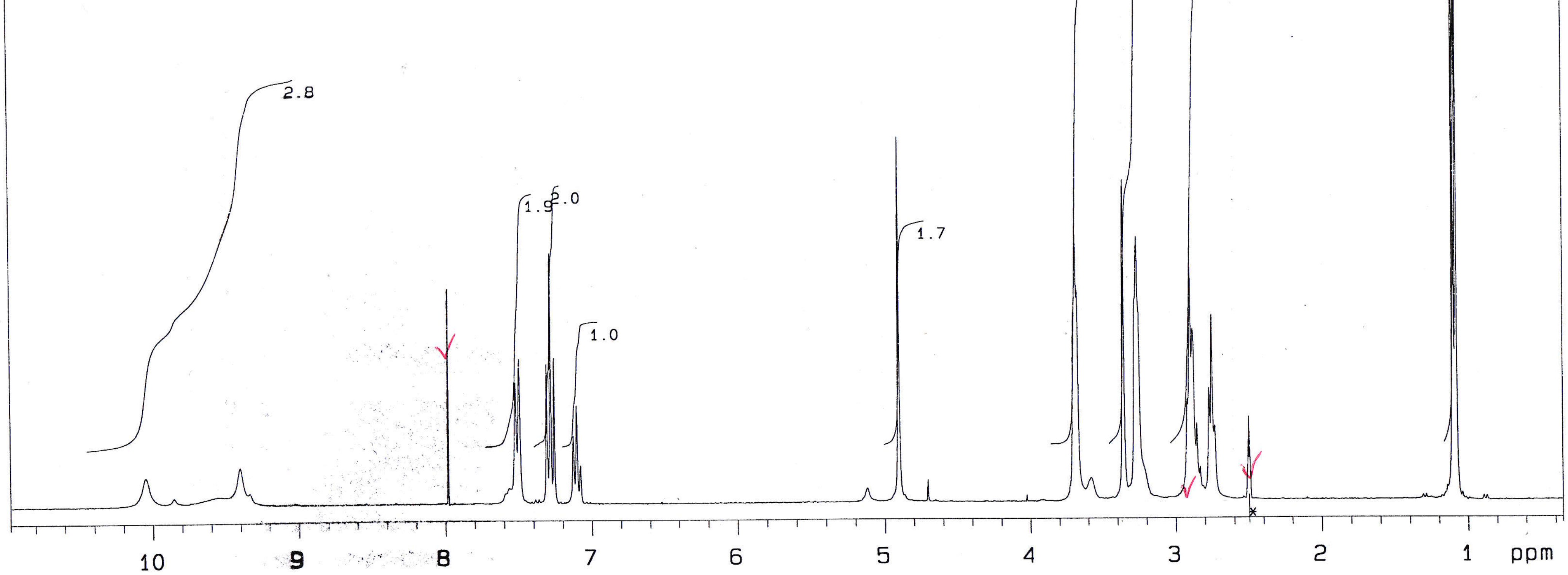

4d

Molecular Structure Research Centre, Yerevan, Armenia, Varian Mercury-300VX   C13 75.465 MHz, nt=608, np=19998, temp=30.0 C, lb=1.0, solvent=DMSO/CCl4 1/3   Apr 12 2023

ANUSH_TEMA t21-174-1

T21-174-1

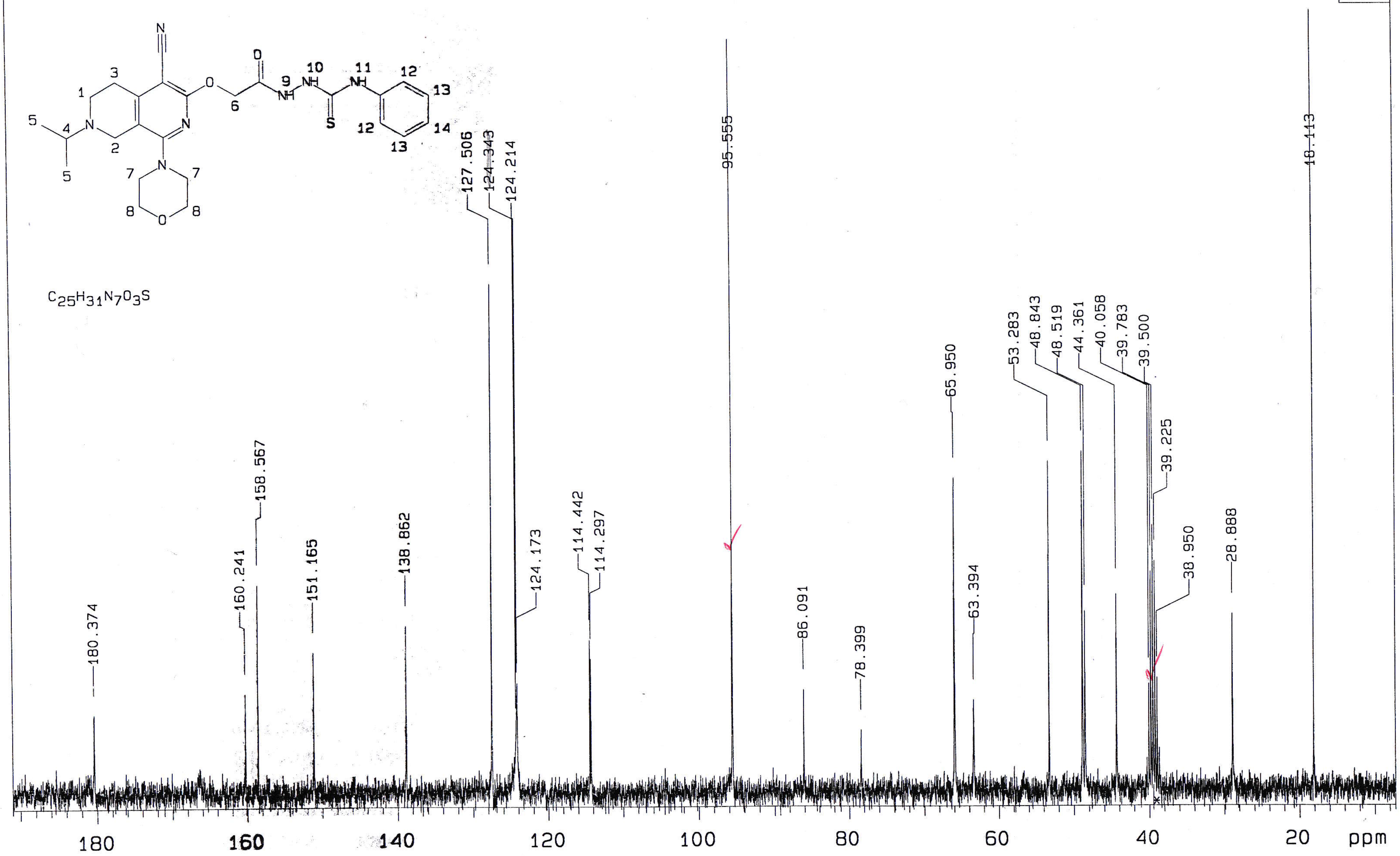

4e

Molecular Structure Research Centre, Yerevan, Armenia, Varian Mercury-300VX H1 300.088 MHz, nt = 16, np = 32000, temp = 30.0 C, lb = -0.2, solvent = DMSO/CCl4 1/3 May 18 2023

NOCI_23 t21-291

+ [illegible]

T21-291

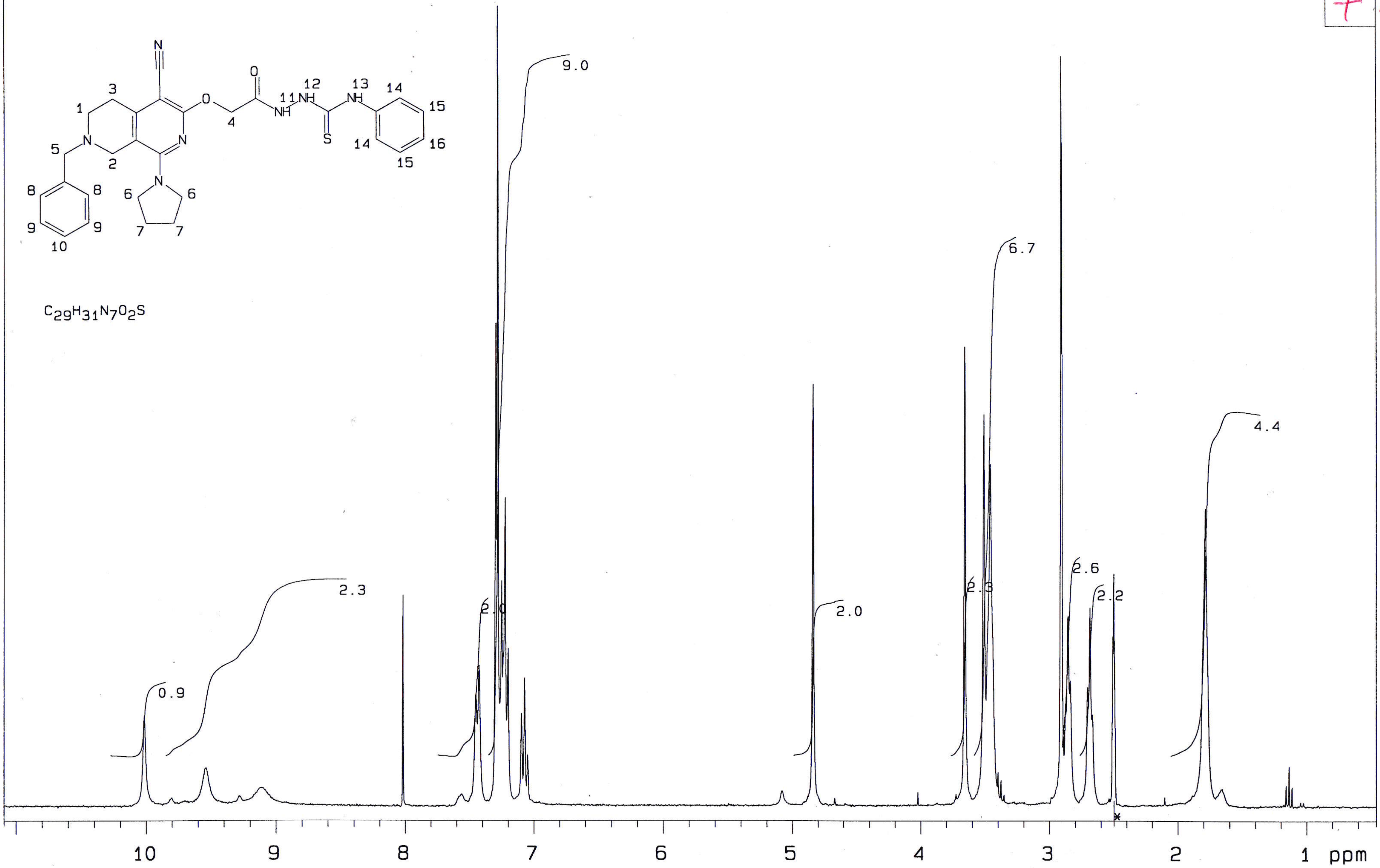

4e

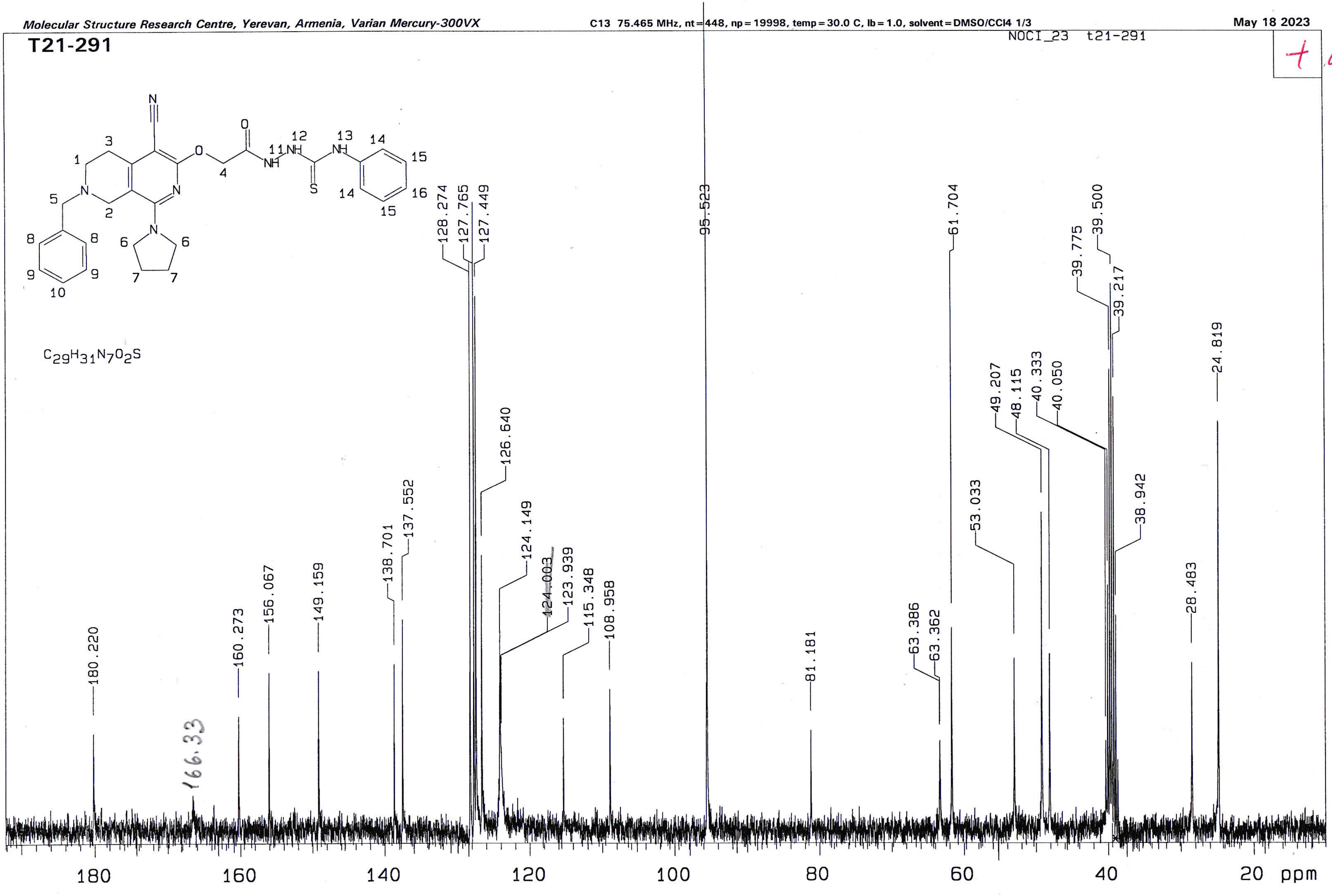

Molecular Structure Research Centre, Yerevan, Armenia, Varian Mercury-300VX
C13 75.465 MHz, nt=448, np=19998, temp=30.0 C, lb=1.0, solvent=DMSO/CCl4 1/3
May 18 2023
T21-291
NOCI_23 t21-291
$C_{29}H_{31}N_7O_2S$
180.220
166.33
160.273
156.067
149.159
138.701
137.552
128.274
127.765
127.449
126.640
124.149
124.003
123.939
115.348
108.958
95.523
81.181
63.386
63.362
61.704
53.033
49.207
48.115
40.333
40.050
39.775
39.500
39.217
38.942
28.483
24.819
ppm

4f

Molecular Structure Research Centre, Yerevan, Armenia, Varian Mercury-300VX

H1 300.088 MHz, nt=16, np=32000, temp=30.0 C, lb=-0.2, solvent=DMSO/CCl4 1/3

May 10 2023

ANUSH_TEMA t21-284

T21-284

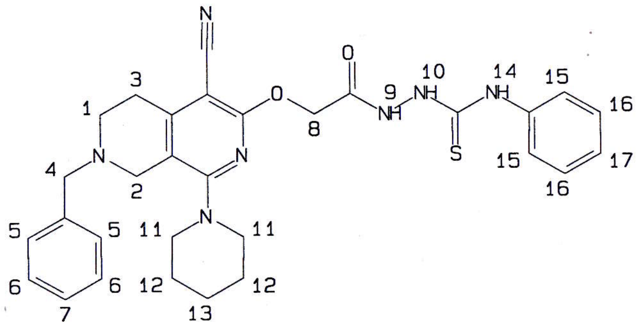

$C_{30}H_{33}N_7O_2S$

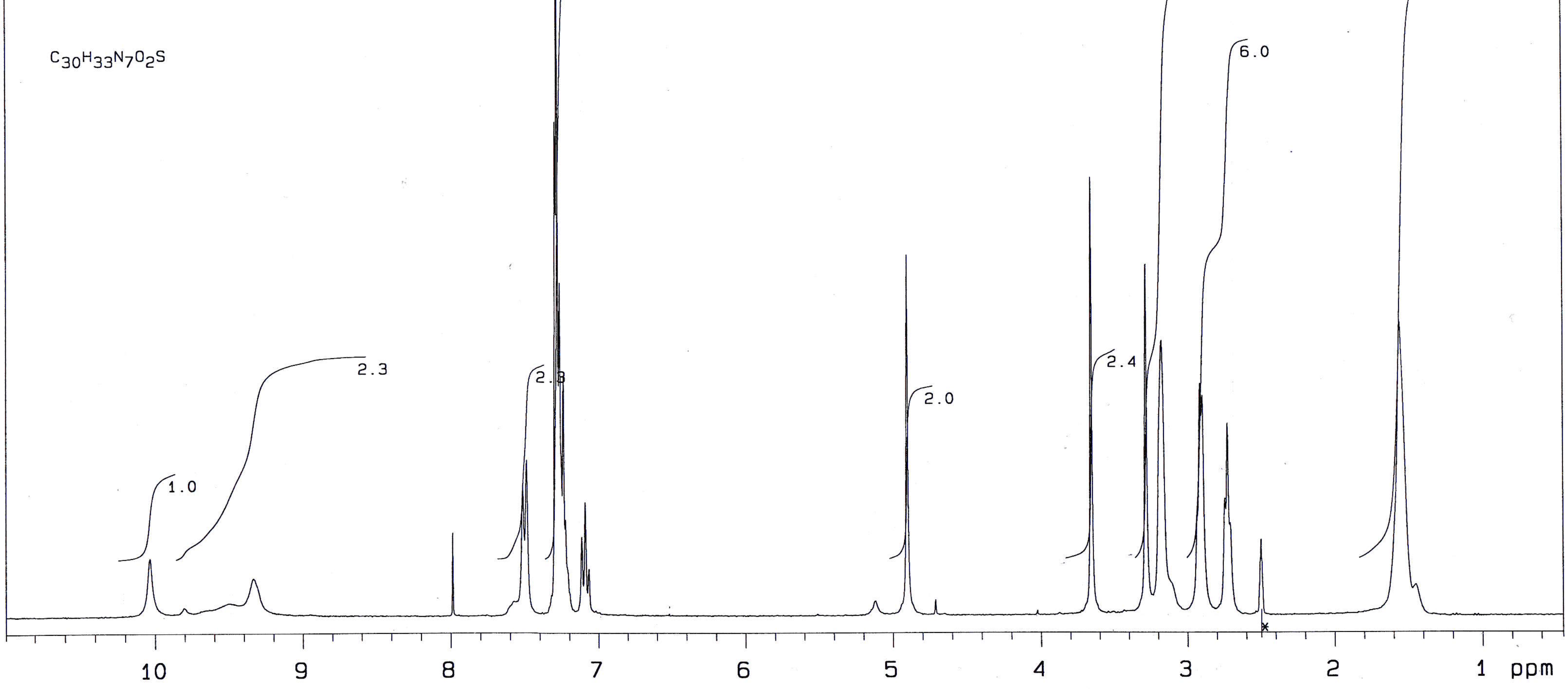

4f

Molecular Structure Research Centre, Yerevan, Armenia, Varian Mercury-300VX

C13 75.465 MHz, nt = 1136, np = 19998, temp = 30.0 C, lb = 1.0, solvent = DMSO/CCl4 1/3

May 10 2023

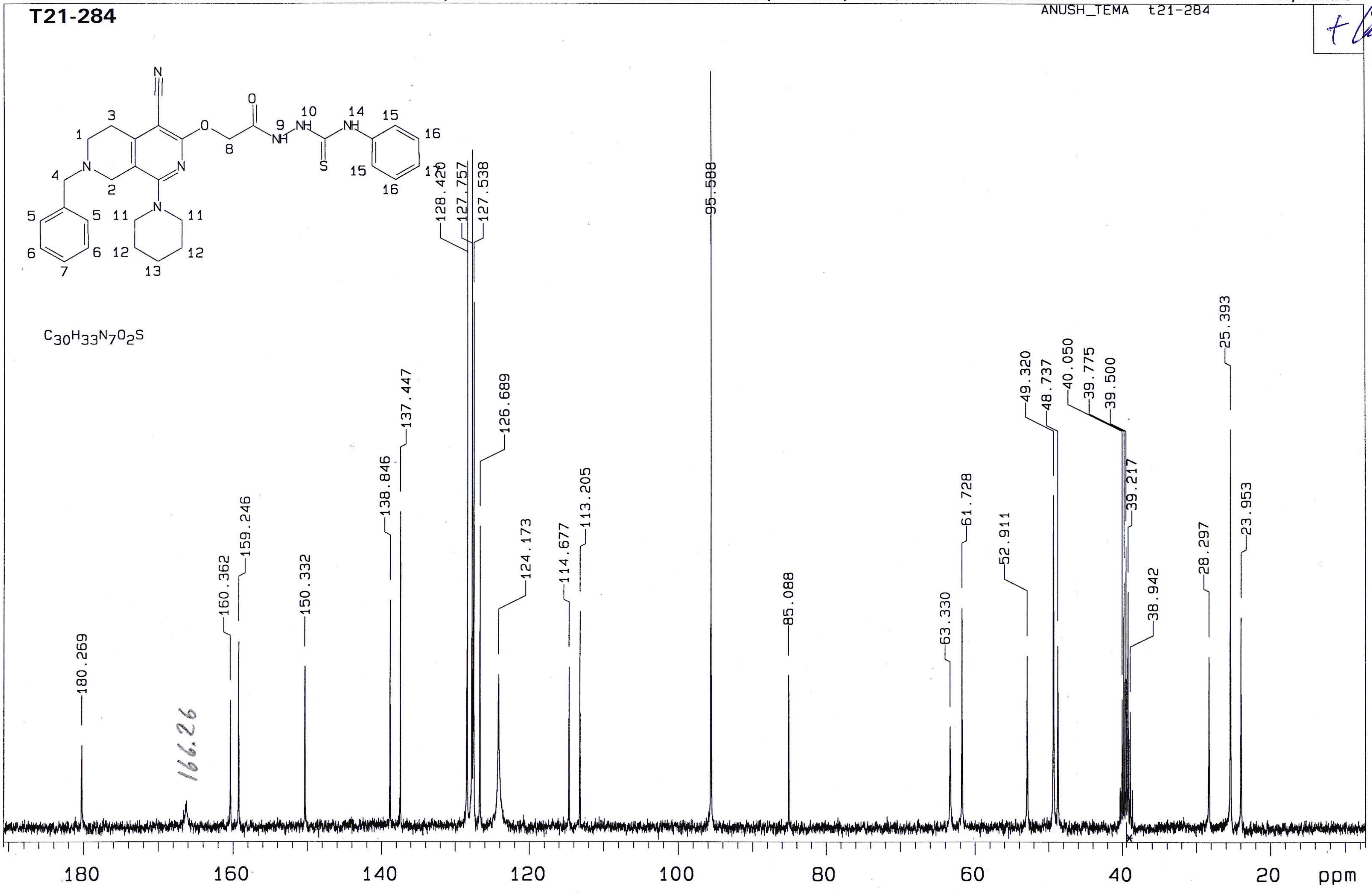

4g

Molecular Structure Research Centre, Yerevan, Armenia, Varian Mercury-300VX

H1 300.088 MHz, nt = 16, np = 32000, temp = 30.0 C, lb = -0.2, solvent = DMSO/CCl4 1/3

Mar 28 2023

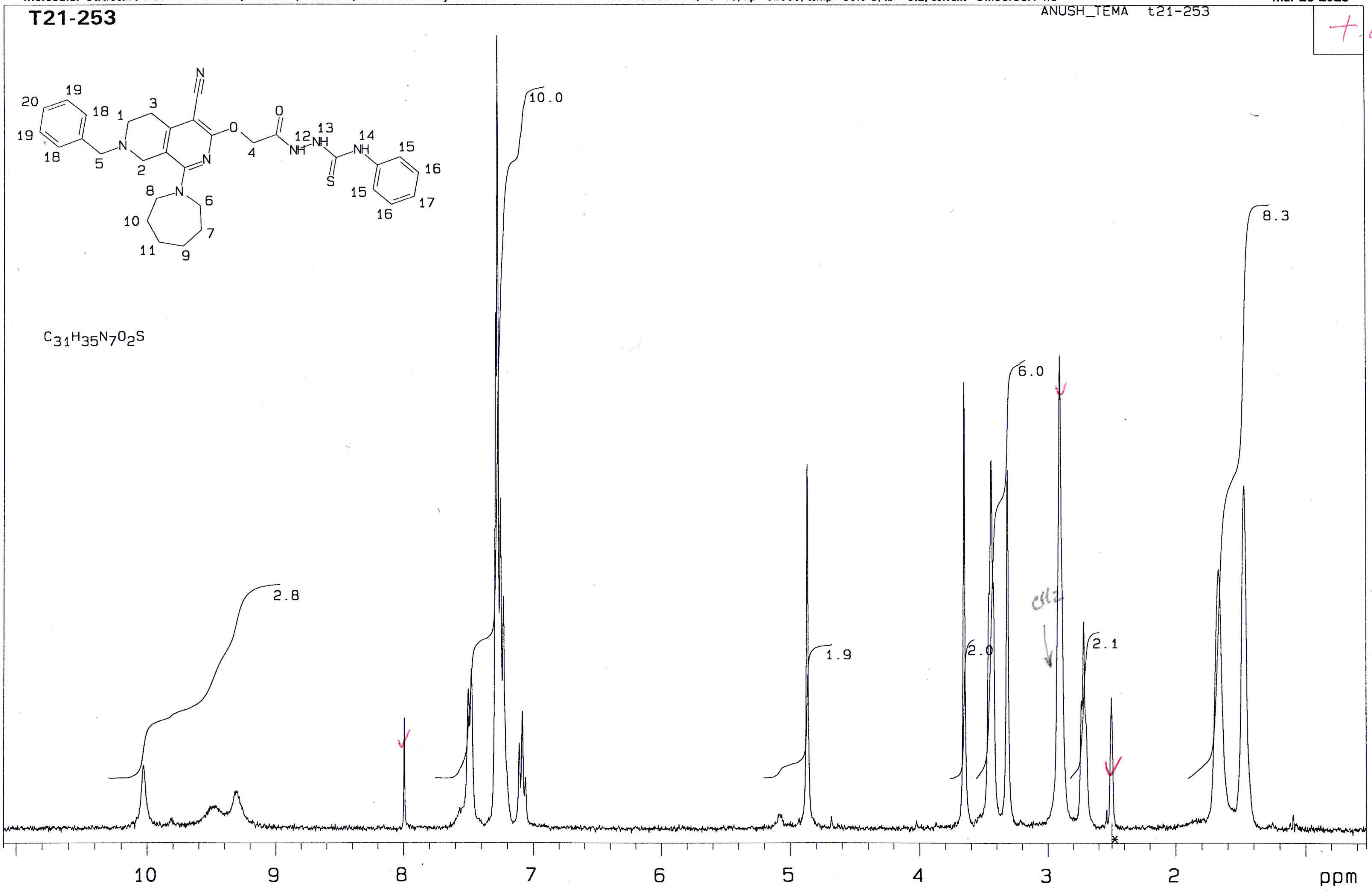

Molecular Structure Research Centre, Yerevan, Armenia, Varian Mercury-300VX   C13 75.465 MHz, nt=848, np=19998, temp=30.0 C, lb=1.0, solvent=DMSO/CCl4 1/3   **Mar 28 2023**

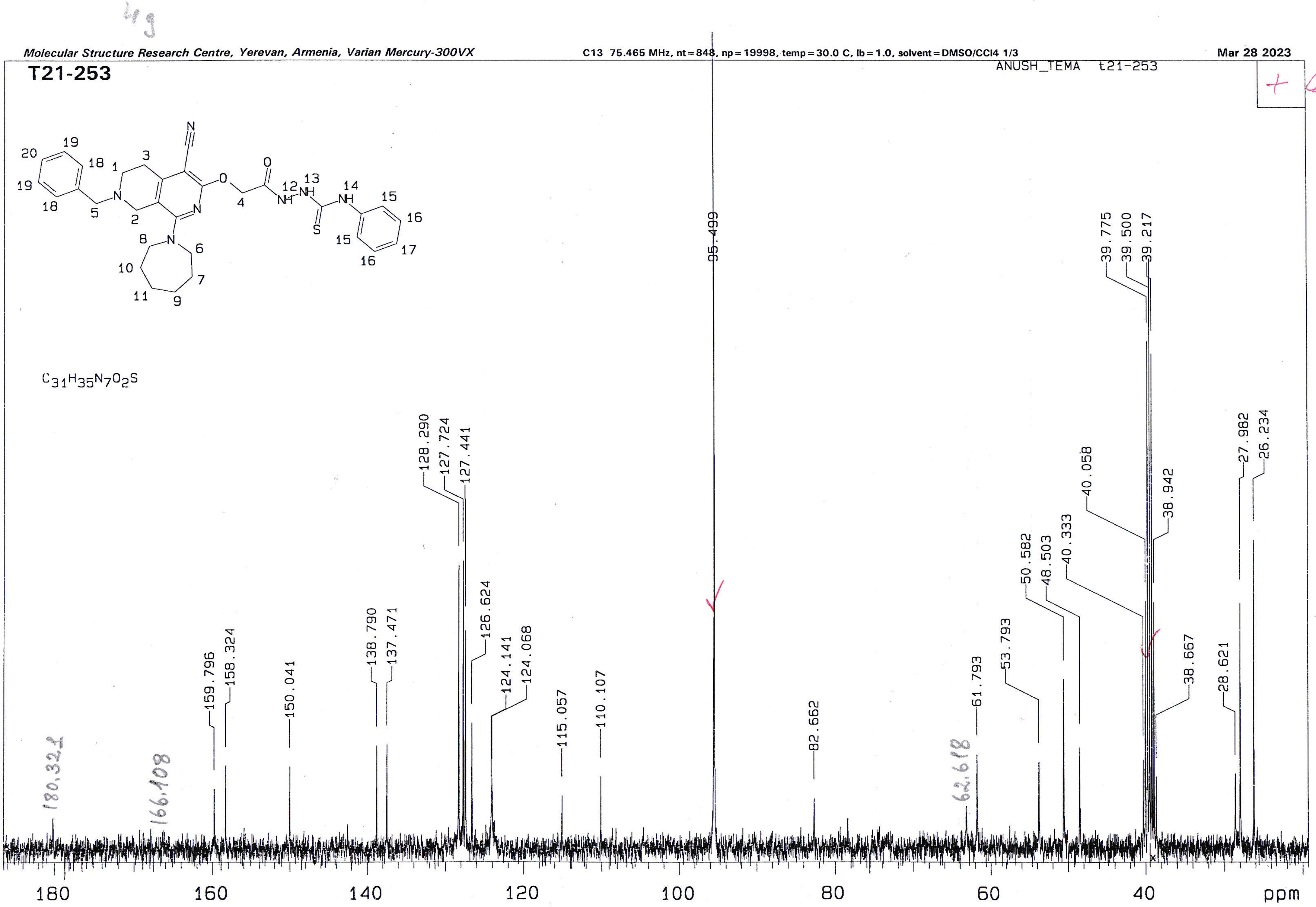

4h

Molecular Structure Research Centre, Yerevan, Armenia, Varian Mercury-300VX H1 300.088 MHz, nt=16, np=32000, temp=30.0 C, lb=-0.2, solvent=DMSO/CCl4 1/3 **Nov 15 2023**

ANUSH_TEMA t21-283-1

T21-283-1

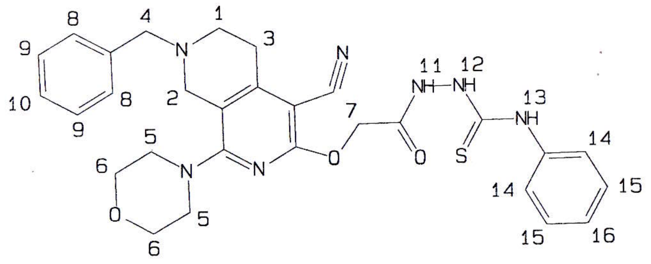

$C_{29}H_{31}N_7O_3S$

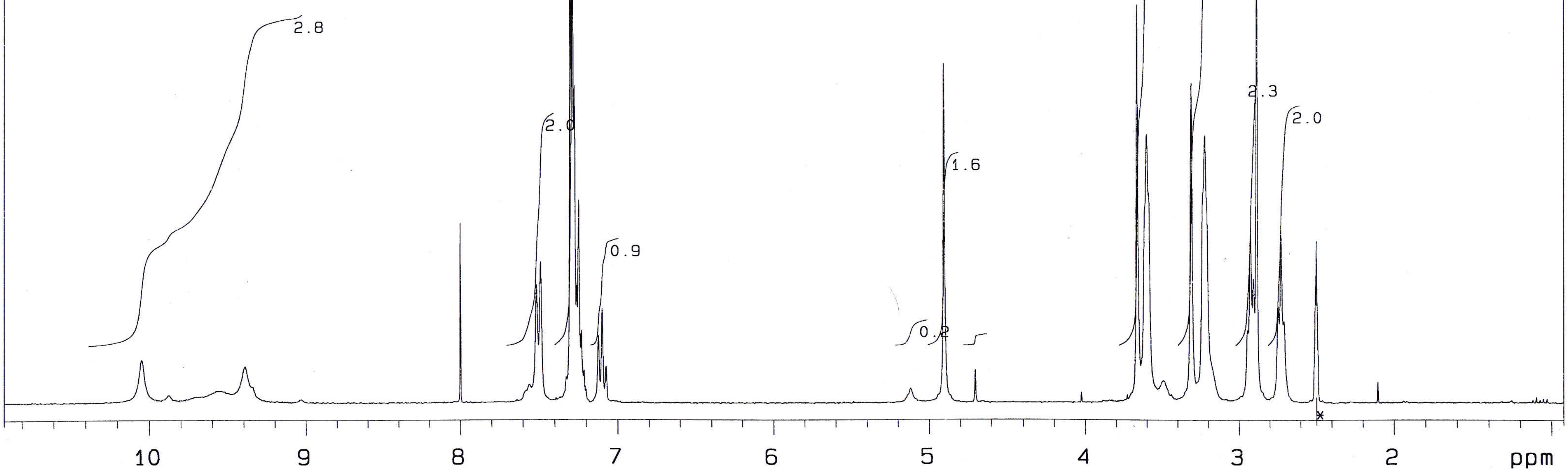

4h

Molecular Structure Research Centre, Yerevan, Armenia, Varian Mercury-300VX

C13 75.465 MHz, nt=4368, np=19998, temp=30.0 C, lb=1.0, solvent=DMSO/CCl4 1/3

Nov 15 2023

ANUSH_TEMA t21-283-1

T21-283-1

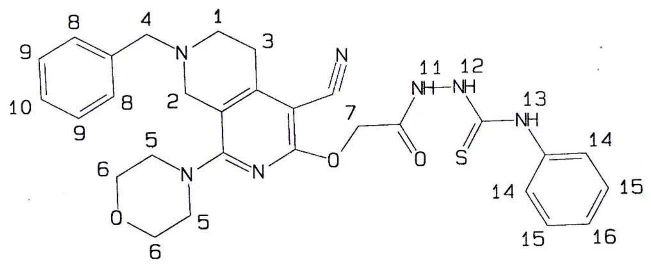

$C_{29}H_{31}N_7O_3S$

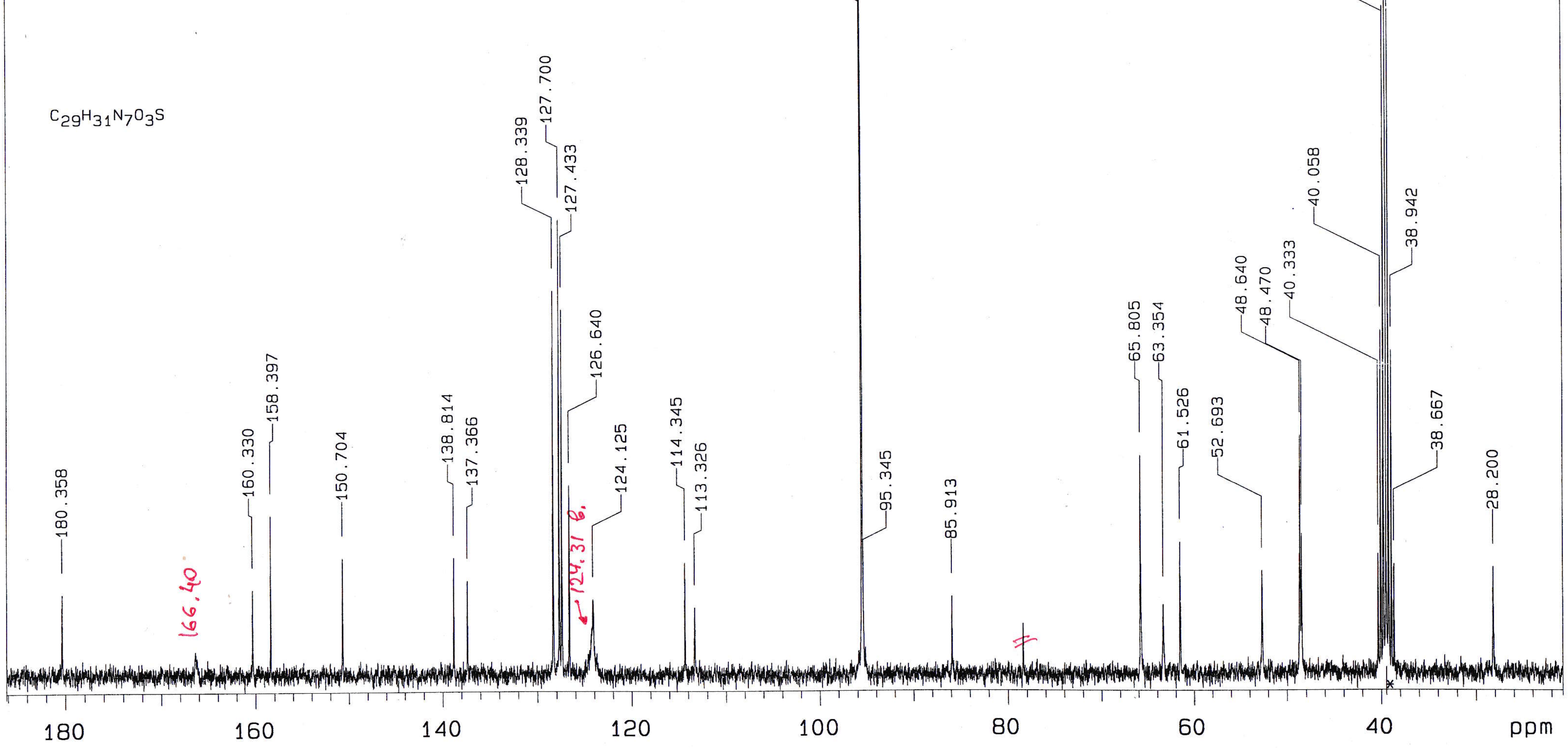

5a

Molecular Structure Research Centre, Yerevan, Armenia, Varian Mercury-300VX

H1 300.088 MHz, nt = 16, np = 32000, temp = 30.0 C, lb = -0.2, solvent = DMSO/CCl4 1/3

Jun 7 2021

S12-024 T21 - 099

ANUSH_TEMA s12-024

$C_{25}H_{29}N_7OS$

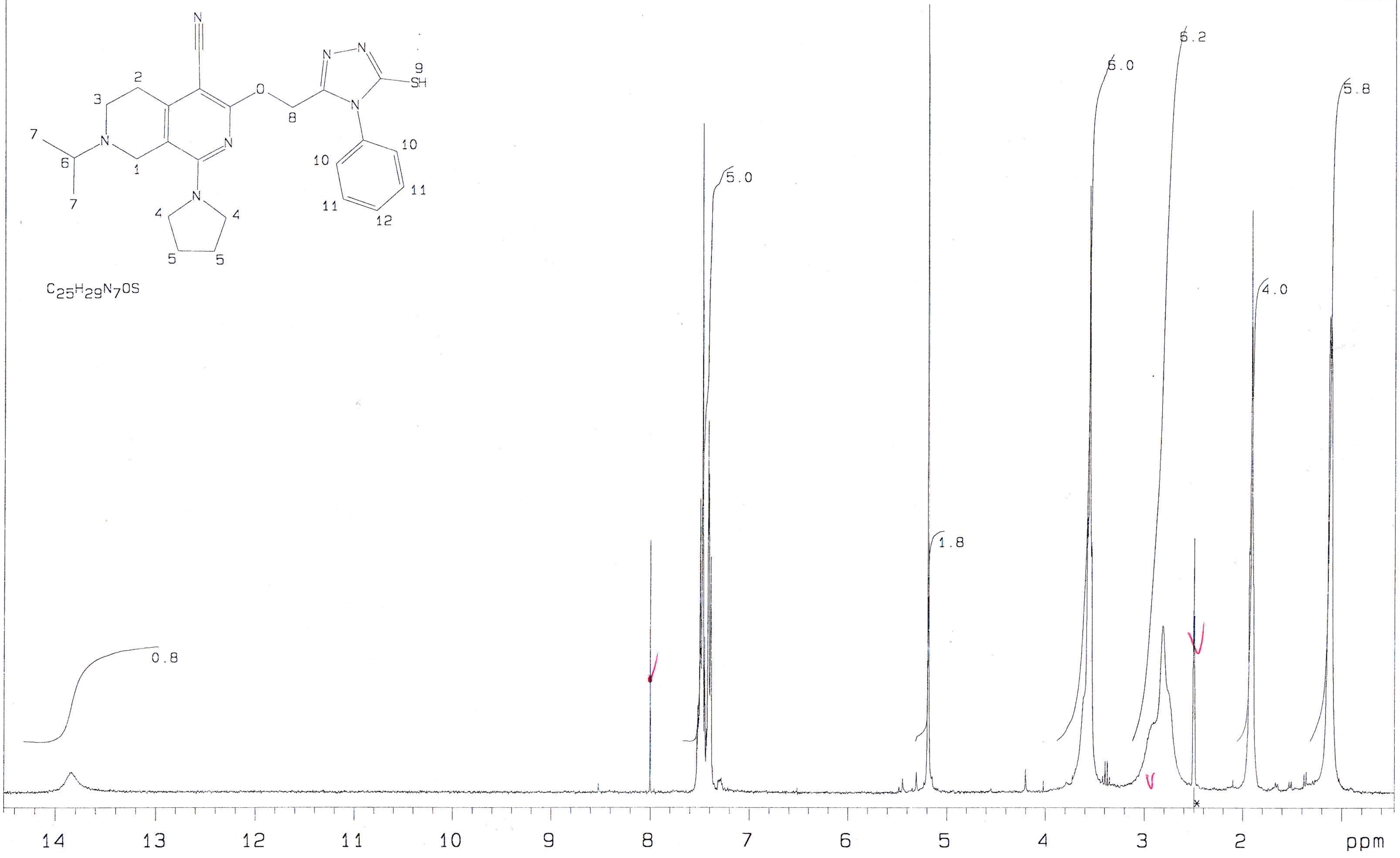

5a

Molecular Structure Research Centre, Yerevan, Armenia, Varian Mercury-300VX

C13 75.465 MHz, nt = 1200, np = 19998, temp = 30.0 C, lb = 1.0, solvent = DMSO/CCl4 1/3

Jun 7 2021

ANUSH_TEMA s12-024

S12-024 T21-099

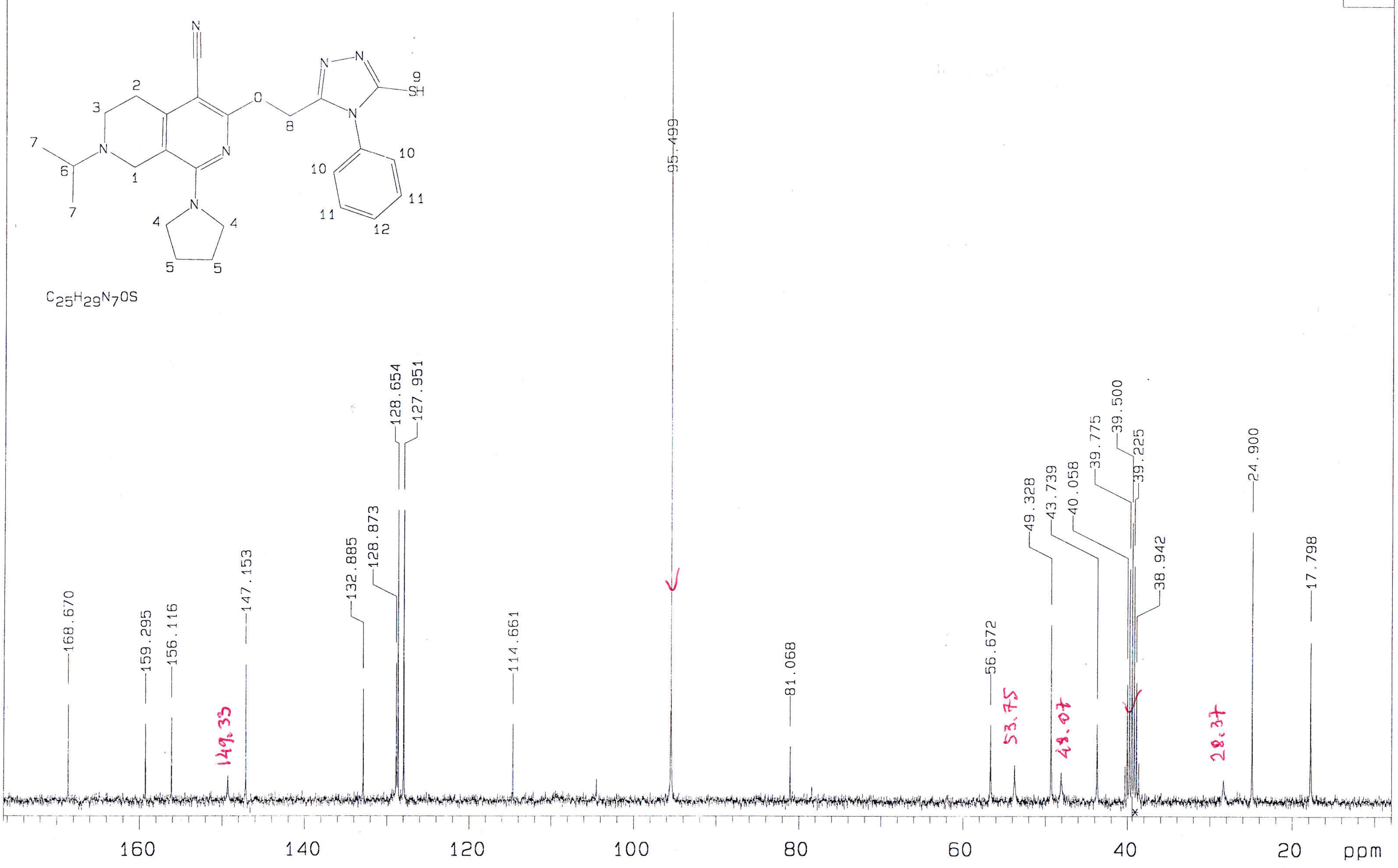

Molecular Structure Research Centre, Yerevan, Armenia, Varian Mercury-300VX

H1 300.088 MHz, nt = 16, np = 32000, temp = 30.0 C, lb = -0.2, solvent = DMSO/CCl4 1/3

Apr 21 2022

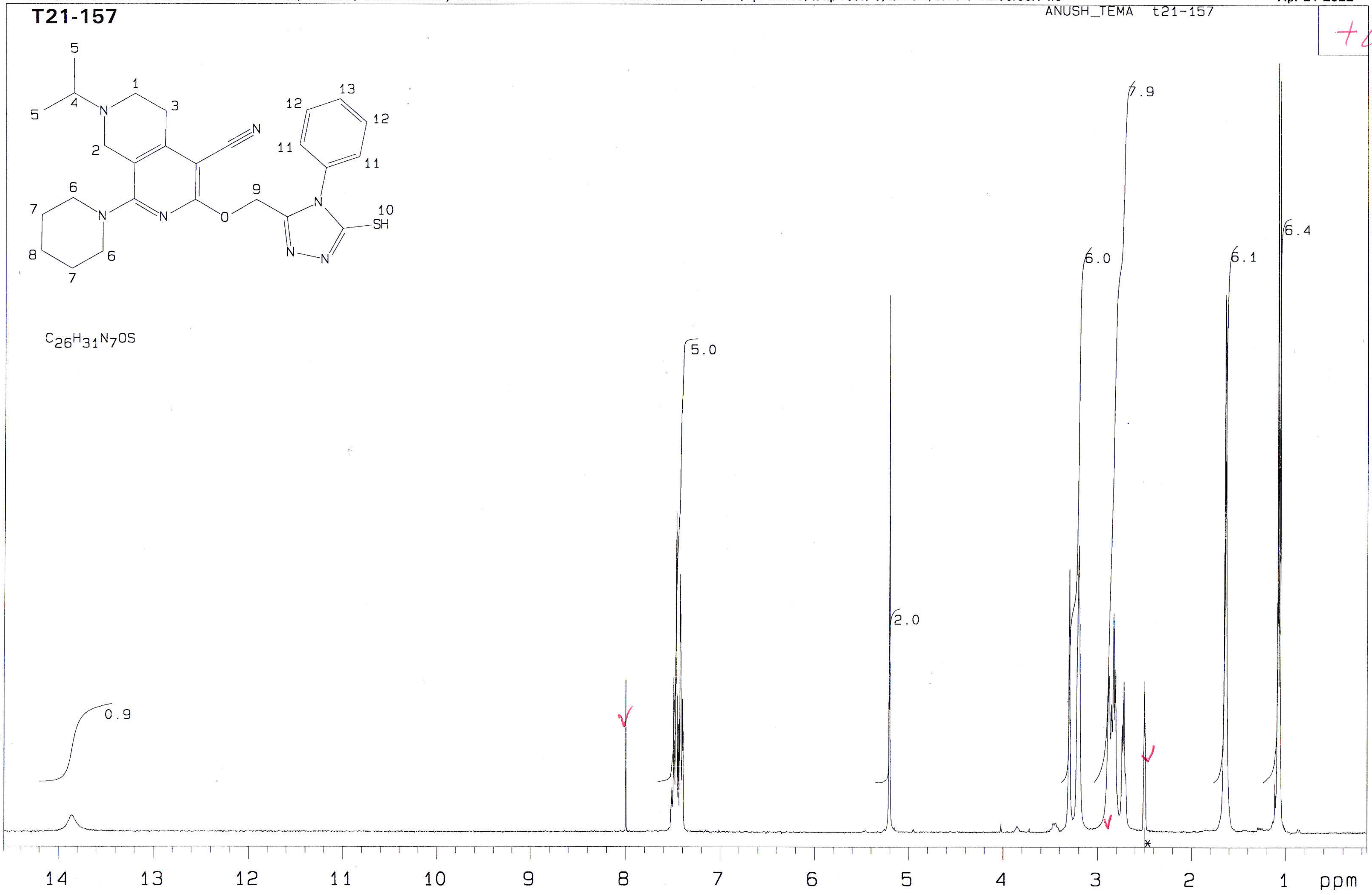

56

Molecular Structure Research Centre, Yerevan, Armenia, Varian Mercury-300VX

C13 75.465 MHz, nt=320, np=19998, temp=30.0 C, lb=1.0, solvent=DMSO/CCl4 1/3

Apr 21 2022

ANUSH_TEMA t21-157

T21-157

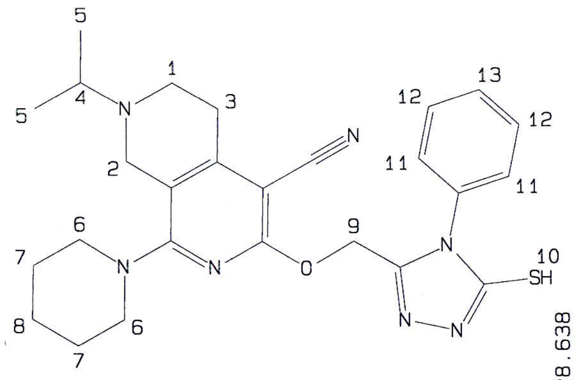

$C_{26}H_{31}N_7OS$

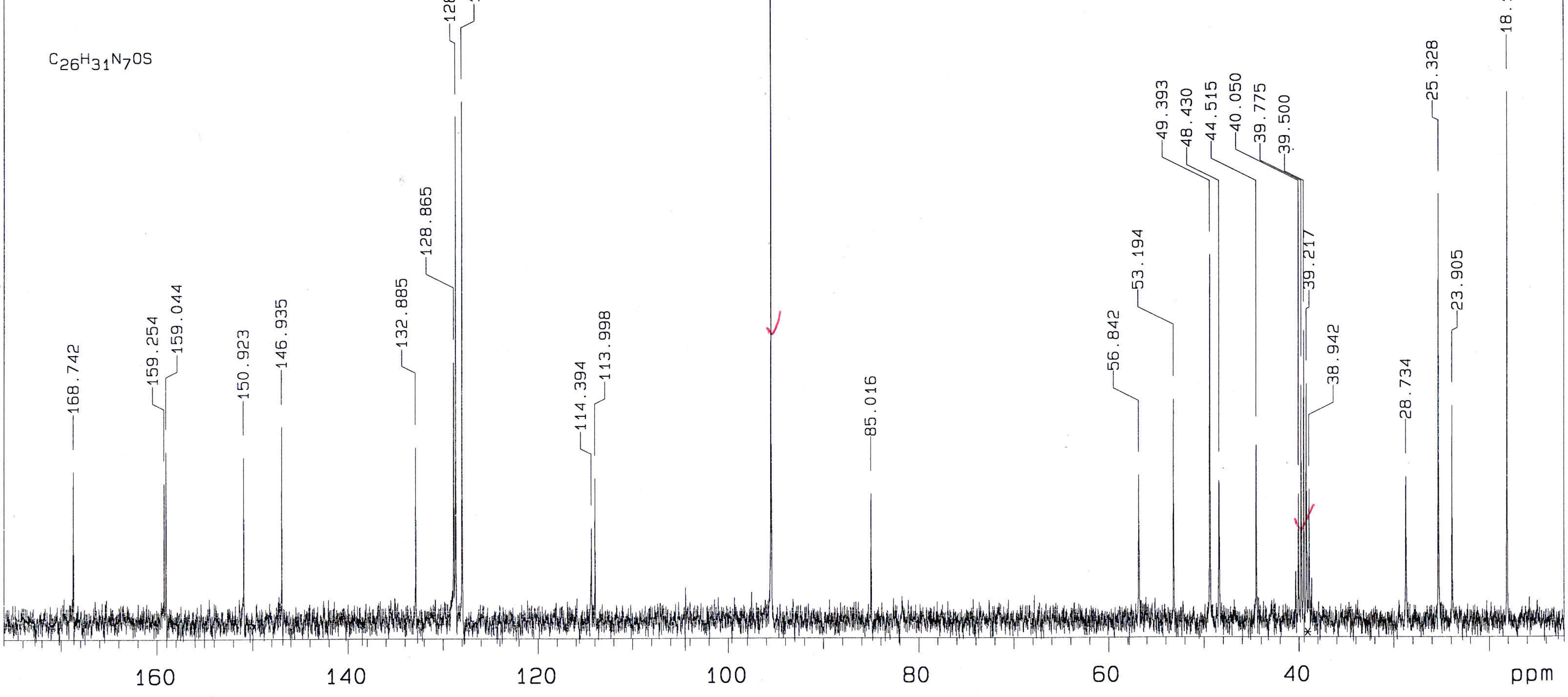

5c

Molecular Structure Research Centre, Yerevan, Armenia, Varian Mercury-300VX

H1 300.088 MHz, nt = 16, np = 32000, temp = 30.0 C, lb = -0.2, solvent = DMSO/CCl4 1/3

Dec 13 2021

ANUSH_TEMA t21-048

T21-048

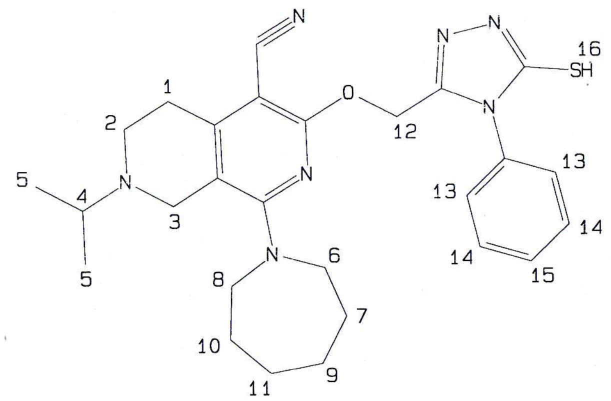

$C_{27}H_{33}N_7OS$

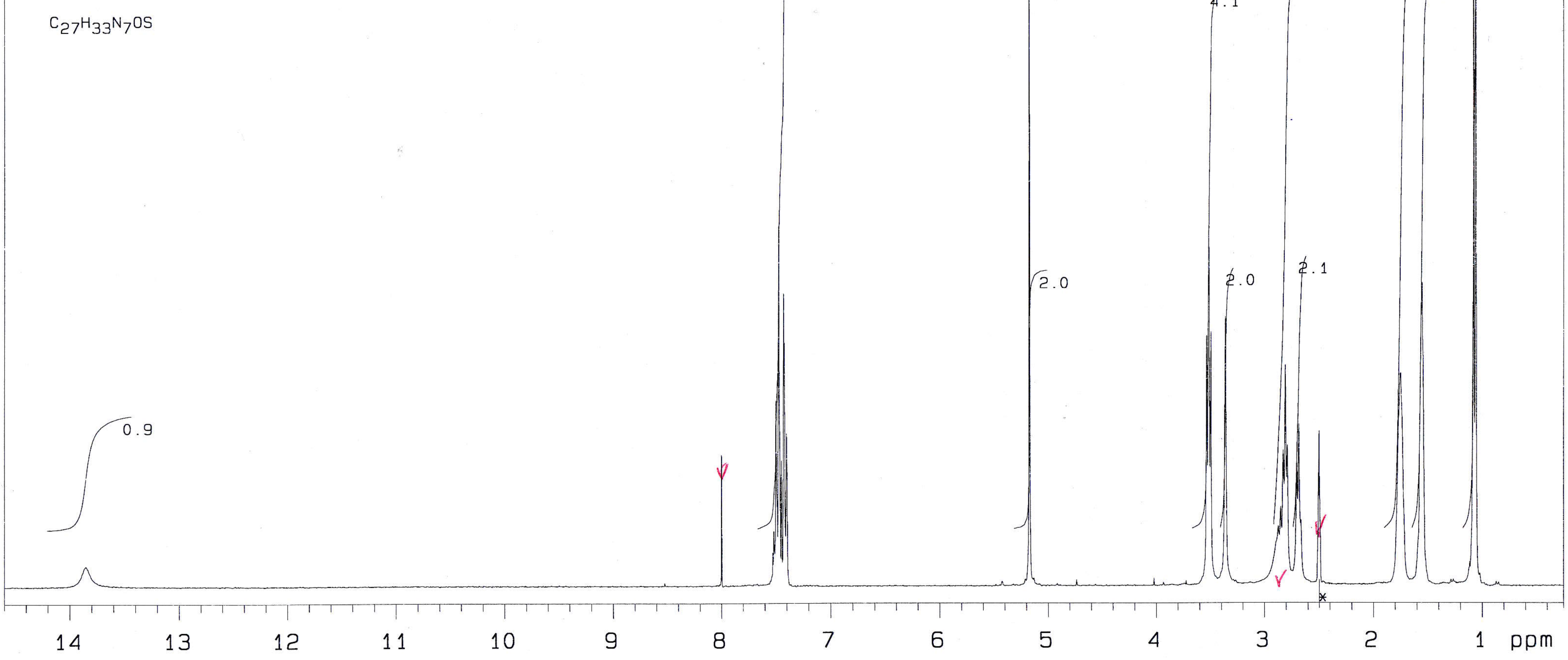

5c

Molecular Structure Research Centre, Yerevan, Armenia, Varian Mercury-300VX

C13 75.465 MHz, nt=640, np=19998, temp=30.0 C, lb=2.0, solvent=DMSO/CCl4 1/3

Dec 13 2021

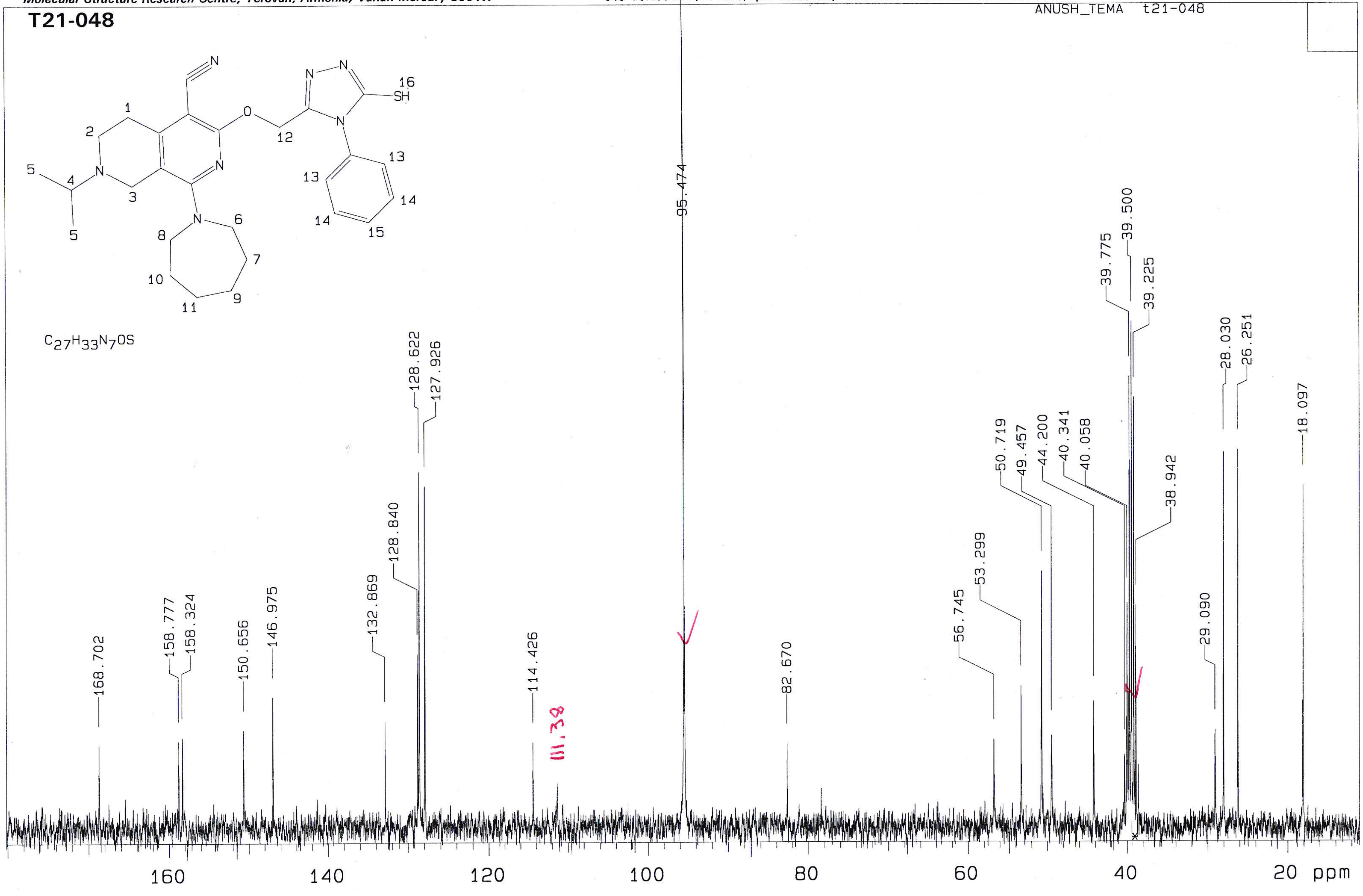

501

Molecular Structure Research Centre, Yerevan, Armenia, Varian Mercury-300VX

H1 300.088 MHz, nt=16, np=32000, temp=30.0 C, lb=-0.2, solvent=DMSO/CCl4 1/3

Jun 8 2022

T21-176

ANUSH_TEMA t21-176

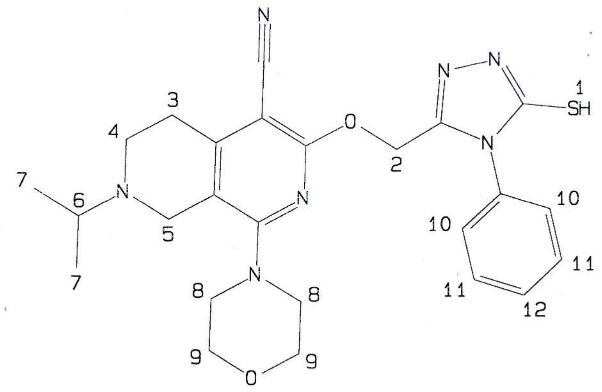

$C_{25}H_{29}N_7O_2S$

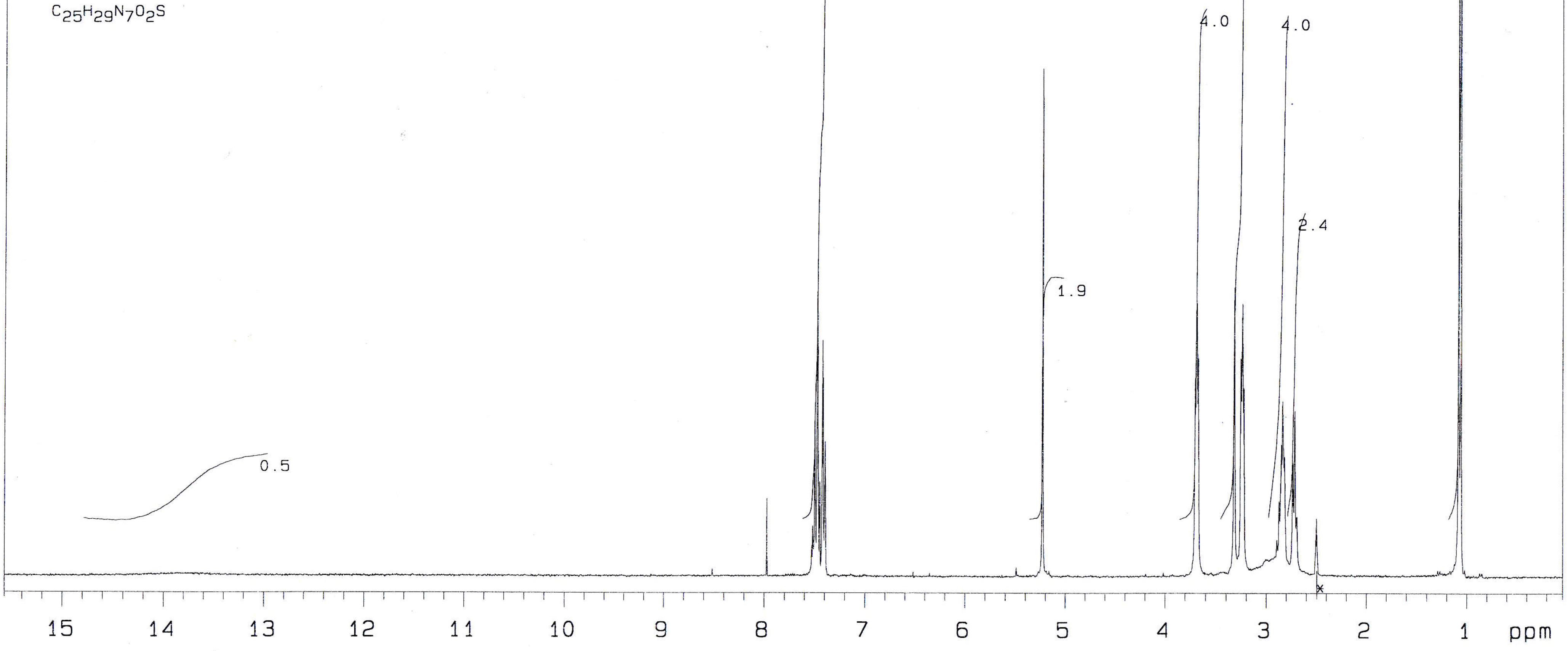

5d

Molecular Structure Research Centre, Yerevan, Armenia, Varian Mercury-300VX

C13 75.465 MHz, nt = 256, np = 19998, temp = 30.0 C, lb = 1.0, solvent = DMSO/CCl4 1/3

Jun 8 2022

T21-176

ANUSH_TEMA t21-176

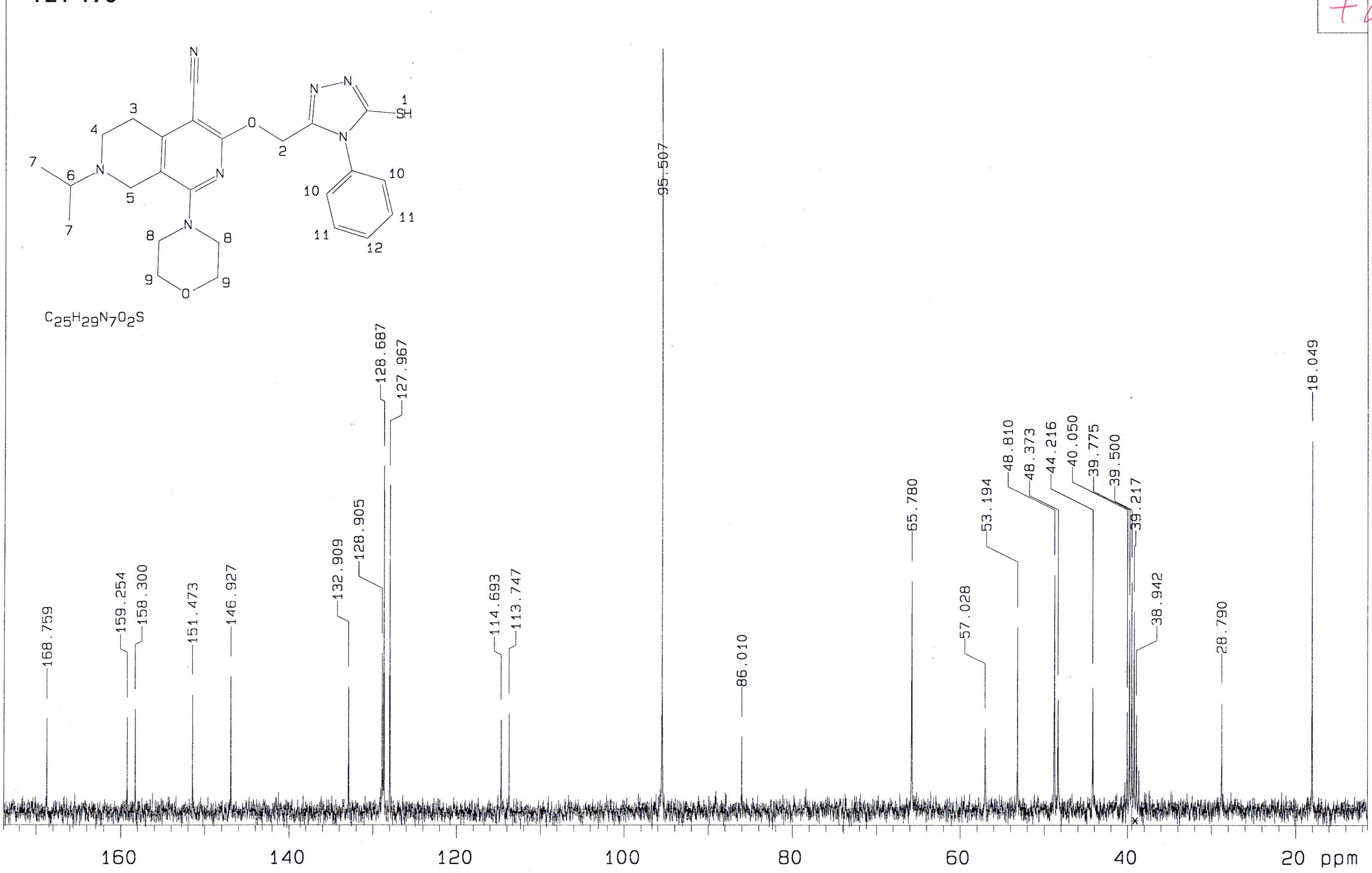

Molecular Structure Research Centre, Yerevan, Armenia, Varian Mercury-300VX

H1 300.088 MHz, nt = 16, np = 32000, temp = 30.0 C, lb = -0.2, solvent = DMSO/CCl4 1/3

May 25 2023

5e

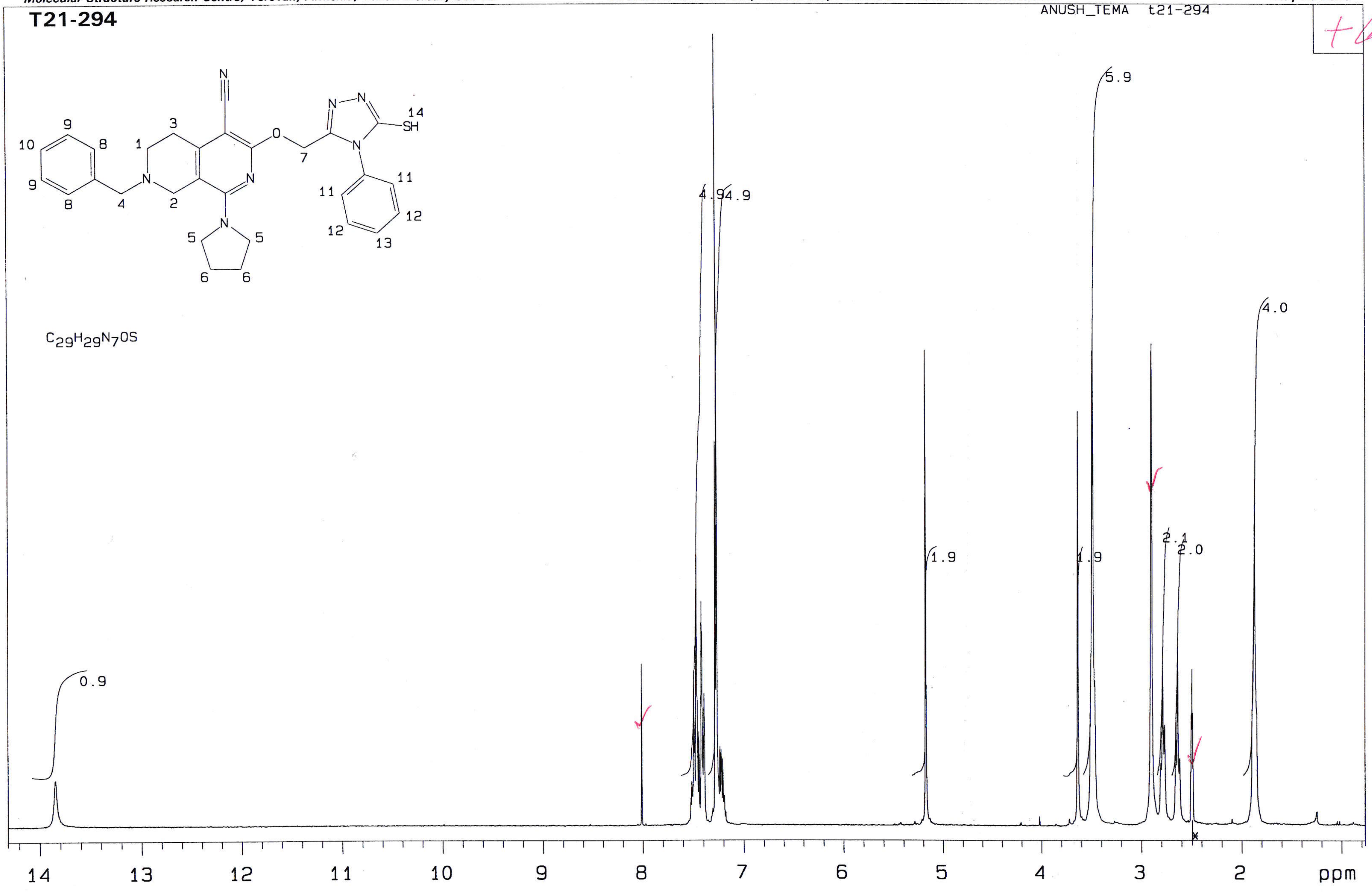

5e

Molecular Structure Research Centre, Yerevan, Armenia, Varian Mercury-300VX

C13 75.465 MHz, nt = 160, np = 19998, temp = 30.0 C, lb = 1.0, solvent = DMSO/CCl4 1/3

May 25 2023

ANUSH_TEMA t21-294

T21-294

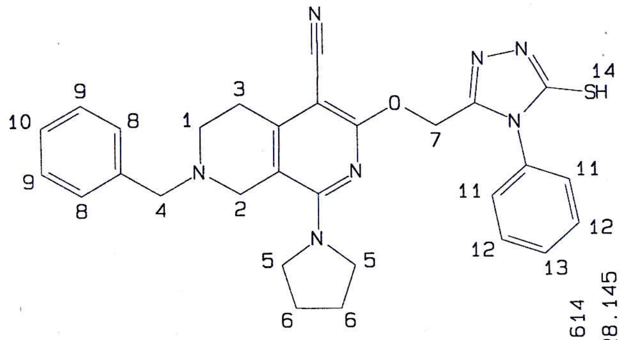

$C_{29}H_{29}N_7OS$

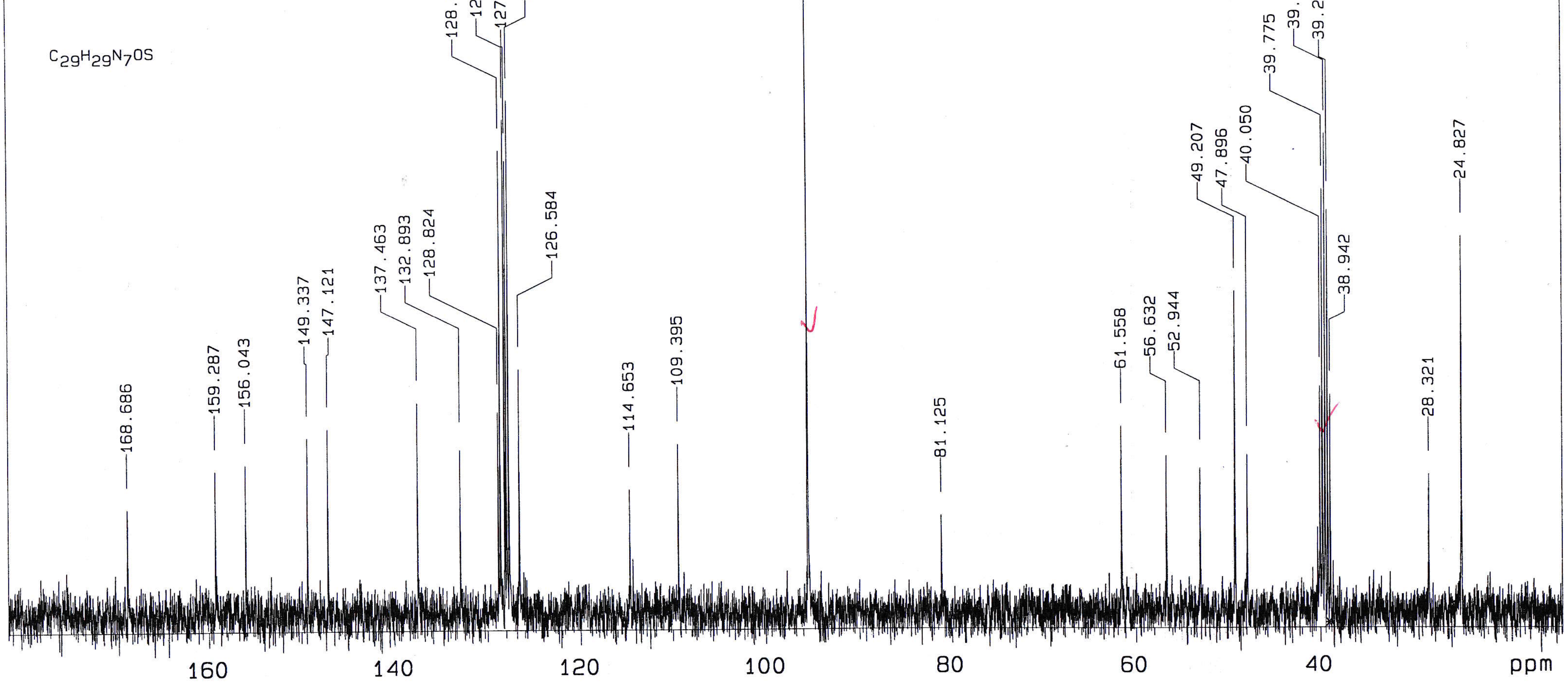

5f

Molecular Structure Research Centre, Yerevan, Armenia, Varian Mercury-300VX

H1 300.088 MHz, nt = 16, np = 32000, temp = 30.0 C, lb = -0.2, solvent = DMSO/CCl4 1/3

May 22 2023

T21-290

ANUSH_TEMA t21-290

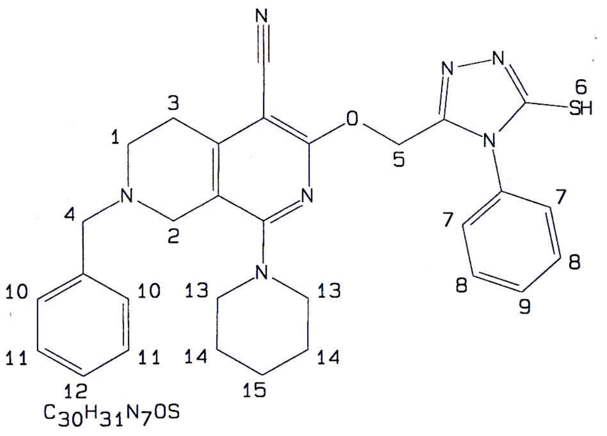

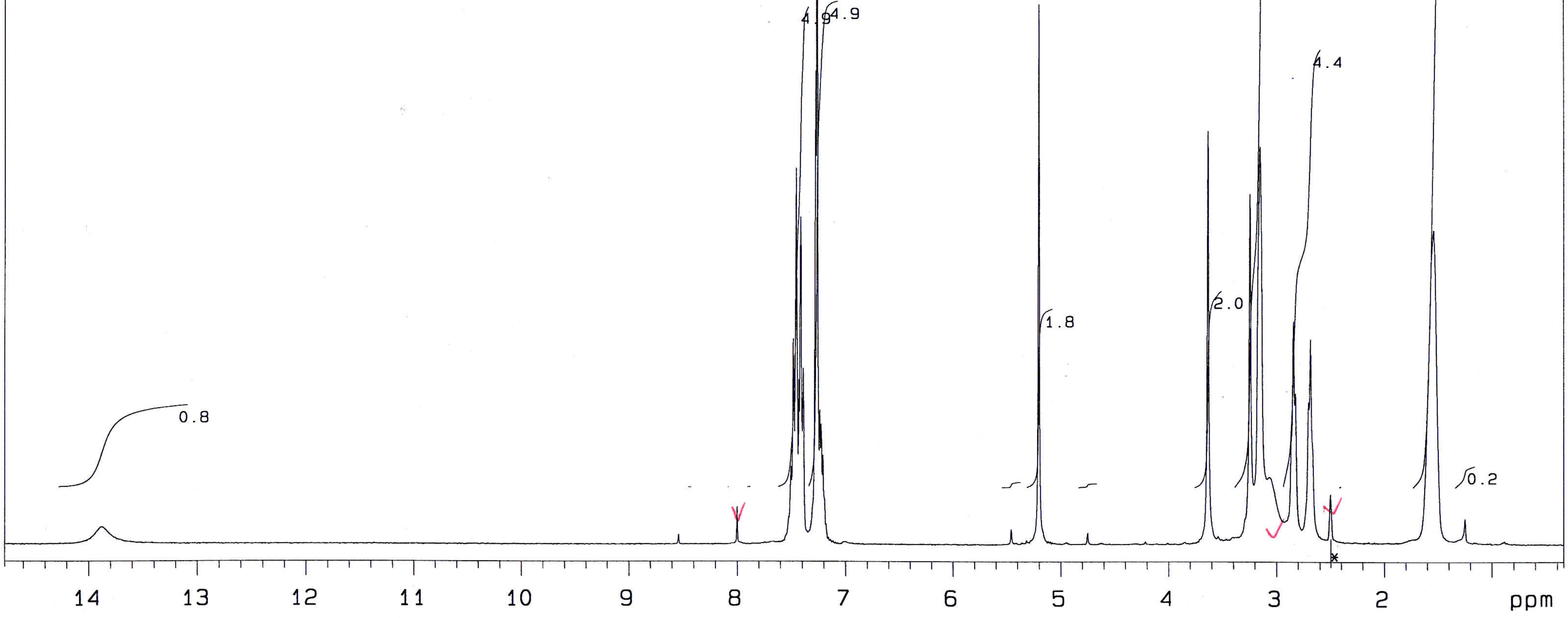

5f

Molecular Structure Research Centre, Yerevan, Armenia, Varian Mercury-300VX

C13 75.465 MHz, nt = 192, np = 19998, temp = 30.0 C, lb = 1.0, solvent = DMSO/CCl4 1/3

May 22 2023

ANUSH_TEMA t21-290

T21-290

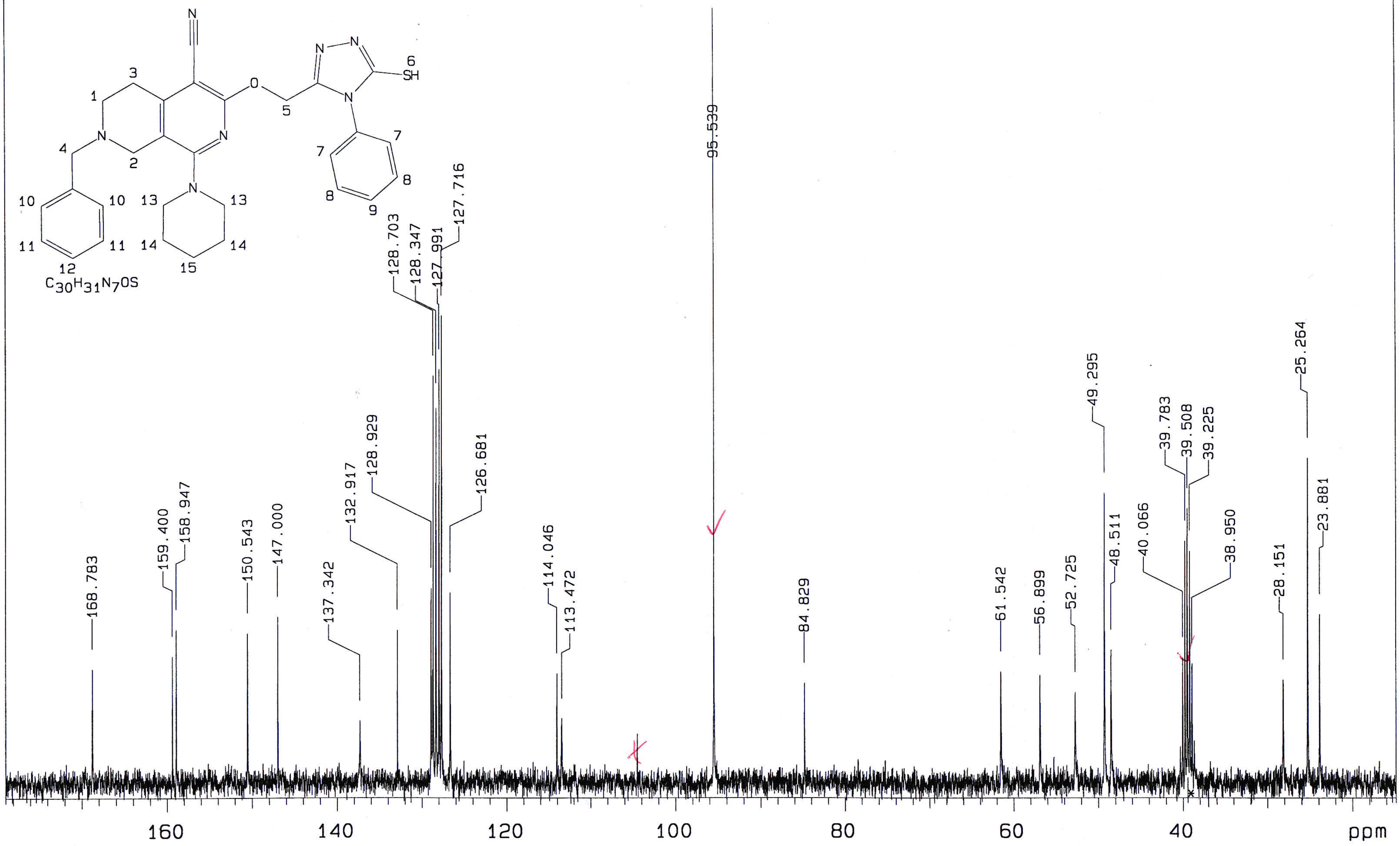

59

Molecular Structure Research Centre, Yerevan, Armenia, Varian Mercury-300VX H1 300.088 MHz, nt=16, np=32000, temp=30.0 C, lb=-0.2, solvent=DMSO/CCl4 1/3 **Apr 6 2023**

ANUSH_TEMA t21-262

T21-262

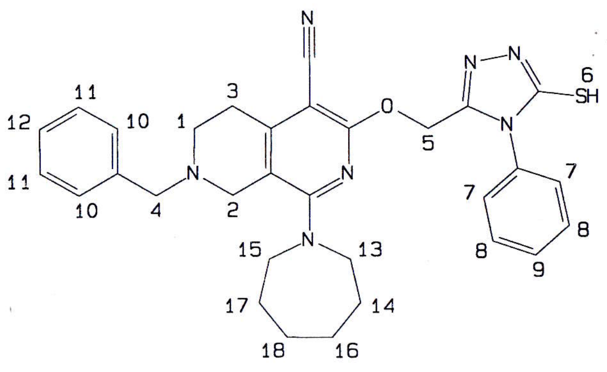

$C_{31}H_{33}N_7OS$

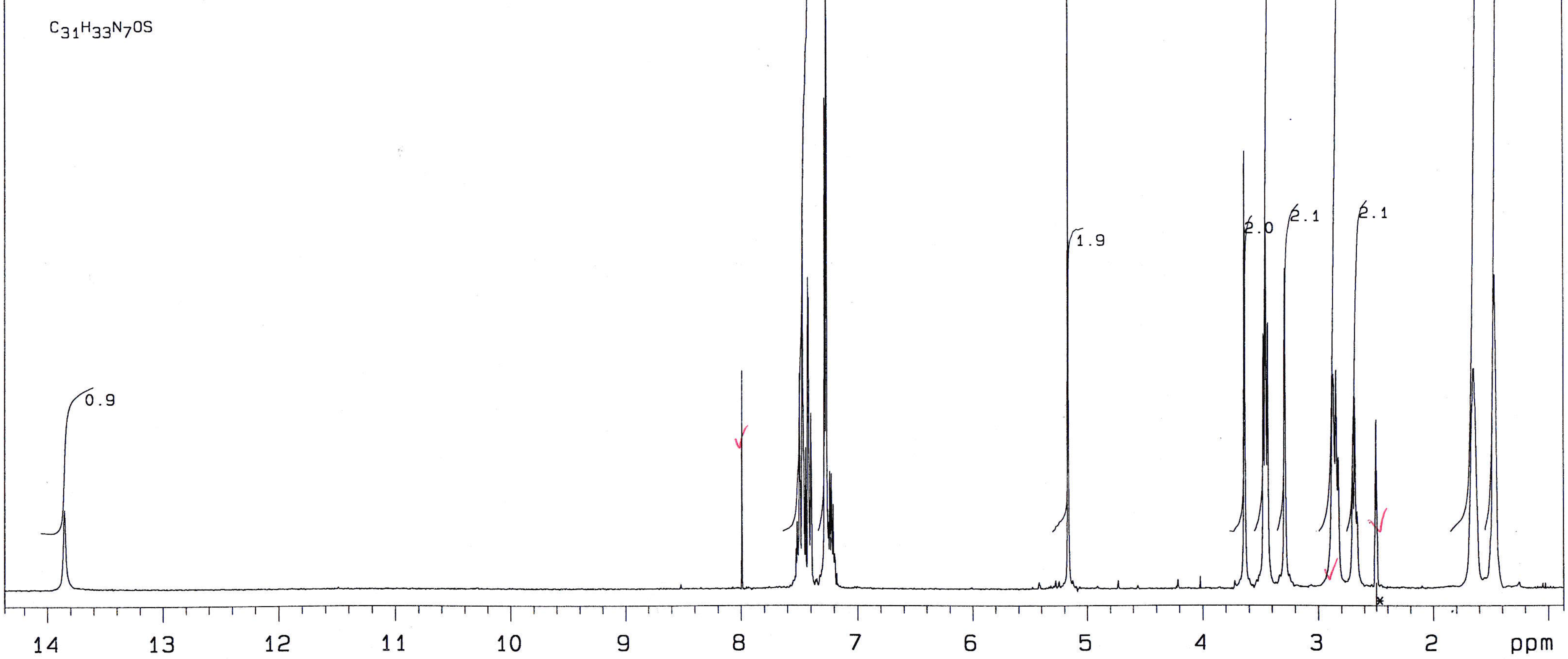

59

Molecular Structure Research Centre, Yerevan, Armenia, Varian Mercury-300VX

C13 75.465 MHz, nt = 192, np = 19998, temp = 30.0 C, lb = 1.0, solvent = DMSO/CCl4 1/3

Apr 6 2023

ANUSH_TEMA t21-262

T21-262

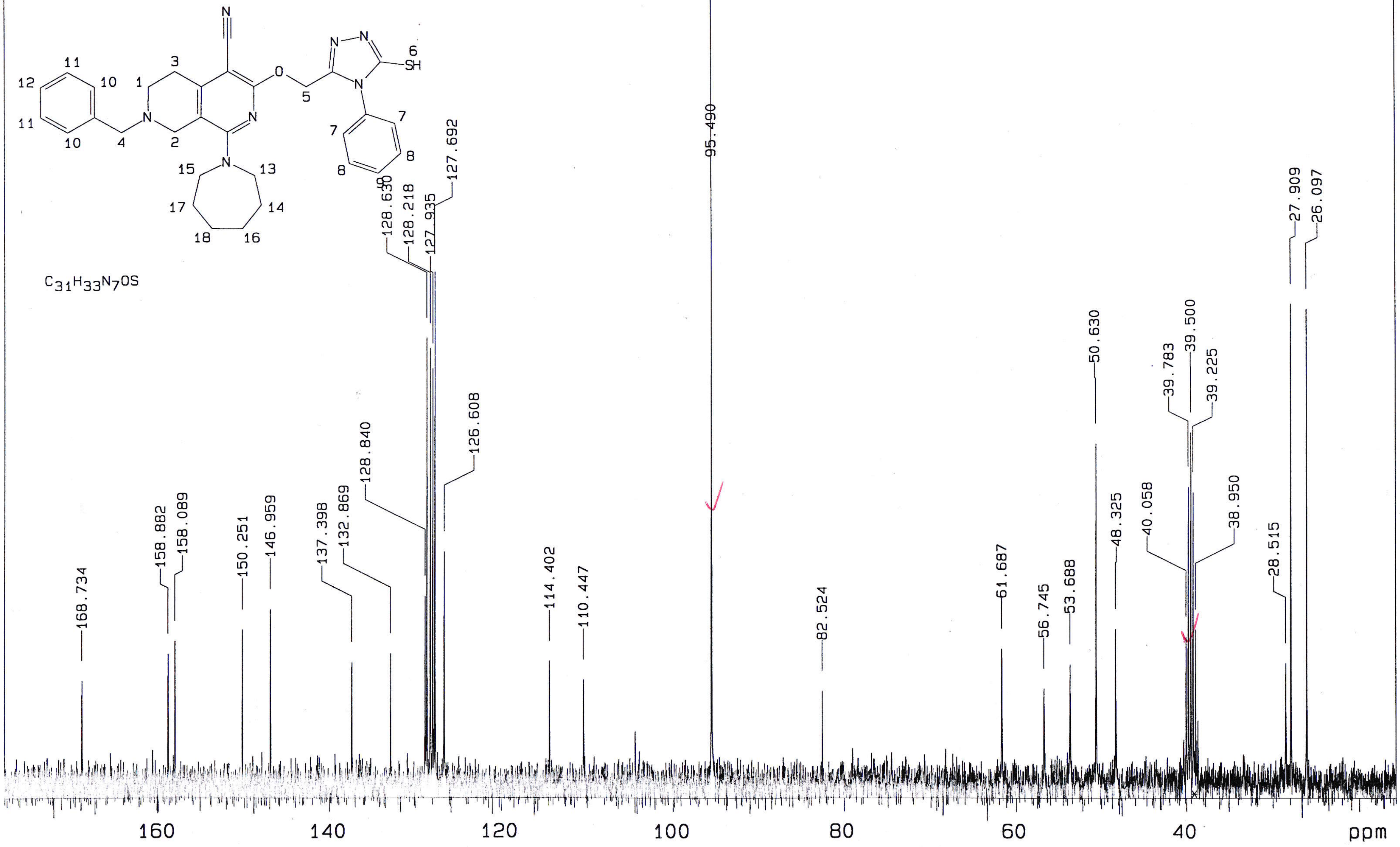

5h

Molecular Structure Research Centre, Yerevan, Armenia, Varian Mercury-300VX

H1 300.088 MHz, nt = 16, np = 32000, temp = 30.0 C, lb = -0.2, solvent = DMSO/CCl4 1/3

May 22 2023

ANUSH_TEMA t21-288

T21-288

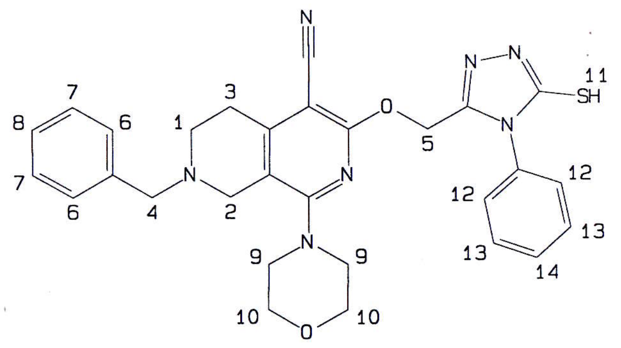

$C_{29}H_{29}N_7O_2S$

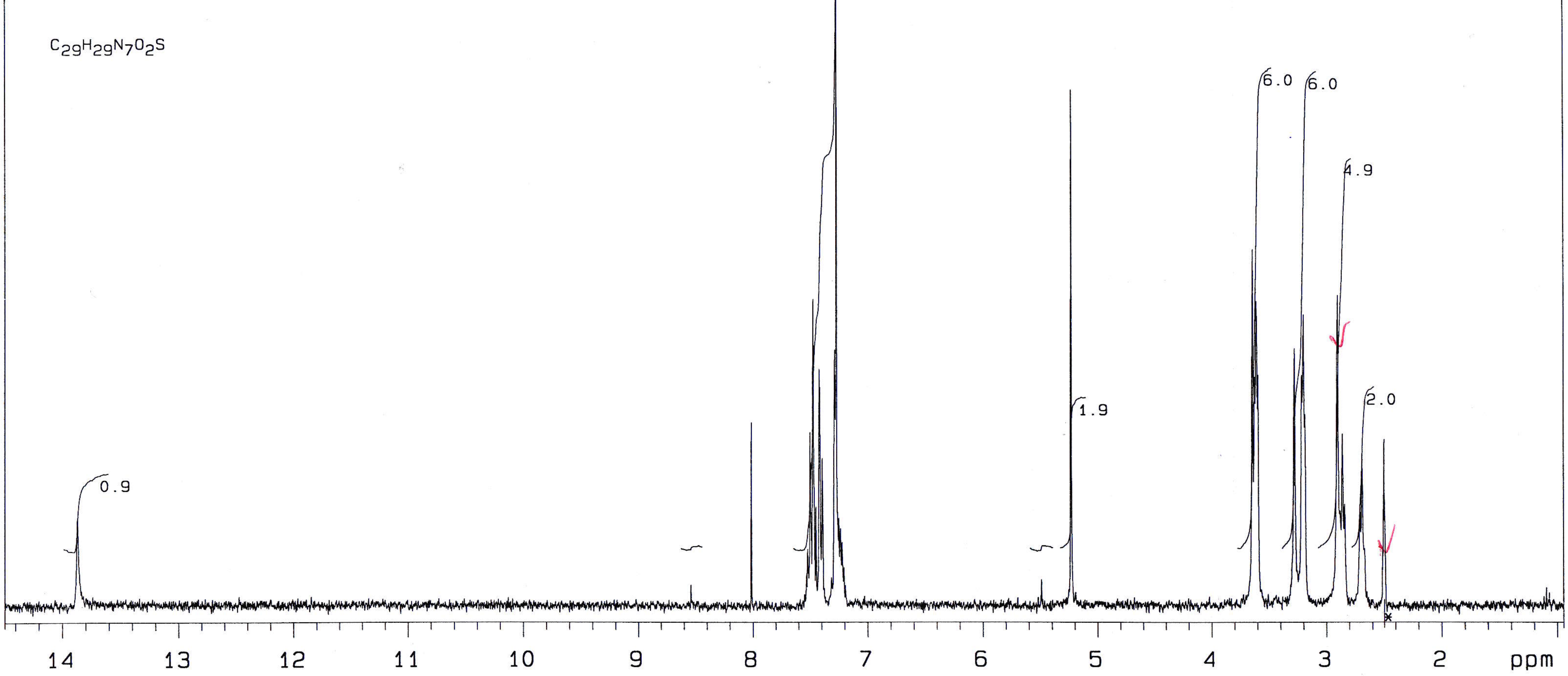

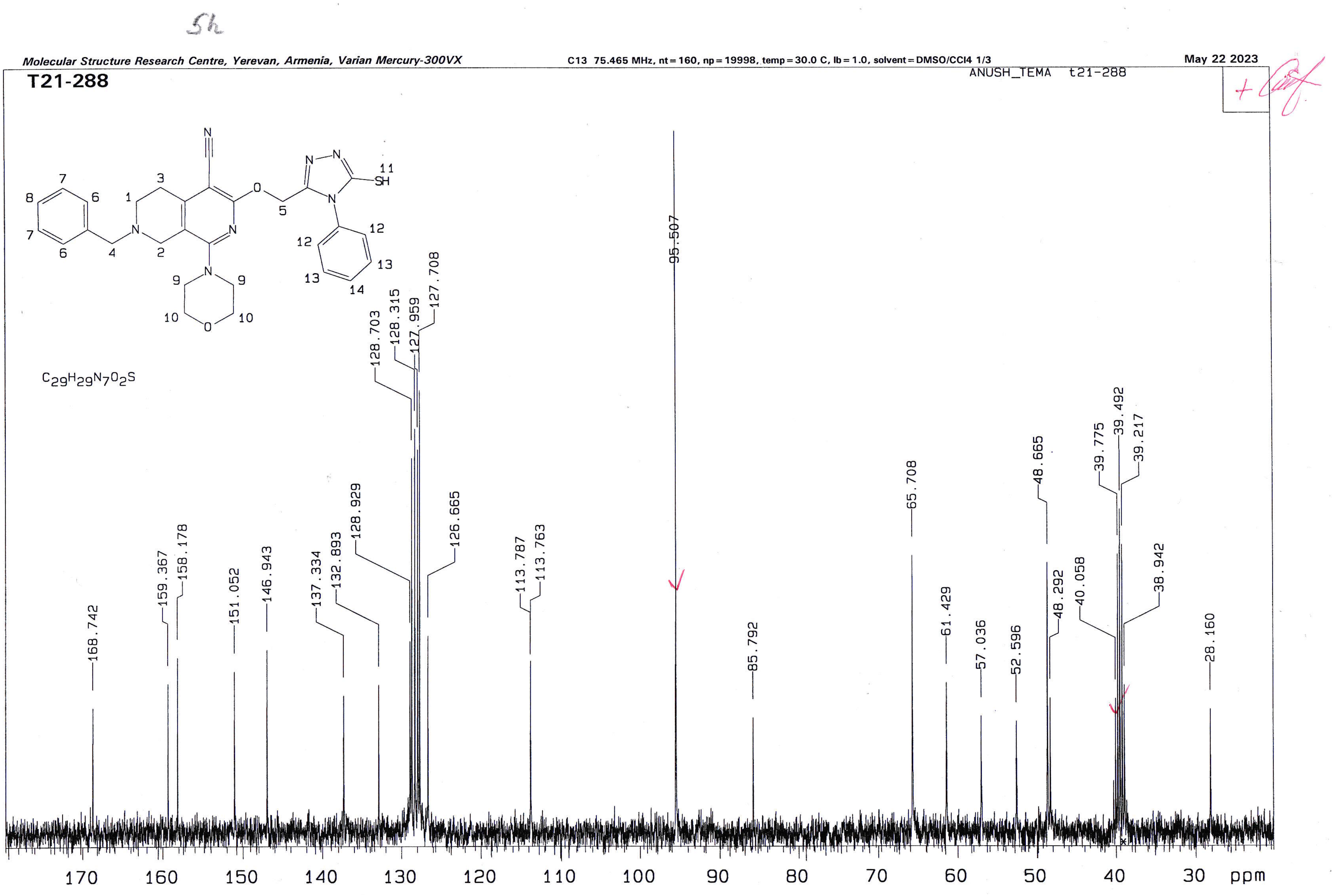
5h
Molecular Structure Research Centre, Yerevan, Armenia, Varian Mercury-300VX
C13 75.465 MHz, nt=160, np=19998, temp=30.0 C, lb=1.0, solvent=DMSO/CCl4 1/3
May 22 2023
T21-288
ANUSH_TEMA t21-288
$C_{29}H_{29}N_7O_2S$
168.742
159.367
158.178
151.052
146.943
137.334
132.893
128.929
128.703
128.315
127.959
127.708
126.665
113.787
113.763
95.507
85.792
65.708
61.429
57.036
52.596
48.665
48.292
40.058
39.775
39.492
39.217
38.942
28.160
170
160
150
140
130
120
110
100
90
80
70
60
50
40
30
ppm

Molecular Structure Research Center, NAS RA, Yerevan, Armenia

7a

December 9, 2025 3:23:14 PM AMT

Avance 400 Neo
TopSpin 4.1.3

k-012, 1H, CDCL3, temp=30

k-012 1 1 F:/FID_BRUKER/NOCI_25

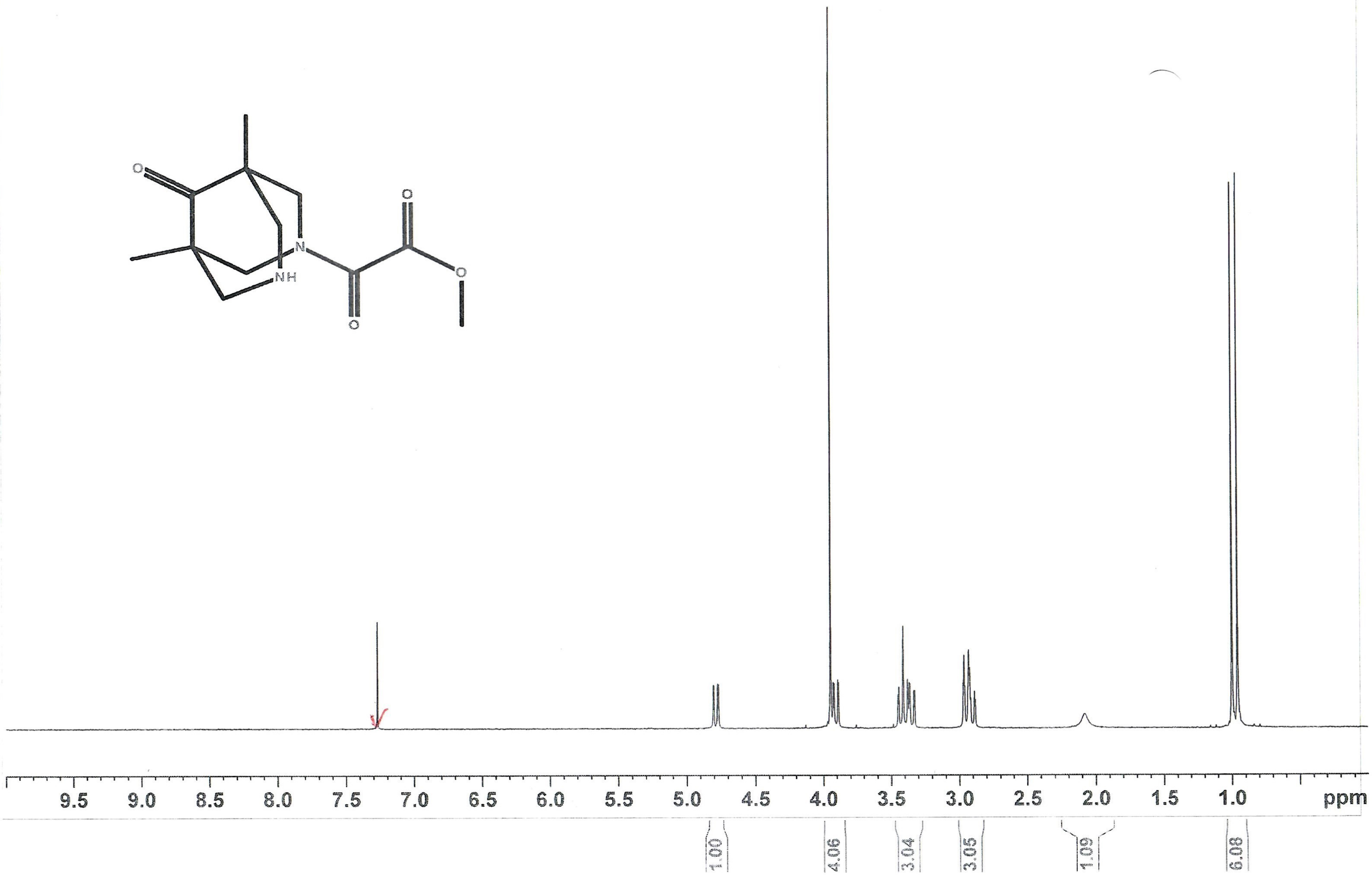

© Molecular Structure Research Center, NAS RA, Yerevan, Armenia

7a

December 9, 2025 3:23:50 PM AMT

Avance 400 Neo
TopSpin 4.1.3

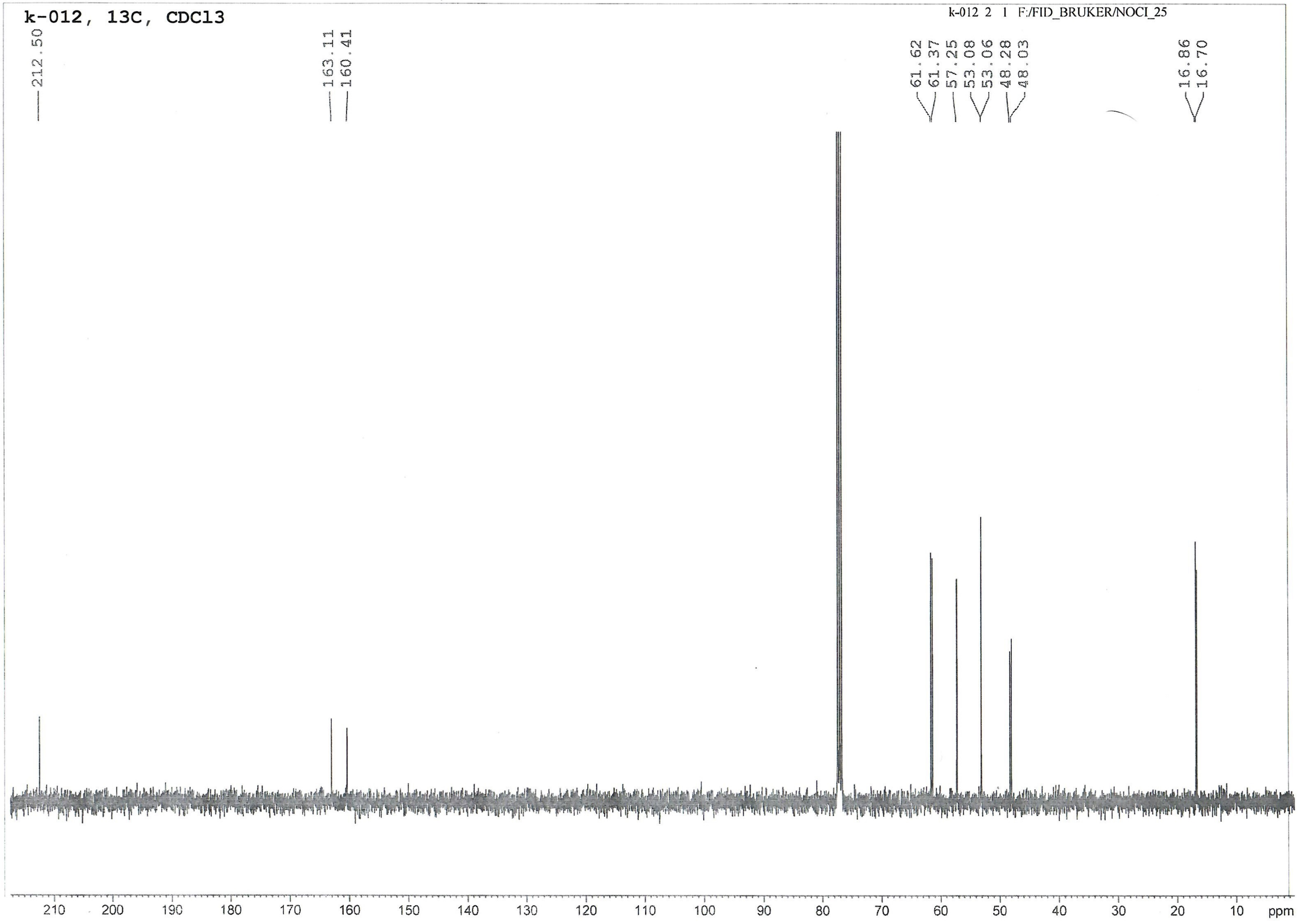

Molecular Structure Research Center, NAS RA, Yerevan, Armenia

76

December 9, 2025 3:20:01 PM AMT

Avance 400 Neo
TopSpin 4.1.3

gk-1849, 1H, CDCL3, temp=30

gk-1849 1 1 F:/FID_BRUKER/NOCI_25

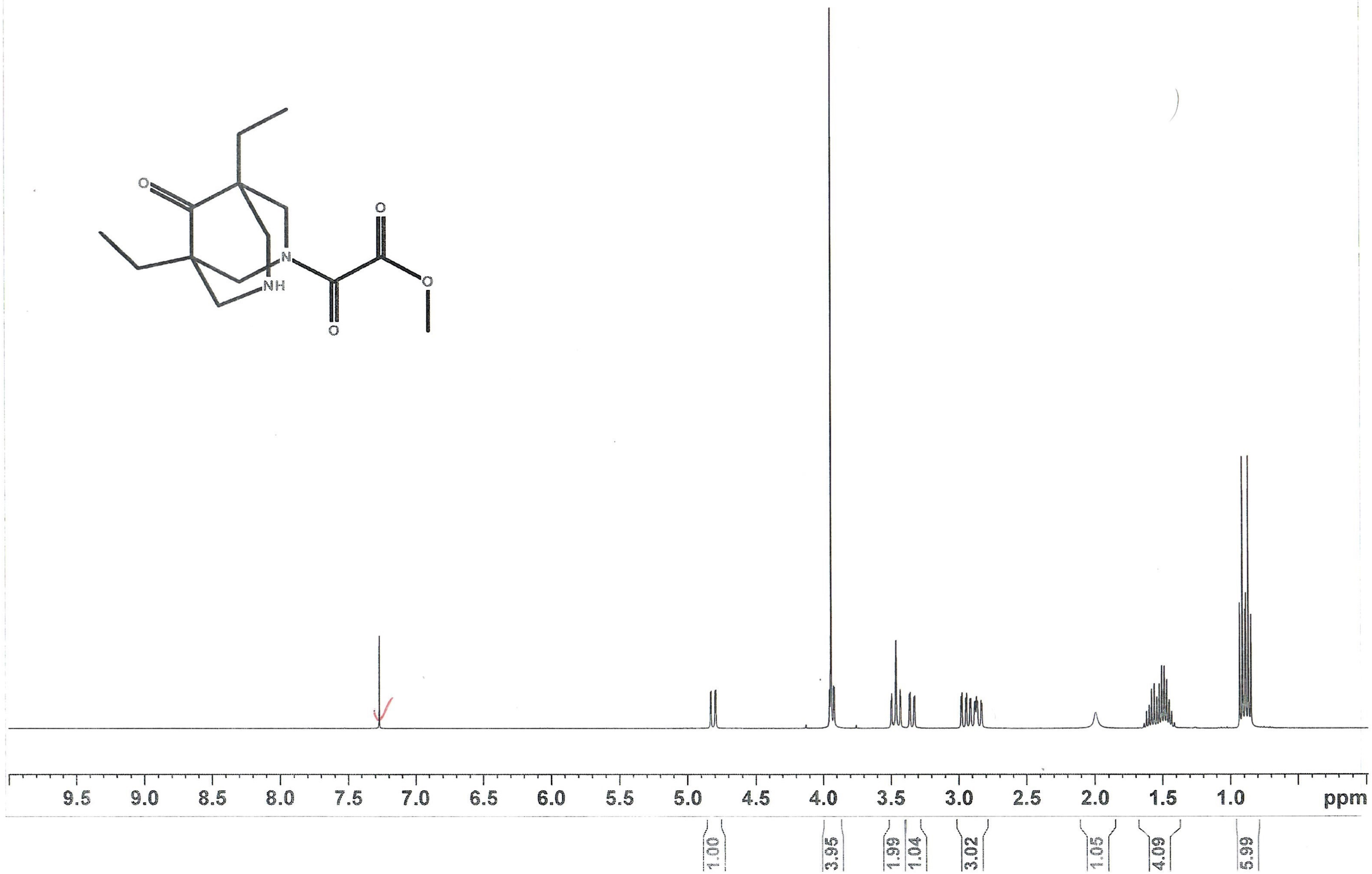

© Molecular Structure Research Center, NAS RA, Yerevan, Armenia

7b

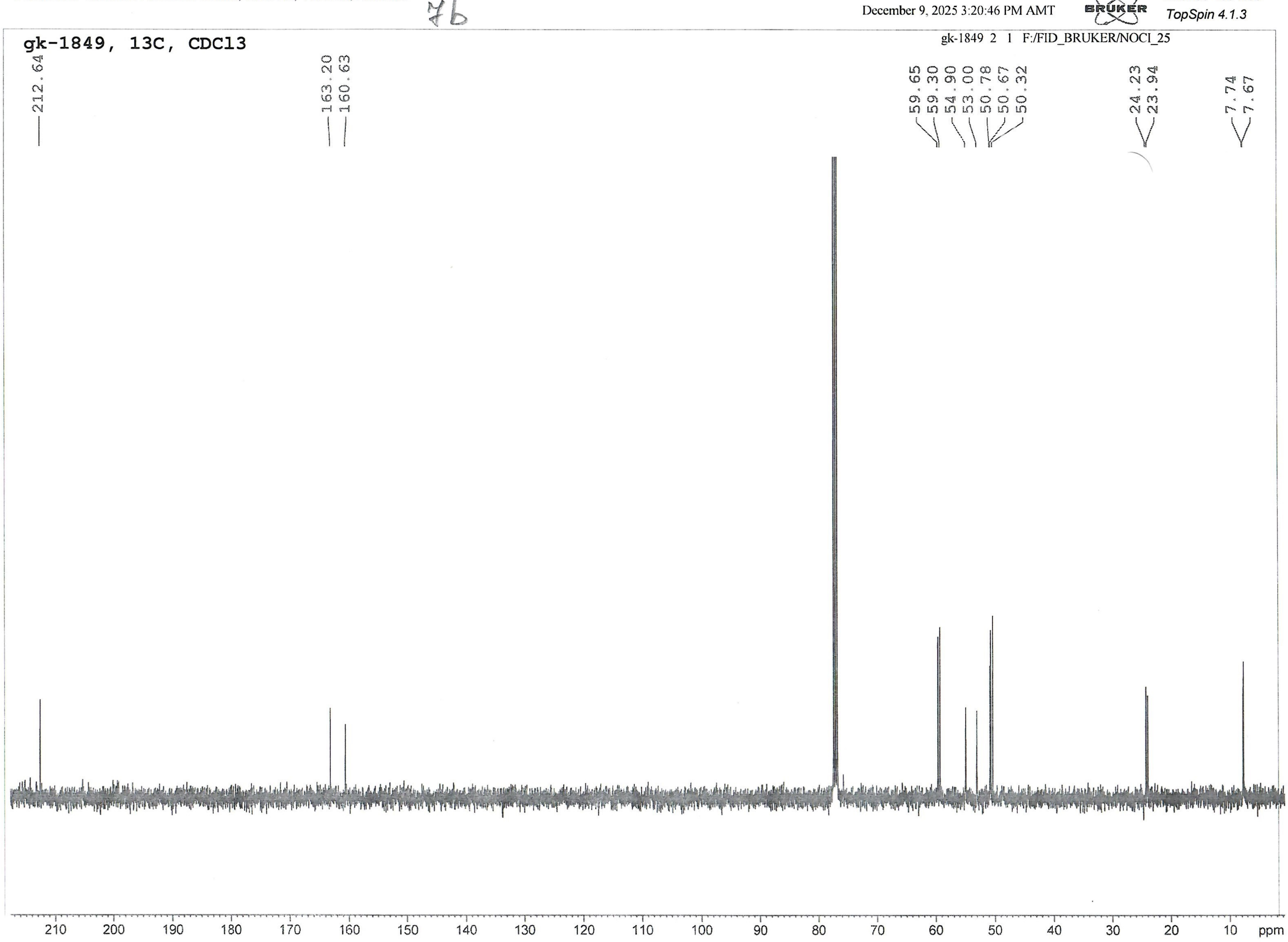

gk-1051 8a

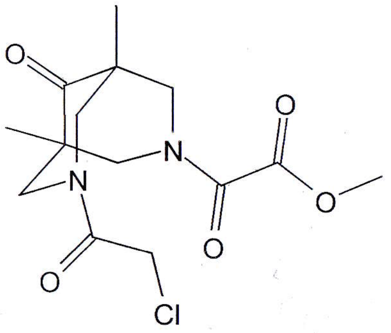

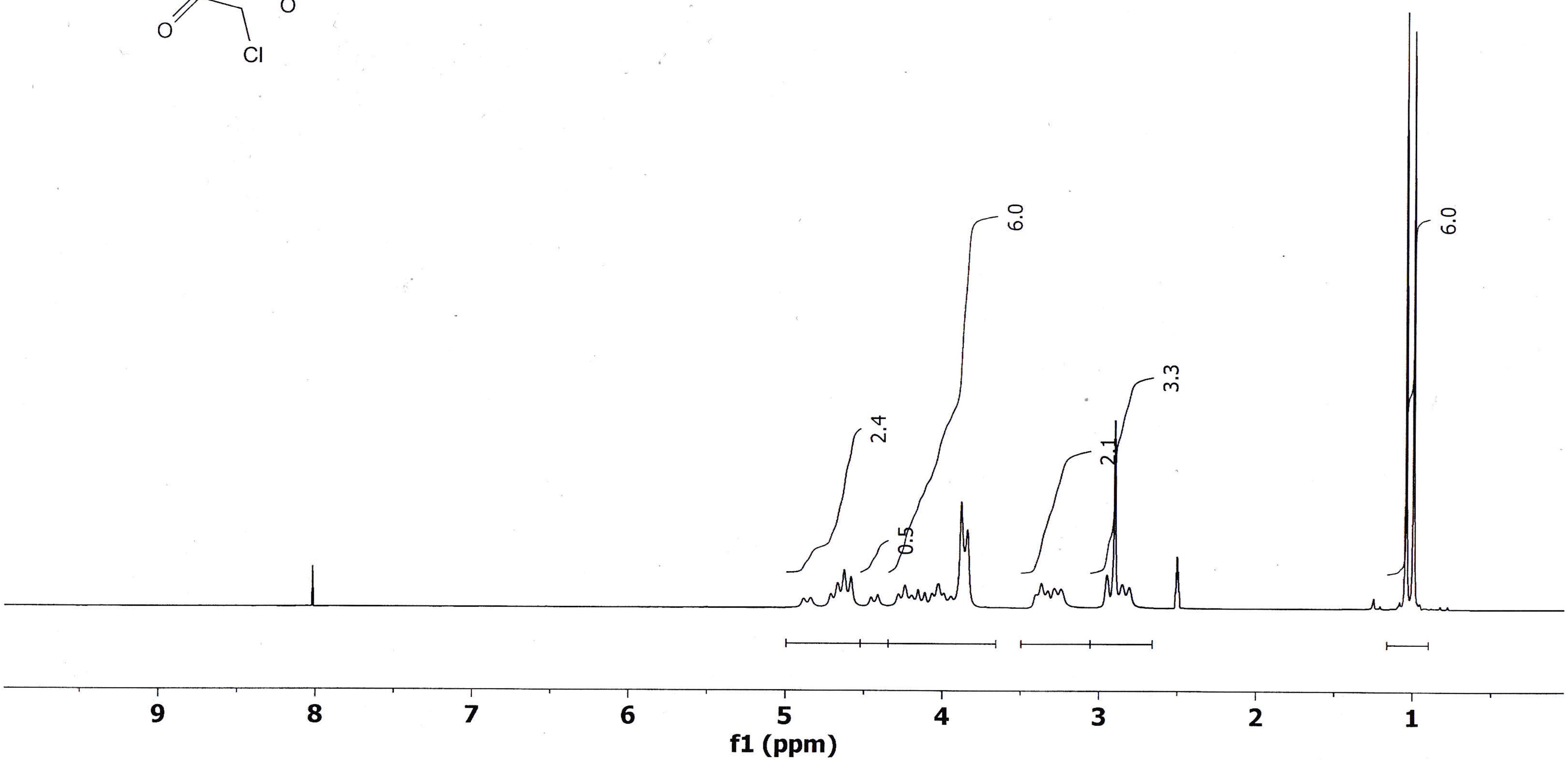

gk-1051_c13 8a

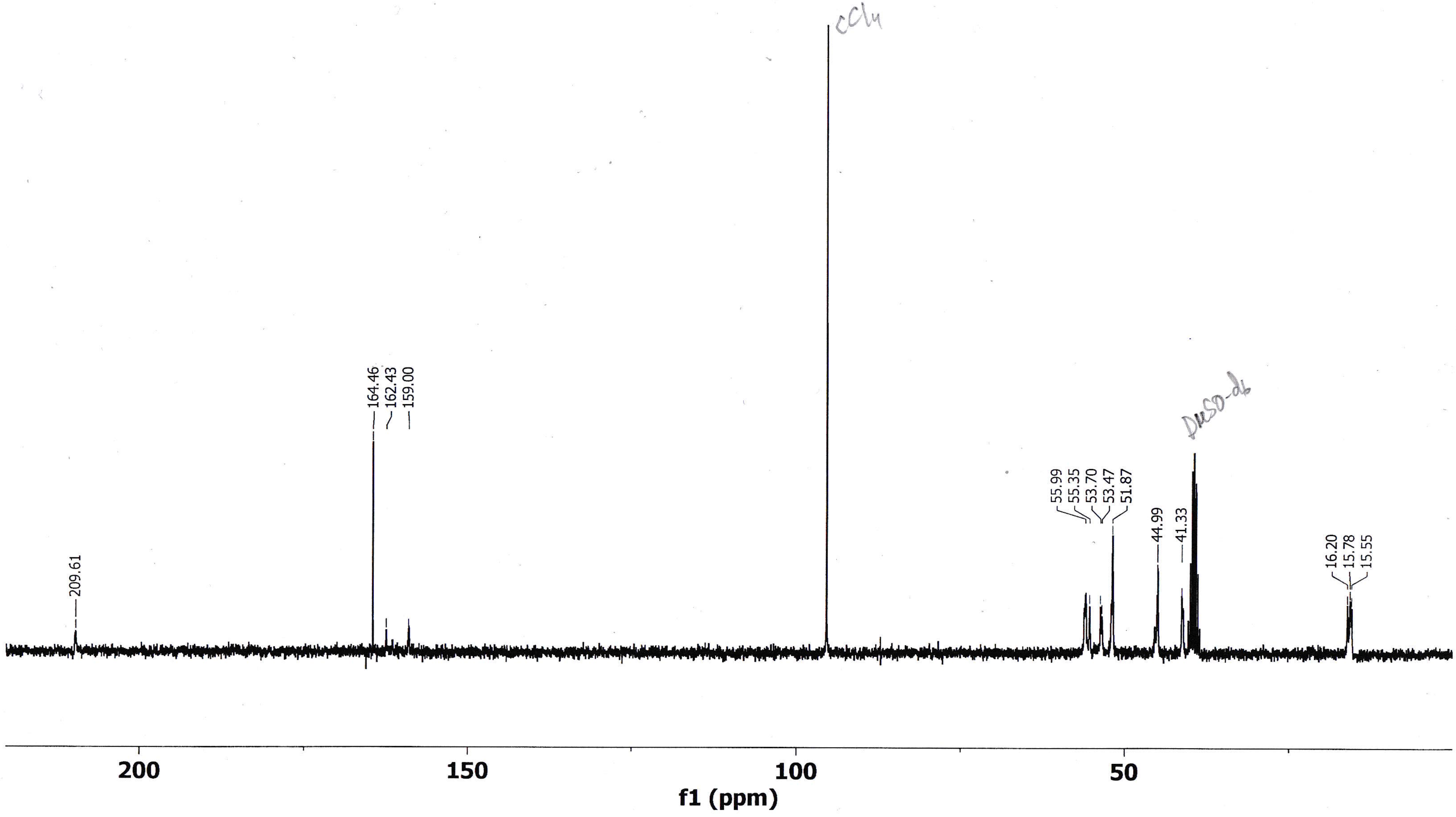

I

8b

Molecular Structure Research Centre, Yerevan, Armenia, Varian Mercury-300VX

H1 300.088 MHz, nt=16, np=32000, temp=30.0 C, lb=-0.2, solvent=DMSO/CCl4 1/3

May 10 2022

GK-1850B

NOCI_22 gk-1850b

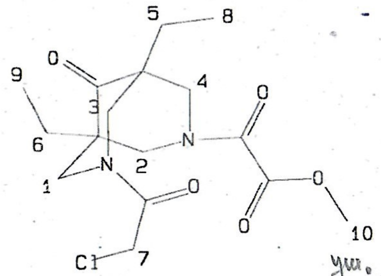

$C_{16}H_{23}ClN_2O_5$

¹H: 0,93 уш. т (3H, J=7.4, CH_3)
0,96 уш. т (3H, J=7.4, CH_3)
1.52 уш. к (2H, J=7.4, $\underline{CH_2}CH_3$)
1.58 уш. к (2H, J=7.4, $\underline{CH_2}CH_3$)
2.76–3.01 м (2H), 3,23–3,43 м (2H),
3,79–4.22 м (6H) и 4,36–4,86 м (3H).

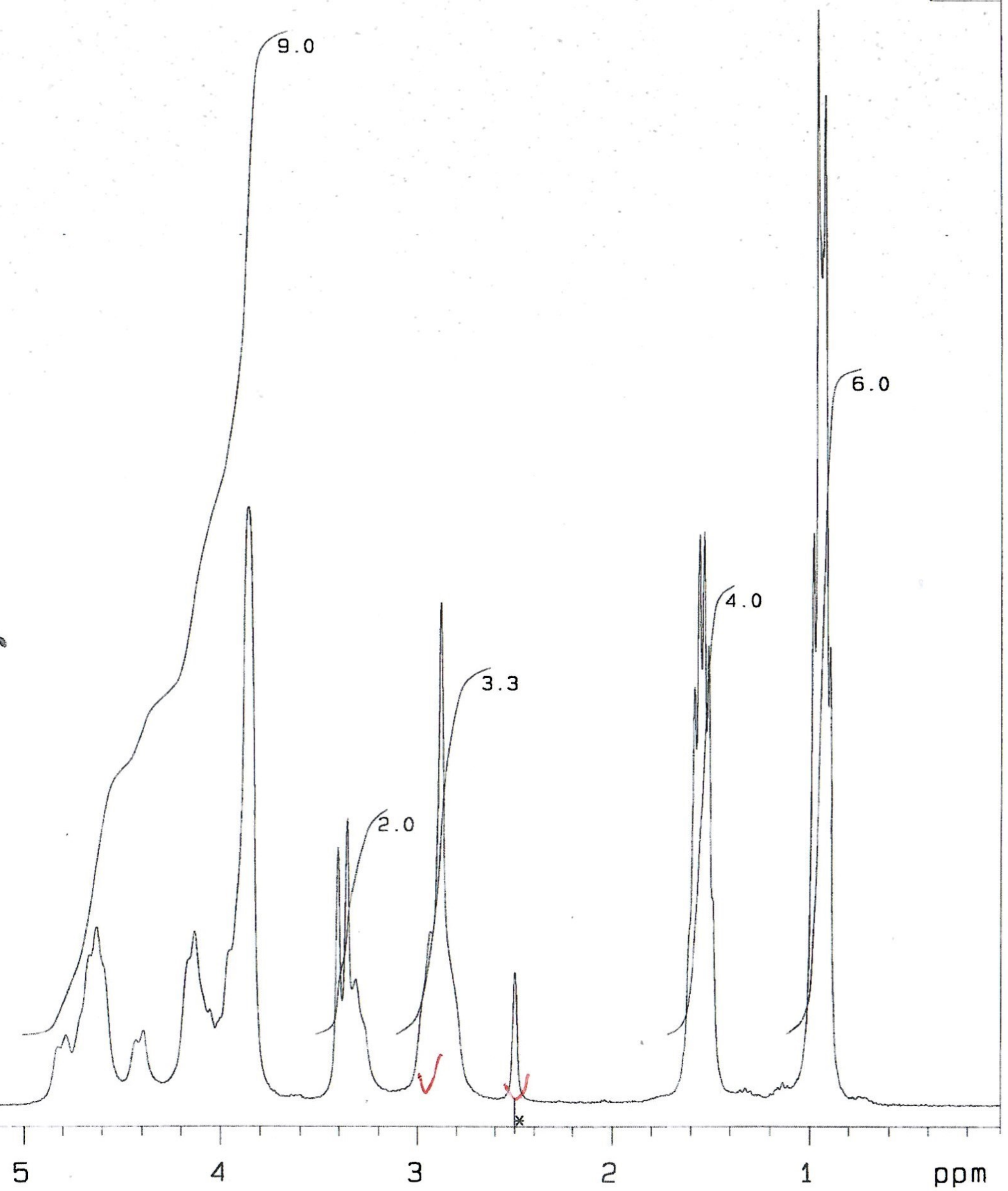

Supplement: RA-016-D6RA00302H-s001 [file RA-016-D6RA00302H-s001.pdf]
