# Supplementary material for: Synthesis, neurotropic activity and docking studies of 1,2,4-triazole-linked hybrids based on 2,7-naphthyridine and bispidine rings
Source: RSC Adv. 2026 Jul 3;16(35):36518–40. doi: 10.1039/d6ra00302h (PMC13330782; doi:10.1039/d6ra00302h)
Supplement: RA-016-D6RA00302H-s002 [file RA-016-D6RA00302H-s002.pdf]

Supplementary Information (SI) for RSC Advances.
This journal is © The Royal Society of Chemistry 2026

Supplementary Data \ File 2

Synthesis, neurotropic activity and docking studies of 1,2,4-triazole-linked hybrids based on 2,7-naphthyridine and bispidine rings

Samvel N. Sirakanyan,[a] Athina Geronikaki,[b,*] Anush A. Hovakimyan,[a,*] Anthi Petrou,[b] Victor G. Kartsev,[c] Hasmik A. Yegoryan,[a] Hasmik V. Jughetsyan,[a] Sahak P. Gasparyan,[a] Ruzanna G. Paronikyan,[a,*] Tatevik A. Araqelyan,[a] Mariam V. Galstyan,[a] Knarik A. Gevorkyan,[a] Amalya D. Harutyunyan,[a] and Elmira K. Hakobyan[a]

[a] Scientific Technological Center of Organic and Pharmaceutical Chemistry of National Academy of Science of Republic of Armenia, Institute of Fine Organic Chemistry of A.L.Mnjoyan, Armenia 0014, Yerevan; shnnr@mail.ru

[b] Department of Pharmacy, School of Health, Aristotle University of Thessaloniki, 54124 Thessaloniki, Greece; geronik@pharm.auth.gr

[c] InterBioScreen, Moscow 119019, Russia; vkartsev@ibscreen.chg.ru

* Correspondence: geronik@pharm.auth.gr (A.G.); anush.hovakimyan@gmail.com (A.A.H.); paronikyan.ruzanna@mail.ru (R.G.P.)

^{1}H, ^{13}C NMR and MS spectra for new synthesized compounds: 9a–p

9a

Molecular Structure Research Centre, Yerevan, Armenia, Varian Mercury-300VX

H1 300.088 MHz, nt = 16, np = 32000, temp = 30.0 C, lb = -0.2, solvent = DMSO

Mar 25 2022

GM-011-1

ANUSH_TEMA gm-011-1

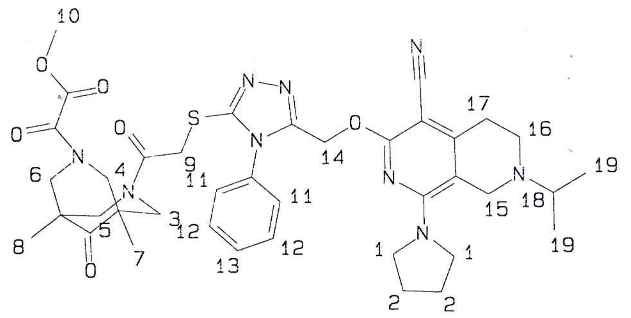

$C_{39}H_{47}N_9O_6S$

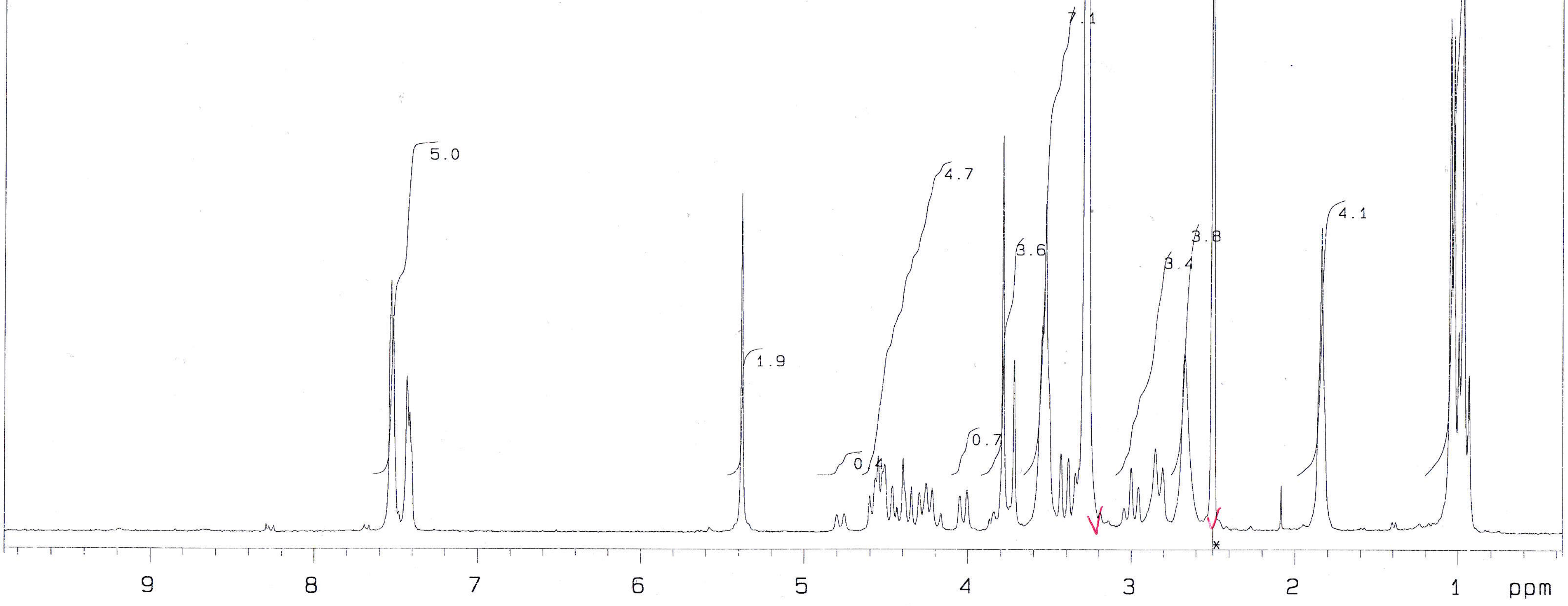

9b

Molecular Structure Research Centre, Yerevan, Armenia, Varian Mercury-300VX

H1 300.088 MHz, nt = 16, np = 32000, temp = 30.0 C, lb = -0.2, solvent = DMSO

May 5 2022

GM-018

NOCI_22 gm-018

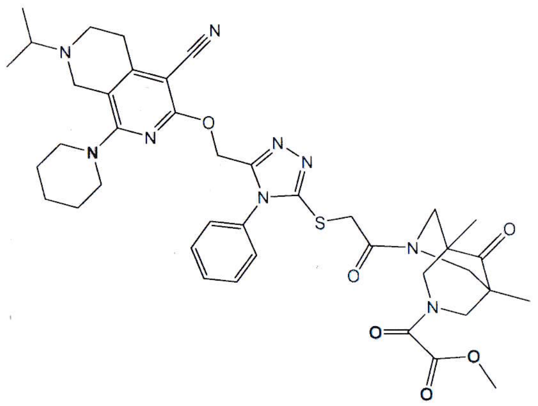

$C_{40}H_{49}N_9O_6S$

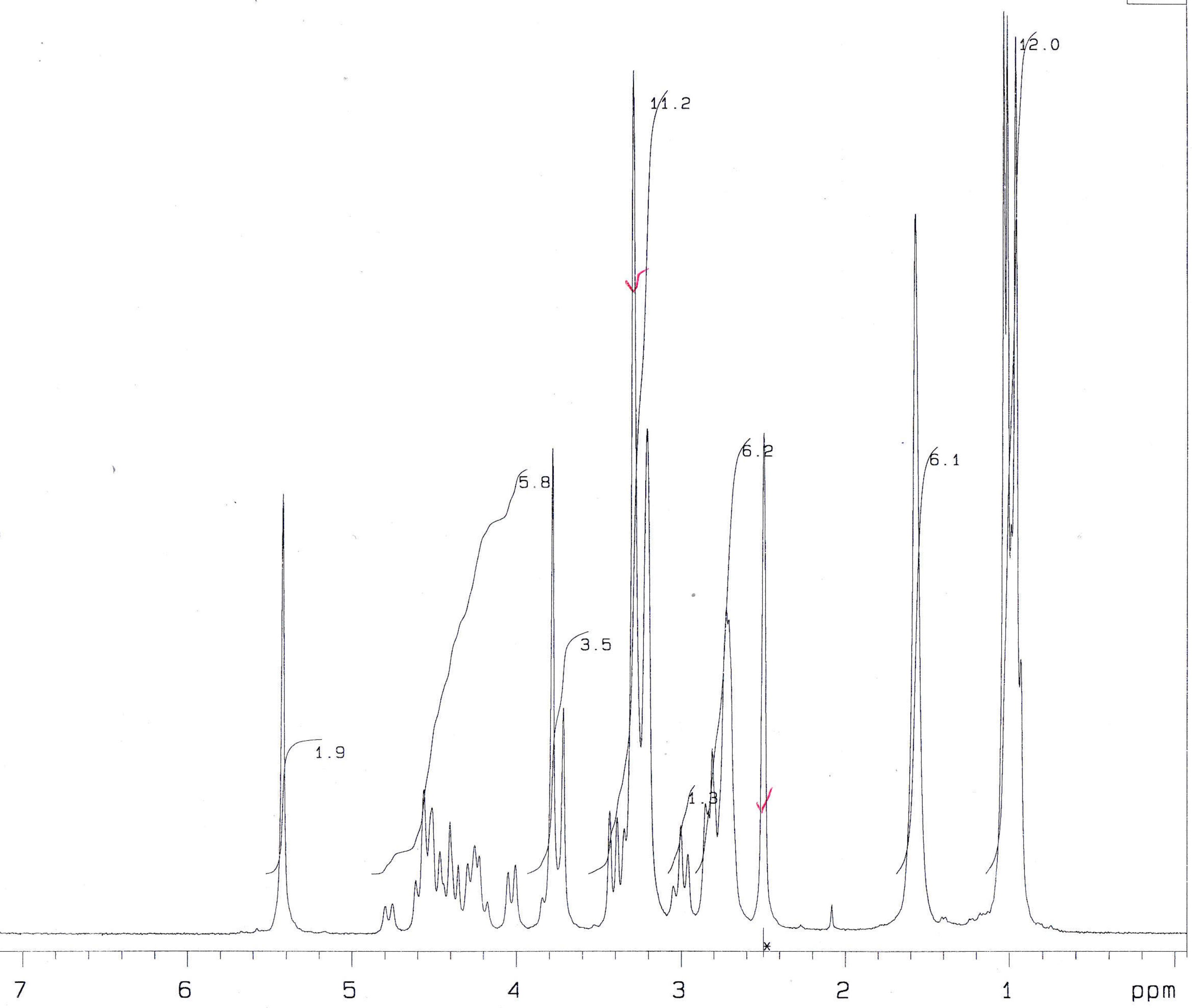

9b

Molecular Structure Research Centre, Yerevan, Armenia, Varian Mercury-300VX

C13 75.465 MHz, nt = 1056, np = 19998, temp = 30.0 C, lb = 1.0, solvent = DMSO

May 5 2022

NOCI_22 gm-018

GM-018

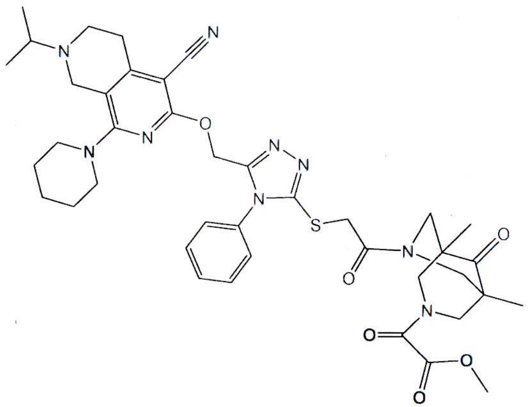

$C_{40}H_{49}N_9O_6S$

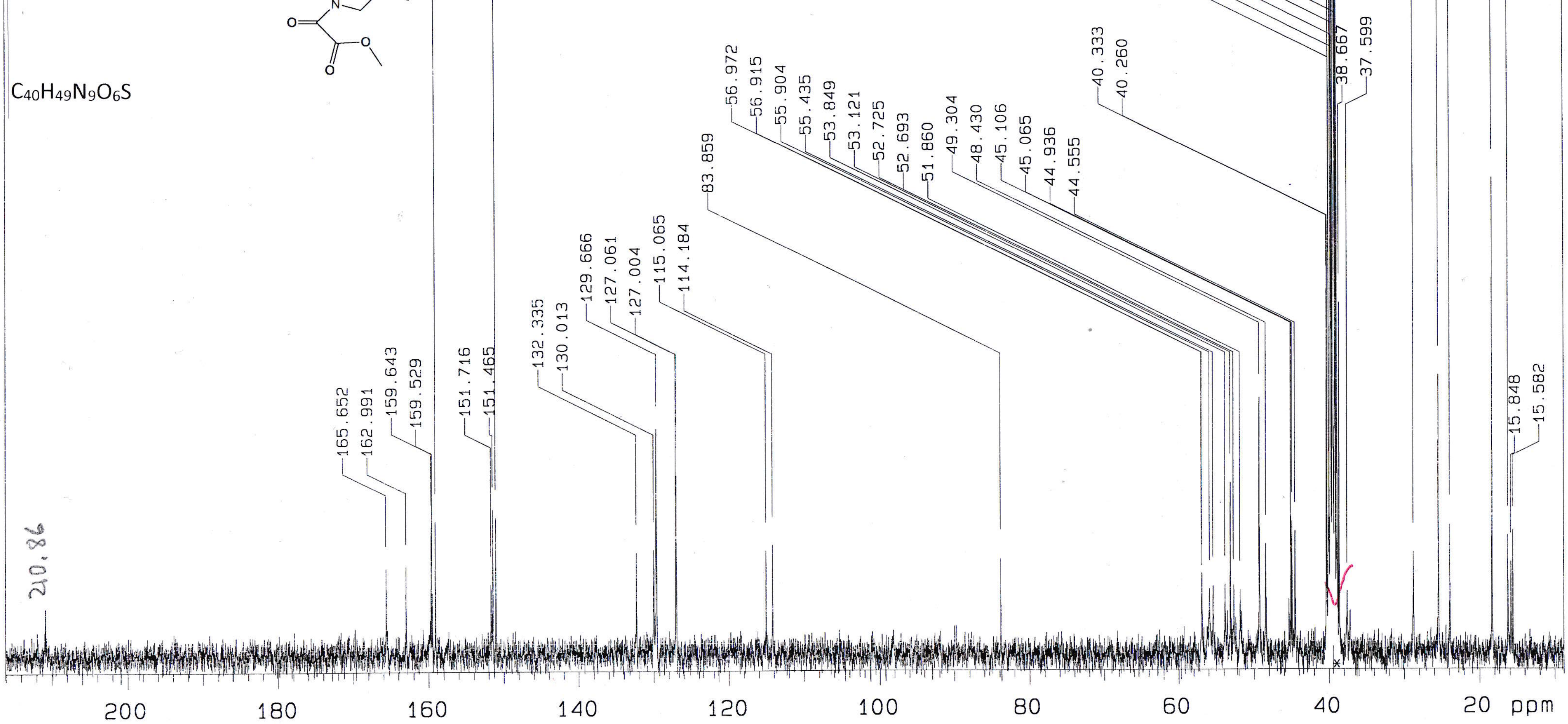

9c

Molecular Structure Research Centre, Yerevan, Armenia, Varian Mercury-300VX

H1 300.088 MHz, nt = 16, np = 32000, temp = 60.0 C, lb = -0.2, solvent = DMSO

Jan 25 2022

GM-002 - pure - dmso

NOCI_22 gm-002

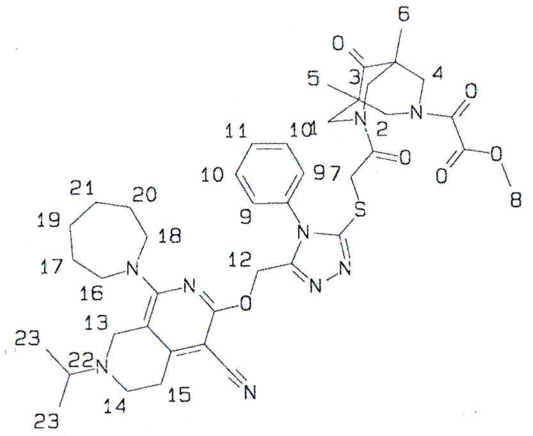

$C_{41}H_{51}N_9O_6S$

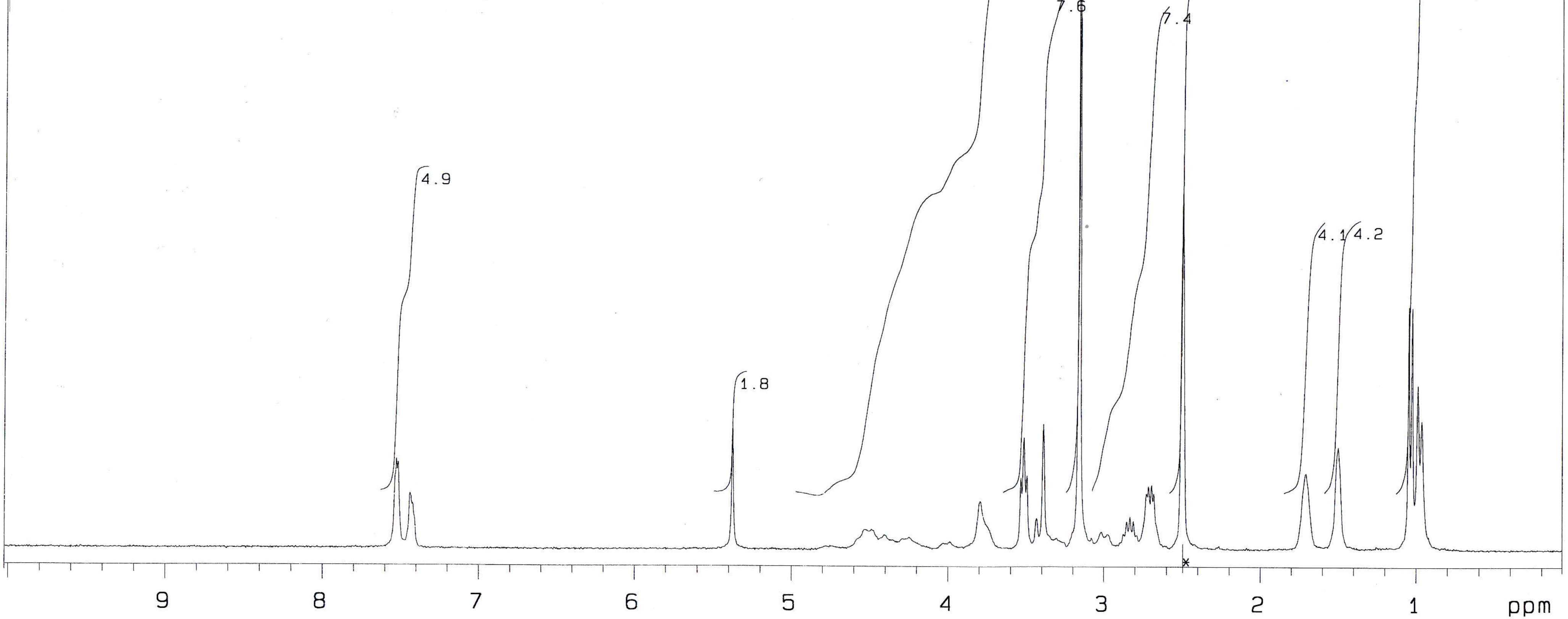

+ [illegible]

9d

Molecular Structure Research Centre, Yerevan, Armenia, Varian Mercury-300VX

H1 300.088 MHz, nt = 16, np = 32000, temp = 30.0 C, lb = -0.2, solvent = DMSO

Jun 16 2022

GM-027

NOCI_22 gm-027

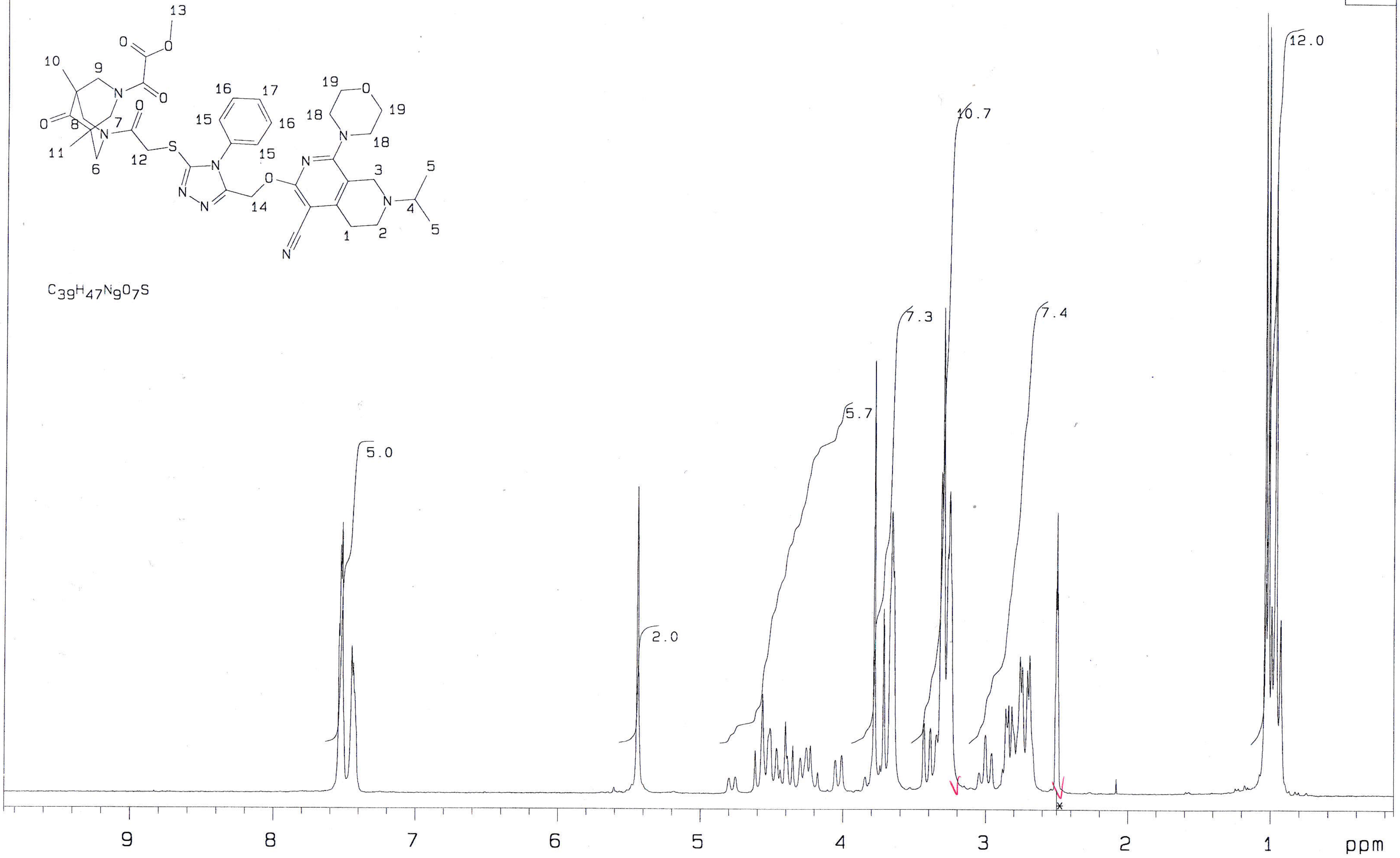

+

9d

Molecular Structure Research Centre, Yerevan, Armenia, Varian Mercury-300VX

C13 75.465 MHz, nt=528, np=19998, temp=30.0 C, lb=1.0, solvent=DMSO

Jun 16 2022

GM-027

NOCI_22 gm-027

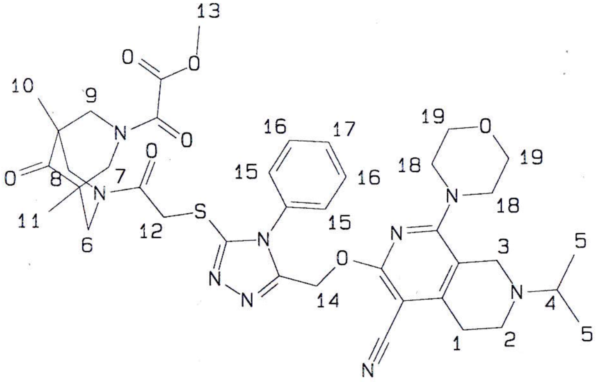

$C_{39}H_{47}N_9O_7S$

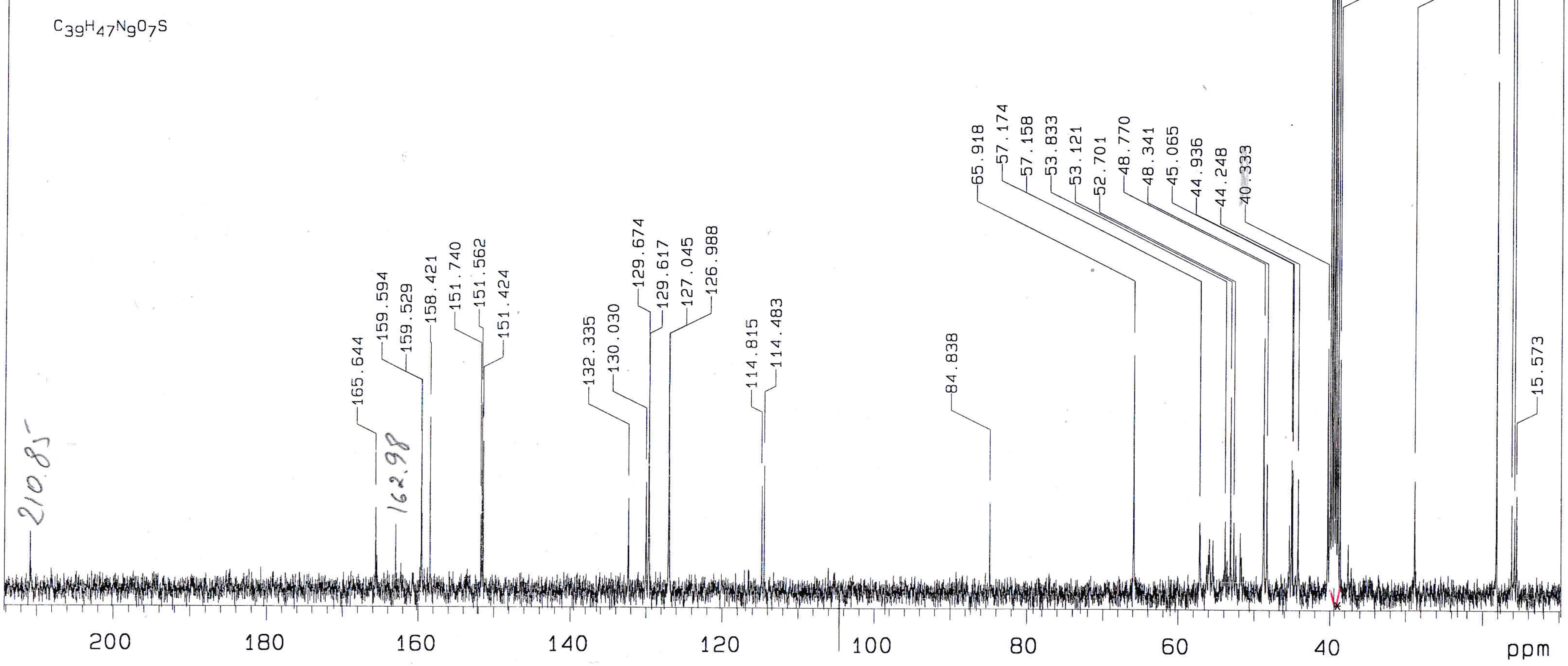

9e

Molecular Structure Research Centre, Yerevan, Armenia, Varian Mercury-300VX

H1 300.088 MHz, nt = 16, np = 32000, temp = 30.0 C, lb = -0.2, solvent = DMSO

Jun 1 2022

NOCI_22 gm-022

GM-022

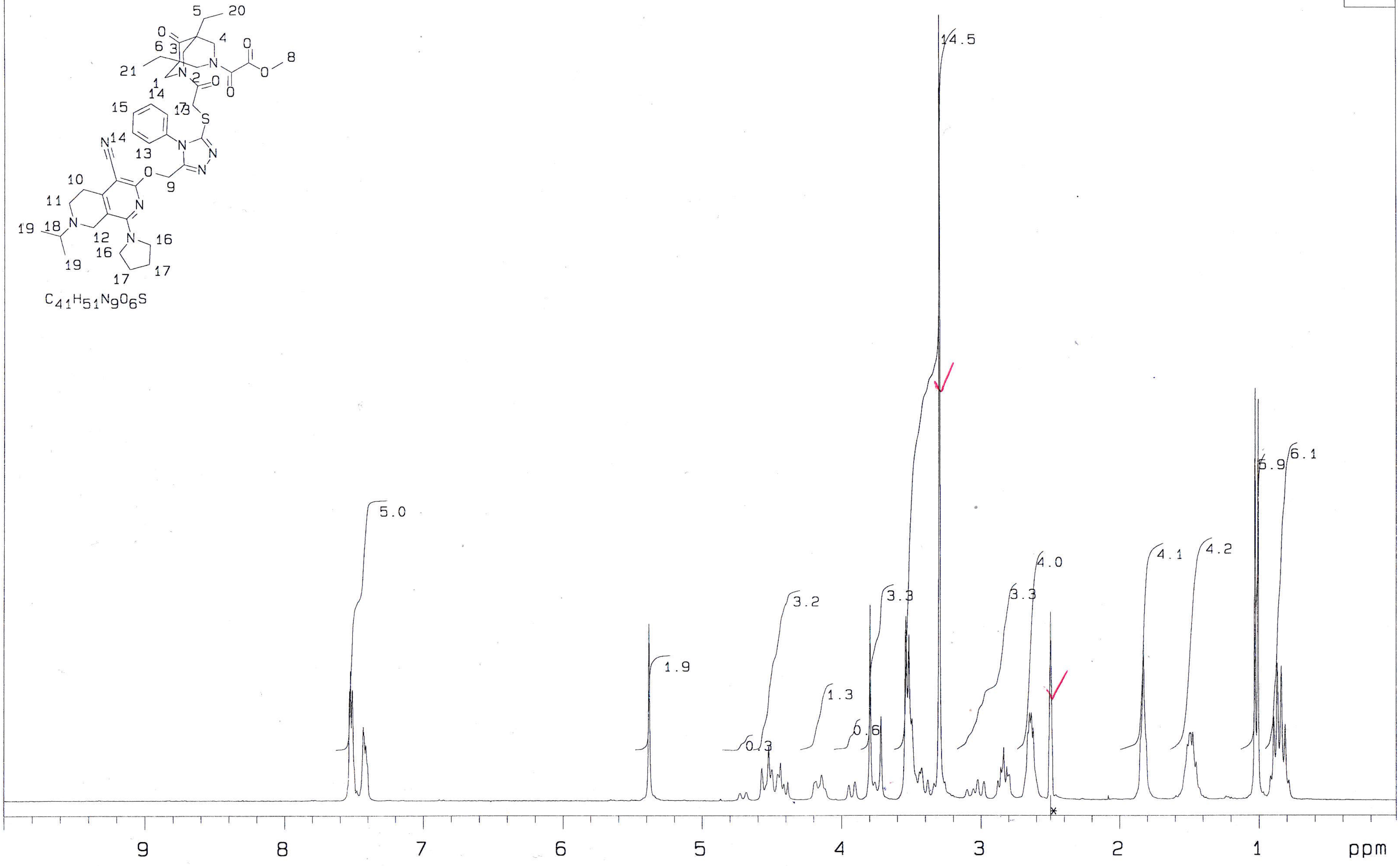

9f

Molecular Structure Research Centre, Yerevan, Armenia, Varian Mercury-300VX

H1 300.088 MHz, nt=16, np=32000, temp=30.0 C, lb=-0.2, solvent=DMSO/CCl4 1/3

Jun 17 2022

NOCI_22 gm-026

GM-026

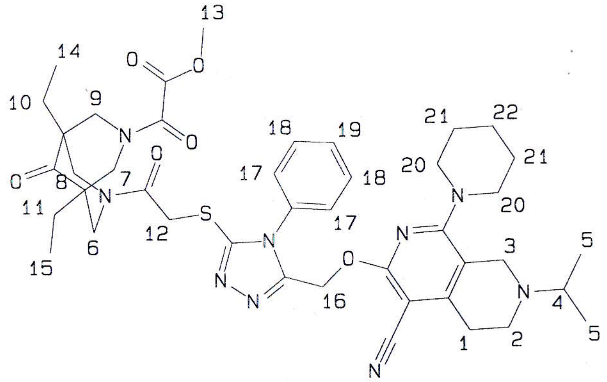

$C_{42}H_{53}N_9O_6S$

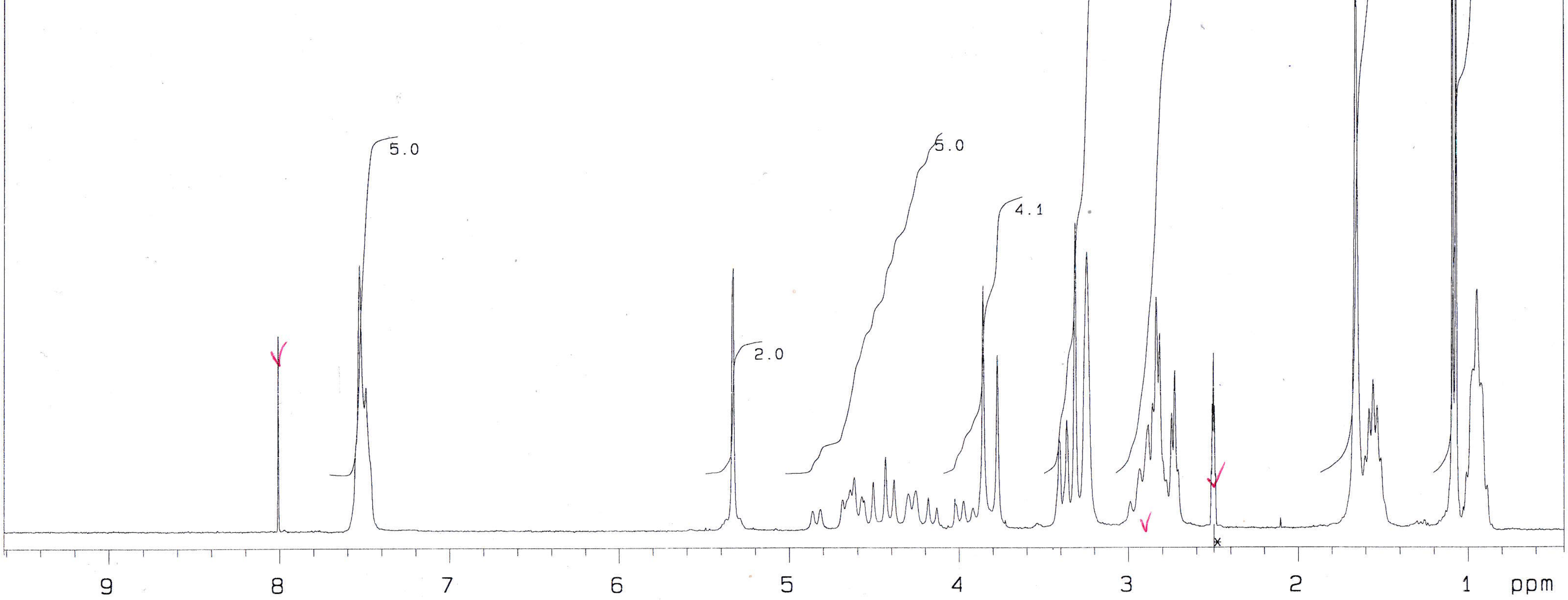

9g

Molecular Structure Research Centre, Yerevan, Armenia, Varian Mercury-300VX

H1 300.088 MHz, nt = 16, np = 32000, temp = 30.0 C, lb = -0.2, solvent = DMSO

Jun 9 2022

NOCI_22 gm-025

GM-025

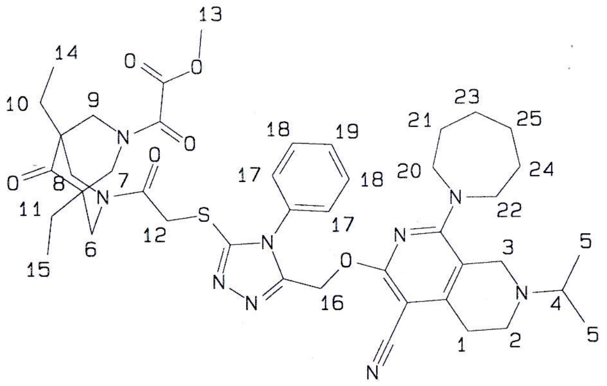

$C_{43}H_{55}N_9O_6S$

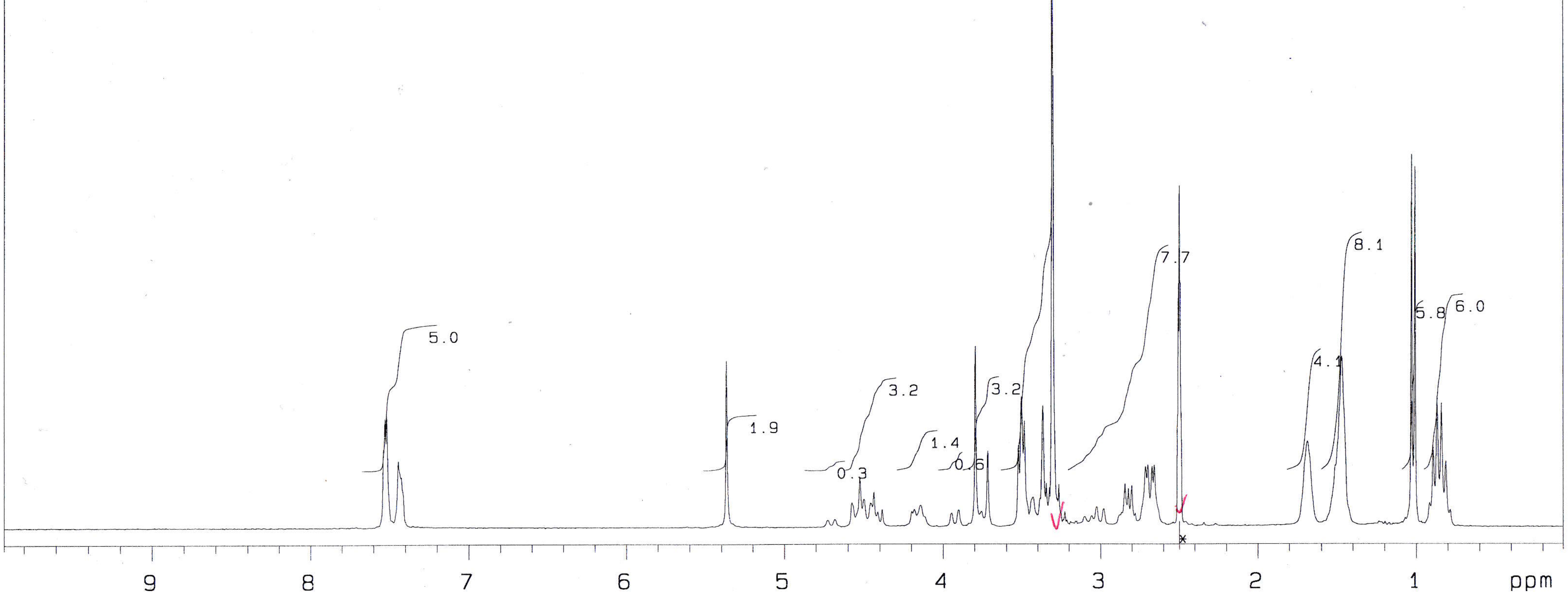

+ [illegible]

9

Molecular Structure Research Centre, Yerevan, Armenia, Varian Mercury-300VX

C13 75.465 MHz, nt = 2304, np = 19998, temp = 30.0 C, lb = 1.0, solvent = DMSO

Jun 9 2022

NOCI_22 gm-025

GM-025

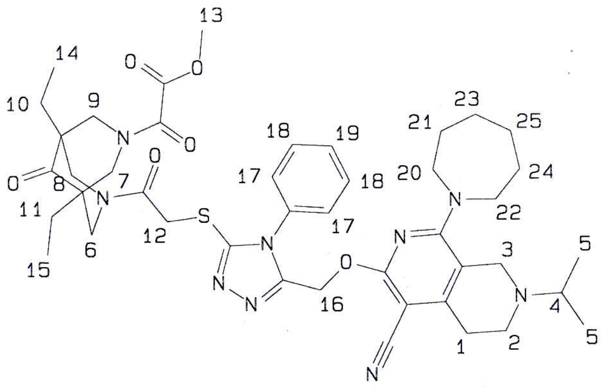

$C_{43}H_{55}N_9O_6S$

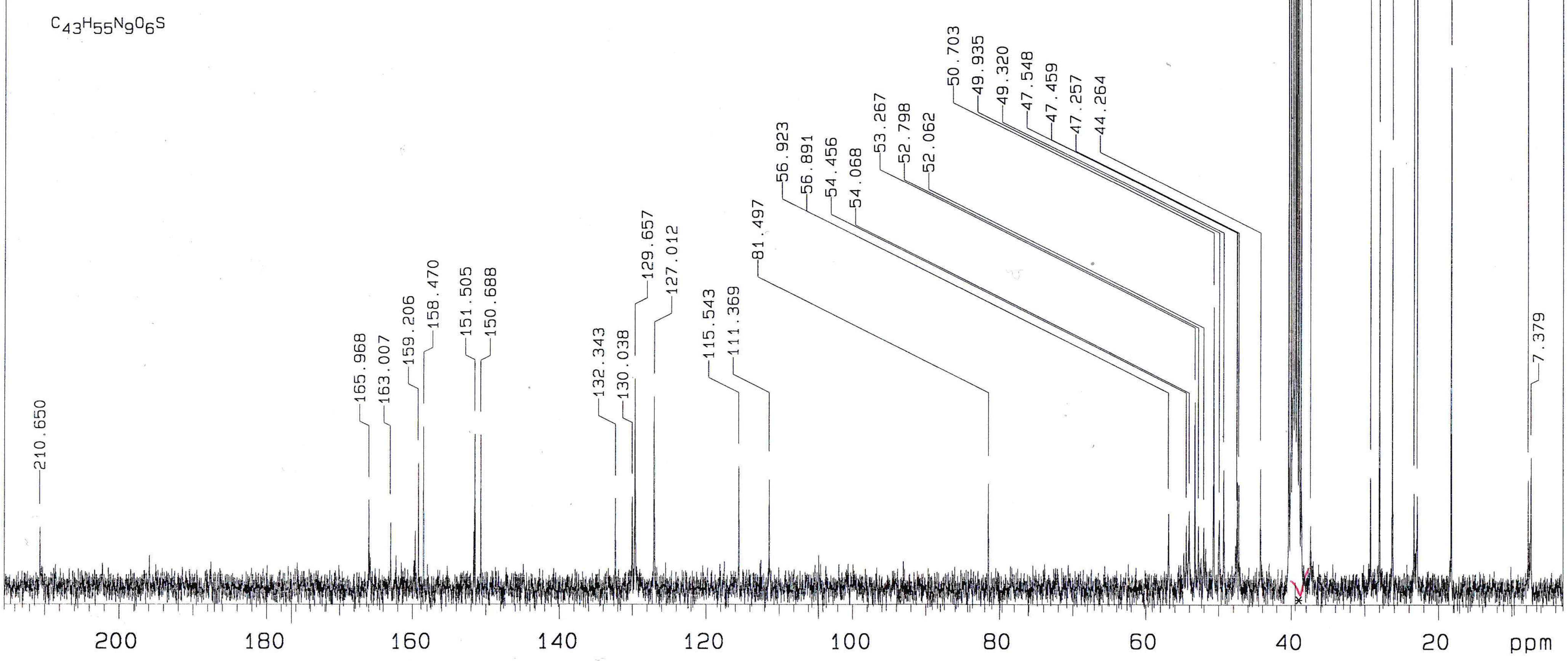

+ [signature]

9h

Molecular Structure Research Centre, Yerevan, Armenia, Varian Mercury-300VX

H1 300.088 MHz, nt = 16, np = 32000, temp = 30.0 C, lb = -0.2, solvent = DMSO/CCl4 1/3

Nov 15 2022

NOCI_22 gm-029

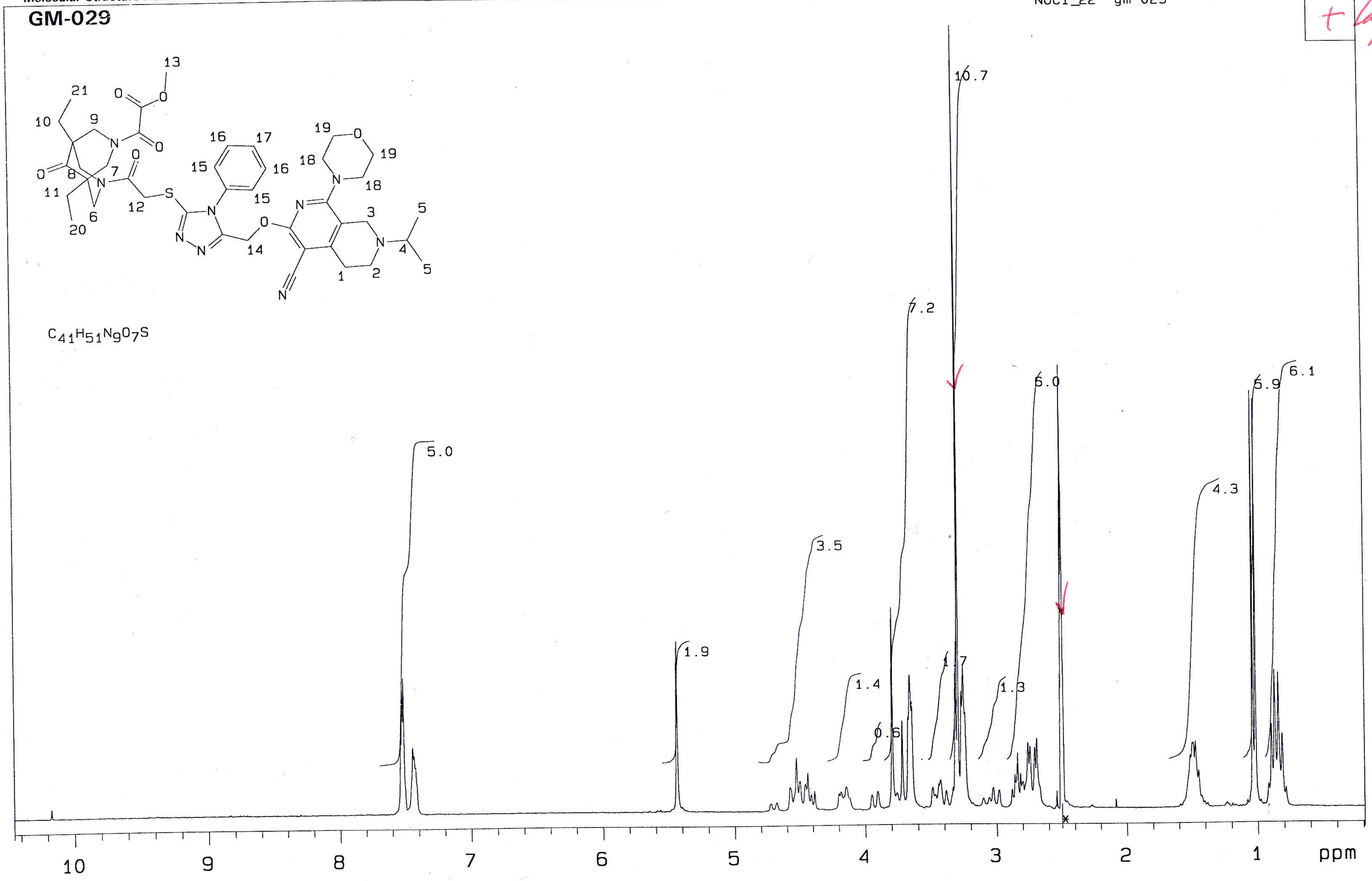

9i

Molecular Structure Research Centre, Yerevan, Armenia, Varian Mercury-300VX

H1 300.088 MHz, nt = 16, np = 32000, temp = 30.0 C, lb = -0.2, solvent = DMSO/CCl4 1/3

Oct 13 2023

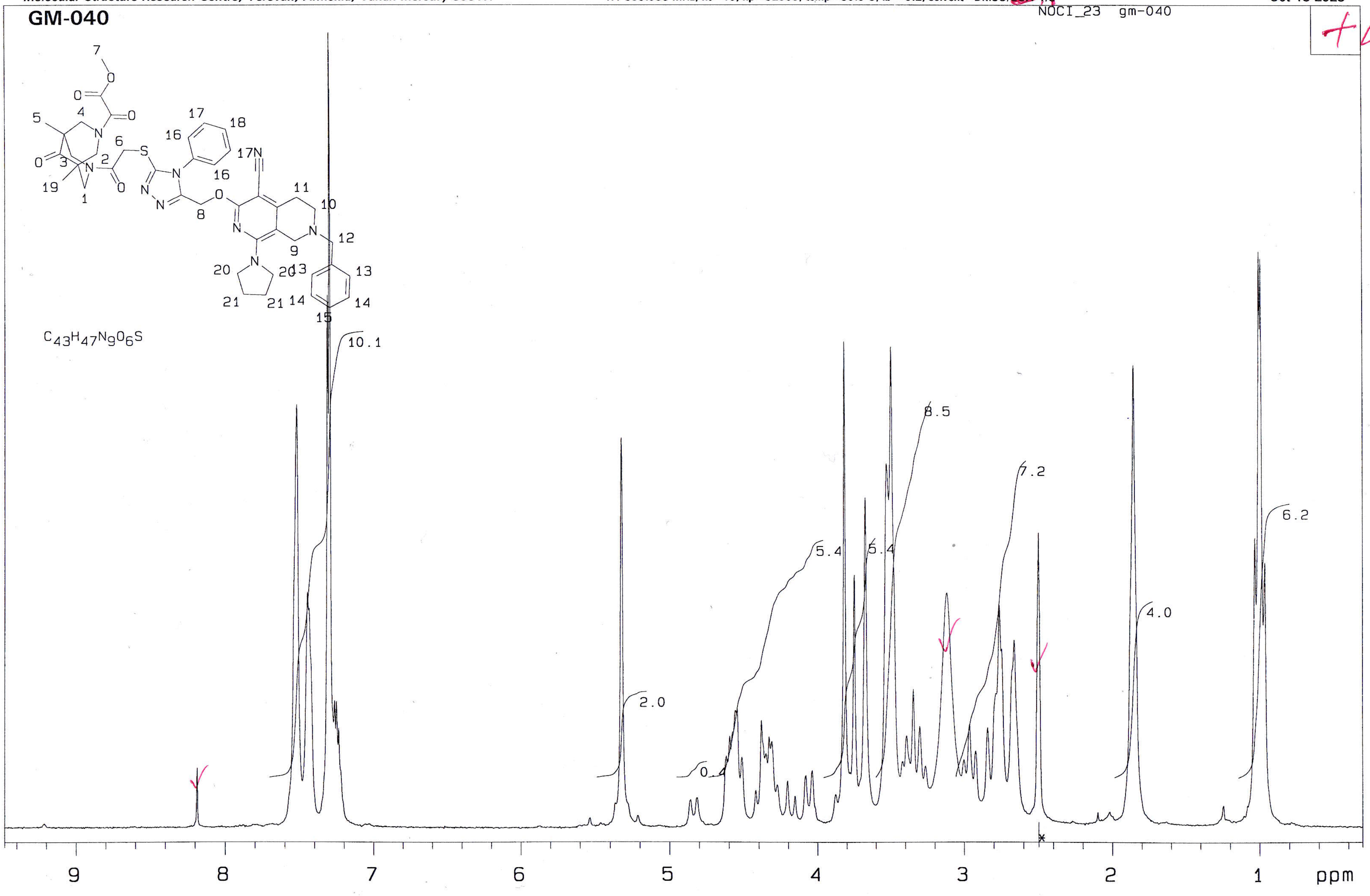

9j

Molecular Structure Research Centre, Yerevan, Armenia, Varian Mercury-300VX

H1 300.088 MHz, nt = 16, np = 32000, temp = 30.0 C, lb = -0.2, solvent = DMSO/CCl4 1/3

Jun 8 2023

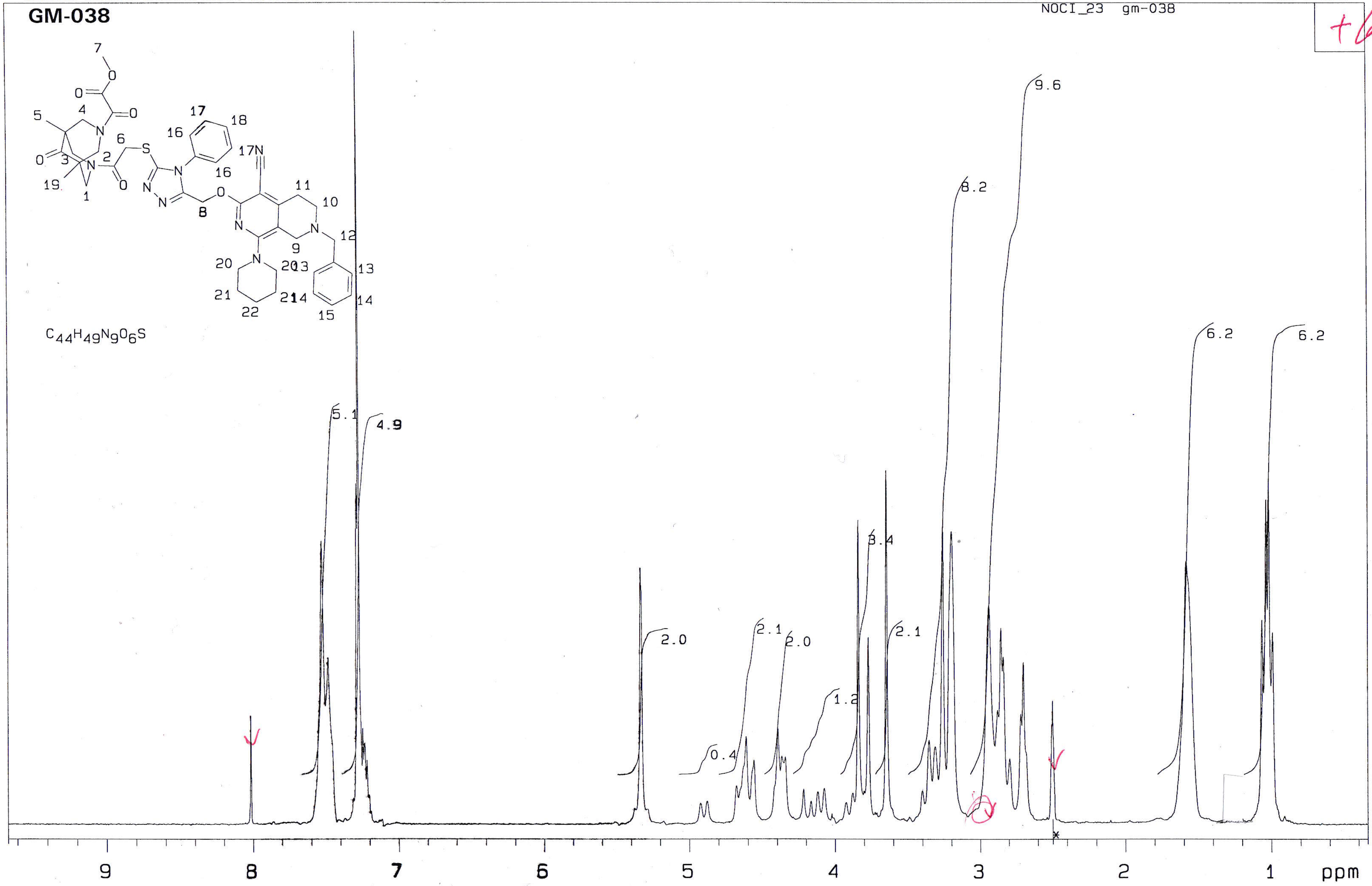

9k

* DMFA

Molecular Structure Research Centre, Yerevan, Armenia, Varian Mercury-300VX

H1 300.088 MHz, nt=16, np=32000, temp=30.0 C, lb=-0.2, solvent=DMSO/~~CCl4 1/3~~

Apr 28 2023

NOCI_23 gm-035

GM-035

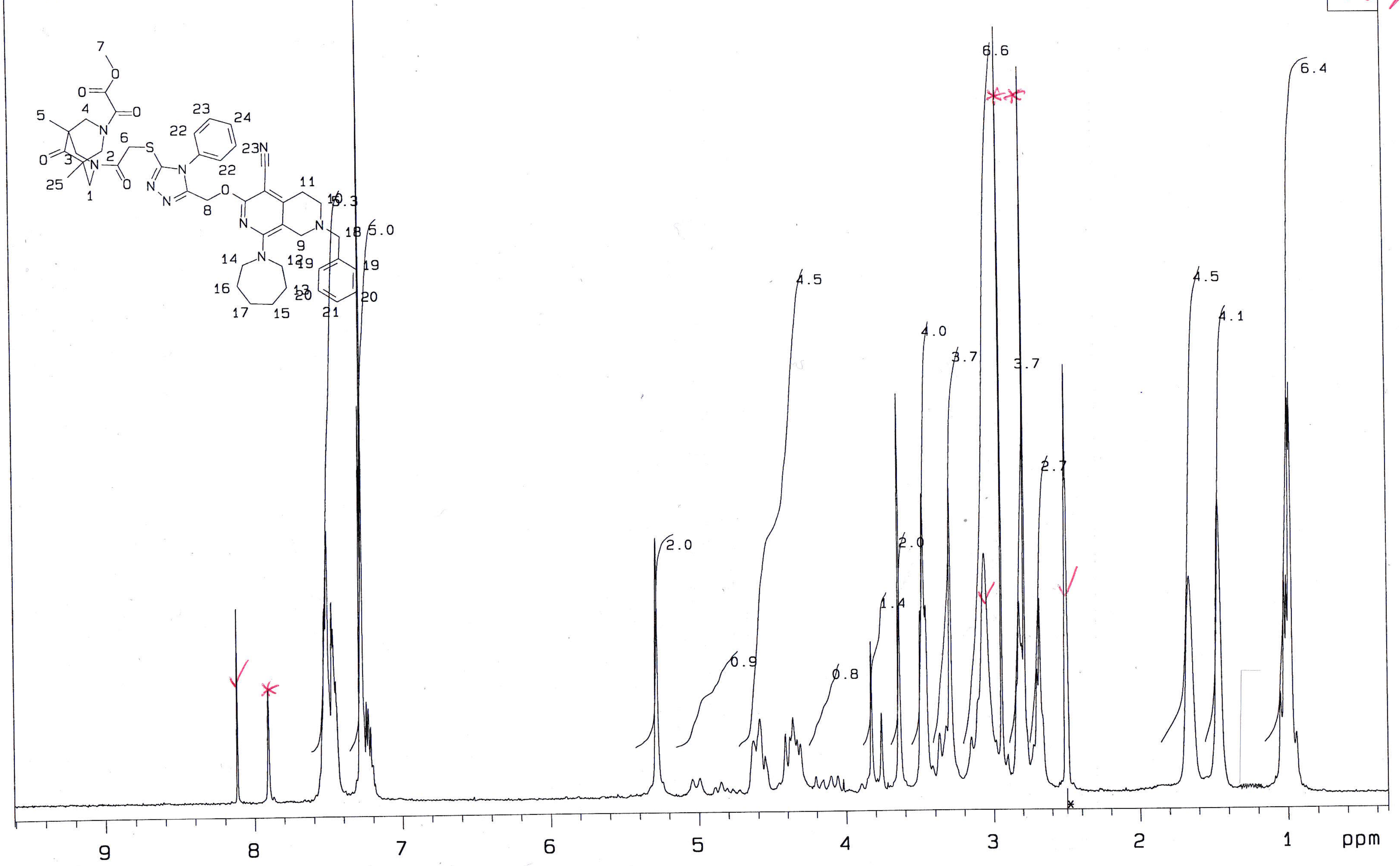

9l

Molecular Structure Research Centre, Yerevan, Armenia, Varian Mercury-300VX

H1 300.088 MHz, nt = 16, np = 32000, temp = 30.0 C, lb = -0.2, solvent = DMSO

Feb 23 2024

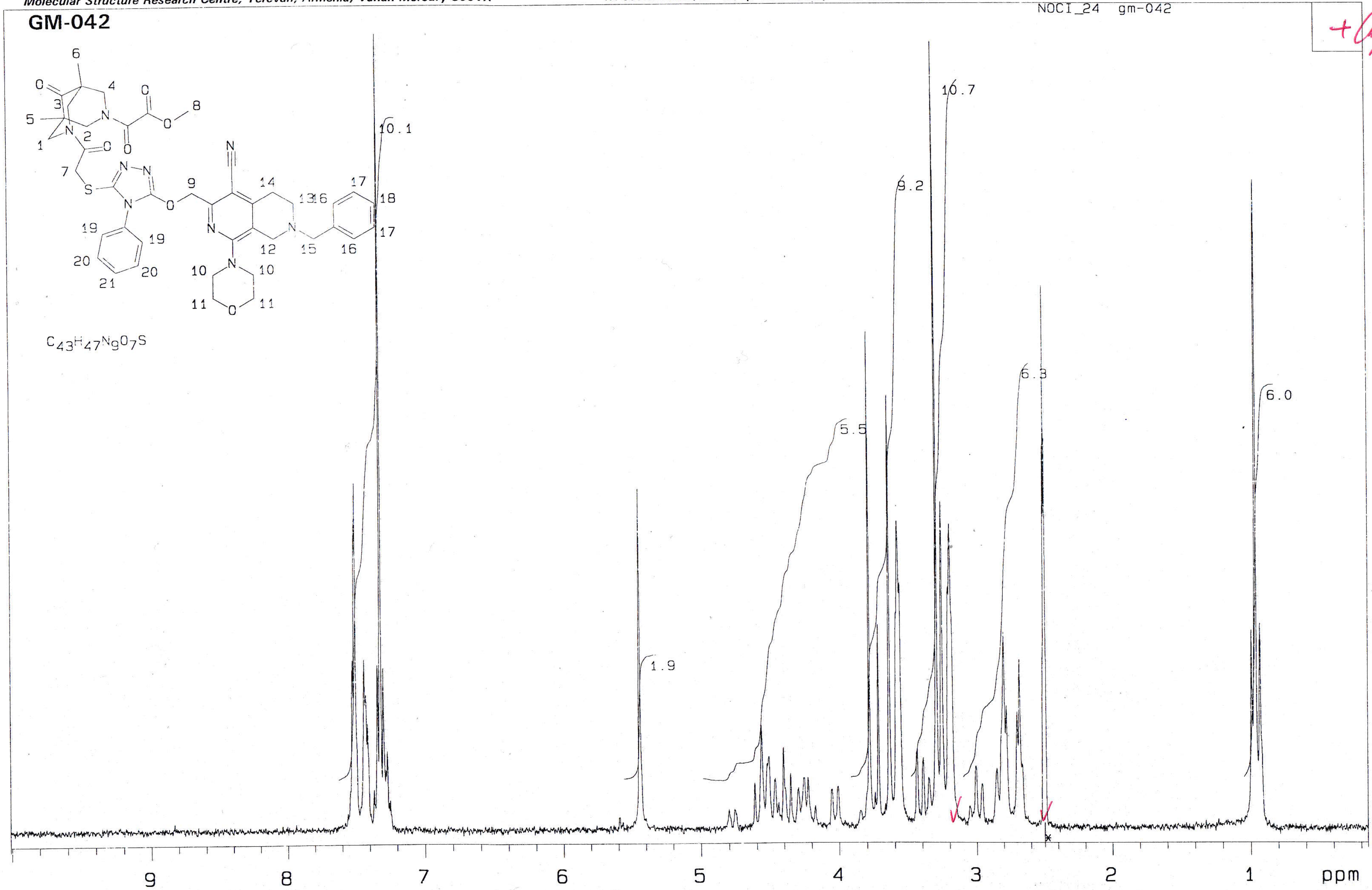

9m

Molecular Structure Research Centre, Yerevan, Armenia, Varian Mercury-300VX

H1 300.088 MHz, nt=16, np=32000, temp=30.0 C, lb=-0.2, solvent=DMSO/~~CCl4 1/3~~

Oct 30 2023

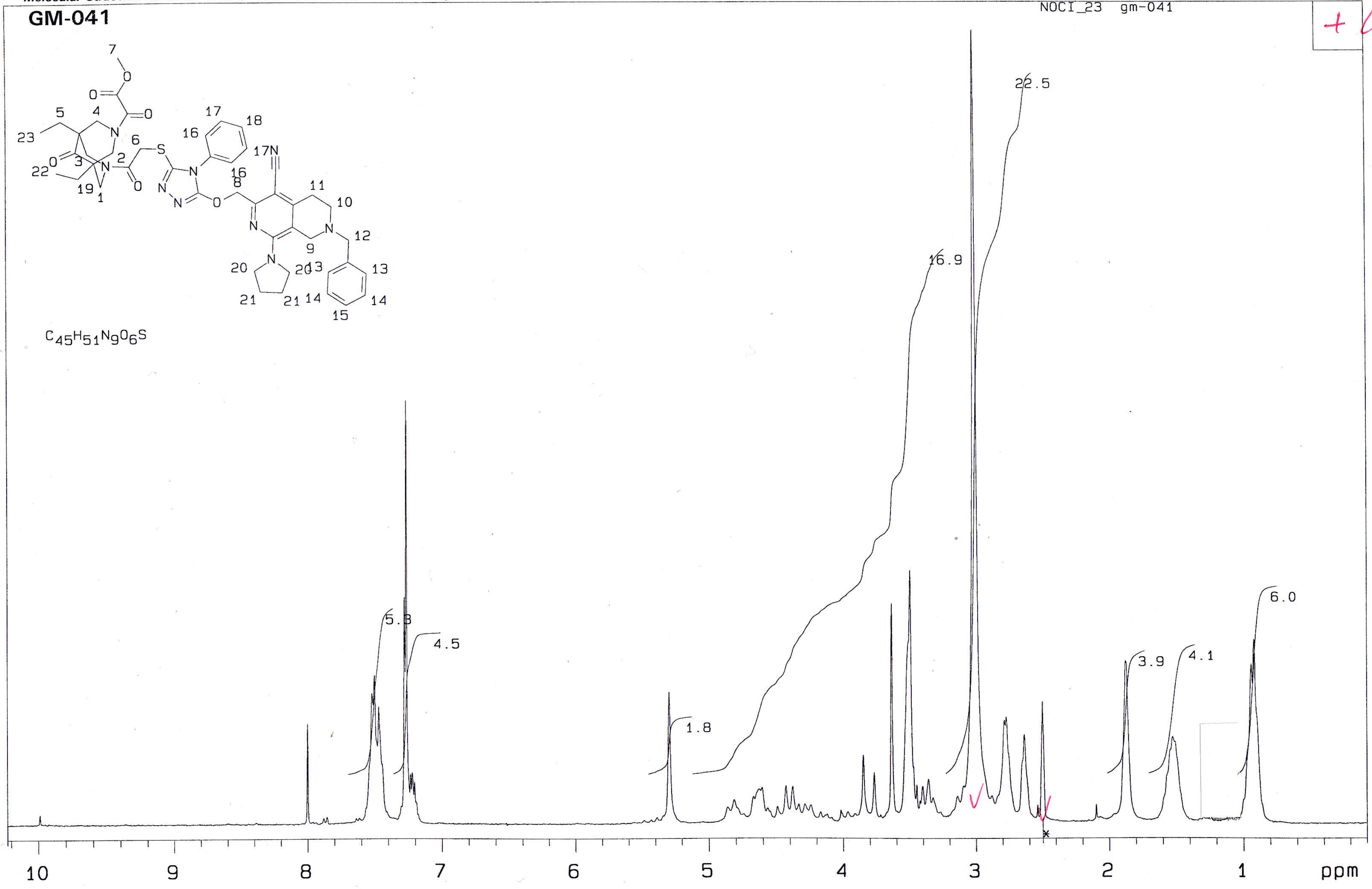

9n

Molecular Structure Research Centre, Yerevan, Armenia, Varian Mercury-300VX

H1 300.088 MHz, nt=16, np=32000, temp=30.0 C, lb=-0.2, solvent=DMSO/~~CCl4 1/3~~

Jul 3 2023

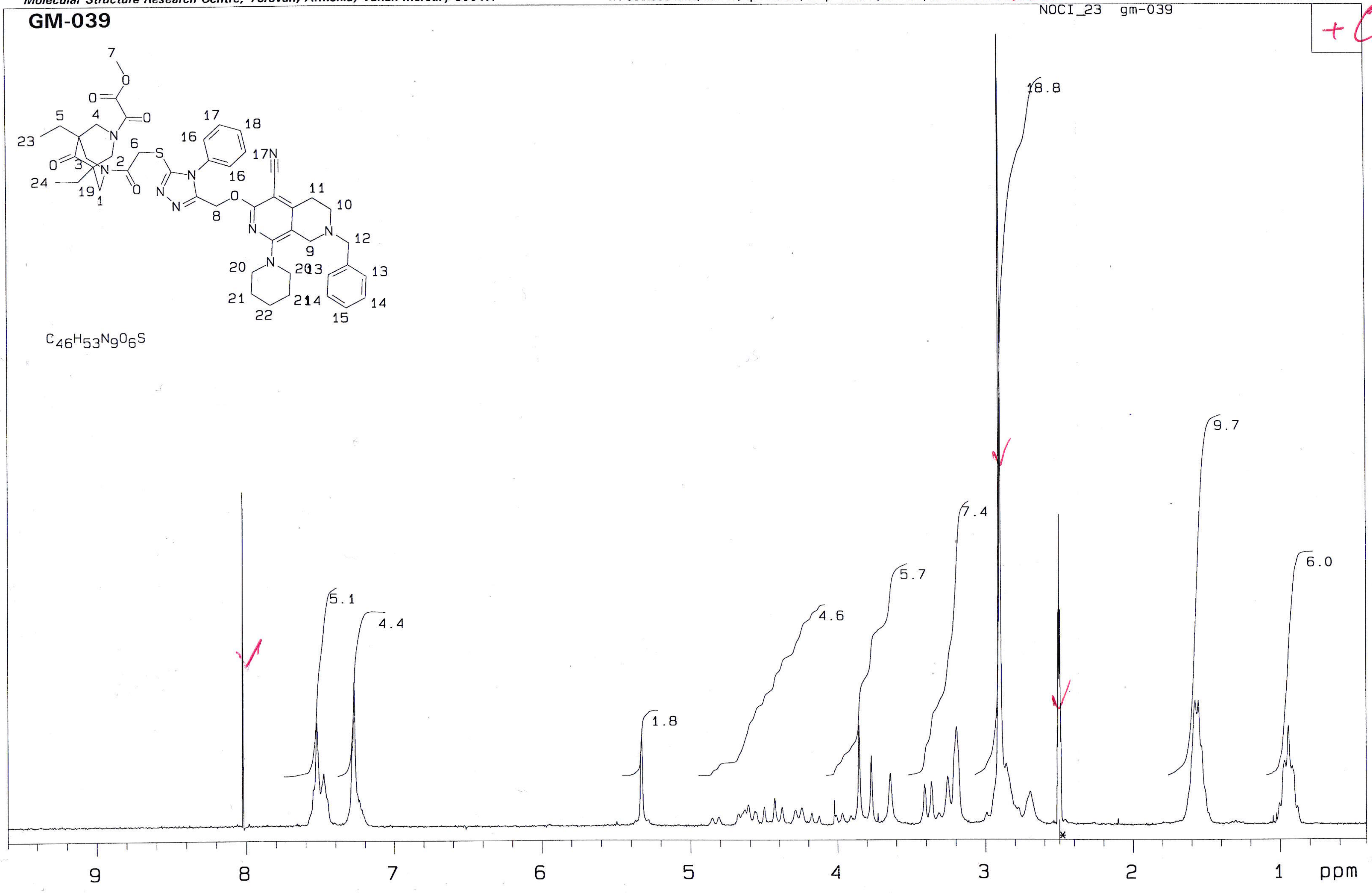

Molecular Structure Research Center, NAS RA, Yerevan, Armenia

90

June 4, 2025 5:03:07 PM AMT

Avance 400 Neo
TopSpin 4.1.3

gm-048, 1H, CDCl3, temp=30

gm-048 1 1 F:/FID_BRUKER/NOCl_25

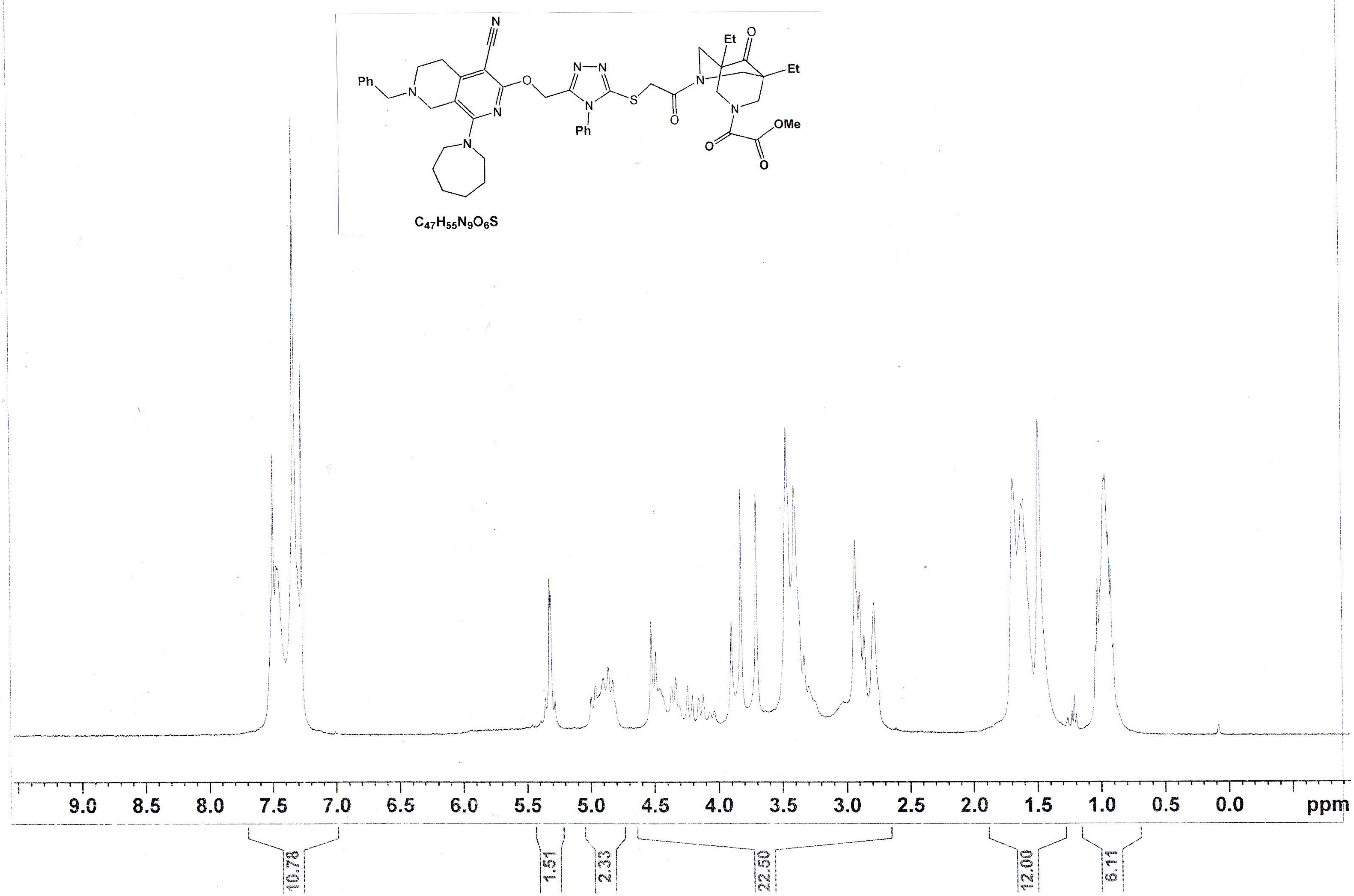

9p

Molecular Structure Research Centre, Yerevan, Armenia, Varian Mercury-300VX

H1 300.088 MHz, nt = 16, np = 32000, temp = 30.0 C, lb = -0.2, solvent = DMSO/[illegible]

Mar 14 2024

NOCI_24 gm-043

GM-043

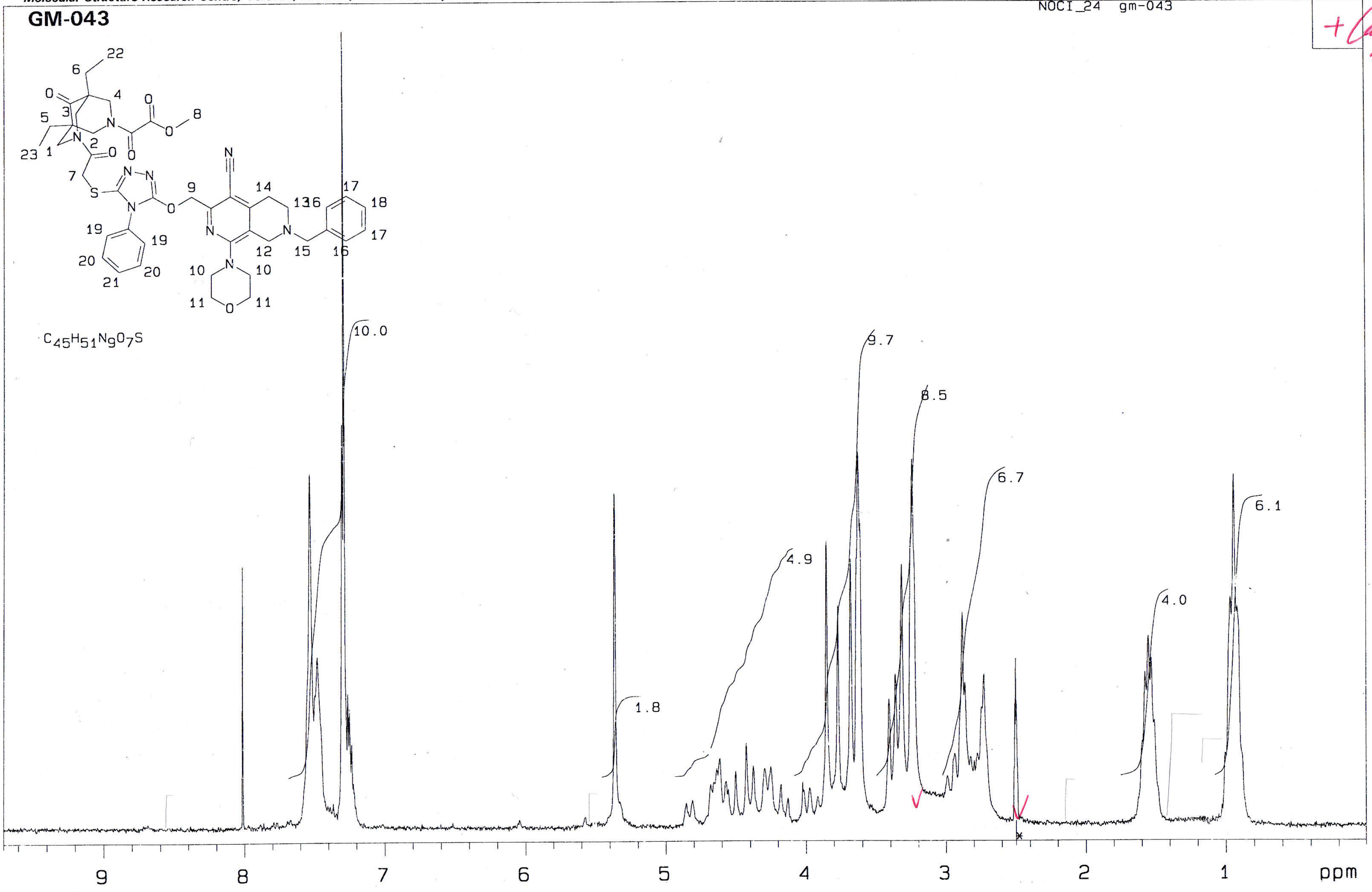

Compound 9a

12.09.2025

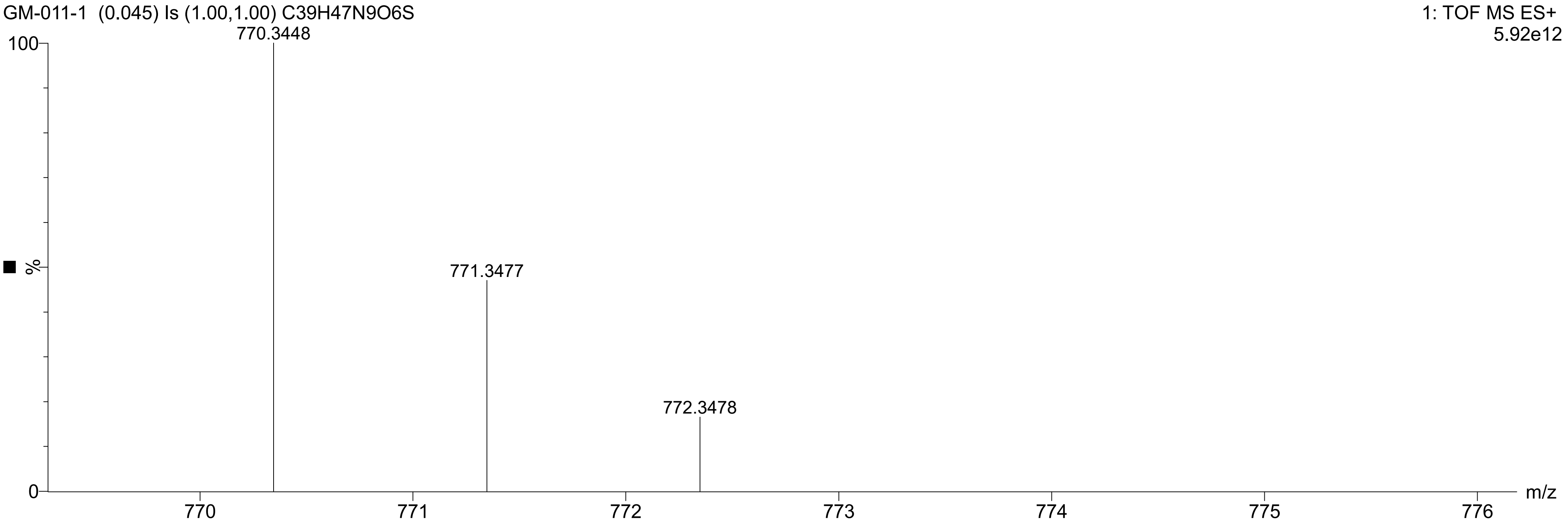

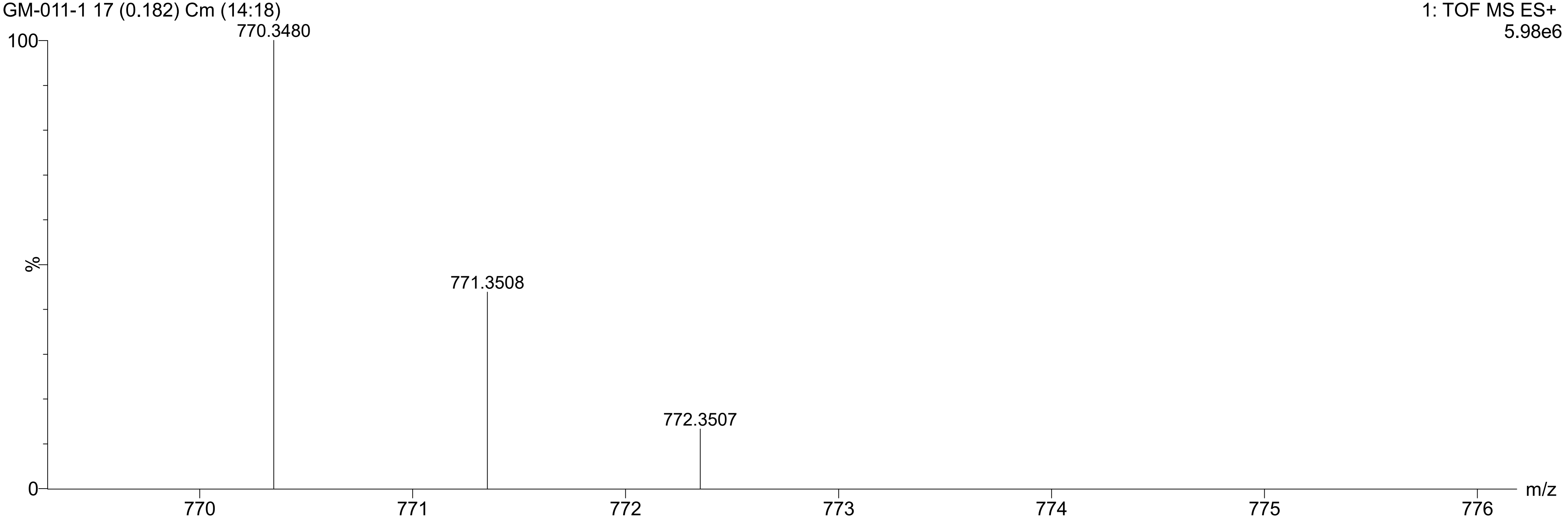

Compound 9b

19.09.2025

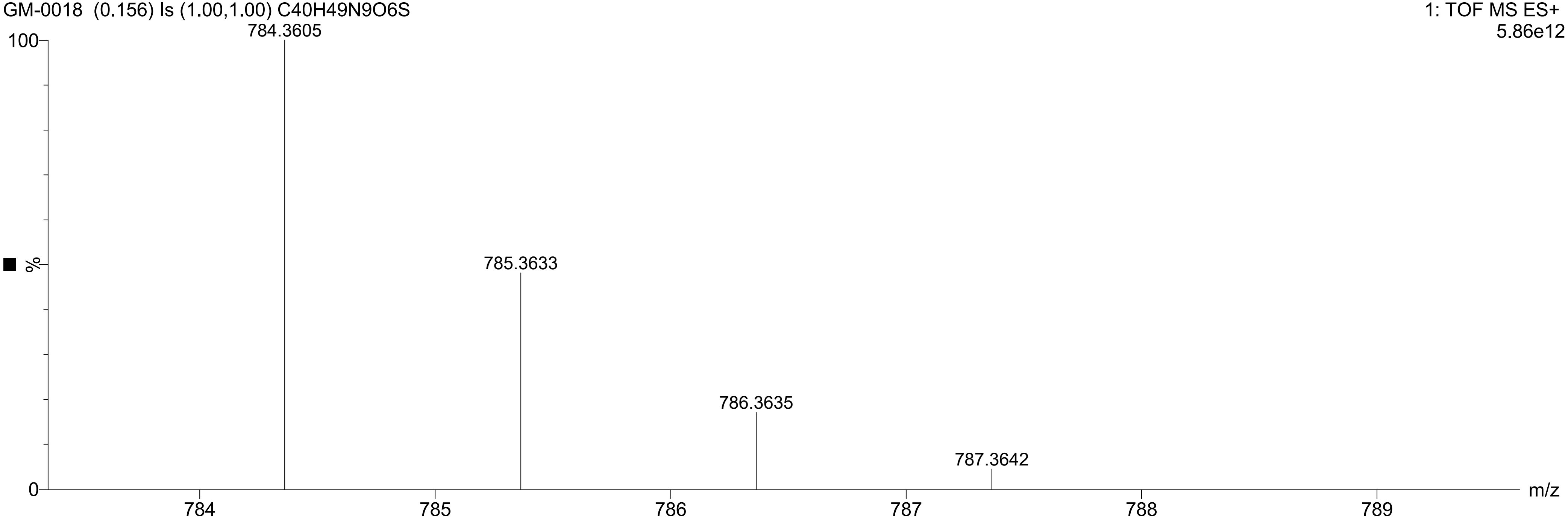

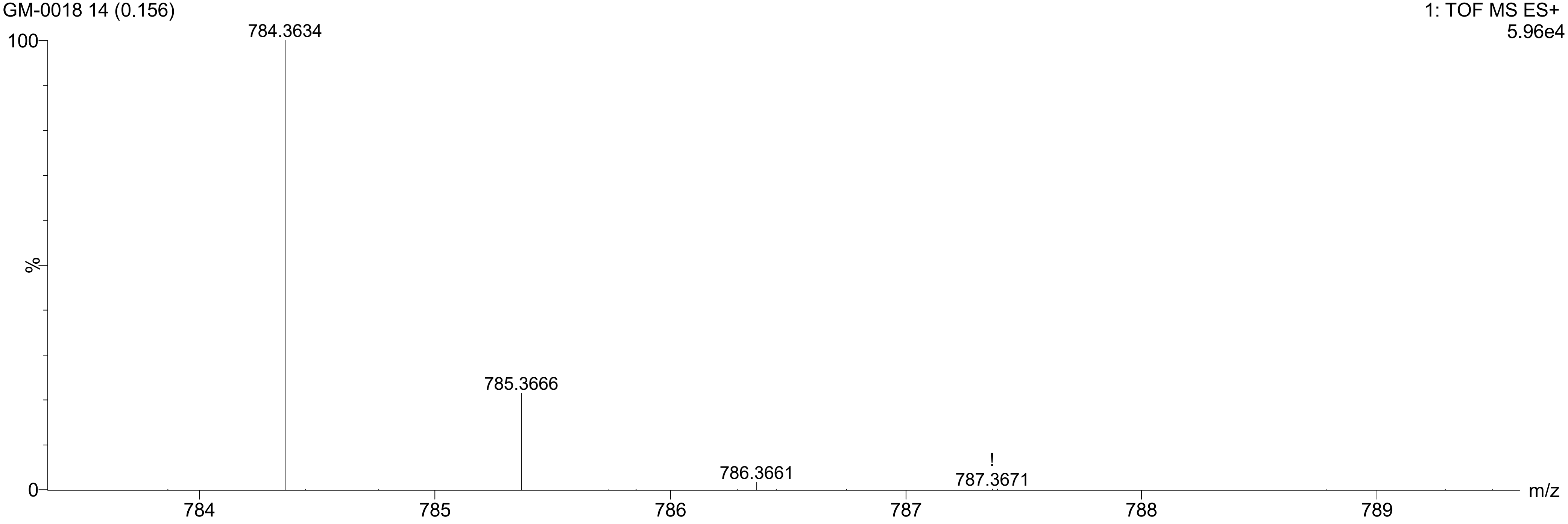

Compound 9c

7.04.2025

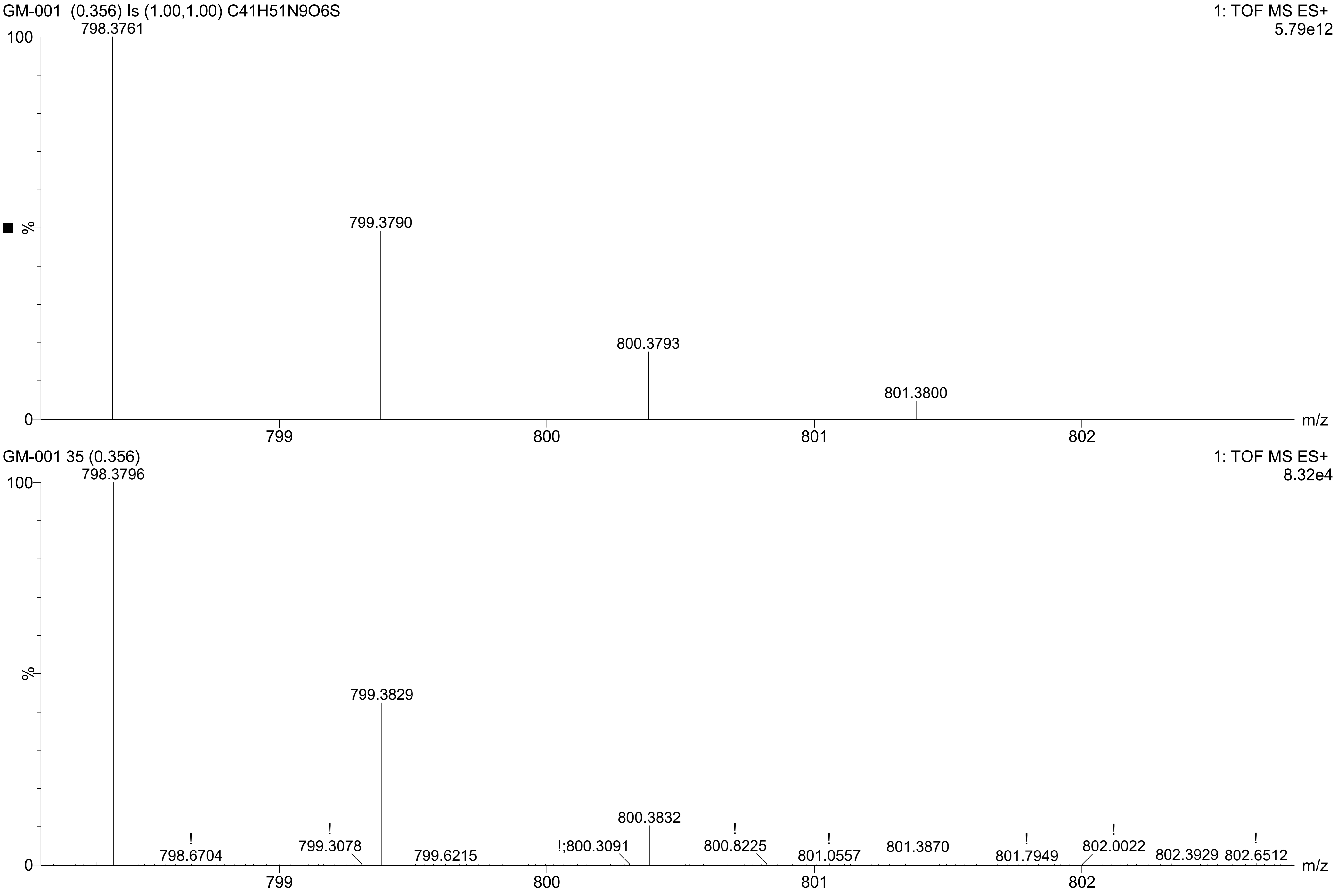

Compound 9d

05.09.2025

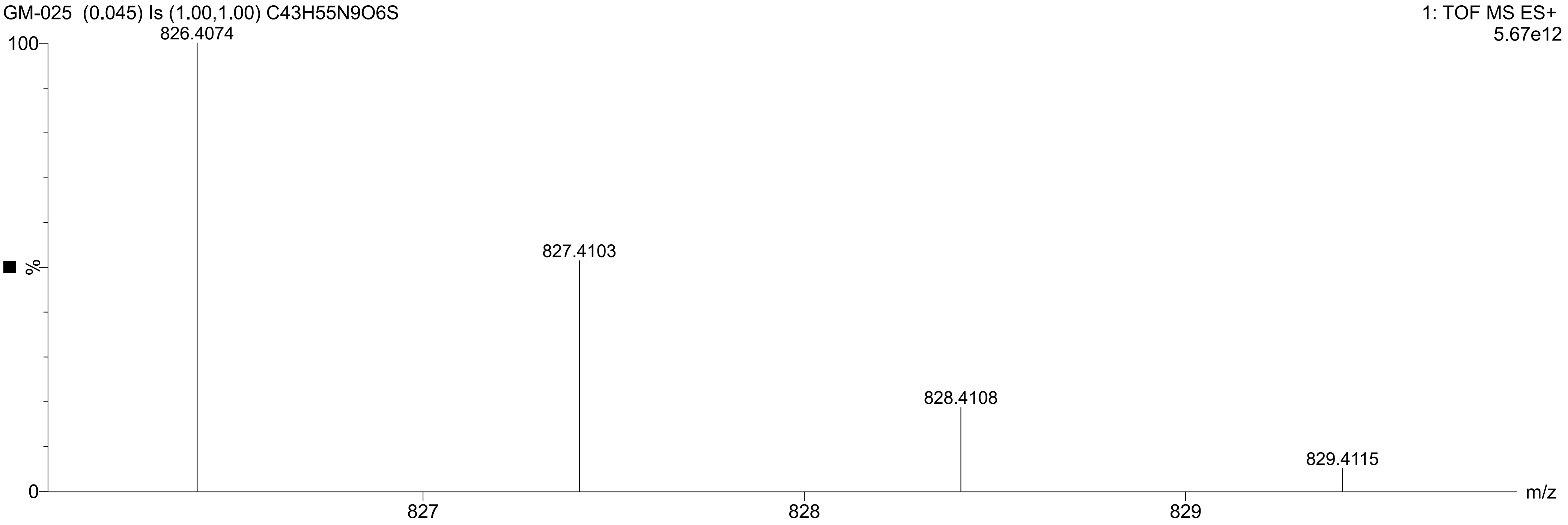

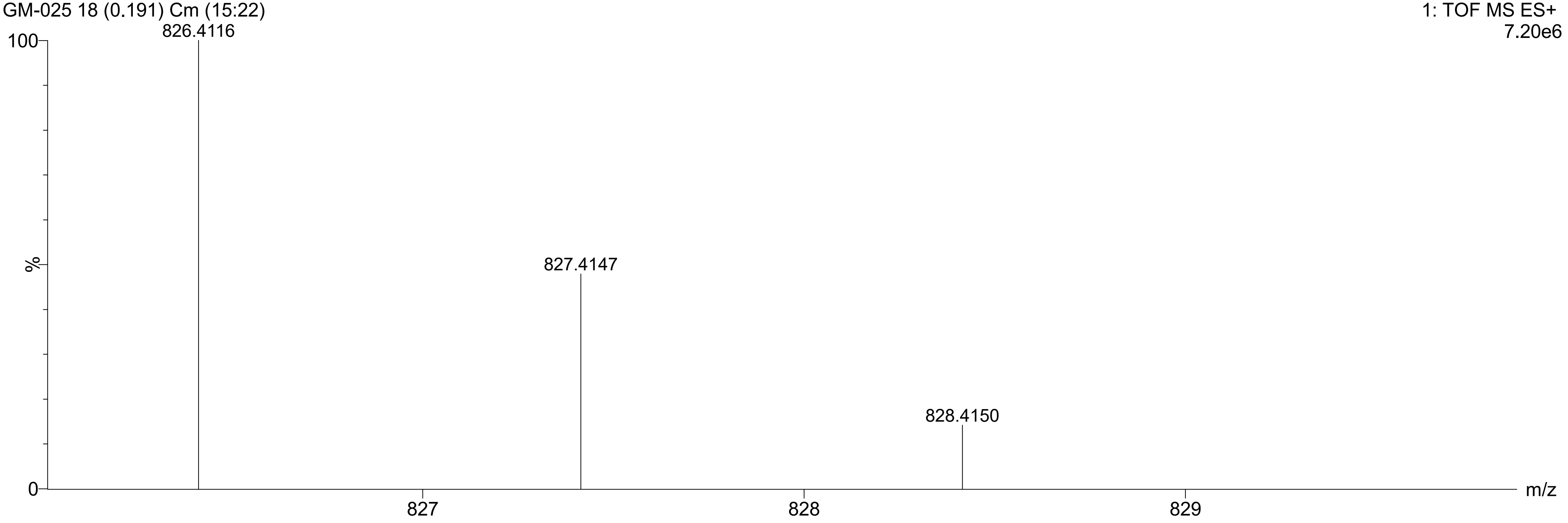

Compound 9e

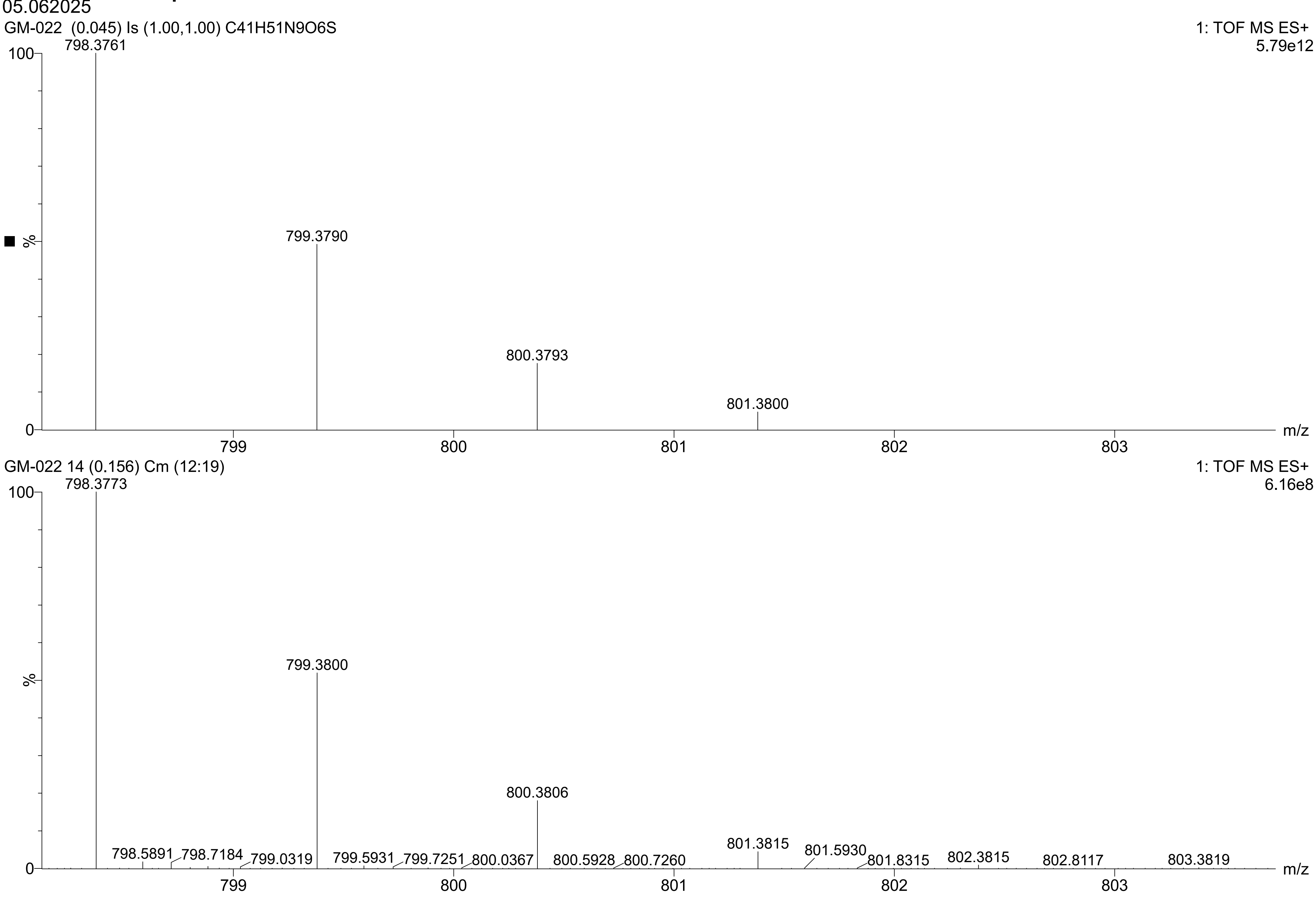
05.062025
GM-022 (0.045) Is (1.00,1.00) C41H51N9O6S
1: TOF MS ES+
5.79e12
798.3761
799.3790
800.3793
801.3800
100
%
0
799
800
801
802
803
m/z
GM-022 14 (0.156) Cm (12:19)
1: TOF MS ES+
6.16e8
798.3773
799.3800
800.3806
801.3815
798.5891
798.7184
799.0319
799.5931
799.7251
800.0367
800.5928
800.7260
801.5930
801.8315
802.3815
802.8117
803.3819

Compound 9f

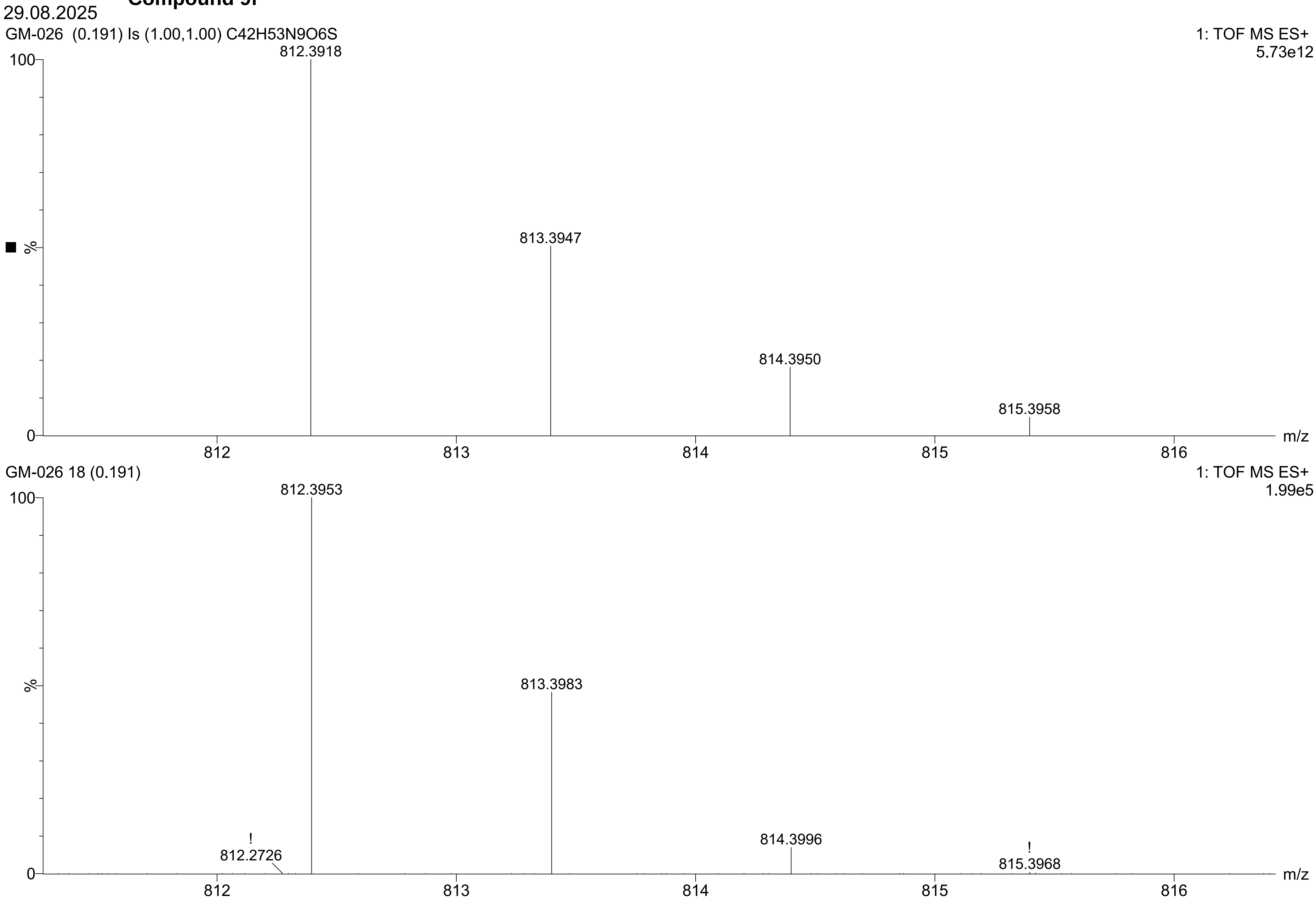
29.08.2025
GM-026 (0.191) Is (1.00,1.00) C42H53N9O6S
1: TOF MS ES+
5.73e12
812.3918
813.3947
814.3950
815.3958
100
%
0
812
813
814
815
816
m/z
GM-026 18 (0.191)
1: TOF MS ES+
1.99e5
812.3953
813.3983
814.3996
812.2726
815.3968
100
%
0
812
813
814
815
816
m/z

Compound 9g

05.09.2025

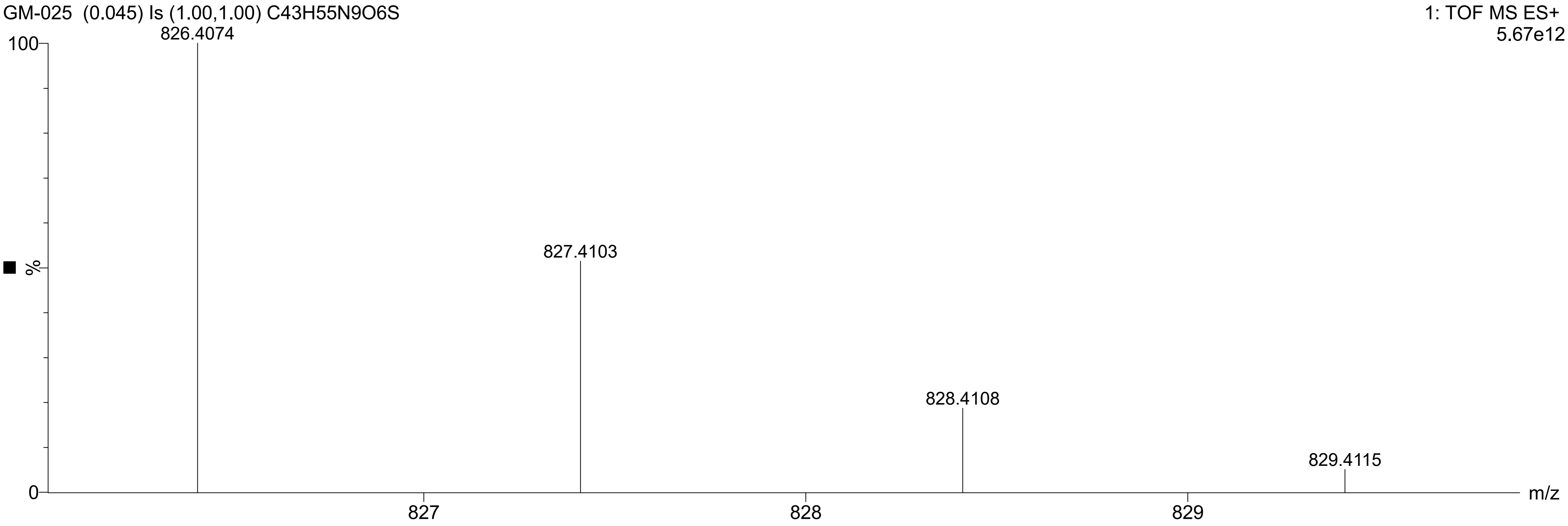

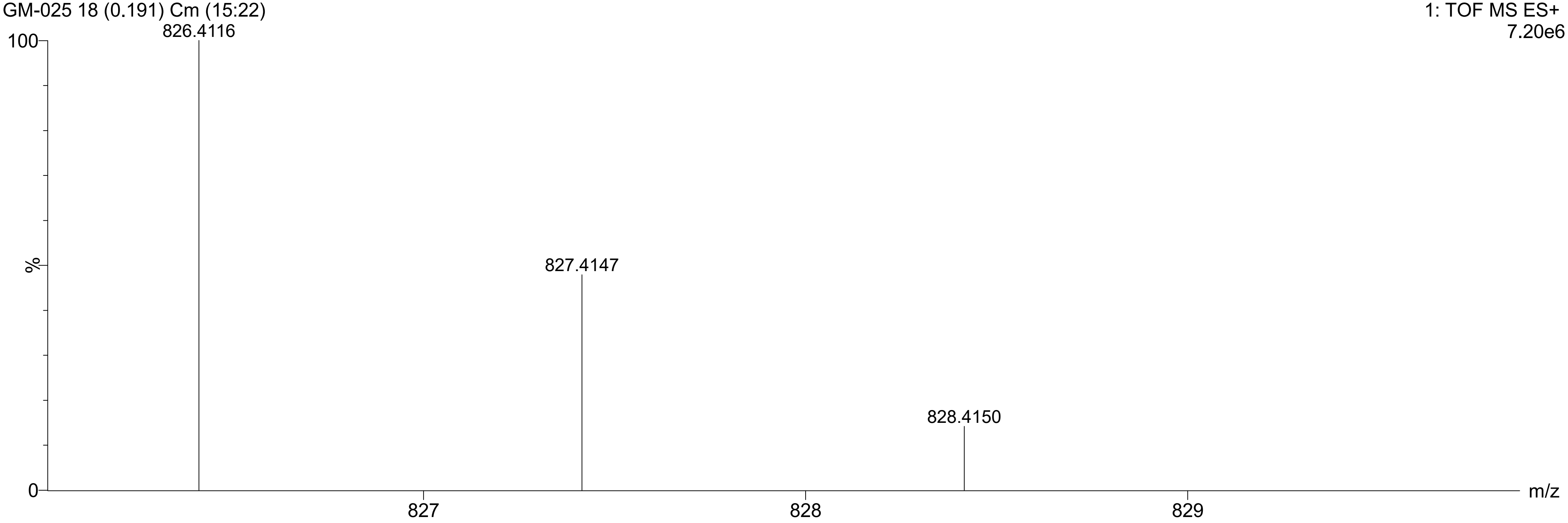

Compound 9h

29.08.2025

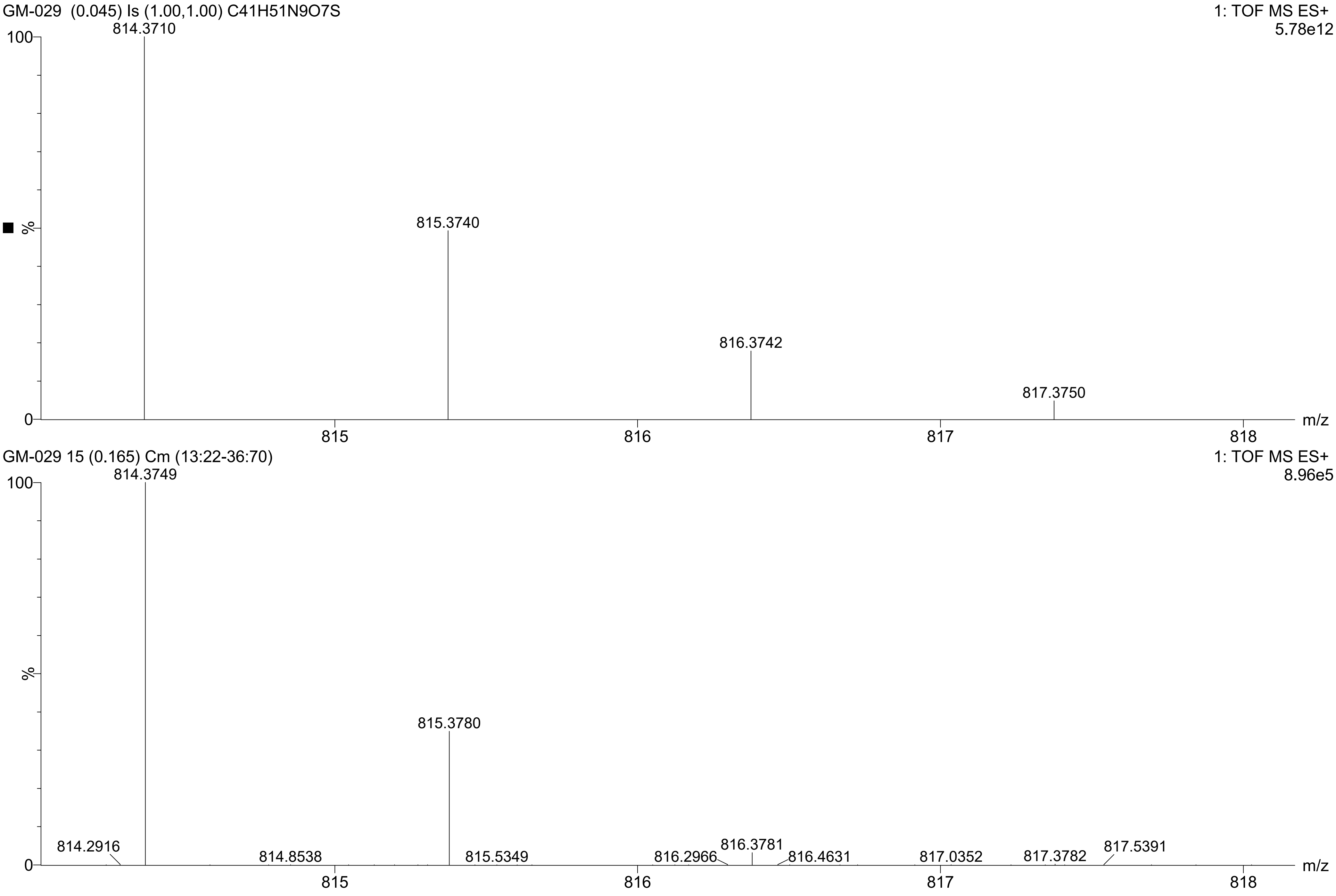

Compound 9i

12.09.2025

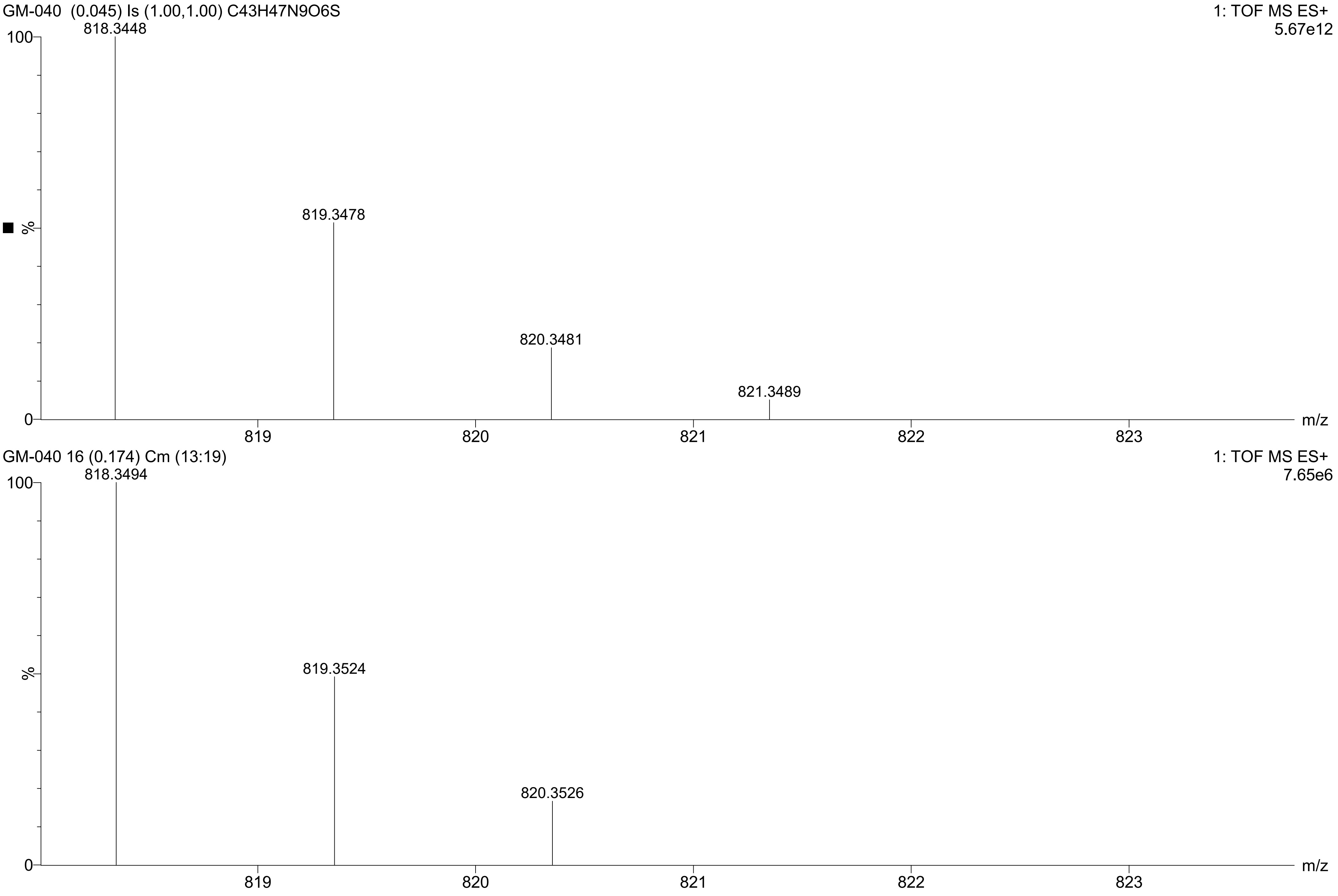

Compound 9j

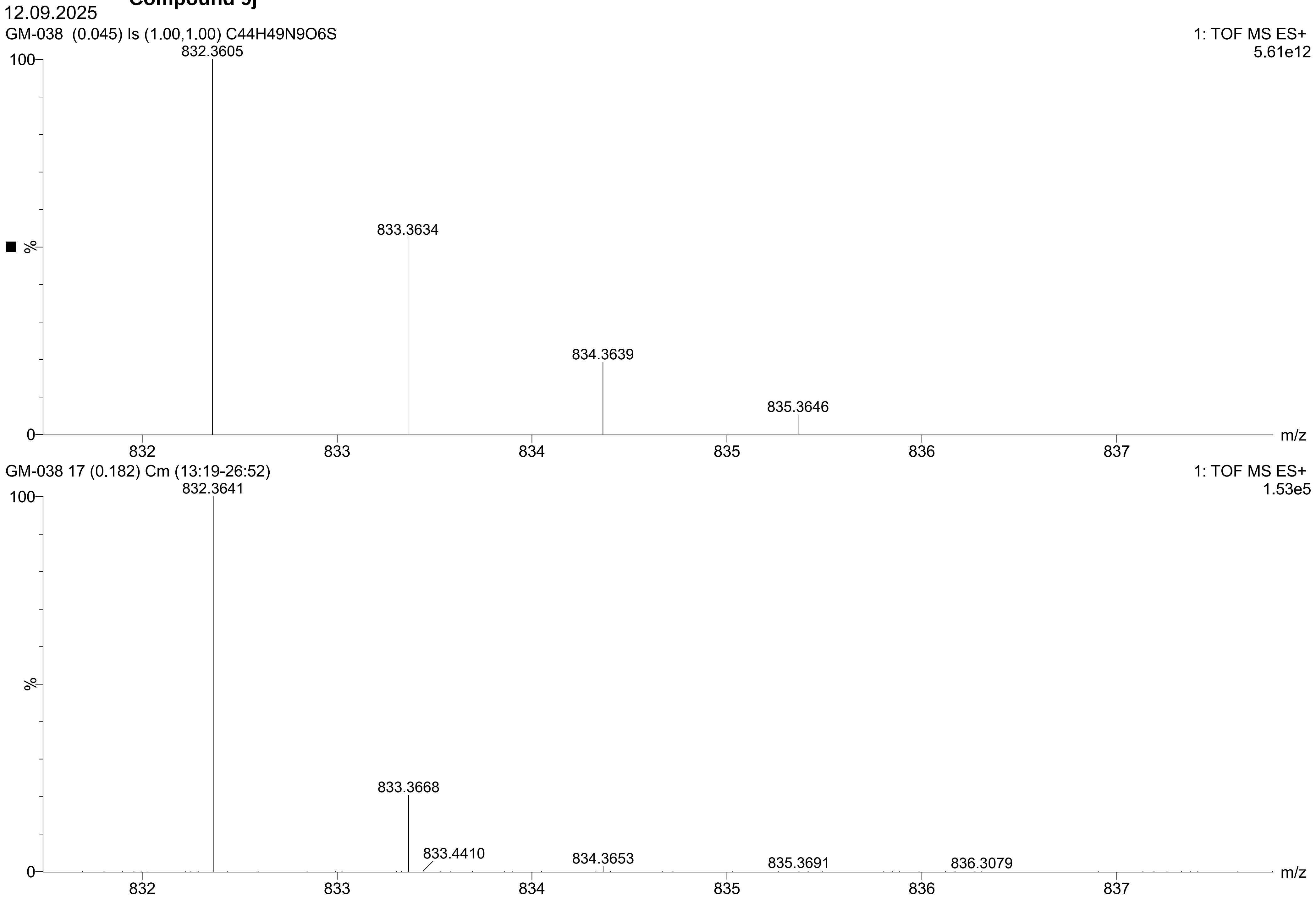

12.09.2025
GM-038 (0.045) Is (1.00,1.00) C44H49N9O6S
1: TOF MS ES+
5.61e12
832.3605
833.3634
834.3639
835.3646
100
%
0
832
833
834
835
836
837
m/z
GM-038 17 (0.182) Cm (13:19-26:52)
1: TOF MS ES+
1.53e5
832.3641
833.3668
833.4410
834.3653
835.3691
836.3079
100
%
0
832
833
834
835
836
837
m/z

Compound 9k

7.04.2025

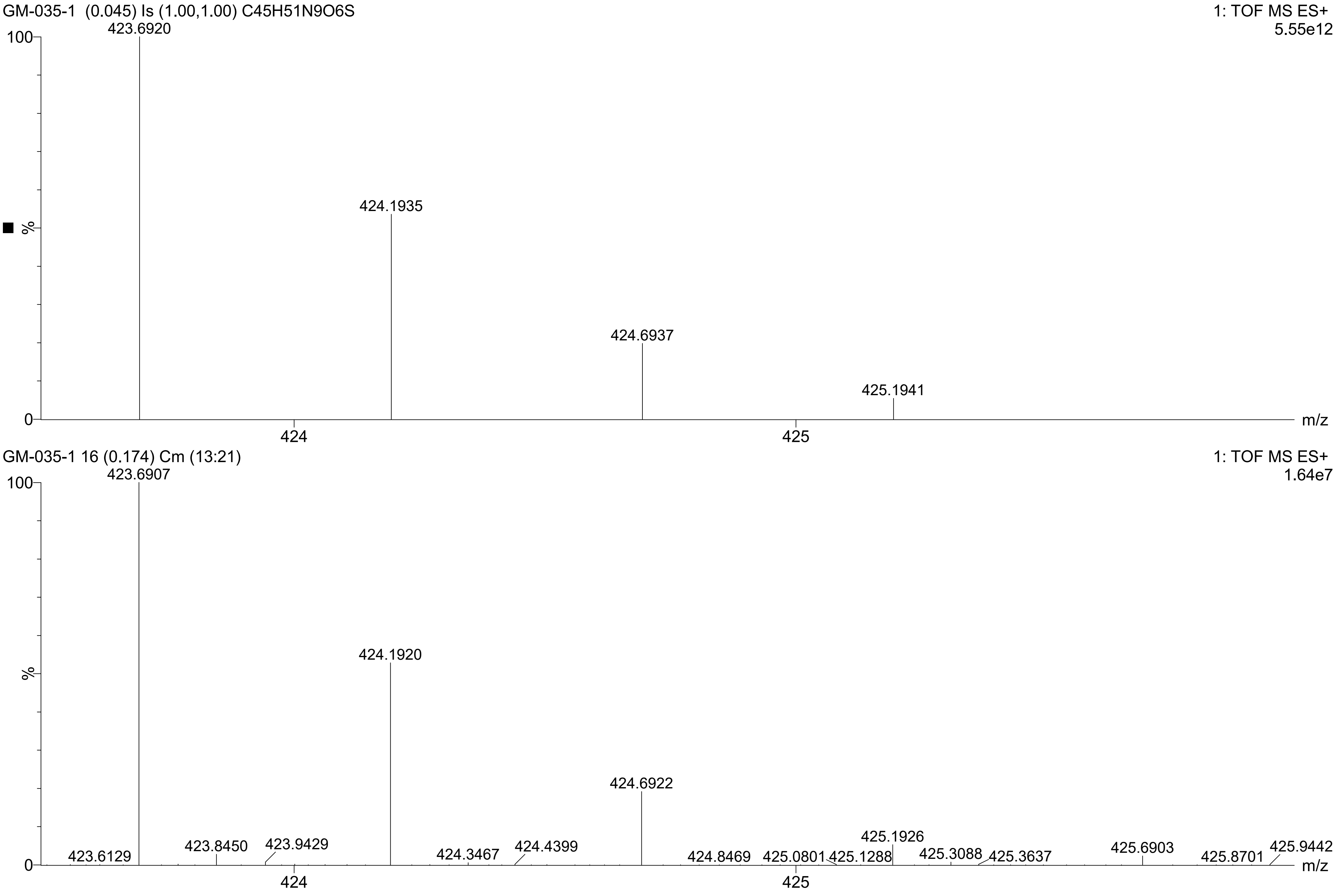

Compound 9l

29.08.2025

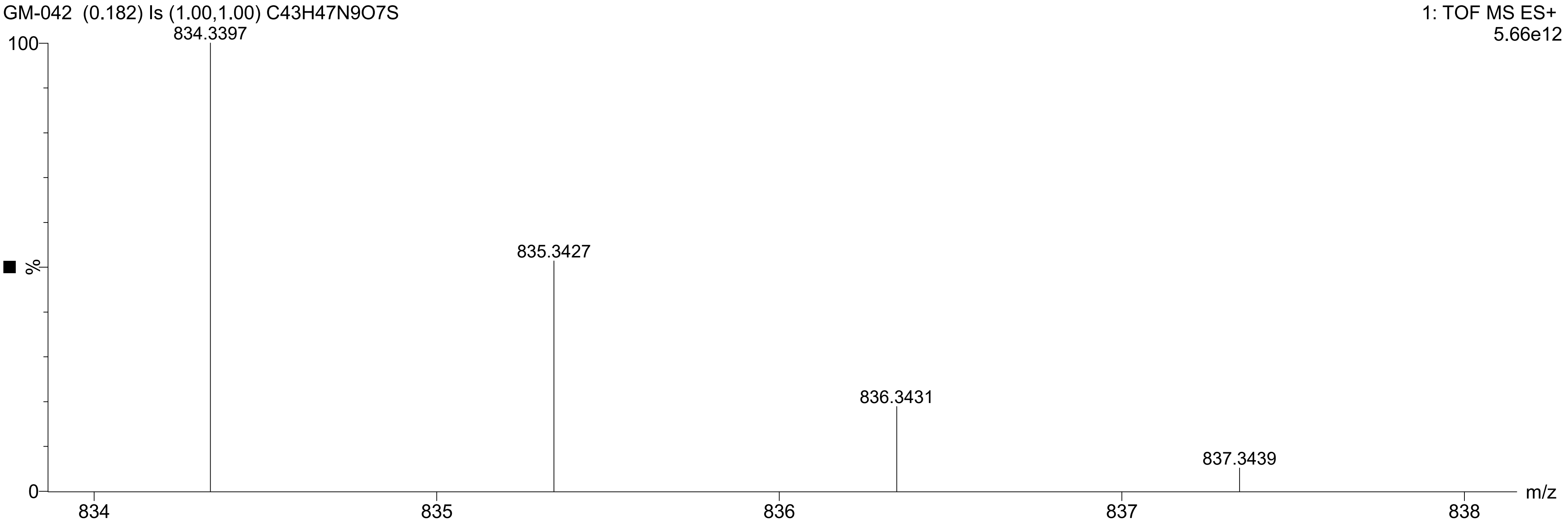

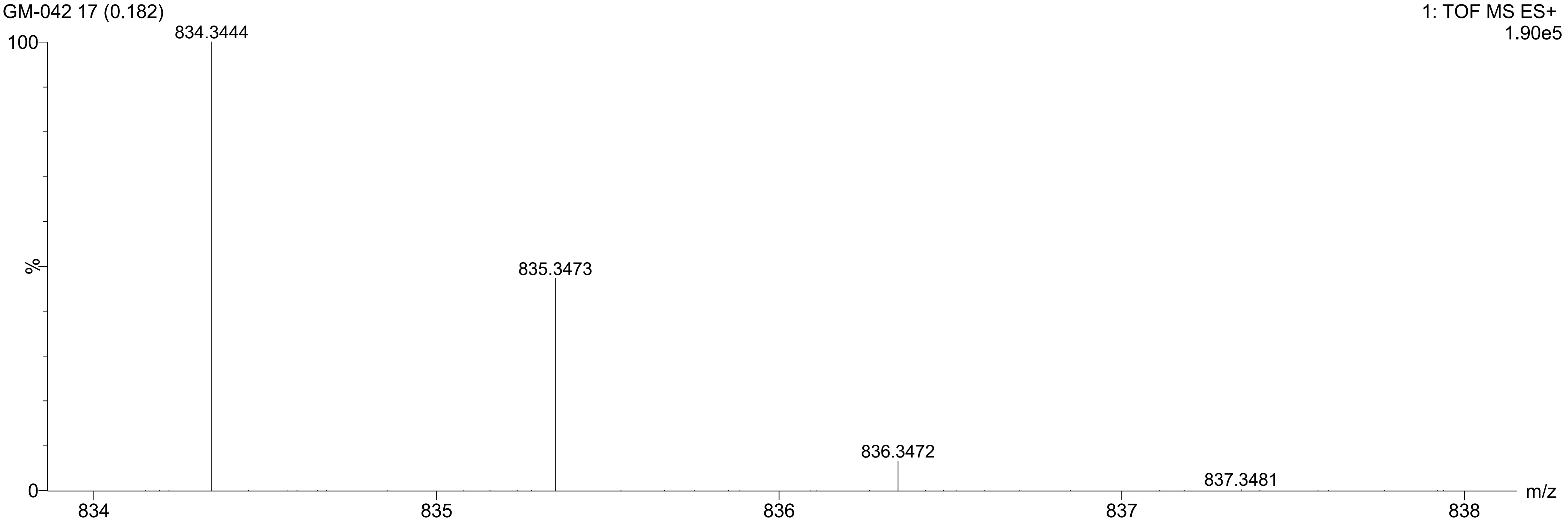

Compound 9m

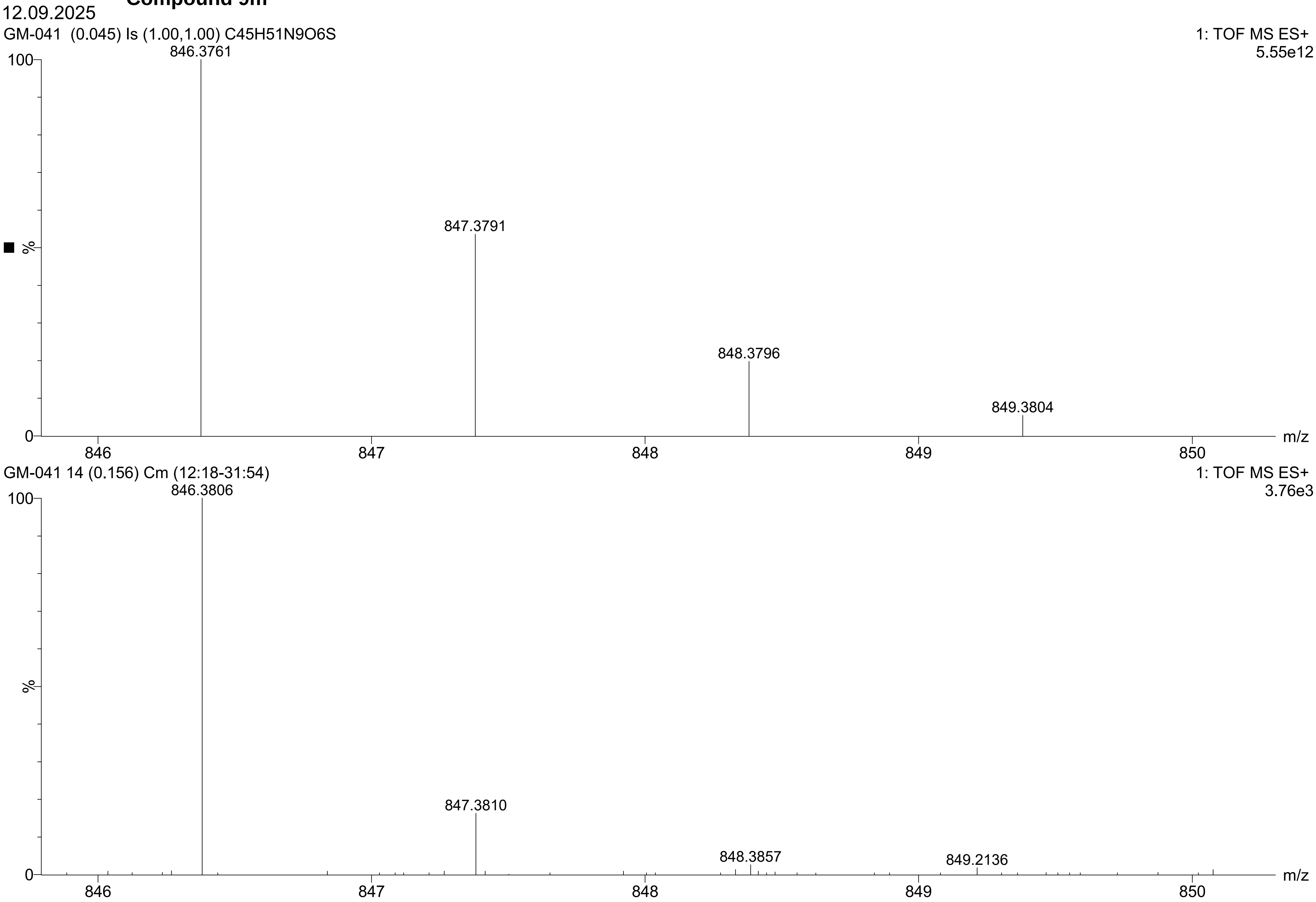
12.09.2025
GM-041 (0.045) Is (1.00,1.00) C45H51N9O6S
1: TOF MS ES+
5.55e12
846.3761
847.3791
848.3796
849.3804
100
%
0
846
847
848
849
850
m/z
GM-041 14 (0.156) Cm (12:18-31:54)
1: TOF MS ES+
3.76e3
846.3806
847.3810
848.3857
849.2136
100
%
0
846
847
848
849
850
m/z

Compound 9n

7.04.2025

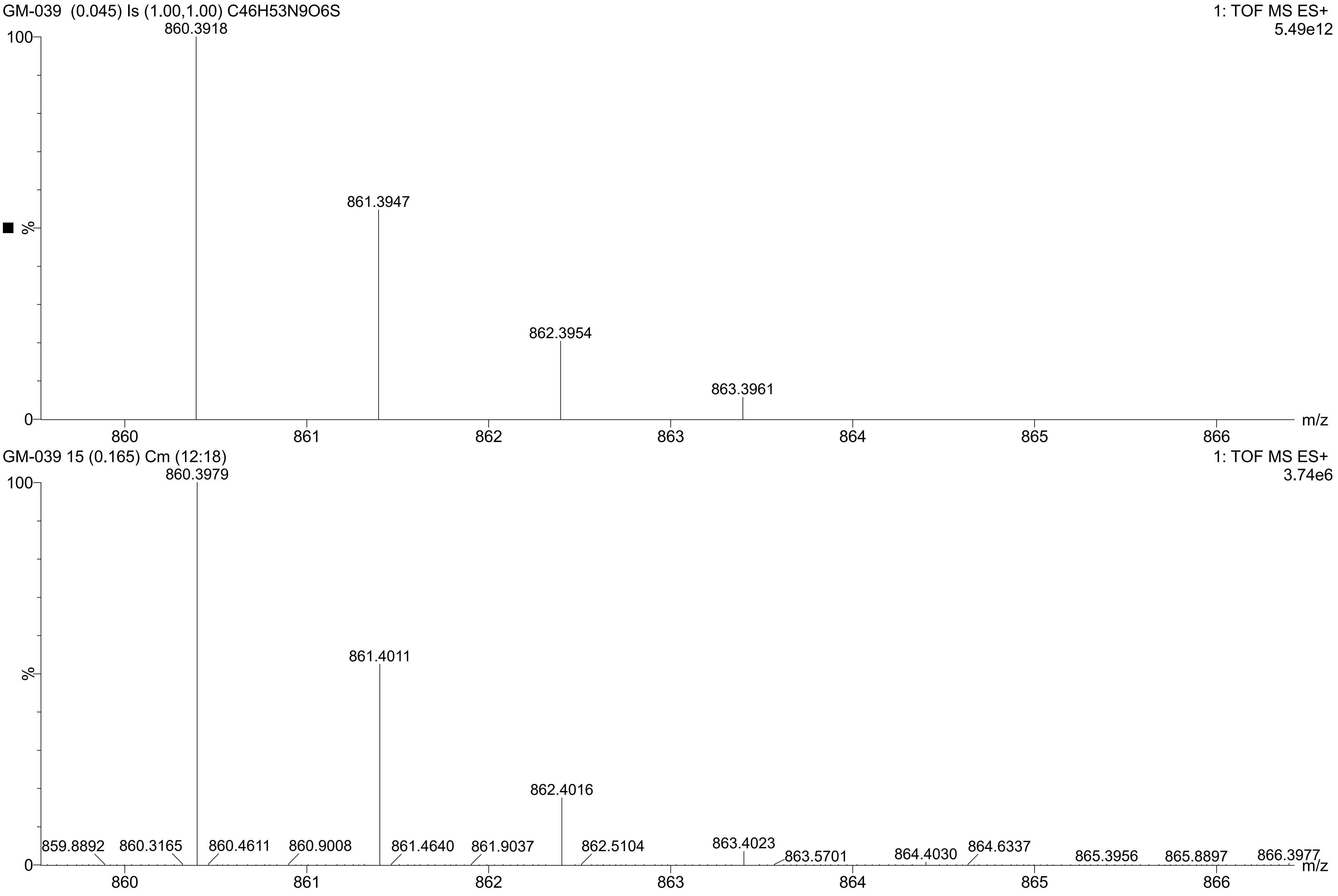

29.08.2025

Compound 9o

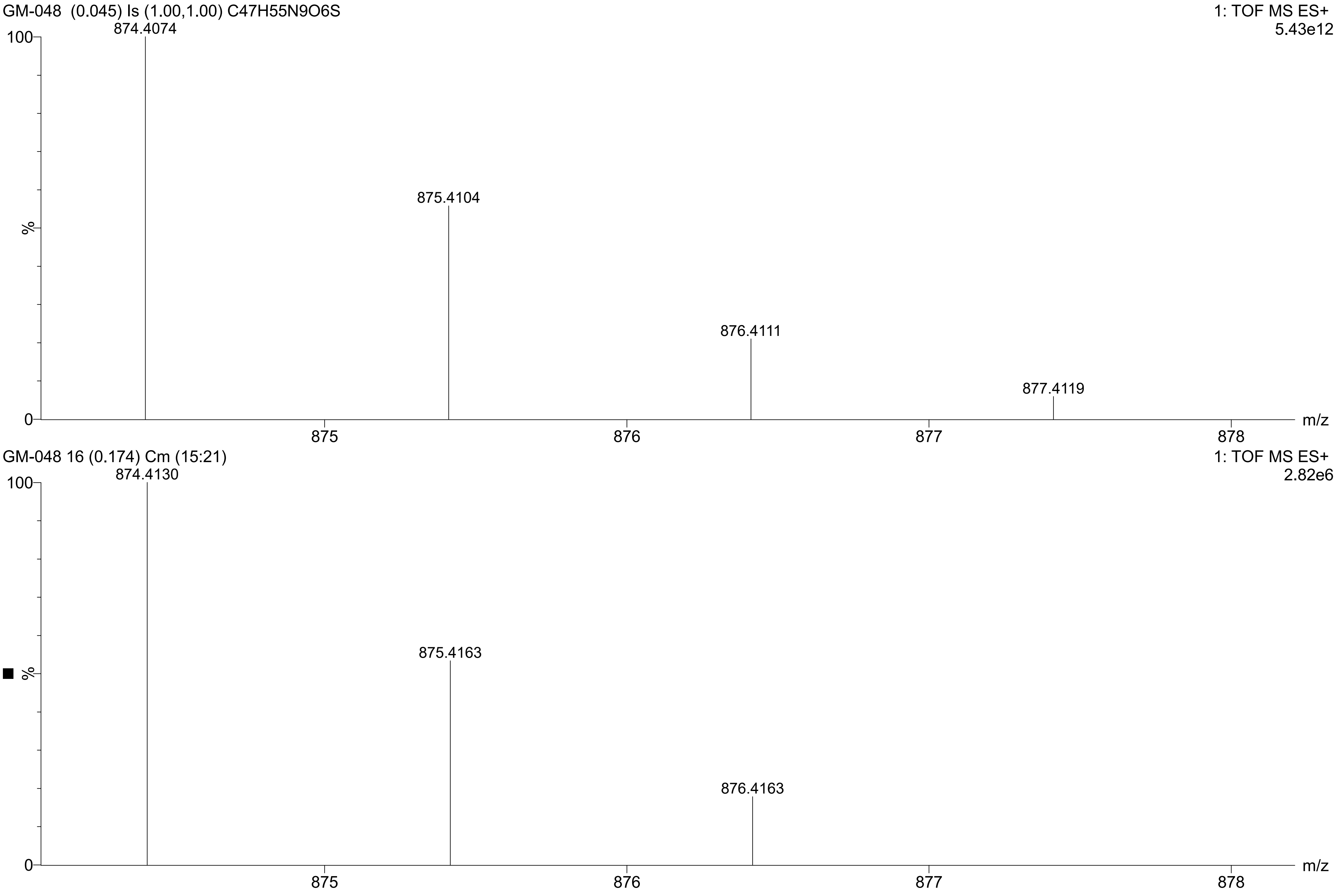

Compound 9p

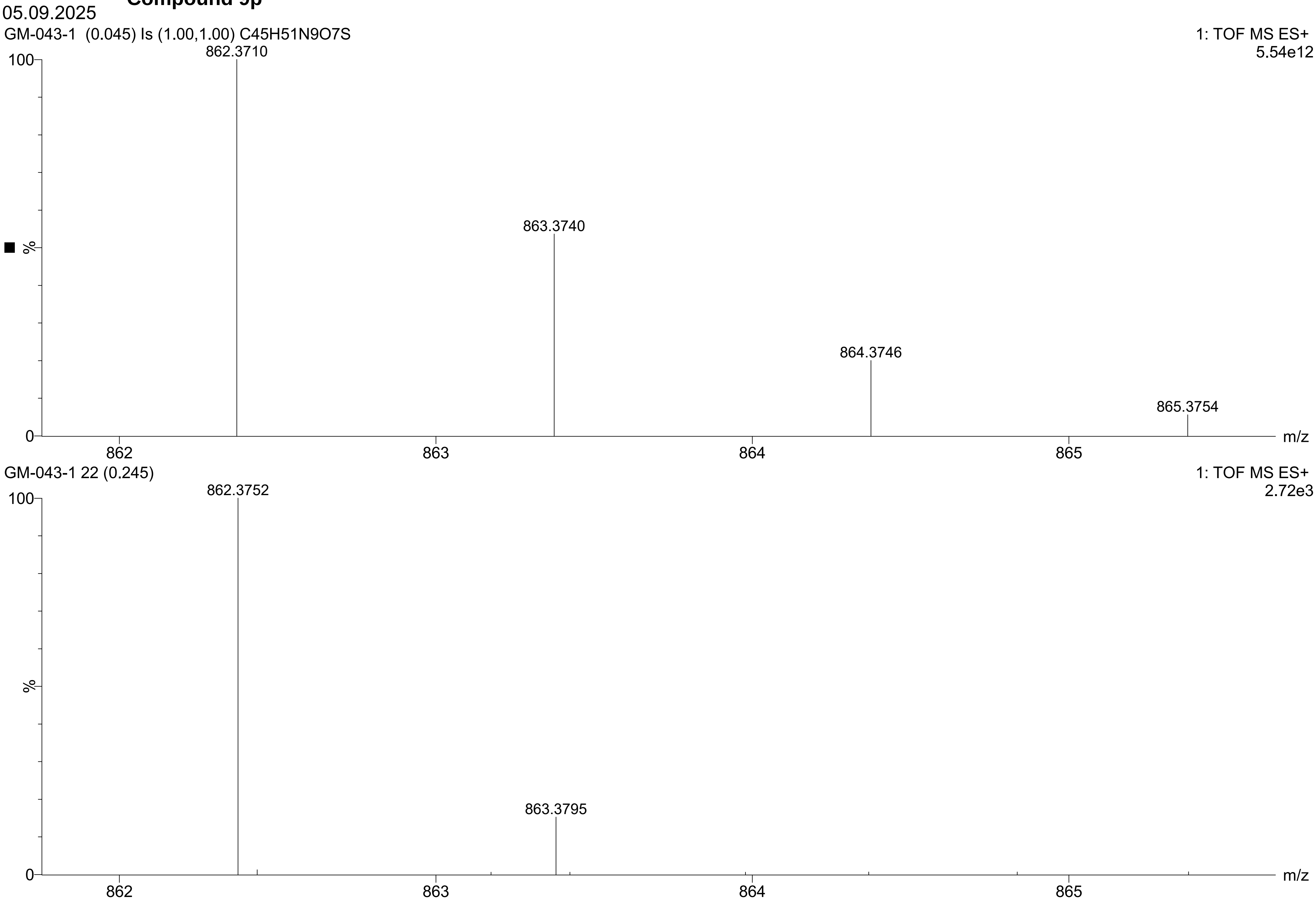

05.09.2025
GM-043-1 (0.045) Is (1.00,1.00) C45H51N9O7S
1: TOF MS ES+
5.54e12
862.3710
863.3740
864.3746
865.3754
100
%
0
862
863
864
865
m/z
GM-043-1 22 (0.245)
1: TOF MS ES+
2.72e3
862.3752
863.3795
100
%
0
862
863
864
865
m/z
